# Supplementary material for: Misinformation about medication during the COVID– 19 pandemic: A perspective of medical staff
Source: PLoS One. 2022 Oct 27;17(10):e0276693. doi: 10.1371/journal.pone.0276693 (PMC9612566; doi:10.1371/journal.pone.0276693)
Supplement: S1 Appendix — (DOCX) [file pone.0276693.s001.docx]

Communicating medical and non-medical information during the COVID 19 pandemic

Dear sir/madam

We invite you to participate in a study on the communication of medical and non-medical information during the Coronavirus pandemic. We are interested in knowing your opinion about the way the pandemic affected your work, the way the authorities communicated information about the virus and the drugs used to treat it, about the types of information regarding preventing and combating the virus, about the veracity of these information and the platforms used for communicating them.

You will be asked to answer the questions comprised in the questionnaire on the above mentioned topic, during 15 minutes.

You may only be vulnerable if personal data will be associated with the answers provided in the study. We assume full responsibility for the protection of personal data, for ensuring anonymity and confidentiality (no email addresses or other data through which you could be identified are collected).

Each participant has the right to withdraw at any time.

For any questions regarding this study, during or after its completion, please contact the principal researcher, Liliana Rogozea and the corresponding researcher, Claudiu Coman, at the e-mail addresses:

[r _liliana@unitbv.ro](mailto:r_liliana@unitbv.ro), [claudiu.coman@unitbv.ro](mailto:claudiu.coman@unitbv.ro)

Thank you for taking the time to answer our questionnaire!

*Mandatory

By checking the "YES" button you state that you have read the information mentioned above and that you agree to participate in this study. *

YES

A. To begin with, please answer a few questions about the way your professional activity was influenced by the COVID 19 pandemic

A1. What is the first word that comes into your mind when you think about the COVID 19 pandemic? *

A2. On a scale from 1 to 7, to what extent do you consider that the pandemic influenced the way you used to carry out your professional activity? *

1 2 3 4 5 6 7

To a very little extent To a very great extent

A3. What do you consider to be the main aspect of your professional activity that was influenced by the COVID 19 pandemic? *

Doctor - patient relationship Work schedule

Collaboration with colleagues

Other:

A4. What were the main difficulties you faced during the pandemic from a professional point of view? (Mention at least two) *

B.Next, please answer a few questions about how the authorities communicated information about the virus in general and about the medicines used to treat it.

B1. Taking into account the strategies adopted by the authorities to communicate information about the virus, how effective do you consider them? *

1 2 3 4 5 6 7

Extremely ineffective Extremely effective

B2. Taking into account the way in which the World Health Organization has communicated information regarding COVID 19, to what extent do you agree with the following statements? *

1

strongly disagree

2 3 4 5 6

7

strongly agree

WHO has coherently communicated information regarding the evolution of the virus.

There were contradictions in the information communicated by WHO about the effects of the drugs tested to treat the virus

WHO provides clear information on the state of development of a vaccine.

WHO concisely communicated the measures recommended to people in order to combat the virus

B3. Through what kind of methods do you think the way public authorities and health organizations communicate information to citizens during the pandemic could be improved? *

B4. Since the pandemic outbreak, the authorities provided information about various drugs tested and used to treat the virus. To what extent do you consider that this information has been coherently communicated ?

1 2 3 4 5 6 7

To a very little extent To a very great extent

B5. About which of the following drugs have you personally heard/seen that information was reported so far? *

Amoxicillin Azithromycin

Chloroquine, Hydroxychloroquine Dexamethasone

Doxycycline Favipiravir Ibuprofen Lopinavir/Ritonavir

Oseltamivir, Peramivir or Zanamivir Remdesivir

Tocilizumab Umifenovir

Other

B6. About which of the following drugs have you heard that they had positive effects in treating the virus? *

Amoxicillin Azithromycin

Chloroquine, Hydroxychloroquine Dexamethasone

Doxycycline Favipiravir Ibuprofen Lopinavir/Ritonavir

Oseltamivir, Peramivir or Zanamivir Remdesivir

Tocilizumab

Umifenovir

Other:

B7. About which of the following drugs have you heard that they hadn't had positive effects in treating the virus? *

Amoxicillin Azithromycin

Chloroquine, Hydroxychloroquine Dexamethasone

Doxycycline Favipiravir Ibuprofen Lopinavir/Ritonavir

Oseltamivir, Peramivir or Zanamivir Remdesivir

Tocilizumab Umifenovir

Other

B8. How effective do you believe it is in treating the virus, the method of using drugs that have had positive results in the past on similar viruses? *

1 2 3 4 5 6 7

Extremely ineffective Extremely effective

B9. On what kind of communication channels did you came across information about the drugs used to treat the virus? *

TV

Radio

On the websites of the authorities

On the official websites of health organizations

On social media

Other:

B10. How satisfied are you with the way information about drugs used in order to treat the virus has been communicated nationally? *

1 2 3 4 5 6 7

Extremely dissatisfied Extremely satisfied

B11. Which drugs do you believe could have beneficial effects in treating the virus?

*

C. Next, please answer a few questions regarding the way non-validated information about methods to prevent or treat the virus were communicated during the pandemic.

C1. To what extent do you consider that social networks are a suitable environment for communicating official information about COVID 19? *

1 2 3 4 5 6 7

To a very little extent To a very great extent

C2. To what extent do you consider that social networks contributed to the spread of false medical information during the pandemic? *

1 2 3 4 5 6 7

To a very little extent To a very great extent

C3. The main types of messages you encountered on social networks regarding the COVID 19 virus are messages about: *

Ways of spreading the virus Ways of preventing the virus Medical treatments

Alternative treatments

Other:

C4. Since the outbreak of the pandemic, various messages have been spread about methods of preventing and treating the virus. Which of the following methods have you personally heard about? *

Drinking alcohol helps you eliminate the virus

Drinking alcohol prevents the infection with the virus

Rinsing the nostrils with disinfectant eliminates the virus

Drinking hot water every 15 minutes eliminates the virus because it passes into the stomach

Pointing hot air to the nostrils leads to the elimination of the virus

Other:

C5. To what extent do you think these methods could have beneficial effects in treating the virus? *

1 2 3 4 5 6 7

To a very little extent To a very great extent

C6. Taking into account their harm potential, you consider that the alternative methods of preventing and treating the virus: *

Affect the individual's health

Do not affect the individual's health

I don't know/I'm not answering

C7. Given the phenomenon of fake news, to what extent do you consider that these methods represent the subject of false news? *

1 2 3 4 5 6 7

To a very little extent To a very great extent

C8. Taking into account the communication channels, do you consider that most messages about these alternative methods can be found: *

In the declarations of the authorities

On news bulletins on TV

On radio broadcasts

On news websites

On the official websites of public authorities

On social networks

Other:

C9. Mention how often have you encountered information about alternative methods of preventing and treating the virus: *

*.*

1

extremely rare

2 3 4 5 6

7

extremely often

In the declarations of authorities

On news bulletins on TV

On radio broadcasts

On news websites

On the official websites of public authorities

On social networks

C10. Personally, you received most messages about alternative methods for preventing and treating the virus: *

On Facebook

On Twitter

On WhatsApp

As a message on the preinstalled app of the phone By e-mail

Other:

C11. You received these messages from *

Acquaintances

Close friends Family members

Work colleagues

Other:

C12.Given the sources of the messages describing these methods of preventing and treating the virus, to what extent do you think they have influenced the behavior of citizens? *

1 2 3 4 5 6 7

To a very little extent To a very great extent

C13. In your opinion, who should be in charge of stopping the spread of medical information that is not validated? *

Health organizations The authorities

Medical specialists

Every type of communication channel

Other:

C14. To what extent do you consider that the dissemination of information about alternative methods of preventing and treating the virus has affected the credibility of the information communicated by doctors? *

1 2 3 4 5 6 7

To a very little extent To a very great extent

C15. In your opinion, how effective are the fake news prevention functions implemented by various social networks? *

1 2 3 4 5 6 7

Extremely ineffective Extremely effective

C16. In addition to information about the prevention and treatment of the virus, various conspiracy theories appeared in the media. Which of the following theories did you encounter information about? *

The virus was created on purpose in a lab

The virus was created by Bill Gates

The "global occult" is the one that manages the evolution of the virus

By vaccinating the population, chip integration is also intended

5G satellites are responsible for the spread of the virus

Other:

C17. By what kind of methods do you believe citizens could protect themselves against the fake news about the virus, that are spread during the pandemic? *

By checking information and consulting only official sources

By reporting messages that contain information which is not validated

By not sharing with other people the non-validated information they have received

Other:

C18. What kind of actions do you think the authorities should take in order to combat fake news about the virus? *

Developing of an official platform for communicating information about the virus

Developing collaboration programs with various social networks

Communicating information about the virus in a unitary and correct way

Other:

C19. How could doctors get involved in the process of combating fake news about the virus? *

C20. Personally, if you had the power to decide, what would be the first three strategies you would adopt in order to manage the information crisis generated by the COVID 19 pandemic? *

D. Lastly, please provide answers to some demographic data

D1. Gender *

Male Female

D2. Age *

D3. You live in the *

Rural area Urban area

D4. Professional degree *

If you are not a student, after answering this question please continue with question D7. If you are a student, please continue with question D6.

Senior Specialist-Medical Doctor Specialist-Medical Doctor Residency doctor

Pharmacist

Nurse with higher education diploma

Nurse with other education than university degree Biochemist, Biolog working in clinical labs

Clinical laboratory assistant, medical lab technicians

Physiotherapist

Social worker Psychologist Journalist

Student at university nursing programme

Medical student

Student at university programme in pharmacy

Student at university programme in social assistance Student at university programme in physiotherapy

Student at university programme for laboratory assistant, medical lab technicians

Student in psychology

Other:

D5. Year of study *

D6. Field of specialization *

D7. In what type of medical unit do you carry out your activity? *

Hospital Polyclinic Medical office Pharmacy University Other:

D8. Do you work in a medical unit/section where COVID 19 patients are treated? *

Yes No

D9. Your experience with the treatment for COVID 19 infection is *

Related to professional activity, being involved in the treatment and care of patients with COVID

Related to personal experience (my own person or people from close entourage) who have been treated for COVID infection

Thank you for your answers!
