# Supplementary material for: Misinformation about medication during the COVID– 19 pandemic: A perspective of medical staff
Source: PLoS One. 2022 Oct 27;17(10):e0276693. doi: 10.1371/journal.pone.0276693 (PMC9612566; doi:10.1371/journal.pone.0276693)
Supplement: S2 Appendix — (DOCX) [file pone.0276693.s002.docx]

Transmiterea de informații medicale și non-medicale în timpul pandemiei de COVID 19

Stimată doamnă/stimate domn,

Vă invităm să participați la un studiu privind transmiterea de informații medicale și non- medicale în timpul pandemiei de Coronavirus. Suntem interesați să cunoaștem opiniile dumneavoastră privind modul în care pandemia a influențat activitatea dumneavoastră profesională, despre modul în care autoritățile au comunicat informații despre virus și despre medicamentele utilizate în tratarea acestei infectii, despre tipurile de informații referitoare la prevenirea și combaterea virusului, despre veridicitatea acestor informații și platformele utilizate pentru transmiterea lor.

Veți fi solicitat/ă să răspundeți la întrebările cuprinse în chestionarul pe tema mai sus menționată, pe durata a 15 minute.

Puteți fi vulnerabil/ă doar dacă datele personale vor fi asociate cu răspunsurile furnizate în cadrul studiului. Ne asumăm întreaga responsabilitate pentru protejarea datelor personale, pentru asigurarea anonimatului și a confidențialității (nu se colectează adrese de e-mail sau alte date prin care ați putea fi identificat).

Fiecare participant are dreptul să se retragă în orice moment.

Pentru orice nelămurire legată de acest studiu, în timpul sau după finalizarea sa, vă rugăm să contactați cercetătoriul principal, Liliana Rogozea și cercetătorul corespondent, Claudiu Coman, la adresele de e-mail r [_liliana@unitbv.ro](mailto:r_liliana@unitbv.ro), [claudiu.coman@unitbv.ro](mailto:claudiu.coman@unitbv.ro)

Vă mulțumim!

*Obligatoriu

Prin bifarea butonului "DA" ați luat la cunoștință informațiile din acest formular și sunteți de acord cu participarea la acest studiu. *

DA

A. Pentru început, vă rugăm să răspundeți la câteva întrebări referitoare la modul în care activitatea dumneavoastră profesională a fost influențată de pandemia de COVID 19.

A1. Care este primul cuvânt care vă vine în minte atunci când vă gândiți la pandemia de COVID 19? *

A2. Pe o scală de la 1 la 7 în ce măsură considerați că pandemia a influențat modul în care obișnuiați să vă desfășurați activitatea profesională? *

1 2 3 4 5 6 7

În măsură extrem de mică În măsură extrem de mare

A3. Care considerați că este principalul aspect al activității dumneavoastră profesionale pe care l-a influențat pandemia de COVID 19? *

Relația medic-pacient Programul de lucru Colaborarea cu colegii Altele:

A4. Care considerați că au fost principalele dificultăți cu care v-ați confruntat în timpul pandemiei din punct de vedere profesional? (Menționați minim două) *

B. În continuare, vă rugăm să răspundeți la câteva întrebări referitoare la modul în care autoritățile au comunicat informații despre virus în general și despre medicamentele utilizate în tratarea sa.

B1. Având în vedere strategiile de comunicare adoptate de autorități în vederea transmiterii informațiilor referitoare la virus, cât de eficiente considerați că sunt acestea? *

1 2 3 4 5 6 7

Extrem de ineficiente Extrem de eficiente

B2. Luând în considerare modul în care Organizația Mondială a Sănătății a comunicat informații referitoare la COVID 19, în ce măsură sunteți de acord cu următoarele afirmații? *

1

7 acord

dezacord puternic

2 3 4 5 6

puternic

OMS a comunicat în mod coerent informații despre evoluți

Au existat contradicții în informațiile comunicate de OMS despre efectele medicamentelor testate pentru a trata virusul.

OMS oferă informații clare despre stadiul în care se află dezvoltarea unui vaccin.

OMS a comunicat concis măsurile recomandate populației în vederea combaterii virusului.

B3. Prin ce fel de metode considerați că ar putea fi îmbunătățit modul în care autoritățile publice și organizațiile din domeniul sănătății comunică informații către cetățeni în perioada pandemiei? *

B4. De la declanșarea pandemiei autoritățile au transmis informații despre diverse medicamente testate și utilizate în tratarea virusului. În ce măsură considerați că aceste informații au fost comunicate în mod coerent? *

1 2 3 4 5 6 7

În măsură extrem de mică În măsură extrem de mare

B5. Dumneavoastră personal, despre dintre următoarele medicamente utilizate în tratarea virusului ați auzit/văzut că au fost comunicate informații până în prezent? *

Amoxicilină Azitromicină

Clorochină, Hidroxiclorochină Dexametazonă

Doxicilină Favipiravir Ibuprofen Lopinavir/Ritonavir

Oseltamivir, Peramivir sau Zanamivir Remdesivir

Tocilizumab Umifenovir

Altele:

B6. Despre care dintre aceste medicamente ați auzit că au avut efecte pozitive în tratarea virusului? *

Amoxicilină Azitromicină

Clorochină, Hidroxiclorochină Dexametazonă

Doxicilină Favipiravir Ibuprofen Lopinavir/Ritonavir

Oseltamivir, Peramivir sau Zanamivir Remdesivir

Tocilizumab Umifenovir

Altele:

B7. Despre care dintre următoarele medicamente ați auzit că nu au avut efecte pozitive în tratarea virusului? *

Amoxicilină Azitromicină

Clorochină, Hidroxiclorochină Dexametazonă

Doxicilină Favipiravir Ibuprofen Lopinavir/Ritonavir

Oseltamivir, Peramivir sau Zanamivir Remdesivir

Tocilizumab Umifenovir

Altele:

B8. Cât de eficientă considerați că este pentru tratarea virusului, metoda utilizării unor medicamente care au avut în trecut rezultate pozitive asupra unor virusuri similare? *

1 2 3 4 5 6 7

Extrem de ineficientă Extrem de eficientă

B9. Pe ce fel de canale de comunicare ați întâlnit informații despre medicamentele utilizate în tratarea virusului? *

TV

Radio

Pe site-urile oficiale ale autorităților

Pe site-urile oficiale ale organizațiilor din domeniul sănătății

Pe rețelele sociale

Altele:

B10. Cât de mulțumit sunteți de modul în care au fost comunicate la nivel național informații despre medicamentele utilizate în tratarea virusului? *

1 2 3 4 5 6 7

Extrem de nemulțumit Extrem de mulțumit

B11. Care medicamente considerați dumneavoastră că ar putea avea efecte benefice în tratarea virusului? *

C. În continuare, vă rugăm să răspundeți la câteva întrebări referitoare la modul în care s-au comunicat în timpul pandemiei informații nevalide despre metode de prevenire și tratare a virusului.

C1. În ce măsură considerați că rețelele sociale reprezintă un mediu potrivit pentru transmiterea de informații oficiale despre COVID 19? *

1 2 3 4 5 6 7

În măsură extrem de mică În măsură extrem de mare

C2. În ce măsură considerați că rețelele sociale au contribuit la răspândirea informațiilor medicale false în perioada pandemiei? *

1 2 3 4 5 6 7

În măsură extrem de mică În măsură extrem de mare

C3. Principalele tipuri de mesaje pe care pe care le-ați întâlnit pe rețelele sociale despre virusul COVID 19 sunt mesaje despre: *

Modalități de transmitere

Modalități de prevenire

Tratamente medicamentoase

Tratamente alternative

Altele:

C4. De la declanșarea pandemiei au început să fie răspândite diverse mesaje despre metode de prevenire și tratare a virusului. Dumneavoastră personal despre care dintre următoarele metode ați auzit? *

Consumul de spirt ajută la eliminarea virusului Consumul de alcool previne infectarea

Clătirea nărilor cu dezinfectant elimină virusul

Consumul de apă caldă din 15 în 15 minute elimină virusul deoarece acesta trece în stomac

Îndreptarea spre căile nazale a feonului cu aer cald duce la eliminarea virusului Altele:

C5. În ce măsură considerați că aceste metode pot avea efecte benefice în tratarea virusului? *

1 2 3 4 5 6 7

În măsură extrem de mică În măsură extrem de mare

C6. Având în vedere gradul de periculozitate al acestor metode, considerați mai degrabă că metodele alternative de prevenire și tratare a virusului: *

*.*

Afectează sănătatea individului

Nu afectează sănătatea individului

Nu știu/Nu răspund

C7. Având în vedere fenomenul fake news, în ce măsură considerați că aceste metode reprezintă subiectul unor știri false? *

1 2 3 4 5 6 7

În măsură extrem de mică În măsură extrem de mare

C8. Având în vedere mijloacele de comunicare, considerați că cele mai multe mesaje despre astfel de metode se regăsesc: *

În declarațiile autorităților

În buletinele de știri la TV

În emisiunile radio

Pe site-urile de știri

Pe site-urile oficiale ale autorităților

Pe rețele sociale

Altele:

C9. Menționați cât de des ați întâlnit dumneavoastră informații despre metode alternative de prevenire și tratare a virusului: *

1 extrem 2 3 4 5 6

de rar

7

extrem de des

În declarațiile

autorităților

În buletinele de știri

la TV

În emisiuni radio

Pe site-uri de știri

Pe site-urile oficiale

ale autorităților

Pe rețelele sociale

C10. Dumneavoastră personal ați primit cele mai multe mesaje despre metode alternative pentru prevenirea și eliminarea virusului: *

Pe Facebook Pe Twitter

Pe WhatsApp

Ca mesaj în aplicația preinstalată a telefonului Pe mail

Altele:

C11. Aceste mesaje le-ați primit de la: *

Cunoștințe

Prieteni apropiați Membrii familiei Colegi de muncă

Altele:

C12. Având în vedere proveniența mesajelor care descriu aceste metode de prevenire și tratare a virusului, în ce măsură considerați că ele au influențat comportamentul cetățenilor? *

1 2 3 4 5 6 7

În măsură extrem de mică În măsură extrem de mare

C13. Cine considerați că ar trebui să se ocupe de stoparea transmiterii de informații medicale care nu sunt validate? *

*Marcați un singur oval.*

Oragnizațiile din domeniul sănătății

Autoritățile

Specialiștii din domeniul medical

Fiecare canal de comunicare în parte

Altele:

C14. În ce măsură considerați că vehicularea informațiilor despre metode alternative de prevenire și tratare a virusului a afectat credibilitatea informațiilor transmise de medici? *

1 2 3 4 5 6 7

În măsură extrem de mică În măsură extrem de mare

C15. Cât de eficiente considerați că sunt funcțiile de prevenire a știrilor false implementate de diverse rețele sociale? *

1 2 3 4 5 6 7

Extrem de ineficiente Extrem de eficiente

C16. Pe lângă informațiile despre prevenirea și tratarea virusului, au apărut în media și diverse teorii ale conspirației. Despre care dintre următoarele teorii ați întâlnit și dumneavoastră informații? *

Virusul a fost creat intenționat în laborator

Virusul a fost creat de Bill Gates

”Oculta mondială” este cea care gestionează evoluția virusului

Prin vaccinarea populației se intenționează și integrarea de cipuri Antenele 5G sunt responsabile pentru răspândirea virusului

Altele:

C17. Prin ce fel de metode considerați că ar putea cetățenii să se apere de știrile false despre virus răspândite în perioada pandemiei? *

Prin verificarea informației și consultarea surselor oficiale Prin raportarea mesajelor care prezintă informații nevalide

Prin a nu distribui mai departe informațiile nevalide pe care le primesc Altele:

C18. Ce fel de acțiuni considerați că ar trebui să realizeze autoritățile pentru a combate știrile false referitoare la virus? *

Dezvoltarea unei platforme oficiale de comunicare a informațiilor despre virus Dezvoltarea unor programe de colaborare cu diverse rețele sociale

Comunicarea informațiilor despre virus într-un mod unitar și corect

Altele:

C19. Cum ar putea să se implice medicii în combaterea știrilor false care se referă la virus? *

C20. Dacă dumneavoastră ați avea putere de decizie, care ar fi primele trei strategii pe care le-ați adopta pentru a gestiona această criză informațională generată de pandemia de COVID 19? *

D. În final, vă rugăm să ne oferiți câteva date de identificare

D1. Genul dumneavoastră *

Maculin Feminin

D2. Vârsta în ani împliniți *

D3. Țara de reședință *

D4. Mediul de proveniență *

Rural Urban

D5. Grad profesional *

Dacă Nu sunteți student, după această întrebare vă rugăm să treceți la înrebarea D7. Dacă sunteți student, vă rugăm să continuați cu întrebarea D6.

Medic primar Medic specialist Medic rezident Farmacist

Asistent medical cu studii superioare

Asistent medical cu PL

Biochimist, Biolog din laboratoare medicale Asistent laborator clinic

Fiziokinetoterapeut; balneofiziokinetoterapeut (studii in domeniul medical)

Asistent social

Psiholog

Jurnalist

Student programul de medicină

Student programul de asistență medicală generală Student la programul de farmacie

Student la programul de asistenta sociala

Student Fiziokinetoterapeut; balneofiziokinetoterapeut (studii in domeniul medical) Student programul de laborator clinic

Student psihologie Altele:

D6. Anul de studiu

D7. Domeniul de specialitate *

D8. Unde vă desfășurați activitatea profesionala? *

*Marcați un singur oval.*

Spital

Policlinică Cabinet medical Farmacie Universitate Altele:

D9. Lucrați într-o unitate medicală/pe o secție unde se tratează pacienți cu COVID- 19? *

Da Nu

D10. Experiența privind tratamentul pentru infecția COVID 19 este: *

Legată de activitatea profesională, fiind implicat în tratarea și îngrijirea pacienților cu COVID

Legată de experiența personală (propria persoană sau persoane din anturajul apropiat) care au fost tratate pentru infecție COVID

Altele:

Vă mulțumim pentru disponibilitatea de a răspunde!
