# Supplementary material for: Misinformation about medication during the COVID– 19 pandemic: A perspective of medical staff
Source: PLoS One. 2022 Oct 27;17(10):e0276693. doi: 10.1371/journal.pone.0276693 (PMC9612566; doi:10.1371/journal.pone.0276693)
Supplement: S1 Table — (DOCX) [file pone.0276693.s003.docx]

**S3 Tables with results to the 2^nd^ research question**

**Table A.** Drugs known to have positive effects in treating the virus: the perception of medical staff

|  | Frequency | Valid percent |
| --- | --- | --- |
| Amoxicillin | 36 | 6.7% |
| Azithromicin | 206 | 38.4% |
| Chloroquine, Hydroxychloroquine | 124 | 23.1% |
| Dexamethasone | 250 | 46.6% |
| Doxycycline | 32 | 6.0% |
| Favipiravir | 74 | 13.8% |
| Ibuprofen | 106 | 19.8% |
| Lopinavir/Ritonavir | 56 | 10.4% |
| Oseltamivir, Peramivir or Zanamivir | 32 | 6.0% |
| Remdesivir | 217 | 40.5% |
| Tocilizumab | 85 | 15.9% |
| Umifenovir | 17 | 3.2% |

| **Table B.** Medical staff’s knowledge about alternative methods of preventing and treating the virus | | | | | |
| --- | --- | --- | --- | --- | --- |
|  | | Frequency | Percent | Valid Percent | Cumulative Percent |
| Valid | drinking alcohol helps you eliminate the virus | 79 | 14.7 | 14.7 | 14.7 |
|  | drinking alcohol prevents the infection with the virus | 130 | 24.3 | 24.3 | 39.0 |
|  | rinsing the nostrils with disinfectant eliminates the virus | 81 | 15.1 | 15.1 | 54.1 |
|  | drinking hot water every 15 minutes eliminates the virus | 114 | 21.3 | 21.3 | 75.4 |
|  | pointing hot air to the nostrils leads to the elimination of the virus | 90 | 16.8 | 16.8 | 92.2 |
|  | other | 42 | 7.8 | 7.8 | 100.0 |
|  | Total | 536 | 100.0 | 100.0 |  |
