# Supplementary material for: Misinformation about medication during the COVID– 19 pandemic: A perspective of medical staff
Source: PLoS One. 2022 Oct 27;17(10):e0276693. doi: 10.1371/journal.pone.0276693 (PMC9612566; doi:10.1371/journal.pone.0276693)
Supplement: S2 Table — (DOCX) [file pone.0276693.s004.docx]

**S4 Tables with results to the 3^rd^ research question**

| **Table C.** Perception about the efficiency of communication strategies adopted by authorities | | | | | |
| --- | --- | --- | --- | --- | --- |
|  | | Frequency | Percent | Valid Percent | Cumulative Percent |
| Valid | Extremely inefficient | 22 | 4.1 | 4.1 | 4.1 |
|  | very inefficient | 38 | 7.1 | 7.1 | 11.2 |
|  | inefficient | 84 | 15.7 | 15.7 | 26.9 |
|  | nor efficient, neither inefficient | 126 | 23.5 | 23.5 | 50.4 |
|  | efficient | 134 | 25.0 | 25.0 | 75.4 |
|  | very efficient | 80 | 14.9 | 14.9 | 90.3 |
|  | extremely efficient | 52 | 9.7 | 9.7 | 100.0 |
|  | Total | 536 | 100.0 | 100.0 |  |
