# Supplementary material for: Misinformation about medication during the COVID– 19 pandemic: A perspective of medical staff
Source: PLoS One. 2022 Oct 27;17(10):e0276693. doi: 10.1371/journal.pone.0276693 (PMC9612566; doi:10.1371/journal.pone.0276693)
Supplement: S3 Table — (DOCX) [file pone.0276693.s005.docx]

**S5 Tables with results to the 4^th^ research question**

| **Table D.**  Perception about the extent to which social media represents an appropriate environment for sharing official COVID – 19 information | | | | | |
| --- | --- | --- | --- | --- | --- |
|  | | Frequency | Percent | Valid Percent | Cumulative Percent |
| Valid | to an extremely little extent | 86 | 16.0 | 16.0 | 16.0 |
|  | to a very little extent | 78 | 14.6 | 14.6 | 30.6 |
|  | to a little extent | 52 | 9.7 | 9.7 | 40.3 |
|  | nor to a little, neither to a great extent | 78 | 14.6 | 14.6 | 54.9 |
|  | to a great extent | 72 | 13.4 | 13.4 | 68.3 |
|  | to a very great extent | 74 | 13.8 | 13.8 | 82.1 |
|  | to an extremely great extent | 96 | 17.9 | 17.9 | 100.0 |
|  | Total | 536 | 100.0 | 100.0 |  |
