# Supplementary material for: Misinformation about medication during the COVID– 19 pandemic: A perspective of medical staff
Source: PLoS One. 2022 Oct 27;17(10):e0276693. doi: 10.1371/journal.pone.0276693 (PMC9612566; doi:10.1371/journal.pone.0276693)
Supplement: S4 Table — (DOCX) [file pone.0276693.s006.docx]

**S6 Tables with results to the 5^th^ research question**

| **Table E. The aspect of professional life which was most influenced by the pandemic** | | | | | |
| --- | --- | --- | --- | --- | --- |
|  | | Frequency | Percent | Valid Percent | Cumulative Percent |
| Valid | patient – doctor relationship | 206 | 38.4 | 38.4 | 38.4 |
|  | work schedule | 144 | 26.9 | 26.9 | 65.3 |
|  | collaboration with peers | 128 | 23.9 | 23.9 | 89.2 |
|  | other | 58 | 10.8 | 10.8 | 100.0 |
|  | Total | 536 | 100.0 | 100.0 |  |

| **Table F.** Main aspect of professional life influenced by the pandemic * professional degree - Cross tabulation | | | | |
| --- | --- | --- | --- | --- |
|  | | Professional degree^1^ | | Total |
|  |  | Medical staff | Student |  |
| A3^2^. Main aspect of professional life influenced by the pandemic | patient – doctor relationship | 144 | 62 | 206 |
|  | work schedule | 70 | 74 | 144 |
|  | collaboration with peers | 62 | 66 | 128 |
|  | other | 18 | 40 | 58 |
| Total | | 294 | 242 | 536 |

^1^Index variable from the professional degrees of respondents. Student: medical student and student at university nursing program, Medical Staff: Senior specialist medical – doctor, Specialist medical – doctor, Resident, Nurse with higher education diploma, Nurse with other studies than higher education

^2^A3 – refers to question 3 from the section A of the manuscript (Main aspect of professional life influenced by the pandemic), section which refers to Influence of the pandemic on the professional activity of medical staff
