# Supplementary material for: Misinformation about medication during the COVID– 19 pandemic: A perspective of medical staff
Source: PLoS One. 2022 Oct 27;17(10):e0276693. doi: 10.1371/journal.pone.0276693 (PMC9612566; doi:10.1371/journal.pone.0276693)
Supplement: Attachment — Submitted filename: PONE-D-22-09134_R1.pdf [file pone.0276693.s007.pdf]

PLOS ONE

Challenges in the communication process during the COVID-19 pandemic- a perspective of medical staff

--Manuscript Draft--

Manuscript Number:	PONE-D-22-09134R1
Article Type:	Research Article
Full Title:	Challenges in the communication process during the COVID-19 pandemic- a perspective of medical staff
Short Title:	Challenges in the communication process during the COVID-19 pandemic
Corresponding Author:	Claudiu Coman<br>Universitatea Transilvania din Brasov<br>Brasov, ROMANIA
Keywords:	Miscommunication; healtcare professionals; trust; Covid - 19 pandemic
Abstract:	Background Healthcare professionals had to face numerous challenges during the pandemic, their professional activity being influenced not only by the virus, but also by the spread of medical misinformation. In this regard, we aimed to analyze, from the perspective of medical staff, the way medical and non - medical information about the virus was communicated during the pandemic in order to raise awareness about the way misinformation affected the medical staff. Methods and findings. The study was conducted on Romanian healthcare professionals including doctors, nurses and medical students. They were asked to answer to a questionnaire and the sample of the research includes 536 respondents. The findings revealed that most respondents stated that information about alternative treatments against the virus affected the credibility of health professionals, and that younger professionals believed to a greater extent that trust in doctors was affected. The research also showed that respondents were well informed about the drugs used in clinical trials in order to treat the virus, and that younger respondents believed that social media should be used to send official information. Among the main limitations of our study we mention the fact that we used only quantitative methods and the fact we focused only on Romanian healthcare professionals. Conclusions Healthcare professionals declared that the spread of misinformation regarding alternative treatments, affected their credibility and the relationship with their patients. Healthcare professionals had knowledge about the drugs used in clinical trials, and they acknowledged the role of social media in spreading medical misinformation. However, younger professionals also believed that social media could be used to share official information about the virus. A future research should focus on studying the opinion of Romanian and international doctors, it should use qualitative methods too and should address the issue of social media being an appropriate environment for sending official information.
Order of Authors:	Claudiu Coman
	Maria Cristina Bularca
	Angela Repanovici
	Liliana Rogozea
Response to Reviewers:	We took into account the comments of the editor and we uploaded again our supporting information in a zip format.<br>The platform required us to upload a version of the manuscript with track changes but we were not required to make any changes to the manuscript.<br>The comments of the editor metioned only to upload our supporting information in zip format.
Additional Information:	
Question	Response
Financial Disclosure	The authors received no specific funding for this work.

Powered by Editorial Manager® and ProduXion Manager® from Aries Systems Corporation

Enter a financial disclosure statement that describes the sources of funding for the work included in this submission. Review the submission guidelines for detailed requirements. View published research articles from *PLOS ONE* for specific examples.<br><br>This statement is required for submission and **will appear in the published article** if the submission is accepted. Please make sure it is accurate.<br><br>**Unfunded studies**<br>Enter: *The author(s) received no specific funding for this work.*<br><br>**Funded studies**<br>Enter a statement with the following details:<br>• Initials of the authors who received each award<br>• Grant numbers awarded to each author<br>• The full name of each funder<br>• URL of each funder website<br>• Did the sponsors or funders play any role in the study design, data collection and analysis, decision to publish, or preparation of the manuscript?<br>• **NO** - Include this sentence at the end of your statement: *The funders had no role in study design, data collection and analysis, decision to publish, or preparation of the manuscript.*<br>• **YES** - Specify the role(s) played.<br><br>* typeset	
**Competing Interests**<br><br>Use the instructions below to enter a competing interest statement for this submission. On behalf of all authors, disclose any competing interests that could be perceived to bias this work—acknowledging all financial support and any other relevant financial or non-financial competing interests.<br><br>This statement is required for submission and **will appear in the published article** if the submission is accepted. Please make	The authors have declared that no competing interests exist.

sure it is accurate and that any funding sources listed in your Funding Information later in the submission form are also declared in your Financial Disclosure statement.<br><br>View published research articles from *PLOS ONE* for specific examples.<br><br>**NO authors have competing interests**<br><br>Enter: *The authors have declared that no competing interests exist.*<br><br>**Authors with competing interests**<br><br>Enter competing interest details beginning with this statement:<br><br>*I have read the journal's policy and the authors of this manuscript have the following competing interests: [insert competing interests here]*<br><br>* typeset	
**Ethics Statement**<br><br>Enter an ethics statement for this submission. This statement is required if the study involved:<br><br>• Human participants<br>• Human specimens or tissue<br>• Vertebrate animals or cephalopods<br>• Vertebrate embryos or tissues<br>• Field research<br><br>Write "N/A" if the submission does not require an ethics statement.<br><br>General guidance is provided below. Consult the submission guidelines for detailed instructions. **Make sure that all information entered here is included in the Methods section of the manuscript.**	APPROVAL OF THE COUNCIL OF THE FACULTY OF SOCIOLOGY AND COMMUNICATION<br>Approval request: Nr.378/30.03.2021<br>Form of consent obtained: oral

**Format for specific study types**<br><br>**Human Subject Research (involving human participants and/or tissue)**<br>• Give the name of the institutional review board or ethics committee that approved the study<br>• Include the approval number and/or a statement indicating approval of this research<br>• Indicate the form of consent obtained (written/oral) or the reason that consent was not obtained (e.g. the data were analyzed anonymously)<br><br>**Animal Research (involving vertebrate animals, embryos or tissues)**<br>• Provide the name of the Institutional Animal Care and Use Committee (IACUC) or other relevant ethics board that reviewed the study protocol, and indicate whether they approved this research or granted a formal waiver of ethical approval<br>• Include an approval number if one was obtained<br>• If the study involved *non-human primates*, add *additional details* about animal welfare and steps taken to ameliorate suffering<br>• If anesthesia, euthanasia, or any kind of animal sacrifice is part of the study, include briefly which substances and/or methods were applied<br><br>**Field Research**<br><br>Include the following details if this study involves the collection of plant, animal, or other materials from a natural setting:<br>• Field permit number<br>• Name of the institution or relevant body that granted permission	
**Data Availability**<br><br>Authors are required to make all data underlying the findings described fully available, without restriction, and from the time of publication. PLOS allows rare exceptions to address legal and ethical concerns. See the PLOS Data Policy and FAQ for detailed information.	Yes - all data are fully available without restriction

A Data Availability Statement describing where the data can be found is required at submission. Your answers to this question constitute the Data Availability Statement and **will be published in the article**, if accepted.<br><br>**Important:** Stating 'data available on request from the author' is not sufficient. If your data are only available upon request, select 'No' for the first question and explain your exceptional situation in the text box.<br><br>Do the authors confirm that all data underlying the findings described in their manuscript are fully available without restriction?	
**Describe where the data may be found in full sentences. If you are copying our sample text, replace any instances of XXX with the appropriate details.**<br><br>• If the data are **held or will be held in a public repository**, include URLs, accession numbers or DOIs. If this information will only be available after acceptance, indicate this by ticking the box below. For example: *All XXX files are available from the XXX database (accession number(s) XXX, XXX.)*.<br>• If the data are all contained **within the manuscript and/or Supporting Information files**, enter the following: *All relevant data are within the manuscript and its Supporting Information files.*<br>• If neither of these applies but you are able to provide **details of access elsewhere**, with or without limitations, please do so. For example:<br><br>*Data cannot be shared publicly because of [XXX]. Data are available from the XXX Institutional Data Access / Ethics Committee (contact via XXX) for researchers who meet the criteria for access to confidential data.*<br><br>*The data underlying the results presented in the study are available from (include the name of the third party*	All relevant data are within the manuscript and its Supporting Information files.

and contact information or URL).<br>• This text is appropriate if the data are owned by a third party and authors do not have permission to share the data.<br><br>* typeset	
Additional data availability information:	

Manuscript Click here to access/download;Manuscript;Manuscript.docx

Challenges in the communication process during the COVID-19 pandemic- a perspective of medical staff

Claudiu Coman[1#a*], Maria Cristina Bularca[1], Angela Repanovici[2], Liliana Rogozea[3]

1 Department of Social Sciences and Communication, Faculty of Sociology and Communication, Transilvania University of Brasov, Brasov, Romania;

2 Department of Product Design, Mechatronics and Environment, Faculty of Product Design and Environment, Transilvania University of Brasov, Brasov, Romania

3 Basic, Preventive and Clinical Sciences Department, Transilvania University of Brasov, Brasov, Romania;

[#a] Current address: Department of Social Sciences and Communication, Faculty of Sociology and Communication, Transilvania University of Braşov, Brasov, România

[*] Corresponding author

E-mail: claudiu.coman@unitbv.ro (CC)

Abstract

Background. Healthcare professionals had to face numerous challenges during the pandemic, their professional activity being influenced not only by the virus, but also by the spread of medical misinformation. In this regard, we aimed to analyze, from the perspective of medical staff, the way medical and non - medical information about the virus was communicated during the pandemic in order to raise awareness about the way misinformation affected the medical staff.

Methods and findings. The study was conducted on Romanian healthcare professionals including doctors, nurses and medical students. They were asked to answer to a questionnaire and the sample of the research includes 536 respondents. The findings revealed that most respondents stated that information about alternative treatments against the virus affected the credibility of health professionals, and that younger professionals believed to a greater extent that trust in doctors was affected. The research also showed that respondents were well informed about the drugs used in clinical trials in order to treat the virus, and that younger respondents believed that social media should be used to send official information. Among the main limitations of our study we mention the fact that we used only quantitative methods and the fact we focused only on Romanian healthcare professionals.

Conclusions. Healthcare professionals declared that the spread of misinformation regarding alternative treatments, affected their credibility and the relationship with their patients. Healthcare professionals had knowledge about the drugs used in clinical trials, and they acknowledged the role of social media in spreading medical misinformation. However, younger professionals also believed that social media could be used to share official information about

the virus. A future research should focus on studying the opinion of Romanian and international doctors, it should use qualitative methods too and should address the issue of social media being an appropriate environment for sending official information.

Introduction

The COVID 19 pandemic generated multiple changes in the way today's society members carry out their daily activities. While many domains were affected by the spread of the virus, such as the educational system or the cultural sector, the health sector was the one that faced the most challenges, the pandemic managing to generate a tremendous global public health crisis [1].

Caused by severe acute respiratory syndrome coronavirus 2 (SARS-CoV-2) [2], the disease was firstly detected in December 2019, in Wuhan, China [3], and it fastly spread all over the world. The World Health Organization was informed about a pneumonia outbreak in Wuhan on December 31 2019, the number of cases continued to increase, and on March 11 2020 the World Health Organization characterized COVID 19 as a pandemic [4]. Being highly contagious, the virus affected a large number of people, and as of November 27 over 61 million cases were reported [5]. Even though many companies and institutions are struggling to develop a vaccine, Pfizer, Gamaleya Research Institute, University of Oxford, and a preliminary analysis of the vaccine proposed by Pfizer showed that the vaccine is able to prevent more than 90% of people from getting infected with COVID 19 [6], so far no vaccine was approved as a general and universal vaccine against COVID 19 [7].

Ever since the pandemic was declared, finding the right treatment for the virus has become a priority for researchers and doctors from all over the world. In this regard, large

number of trials started to be conducted, and in order to find an efficient drug treatment against the virus, one method that was adopted was testing and administrating to patients, drugs that were previously used for curing other viruses [8]. Thus, on March 20 2020, The World Health Organization launched the SOLIDARITY clinical trial, a trial that monitored the effects on patients infected with COVID 19, of specific drugs that proven to be effective in the treatment of other diseases: remdesivir, interferon beta, chloroquine and hydroxychloroquine - previously used for Malaria, as well as drugs used on HIV patients: lopinavir and ritonavir [9]. However, according to the interim results published on October 15 2020 by WHO, even though those drugs were taught to have positive effects on treating COVID 19, they had little influence or no influence at all on mortality in general, on the need and initiation of ventilation and on the recovery process [10].

With the development of many trials and programs meant to find a cure for COVID 19 and with the use of diverse drug combinations, another major problem arose: misinformation and fake news about the virus, its treatment or methods to combat it. In this regard, along with the pandemic, people also had to face an epidemic of information, described by the general director of WHO as an „infodemic” [11]. In other words, information about COVID 19 began to be spread by people on every available communication channel, both in the online and offline environment. However, very often and especially on social media, the information was poorly communicated, it was distorted and there usually wasn’t enough scientific evidence to demonstrate its validity [12].

Taking into account the previously mentioned aspects the paper addresses the issues of drugs tested and used for the treatment of COVID 19 and how information about COVID 19 was

communicated in the offline and online environment. The purpose of the paper is to analyze, from the perspective of medical staff, the way medical and non - medical information about the virus was communicated during the pandemic in order to raise awareness about the way misinformation affected medical staff. Thus, the paper aims at finding an answer to three research questions: (1) to what extent information about alternative treatments affected the credibility of medical staff? (2) What is the knowledge of medical staff about the type of drugs that had positive effects on treating the disease and about alternative treatments? (3) How satisfied is the medical staff with the way medical and non-medical information was communicated online and offline during the pandemic? (4) What is the perception of medical staff about the role of social media in spreading misinformation about the virus? (5) What aspects of the professional activity of the medical staff were affected most by the COVID – 19 pandemic?

Hence, considering the purpose of our paper and the research questions, we believed it was necessary to analyze the literature on the drugs used to treat COVID – 19, on the role of social media platforms in spreading fake information about the virus and potential treatments, and on the way the pandemic influenced the credibility of doctors and their relationship with their patients.

Literature review

Information on drugs used to treat COVID 19

Before analyzing the way information about the virus was communicated in the online environment, it is important to take a look at the drugs used to treat the disease. Hence, one of the most important issues that appeared with the COVID 19 pandemic, was finding the right

treatment for the virus. In this regard, researchers started to develop many experimental trials and used diversified drug combinations in order to treat patients with COVID 19. However, information that was communicated about the effectiveness of certain drugs was often contradictory.

Chloroquine and hydroxychloroquine are two drugs that were tested and included in many trials. Both drugs were previously used to treat malaria but they also have antiviral effects on viruses like HIV since they have the ability to prevent the virus to enter in the host cells [13]. Even though they have similar compounds, chloroquine is taught to have more negative effects than hydroxychloroquine [14], and hydroxychloroquine is considered safer due to the fact that it can be tolerated better for a longer period of time [15].

While some studies show positive effects of hydroxychloroquine in inhibiting the infection with the virus in vitro [16, 17], other studies found no influence of the drug on mortality rate or time spent by patients in the hospital [18]. However, when hydroxychloroquine was combined with other drugs such as azithromycin, it showed beneficial effects in treating patients with COVID 19 [19].

Nonetheless the findings regarded the effectiveness of these drugs were contrasting. For example, on March 28 2020 the Food and Drug Administration (FDA) issued an Emergency Use Authorization for using hydroxychloroquine in treating people suffering from COVID 19 [20], and in June 15 2020, the FDA retracted the authorization stating that the trials in which the drug was involved showed that the drug had no effect on the faster recovery of patients or on decreasing chances of death [21]. Even more, on 5th June 2020 the UK trial, Randomised Evaluation of COVID 19 THERAPY (RECOVERY), also stopped testing the drug on patients

because the results showed no benefits in improving the conditions of hospitalized patients with COVID 19 [22].

Studies were carried out with other drugs such as lopinavir/ritonavir, an antiviral drug used in the treatment of HIV [23]. While in concentration of 4 μg/ml and 50 μg/ml, the drug showed positive effects against the virus in vitro [24], a study on 199 patients, from which 99 received the drug and the other 100 did not receive the drug, revealed that lopinavir/ritonavir had no benefits when it comes to diminishing mortality or improving the state of patients with severe symptoms [25].

Controversial discussions also involved the use of Ibuprofen, a Non-steroidal anti-inflammatory drug that is used to treat fever, or inflammation [26]. Since the pandemic was declared there has been a preoccupation regarding ibuprofen and its role in making people more vulnerable to contacting the virus. Thus, right after the declaration of the pandemic, in a letter addressed to The Lancer Journal, researchers pointed out that ibuprofen could make people with diabetes, cardiac disease or hypertension more likely to get infected with virus and have severe symptoms [27]. However, while firstly, WHO recommended people who are infected with the virus not to take ibuprofen, only one day after that recommendation, on 18 March 2020, WHO corrected its statement and mentioned that it ”does not recommend against ibuprofen” [28]. Even more, a study focusing on the use of ibuprofen showed that the drug does not make patients feel worse [29] and another study that analyzed the use of ibuprofen and paracetamol of 403 COVID 19 confirmed patients revealed that compared to paracetamol, ibuprofen did not aggravated the clinical state of the patients [30].

While other drugs failed to show beneficial effects on the treatment of COVID 19, drugs like dexamethasone, which is included in the UK RECOVERY trial, revealed positive effects on people suffering from COVID 19: the drug lowered the risk of death in patients on ventilators from 40% to 28% and in patients who were in need of oxygen, from 25% to 20%, but did not influence the state of patients who did not need oxygen [31, 32].

Another highly tested drug was Remdesivir, an antiviral drug produced by Gilead Sciences that was previously used in treating Ebola [33]. The information regarding its positive effects on treating COVID 19 is also contradictory. A study conducted from February 6 2020 until March 12 2020, on 237 patients, showed that the drug did not bring any benefits for people that had severe symptoms of COVID 19 [34], while a more recent study revealed that Remdesivir had a more positive effect in reducing the time of recovery in patients with COVID 19 that showed signs of respiratory issues, than it had the placebo effect [35]. However, the FDA approved on October 22 2020, the use of Remdesivir in the case of adults and also children aged 12 or older who have at least 44 kilograms, who are infected with the virus and need to be treated in the hospital [36], and as of November 20 2020, FDA allows, in emergency cases, the use of Remdesivir in combination with Baricitinib, for adults and children aged two or older that require oxygen and treatment in the hospital [37].

Social media and COVID 19 misinformation

Together with the health crisis, the COVID 19 pandemic generated an information crisis, often described as an infodemic, that is represented by the spread of fake news, misguided and false information, especially in the online environment [38].

In this context, social media plays an essential role in disseminating information. Social media consists of internet based channels that provide people with the opportunity to interact, communicate in asynchronous way and in real time, with either small or large audiences where value is derived from user generated content [39]. Social media comprises multiple social networks, which according to Boyd and Ellison, offer users the possibility to create profiles that are public, or semi-public, to create a list of people with whom they can interact and share information and to view the list of connections that other users make [40].

Social media channels are often used in time of crisis not only by citizen, but also by official authorities, emergency services, because they can facilitate communication and the spread of valuable information that can contribute to surpassing the crisis [41]. Social networks like Facebook, Whatsapp, Twitter, Instagram can function as sources that have the ability to confirm or complete the information communicated by the authorities, while also receiving feedback from the public [42]. Thus, sending messages through social media channels is a strategy that can help authorities obtain feedback on certain proposals regarding public health policies [43]. Even more, a study regarding the influence of social media on the way people protect their health during the pandemic, showed that social media can have positive impact on increasing awareness about public health and protection against the virus [44].

However, during the pandemic, while authorities can use social media to keep the public informed, a major issue generated by social media, that public health representatives have to face, is the spread of fake news [45].

Fake news are represented by fabricated information designed in the form of news communicated by the media that do not share the same process of organization and do not

have the same intent, and fake news are related to misinformation: information that is false or misleading, and disinformation: a type of false information whose aim is to deceive people [46].

Thus, the internet became a favorable environment for spreading conspiracy theories or false information about alternative treatment for the virus. Since people were stressed and frightened by the uncertainty of the situation, they started to consider reasonable and valid any information that presented explanations in regards to the virus [47]. Thus, when referring to health information, false news often undermine the credibility of official sources, they create confusion among people and favor the faster spread of the virus [48].

Misinformation during the pandemic can negatively influence peoples' health because false information is not easy to recognize, because it can determine people to change their behavior in a way that is harmful to their health and those around them. Thus, since the pandemic was declared, false information has been spread about the origin of the virus, about what caused it, how it spreads and what treatment is efficient for eliminating it [49]. However, a study focusing on the WhatsApp platform showed that when the information on social media is shared by trusted sources, it can increase knowledge about the virus and encourage people to adopt preventive behavior [50].

During the time of crisis, on platforms like WhatsApp or Facebook, more and more false news and unverified information about the virus began to be shared. With millions of users worldwide, WhatsApp became one of the platforms where most fake news were shared by forwarding messages to many users [51], while Facebook was characterized as the core, epicenter of misinformation [52].

When it comes to health misinformation on social media, the most discussed subjects are alternative cures involving certain food or drinks, hygiene related actions and treatment drugs. Thus, among the most “recommended” practices for preventing or curing COVID were drinking hot water every 15 minutes in order for the virus to go into the stomach, eating garlic, taking vitamin C or even pointing a hairdryer to the nostrils because the heat could eliminate the virus [53].

False news that circulated on social media regarding the virus also involve the idea that the virus was created on purpose in a lab, three in ten Americans considering true this information [54].

However, many other unverified methods were shared and the most forwarded messages on WhatsApp presented information about the fact that if people hold their breath for ten seconds without coughing then they are not infected with the virus, about the idea that at temperatures of 30-35 Celsius degrees the virus will die, messages about the release of the vaccine or about drugs allegedly recommended by Chinese doctors that could be efficient in eliminating the virus [55].

Nonetheless, misinformation became a major issue in the context of the pandemic, but also a subject of interest for researchers. A study focusing on the spread of fake news showed that most news reconfigure and twist the original information thus creating a different context, and that most of them contain false information about public authorities and health organizations [56].

Another study found that people who tend to rely on their intuition or who possess little scientific knowledge about certain subjects, encountered difficulties in differentiating true and

false information [57]. Thus, misleading or unverified information can negatively influence the way people behave. For example, people in USA who died after they consumed chloroquine may have used the drug because news about it mentioned that it could treat and eliminate the virus [58]. Even more, a study concerning misinformation on Facebook revealed that posts made from verified accounts contained more false information than the accounts that were not verified [59], while other study conducted from 23 April 2020 to 27 April 2020, focused on perception about contradictory information and stated that 73% of participants mentioned they observed or were exposed to contrasting messages usually communicated by politicians or health experts [60].

Apart from influencing peoples' beliefs or health practices, COVID 19 fake news also influenced the activity of health professionals. Social media managed to increase the level of trust in information that comes from people's personal opinions rather than professionals [61], and doctor's credibility is often affected. In order to improve these situations, doctors must be willing to use social media not just to send messages, but to actively communicate with people, to offer feedback, to share their experiences and rectify and clarify the fake news presented on social media [62].

Among action from health professionals, in order to combat COVID 19 fake news, social media networks as well as public authorities must implement some strategies. For example, the government of United Kingdom developed collaboration programs between its rapid response teams and social media platforms, and Taiwan introduced greater fines for news that were proven to be false [63]. Moreover, even though some social networks such as Facebook or Twitter already implemented algorithms to identity and remove fake accounts [64], or to

correct information [65], they should further develop efficient strategies in order to validate the information that people share [66].

The influence of the pandemic on doctors' credibility and relationship with patients

The way information regarding the virus was communicated online and offline during the pandemic played an essential role in the process of maintaining trust in health professionals. In this regard, a previous longitudinal study conducted in Poland revealed that trust in physicians has declined from 2018 – 2020, and emphasized the idea that the decrease may be caused by the health problems that people had to cope with during the pandemic and the problems with the healthcare system of the country [67]. In Romanian context, a previous study showed that the communication process of the healthcare system was poor and confusing, and that public health authorities at national level focused more on global information about the virus, while local authorities failed to succeed in providing their "share of information" [68]. Another study, which focused on analyzing the online communication of Public Health Agencies from Italy, United States and Sweden, revealed that compared to Sweden and the United States, agencies from Italy collaborated more with other organizations, and that overall, the communication process of the agencies was coordinated by their members, that agencies also communicated with governments, but they rarely collaborated with political or non-governmental organizations [69]. Hence, while trust in the government and communication from authorized organizations is essential, the importance of trusting the professionals is highlighted by a study conducted in Thailand, which showed that in the cases in which people have low levels of trust

in the government, trust in professionals can have a positive influence on the adoption of protective measures at the individual level [70].

Furthermore, another previous study conducted in Poland, revealed that information can have the power to influence the level of trust that people have in the healthcare system and in healthcare professionals, suggesting that an increase of trust in hospitals, may be associated with a decrease of trust in physicians [71].

While focusing on studying people's response to non- pharmaceutical interventions, conspiracy theories and alternative treatments, a study conducted in Finland showed that the level of trust people have in the system implemented in order to provide information about the virus, has an essential role in the way people react to the official measures recommended. Hence, most participants in the study were between 40 and 60 years of age, and the study emphasized that people who were less willing to comply with the non-pharmaceutical interventions implemented by the government, tended to believe more in conspiracies and had low levels of trust in the sources which provided information about the virus [72].

Another study, which focused on examining the relationship between trust in the healthcare system and people's choice of seeking medical help when they experienced COVID – 19 symptoms, concluded that high levels of trust in the healthcare system can increase the probability of asking for medical help when people first notice COVID – 19 symptoms [73].

Taking into account the aspects mentioned above, we can infer that peoples' trust in doctors was affected during the pandemic. In this regard, in the context of misinformation, one of the reasons why people lost trust in doctors may be the fact that, besides using social media for communicating information, for networking or for interacting with patients, many medical

or dental practitioners used social media to express their professional opinions about the virus, opinions which were not validated and which later proven to be inaccurate [74]. In other words, health professionals may have contributed to the spread of misinformation, and such behavior can contribute to the decrease of trust in medical processes and in healthcare professionals [75]. Other researchers who focused on examining medical misinformation, found that most doctors (94.2%) stated that patients had medical misinformation, and the subjects about they had the most inaccurate information were represented by COVID – 19 vaccines, COVID – 19 origin, treatment or essential oils [76]. Furthermore, a previous study discovered that trust in doctors increased with age, and communication difficulties decreased, and that trust in doctors decreased while the level of education and communication difficulties increased [77].

Hence, while acknowledging that the pandemic influenced the trust in medical professionals, another aspect that was negatively influenced was the relationships between doctors and their patients. A study which focused on examining the doctor – patient interaction from the perspective of both groups of people, revealed differences in the respondents' opinions. Thus, most doctors stated that they still make eye contact (72%) and that they still show patients empathy, but only few patients declared that their doctors made eye contact (56,8%) or showed them empathy (43,2%) [78].

Methods and materials

Sampling and data collection procedures

The present study was conducted on Romanian healthcare professionals including doctors, nurses and medical students. The questionnaire was administered online, the data was collected through the help of Google forms, and was disseminated on groups of healthcare

professionals and students on platforms such as Facebook and WhatsApp, during the period April 2021– June 2021. The data we collected was firstly exported to Microsoft Excel, and then it was analyzed with IBM Statistical Package for the Social Sciences, version 20. At the beginning of the questionnaire, the respondents were informed about the purpose of the study, about the fact that they were allowed to withdraw at any time, and they were asked to give their consent for participating in the study. The average time needed to complete the questionnaire was 15 minutes, and the research received approval from The Council of the Faculty of Sociology and Communication, approval request Nr.378/30.03.2021.

The sample of our study comprises 536 respondents. Out of the 536 respondents, 460 (85.8%) were female and 76 (14.2%) were male. A total of 411 respondents live in the urban area (76.7%), while 125 (23.3%) live in the rural area. Most respondents (286, 53.4%) are between 18 and 35 years of age, 142 respondents (26.5%) are between 36 and 50 years of age, 102 respondents (19.0%) are between 51 and 65 years of age, and 6 of them (1.1) are over 65 years of age. When it comes to the professional degree of the respondents, most of them are students at a university nursing program (122, 22.8%), and medical students (120, 22.4%). However, a total of 102 respondents (19.0%) are senior specialists medical – doctors, and 70 (13.1%) are nurses who have a higher education diploma. When it comes to the respondents field of specialization, most of them (70.5%) operate in the field of general medicine, while others are family doctors (10.4%), pediatricians (3%), dentists or oncologists (1.9%), surgeons of doctors who are specialized in internal medicine (1.5%), or infectious disease doctors, radiologists or cardiologists (1.1%). Furthermore, most of the respondents (77.2%) stated that they did not work a unit with COVID – 19 patients while few of them (22.8%) stated that they

worked in such a unit at the time the research was conducted. Thus, all the characteristics of the sample are presented in Table 1.

Table 1. Sample characteristics (n = 536).

	Category	Count	Percentage
Gender	Female	460	88.8%
	Male	76	14.2%
Living environment	Urban	411	76.7%
	Rural	125	23.3%
Age	18-35 years old	286	53.4%
	36-50 years old	142	26.5%
	51 -65 years old	102	19.0%
	Over 65 years old	6	1.1%
Professional degree	Senior specialist medical - doctor	102	19.0%
	Specialist medical - doctor	46	8.6%
	Resident	28	5.2%
	Nurse with higher education diploma	70	13.1%
	Nurse with other studies than higher education	48	9.0%
	Medical student	120	22.4%
	Student at university nursing program	122	22.8%
Field of specialization	General medicine	378	70.5%
	Family doctor	56	10.4%
	Pediatrics	16	3%
	Stomatology	10	1.9%
	Oncology	10	1.9%
	Surgery	8	1.5%
	Internal medicine	8	1.5%
	Virology/ infectious disease doctor	6	1.1%
	Cardiology	6	1.1%
	Radiology	6	1.1%

	Other	32	6%
Works in a unit with COVID – 19 patients	Yes	122	22.8%
	No	414	77.2%

The research instrument

In order to conduct the research we used a quantitative method while having a questionnaire as an instrument. In this regard, we developed a questionnaire which comprises four sections: A. Influence of the pandemic on the professional activity of medical staff (items A1 to A4), B. Perception about the authorities' communication process (items B1 to B11), C. Perception about the communication of non- validated treatments (items C1 to C20), and D. Sociodemographic questions (items D1 – D9), such as: gender, age, living environment, professional degree, field of specialization. The sociodemographic questions were used in order to identify different or similar attitudes between specific groups. The questionnaire can be found in "S1.Appendix English version of the questionnaire", and in "S2. Appendix Romanian version of the questionnaire." Before disseminating the questionnaire, the instrument was tested on 30 doctors who work in the field of cardiology and general medicine. The respondents understood clearly the questions and did not report any issue in the process of answering them. Hence, the questionnaire comprises close ended and open ended questions (Items A1, A4,B3, B11, C19, C20, D2, D5, D6,) dihotomic questions as well as questions whose answers were measured on a 7 point Likert scale. For example, item A2 measured the extent to which the respondents considered that the pandemic influenced the way they carried out their

professional activity (1- “to an extremely little extent, 7 “to an extremely great extent”), or item B2 measure the respondents’ level of agreement with statements regarding the way authorities communicated during the pandemic (1 – “strongly disagree, 7-“ strongly agree”).

Data analysis

Data was analyzed with IBM Statistical Package for the Social Sciences, version 20. In order to analyze the data and identify differences and similarities between the attitudes of certain groups, t tests for independent samples were performed. The t test were performed among groups: male/female, working in unit with COVID – 19 patients/ not working in unit with COVID – 19 patients, urban/rural area, and professional degree: medical staff/students. Hence, in order to be able to analyze the results depending on professional degree, we computed the variable of professional degree which had the following values: senior specialist medical – doctor, specialist medical – doctor, resident, nurse with higher education diploma, nurse with other studies than higher education, medical student, student at university nursing program, in a new variable. Thus, doctors, nurses and residents, were integrated in a new group called “medical staff”, while medical students and students at university nursing programs were integrated in the group “students”. Moreover, for a better understanding of the way some variables correlate with each other, (for example: respondents satisfaction with the way authorities communicated during the pandemic and age, respondents’ opinion about the way misinformation about alternative treatments influenced doctors’ credibility and age), we also calculated the Pearson coefficient.

Results

1) To what extent information about alternative treatments affected the credibility of medical staff?

The results of our research revealed that respondents were of the opinion that information about alternative treatments for COVID -19 affected the credibility of healthcare professionals. Hence, most respondents (32.5%), stated that trust in healthcare professionals was affected to a an extremely great extent by the information about alternative treatments, many of them declared that credibility was affected to a very great extent (23.1%), and to a great extent (21.3%) (Table 1).

Table 1. The extent to which information about alternative treatments affected trust in physicians

		Frequency	Percent	Valid Percent	Cumulative Percent
Valid	to an extremely little extent	14	2.6	2.6	2.6
	to a very little extent	10	1.9	1.9	4.5
	to a little extent	42	7.8	7.8	12.3
	nor to a little, neither to a great extent	58	10.8	10.8	23.1
	to a great extent	114	21.3	21.3	44.4
	to a very great extent	124	23.1	23.1	67.5
	to an extremely great extent	174	32.5	32.5	100.0
	Total	536	100.0	100.0	

Furthermore, the Pearson correlation performed between the extent to which respondents believed that information about alternative treatments affected people's trust in doctors and the age of the respondents, revealed a weak, negative and statistically significant correlation between the two variables (r(534)= -.155, p=0.001) (Table 2). Hence, as the age of

the medical staff decreases, the extent to which they believe the credibility of doctors was affected increases. In other words, compared to older healthcare professionals, younger healthcare professionals tend to believe more that information about alternative treatments affected trust in doctors. One possible explanation for this result can be that younger people tend to be fonder of keeping up with trends and being up to date, and in this context, it is possible that they came into contact more frequently with information about certain alternative treatments for COVID – 19, this making them more aware about the way such treatments can undermine doctor's credibility.

Table 2. Pearson correlation between information about alternative treatments and age

		C14. The extent to which information about alternative treatments affected trust in physicians	D2. Age
C14. The extent to which information about alternative treatments affected trust in physicians	Pearson Correlation	1	-.155**
	Sig. (2-tailed)		.000
	N	536	536
D2. Age	Pearson Correlation	-.155**	1
	Sig. (2-tailed)	.000	
	N	536	536

**. Correlation is significant at the 0.01 level (2-tailed).

In order to observe if there any differences in the opinion of the respondents depending on certain variables including, age, gender, or living environment, we performed t tests for independent samples. The results of the significant t tests (Table 3), showed that students believed to a greater extent (M= 5.60, SD=1.49), that information about alternative treatments negatively affects the credibility of doctors, than the medical staff (M=5.33, SD=1.54). Also, respondents who declared they worked in a unit without COVID – 19 patients (M=5.53,

SD=1.49), were more of the opinion that information about alternative cures affected trust in health professionals, than respondents who worked in a unit with COVID – 19 patients (M=5.19, SD=1.61). One possible explanation would be that, doctors who interacted with COVID – 19 patients may have observed that when being put in the situation to receive medical care in the hospital, patients still had faith and trust in doctors. Moreover, another explanation is that respondents who did not come into contact with COVID – 19 patients were not that close with the situation and thus they might have had a more distorted perception about the situation than those professionals who interacted with COVID – 19 patients. Moreover, the results of the research also showed that female respondents (M=5.51, SD=1.48), believed more than male respondents (M=5.10, SD=1.70), that trust in healthcare professionals was affected by the information about alternative treatments.

Table 3. Significant t-test results: comparisons between variables

	t-test for Equality of Means										
	Group	N	Mean	S. D.	t	df	p	Mean Difference	Std. Error Difference	CI4 Lower	CI4 Upper
Information about alternative treatments _ Professional degree[1]	Medical staff	294	5.33	1.54	-2.04	534	.04	-.27	.13	-.52	-.01
	Student	242	5.60	1.49							
Information about alternative treatments _working unit	Unit with COVID -19 patients	122	5.19	1.61	-2.13	534	.03	-.33	.15	-.64	-.02
	Unit without COVID 19 patients	414	5.53	1.49							
Information about alternative treatments _gender	Male	76	5.10	1.70	-2.16	534	.03	-.40	.18	-.77	-.03
	Female	460	5.51	1.48							

[1]Index variable from the professional degrees of respondents. Student: medical student and student at university nursing program, Medical Staff: Senior specialist medical – doctor, Specialist medical – doctor, Resident, Nurse with higher education diploma, Nurse with other studies than higher education

2) What is the knowledge of medical staff about the type of drugs that had positive effects on treating the disease and about alternative treatments?

Considering the type of drugs which were known to have positive effects on treating the virus, the research revealed that type of drug about which the respondents have heard it had positive effects against the virus was Dexamethasone (46.6%), closely followed by Remdesivir (40.5%) and Azithromicin (38.4%). However, some of the respondents also mentioned Chloroquine, Hydroxychloroquine (23.1%), Ibuprofen (19.8%), Tocilizumab (15.9%), and Favipiravir (13.8%) as drugs known to have positive effects when dealing with COVID – 19 (Table 4). Hence, the research showed that the medical staff had knowledge about the type of drugs tested or used against the virus, which were taught to be efficient in treating the disease.

Table 4. Drugs known to have positive effects in treating the virus: the perception of medical staff

	**Frequency**	**Valid percent**
Amoxicillin	36	6.7%
Azithromicin	206	38.4%
Chloroquine, Hydroxychloroquine	124	23.1%
Dexamethasone	250	46.6%
Doxycycline	32	6.0%
Favipiravir	74	13.8%
Ibuprofen	106	19.8%
Lopinavir/Ritonavir	56	10.4%
Oseltamivir, Peramivir or Zanamivir	32	6.0%
Remdesivir	217	40.5%
Tocilizumab	85	15.9%

Umifenovir	17	3.2%

In the context of respondents' perception about alternative methods of preventing and treating the virus, the findings show that, most of them stated that they heard about the fact that alcohol consumption can prevent the infection with the virus (24.3%), that drinking warm water every 15 minutes may help eliminate the virus (21.3%), but also that pointing the hot air of the hairdryer to the nostrils leads to the elimination of the virus (16.8%) (Table 5).

Table 5. Medical staff's knowledge about alternative methods of preventing and treating the virus

		Frequency	Percent	Valid Percent	Cumulative Percent
Valid	drinking alcohol helps you eliminate the virus	79	14.7	14.7	14.7
	drinking alcohol prevents the infection with the virus	130	24.3	24.3	39.0
	rinsing the nostrils with disinfectant eliminates the virus	81	15.1	15.1	54.1
	drinking hot water every 15 minutes eliminates the virus	114	21.3	21.3	75.4
	pointing hot air to the nostrils leads to the elimination of the virus	90	16.8	16.8	92.2
	other	42	7.8	7.8	100.0
	Total	536	100.0	100.0	

3) How satisfied is the medical staff with the way medical and non-medical information was communicated during the pandemic?

The findings of the study revealed that respondents were mostly dissatisfied with the way medical and non – medical information was communicated during the pandemic. Hence, the

sum of the responses with negative valences of the study participants (extremely dissatisfied, very dissatisfied and dissatisfied), showed that 238 of them, (44.4%) were dissatisfied with the process of sending medical and non- medical information, while the sum of the positive responses (satisfied, very satisfied, extremely satisfied) showed that 162 of them (30.2%), were satisfied with the communication process (Table 6). In other words, the study highlighted that respondents registered mostly low level of satisfaction with the way information was sent during the pandemic.

Table 6. The level of satisfaction with the way information about drugs used to treat the virus were communicated at national level

		Frequency	Percent	Valid Percent	Cumulative Percent
Valid	extremely dissatisfied	52	9.7	9.7	9.7
	very dissatisfied	76	14.2	14.2	23.9
	dissatisfied	110	20.5	20.5	44.4
	Nor dissatisfied, neither satisfied	136	25.4	25.4	69.8
	satisfied	108	20.1	20.1	89.9
	very satisfied	30	5.6	5.6	95.5
	Extremely satisfied	24	4.5	4.5	100.0
	Total	536	100.0	100.0	

Furthermore, in the context of the medical staff's satisfaction with the way information about drugs used to treat the virus was communicated at national level, the research showed that as age of the respondents decreases, the level of satisfaction increases ($r(534)= -.091$, $p=0.035$) (Table 7). Thus, according to this result, it can be inferred that younger people were more satisfied than older people, with how information about drugs used to treat the virus was communicated.

Table 7. Pearson Correlation: satisfaction with the way information about drugs used to treat the virus was communicated and age

		B10. Satisfaction with the way information about drugs used to treat the virus was communicated	D2. Age
B10. Satisfaction with the way information about drugs used to treat the virus was communicated	Pearson Correlation	1	-.091*
	Sig. (2-tailed)		.035
	N	536	536
D2. Age	Pearson Correlation	-.091*	1
	Sig. (2-tailed)	.035	
	N	536	536

*. Correlation is significant at the 0.05 level (2-tailed).

Moreover, when asked to evaluate the efficiency of the communication strategies adopted by authorities in order to send information about the virus, most respondents stated that the strategies were effective. Thus, the sum of the responses with negative valences shows that 144 of them (26, 9%) described the communication strategies as inefficient, while 266 of them (49, 6%) described them as efficient (Table 8). One interesting result of the analysis, was that, when trying to examine if the responses of the study participants about the efficiency of communication strategies differ depending on certain variables such as working unit, gender, working unit, living environment, the analysis found no differences between the responses of males and females, of people working in units without COVID – 19 patients and people not working in units with COVID – 19 patients, or in people from the rural and urban area.

Table 8. Perception about the efficiency of communication strategies adopted by authorities

		Frequency	Percent	Valid Percent	Cumulative Percent
Valid	Extremely inefficient	22	4.1	4.1	4.1
	very inefficient	38	7.1	7.1	11.2
	inefficient	84	15.7	15.7	26.9
	nor efficient, neither inefficient	126	23.5	23.5	50.4
	efficient	134	25.0	25.0	75.4
	very efficient	80	14.9	14.9	90.3
	extremely efficient	52	9.7	9.7	100.0
	Total	536	100.0	100.0	

In the context of the information about drugs tested and used in the treatment against COVID – 19, the results showed that students believe to a greater extent that such information was communicated in a coherent manner (M=4.05, SD=1.63), than the medical staff (M=3.79, SD=1.53) ($t(534)= -2.05$, $p<0.05$) (Table 9.). Hence, one possible explanation for this result would be that, due the experience and knowledge of the medical staff, people who were already working in the healthcare system, such people have greater expectations from authorities when it comes to sending medical information, than medical students.

Table 9. Significant t test for information about drugs used to treat the virus and professional degree

	t-test for Equality of Means										
	Group	N	Mean	S. D.	t	df	p	Mean Difference	Std. Error Difference	CI4	
										Lower	Upper
Information about drugs tested and used to treat the disease[1] _ Professional degree[2]	Medical staff	294	3.79	1.53	-2.05	534	.03	-.28	.13	-.55	-.01
	Student	242	4.05	1.63							

[1] The extent to which respondents believe that information about drugs tested and used to treat the virus was communicated in a coherent manner

[2] Index variable from the professional degrees of respondents. Student: medical student and student at university nursing program, Medical Staff: Senior specialist medical – doctor, Specialist medical – doctor, Resident, Nurse with higher education diploma, Nurse with other studies than higher education

(4) What is the perception of medical staff about the role of social media in spreading misinformation about the virus?

The results of the research revealed that respondents were inclined to believe more that social media was a proper environment for spreading fake medical information during the pandemic. By analyzing the information from Table 10, it can be observed that the sum of the responses with negative valences (4.5%) (to an extremely little extent, to a very little extent and to a little extent) is much lower than the sum of the responses with positive valences (89.9%) (to an extremely great extent, to a very great extent, to a great extent). Hence, most participants of the study believe that social media platforms favored the transmission of fake medical news during the pandemic. Furthermore, when trying to find differences in the responses of the participants depending on age, gender, living environment, professional degree or working unit (with COVID – 19 patients or without COVID – 19 patients), we observed that their responses did not differ depending on such variables. Thus, it can be inferred that, regardless of age, gender, living environment, professional degree or working unit, respondents' perception was that social media had a role in spreading fake medical information.

Table 10. Perception about the extent to which social media contributed to the spread of medical fake news

		Frequency	Percent	Valid Percent	Cumulative Percent
Valid	to an extremely little extent	2	.4	.4	.4
	to a very little extent	10	1.9	1.9	2.2
	to a little extent	12	2.2	2.2	4.5

	nor to a little, neither to a great extent	30	5.6	5.6	10.1
	to a great extent	62	11.6	11.6	21.6
	to a very great extent	88	16.4	16.4	38.1
	to an extremely great extent	332	61.9	61.9	100.0
	Total	536	100.0	100.0	

However, even though respondents were of the opinion that social media was an environment in which was sent fake medical information, some of them still believe that social media platforms are appropriate for sending official information about the virus. Thus, considering the results from Table 11, the sum of responses with positive valences (40.3%) is almost equal to the sum of responses with negative valences (45.1%) meaning that the opinions of the study participants were divided when it comes to sending official information about the virus on social media.

Table 11. Perception about the extent to which social media represents an appropriate environment for sharing official COVID – 19 information

		Frequency	Percent	Valid Percent	Cumulative Percent
Valid	to an extremely little extent	86	16.0	16.0	16.0
	to a very little extent	78	14.6	14.6	30.6
	to a little extent	52	9.7	9.7	40.3
	nor to a little, neither to a great extent	78	14.6	14.6	54.9
	to a great extent	72	13.4	13.4	68.3
	to a very great extent	74	13.8	13.8	82.1
	to an extremely great extent	96	17.9	17.9	100.0
	Total	536	100.0	100.0	

A factor which showed a weak but statistically significant influence on respondents' opinion about sending COVID – 19 official information on social media was age. Hence, the results of

the Pearson correlation (r (534) = -.175, p=0.000), showed that as age decreases, the extent to which respondents believed that social media is an environment in which official information about the virus should be communicated decreases (Table 12). In other words, younger respondents believed to a greater extent than older respondents that official information should also be communicated on social media. One possible explanation for this results would be that young people gather most of their information from online sources, and they also engage more with social media platforms, and thus it is possible that they would also like to see official and important information on such platforms.

Table 12. Person correlation between the extent to which social media represents an appropriate environment for sharing official COVID – 19 info and age

		C1. The extent to which social media represents an appropriate environment for sharing official COVID – 19 info	D2. Age
C1. The extent to which social media represents an appropriate environment for sharing official COVID – 19 info	Pearson Correlation	1	-.175**
	Sig. (2-tailed)		.000
	N	536	536
D2. Age	Pearson Correlation	-.175**	1
	Sig. (2-tailed)	.000	
	N	536	536

**. Correlation is significant at the 0.01 level (2-tailed).

Furthermore, when dividing the study participants in medical staff (doctors, nurses) and students (medical students or students at the university nursing programs), we found that students (M=4.31, SD=2.11) believed to a greater extent than the medical staff (M= 3.88, SD=2.07) that official information about the virus should also be sent on social media (t (534) = -

2.36, $p < 0.05$) (Table 13). Next, when dividing the sample by living environment, participants living in the urban area (M=4.19, SD=2.10) were inclined more than those living in the rural area (M=3.72, SD=2.05), to believe that official information could also be sent on social media (t (534) = 2.23, $p < 0.05$) (Table 13).

Table 13. Significant t tests for sharing official information on social media professional degree and living environment

	t-test for Equality of Means										
	Group	N	Mean	S. D.	t	df	p	Mean Difference	Std. Error Difference	CI4 Lower	CI4 Upper
Official information on social media _ Professional degree[1]	Medical staff	294	3.88	2.07	-2.36	534	.01	-.42	.18	-.78	-.07
	Student	242	4.31	2.11							
Official information on social media _living environment	Urban area	411	4.19	2.10	2.23	534	.02	.47	.21	.05	.89
	Rural area	125	3.71	2.05							

[1]Index variable from the professional degrees of respondents. Student: medical student and student at university nursing program, Medical Staff: Senior specialist medical – doctor, Specialist medical – doctor, Resident, Nurse with higher education diploma, Nurse with other studies than higher education

(5) What aspects of the professional activity of the medical staff were affected most by the COVID – 19 pandemic?

The findings of our research showed that most respondents stated that the patient – doctor relationship was most affected by the pandemic (38.4%). However, a smaller percent of respondents declared that the working schedule was the most affected (26.9%), or the collaboration with their peers (23.9%) (Table 14).

Table 14. The aspect of professional life which was most influenced by the pandemic

	Frequency	Percent	Valid Percent	Cumulative Percent

Valid	patient – doctor relationship	206	38.4	38.4	38.4
	work schedule	144	26.9	26.9	65.3
	collaboration with peers	128	23.9	23.9	89.2
	other	58	10.8	10.8	100.0
	Total	536	100.0	100.0	

Furthermore, taking into account the group of medical staff (doctors, nurses) and the group of students (medical students and students at university nursing program), the results revealed that the most respondents who stated that the patient- doctor relationship was affected most by the pandemic was the group of medical staff (144 compared to 62) (Table 15). One possible explanation for this result is that, by being in constant contact with their patients, doctors and nurses were more inclined to perceive that the relation with their patients has deteriorated during the pandemic.

[illegible]able 15. Main aspect of professional life influenced by the pandemic * professional degree - Cross tabulation

		Professional degree[1]		Total
		Medical staff	Student	
A3. Main aspect of professional life influenced by the pandemic	patient – doctor relationship	144	62	206
	work schedule	70	74	144
	collaboration with peers	62	66	128
	other	18	40	58
Total		294	242	536

[1]Index variable from the professional degrees of respondents. Student: medical student and student at university nursing program, Medical Staff: Senior specialist medical – doctor, Specialist medical – doctor, Resident, Nurse with higher education diploma, Nurse with other studies than higher education

Discussion

During the COVID – 19 pandemic, one of the major issues people had to face, was the spread of misinformation about the virus, its origins and its treatment. In this regard, we analyzed the perception of medical staff (including doctors, nurses, medical students and students in the university nursing program) about the way medical and non – medical information was communicated during the pandemic. In the context of the so called infodemic [11], and the effects of misinformation on people's trust in doctors, most participants of our study declared that the information about alternative treatments for the virus affected the credibility of health professionals. Hence, from this point of view, our study is in line with previous studies which highlighted the fact that lately, trust in physician decreased [67], and which suggested that social media managed to determine people to trust the personal opinions of other people rather than the opinion of the professionals [61]. Furthermore, since other researchers pointed out that many medical practitioners used social media to express professional opinions that were later found inaccurate [74], and thus they may have contributed to the spread of misinformation [75], we argue that the credibility of physicians might have also been affected by this type of behavior.

An interesting result of our research showed that as the age of medical staff decreases, the extent to which they believe that information about alternative treatments affects doctors' credibility increases. Hence, younger healthcare professionals believed to a greater extent than older healthcare professionals, that information about alternative treatments affected negatively people's trust in doctors. This results might have as possible explanation, the fact that younger people tend to spend more time on social media platforms, and they may have

interacted more than older professionals, with misinformation about the virus, this making them more able to be aware of the negative effects of fake news. Moreover, the type of unit in which the respondents worked, was a factor which influenced the opinion of the respondents, our findings showing that, the medical staff who did not work in unit with COVID -19 patients, believed to a greater extent than those who worked in such units, that information about alternative treatments negatively influenced doctors' credibility. Given this result we argue that is it possible for those professionals who did not interact with COVID -19 patients, and who thus were more distant from the situation, to have a more distorted image regarding the way people's levels of trust in them changed in the context of the pandemic.

Considering the role of social media in spreading misinformation, our study is in line with previous studies which support the idea that such channels favored the communication of fake news during the pandemic [49, 50, 51]. In this regard, regardless of age, professional degree or living environment, most healthcare professionals who participated in our study were of the opinion that social media contributed to the spread of misinformation. However, our study also showed that when it comes to communicating official information on social media, younger respondents (students) believed to a greater extent than older respondents (doctors, nurses), that such channels should be used to send official information about the virus. Taking into account these results, the fact that healthcare professionals acknowledge that social media favors the spread of misinformation, and that many of them still believe they should be used in order to communicate official information, shows that at personal level, professionals were not affected that much by misinformation, them being able to differentiate more easily between real and fake news. In other words, we argue that while people in general were negatively

influenced by the fake news they read on social media, as it was shown in previous studies which highlighted that people trusted the information on social networks, they shared un-validated information and had trouble with differentiating real from fake news [57, 79] or that exposure to health misinformation may influence people's intention to engage in certain behaviors [80], healthcare professionals may be less influenced by fake news, due to their knowledge.

Considering the knowledge of medical staff about the type of drugs that had positive effects on treating the virus, the findings of the research showed that the respondents had opinions which were in line with the results found in other studies. Hence, according to the research, most respondents stated that the drug which was known to have positive effects against the virus was Dexamethasone (46.6%), it being followed by Remdesivir (40.5%). Thus, positive effects of Dexamethasone were also highlighted by studies [31, 32], while study [35] showed positive effects of Remdesivir.

In the context of medical staff's knowledge about alternative treatments, most respondents declared they had heard about the fact that alcohol can prevent the infection, that warm water drunk every 15 minutes, and the hot air from the hairdryer pointed to the nostrils can help eliminate the virus. From this point of view, our study is in line with a previous study [53], which also described these methods.

When it comes to the respondents' level of satisfaction about the way medical and non – medical information was communicated during the pandemic, generally, the research revealed that most respondents were dissatisfied with the communication process. In the case of communication strategies adopted by authorities, the results showed that most respondents

were satisfied with them. However, in the context of sending information about the drugs used to treat the disease, the research showed that younger healthcare professionals were more satisfied with the communication process than older healthcare professionals. This results might be due to the fact that physicians with more experience have higher expectations from authorities than students.

Another area on which we focused our research was the professional activity of the medical staff during the pandemic. In this regard, our findings revealed that, according to the respondents of our study, the aspect that was mostly affected by the pandemic was the doctor-patient relationship. Hence, our research is in line with other studies [78], which showed that the pandemic affected the way doctors interacted with their patients.

Furthermore, on the basis of the results of our study we argue that not only the process of vaccination created ethical issues, but also the process of communication [81]. Thus, these ethical issues were perceived by the medical staff and they would require a further examination in order to be able to create communication guides which can be regarded as essential instruments not only for the research process of the medical staff and healthcare professionals with management positions, but also for their current medical activity [82,83].

Conclusions

During the pandemic, healthcare professionals did not have to deal only with challenges regarding their health and the health of their patients, but also with the problems created by the spread of medical misinformation. In this regard, besides fighting the pandemic, physician also had to fight the so called infodemic. Fake news spread on social media about various alternative treatments for the virus and the opinions of certain professionals about treatment

methods which later proven to be inaccurate negatively influenced the credibility of doctors. Hence, according to the results of our research, generally, the medical staff (doctors, nurses, medical students, students at university nursing program), believed that information about alternative treatments affected people's trust in doctors, but younger healthcare professionals and those working in units without COVID - 19 patients believed to a greater extent than older healthcare professionals and people working in units with COVID – 19 patients that fake news about treatments for the virus affected the credibility of doctors.

Furthermore, regardless of age, age, gender, living environment, professional degree or working unit, the medical staff acknowledged the role of social media in spreading fake news, but when it comes to using social media in order to communicate official information, younger healthcare professionals were more inclined to believe that such networks were appropriate for the communication of official information. This results can suggest that while professionals were aware of the role of social media in spreading medical misinformation and in affecting trust in doctors, due to their knowledge, at personal level they were less affected by that type of information, many of them believing that social media should also be used for sending official information.

In the context of the drugs used to treat the virus, the results pointed out that the medical staff had knowledge about the drugs known to have positive effects in treating the virus, their perception being in line with previous studies which focused on this matter. Moreover, the medical staff was aware of the alternative treatments which were promoted on social media, the method of drinking alcohol in order to prevent the infection being the method that most of the respondents have heard about.

When it comes to the influence of the pandemic on the professional activity of the medical staff, the respondents declared that the aspect which was most affected was the doctor – patient relationship. In this regard, we argue that, by influencing peoples' trust in doctors, the medical fake news spread during the pandemic, implicitly had a role in deteriorating the relation between doctors and their patients.

Therefore, the healthcare professionals were generally dissatisfied with the way medical and non – medical information was communicated during the pandemic, but younger professionals were satisfied than older professionals. Overall, the medical staff believed that fake news managed to undermine doctors' credibility that social media platforms favor the spread of such news, and they had knowledge about the drugs which were known to have positive effects on the virus and about the alternative treatments.

Taking into account the results of the research, the paper has some theoretical and practical implications. From a theoretical point of view, the paper contributes to the literature on the matter of fake news and its influence on the trust of healthcare professionals, a strength of the paper being the fact that it analyzed the opinions of medical staff (doctors, nurses, medical students and students at university nursing program). From a practical point of view, the paper brings awareness to the phenomenon of fake news regarding medical treatments and the negative influence it has on doctors' credibility. Another practical implication refers to the fact that the paper brings attention to the issue of using social media as a mean to communicate official information, many healthcare professionals, especially the younger ones, stating that such networks could be appropriate for sharing official information. Furthermore, by highlighting that the most affected aspect of the professional activity of doctors was the

relationship with their patients, the study also shows that actions need to be taken in order to restore people’s trust in doctors and improve the process of communication between them. Hence, on the basis of the findings and implications of the study, we further discuss limitations and future research directions.

Limitations and future research directions

While our study proved relevant information regarding the perception of healthcare professionals about the way medical and non – medical information was communicated in time of the pandemic, it also has some limitations.

One limitation is represented by the fact that the perception of healthcare professionals was studied only by using quantitative methods. In this regard, a future research should focus on obtaining information from doctors while using qualitative methods too. Next, the study was conducted only on Romanian healthcare professionals, and thus, a future research should take into consideration a comparison between the opinions of professionals from different countries. Another limitation is represented by the fact that we only asked respondents to state the aspect which was most influenced by the pandemic, but we did not asked them to offer detail about other type of challenges encountered. Thus, a future research should focus on analyzing the extent to which aspects of the professional activity of doctors were affected, and on analyzing more deeply the challenges they had to face during the pandemic.

Furthermore, since our research revealed that many respondents believed that social media platforms could be appropriate for sharing official information, we draw attention to a problem that can arise in this context. Since people know that such platforms favor the spread

of fake news, if we encourage the use of social media in order to communicate official information, don’t we risk to discredit that information as it is possible for people to consider that such information is fake too? We believe that this issue should be taken into account and studied in a future research.

Author Contributions

Conceptualization: Claudiu Coman, Maria Cristina Bularca

Data curation: Claudiu Coman, Liliana Rogozea, Angela Repanovici, Maria Cristina Bularca

Formal analysis: Maria Cristina Bularca, Claudiu Coman

Investigation: Claudiu Coman, Maria Cristina Bularca, Angela Repanovici, Liliana Rogozea

Methodology: Maria Cristina Bularca, Claudiu Coman

Project administration: Claudiu Coman, Liliana Rogozea, Angela Repanovici

Resources: Maria Cristina Bularca, Liliana Rogozea, Angela Repanovici

Supervision: Claudiu Coman, Liliana Rogozea, Angela Repanovici

Writing – original draft: Maria Cristina Bularca, Claudiu Coman

Writing – review & editing: Maria Cristina Bularca, Claudiu Coman, Liliana Rogozea, Angela Repanovici

References

1. Sanders JM, Monogue ML, Jodlowski TZ, Cutrell J. B.Pharmacologic Treatments for Coronavirus Disease 2019 (COVID-19): A Review. *JAMA.* 2020; 323(18):1824–1836. doi:10.1001/jama.2020.6019

2. Shereen MA, Khan S, Kazmi A, Bashir N, Siddique R. COVID-19 infection: Origin, transmission, and characteristics of human coronaviruses. *J Adv Res*. 2020; 24:91-98 doi: 10.1016/j.jare.2020.03.005

3. Kristina SA, Herliana N, Hanifah S. The perception of role and responsibilities during covid-19 pandemic: A survey from Indonesian pharmacists. *Int J Pharm Res* , 2020; *12*(2). doi: 10.31838/ijpr/2020.SP2.369

4. World Health Organization (WHO). Timeline of WHO’s response to COVID-19. [Internet]. World Health Organization. 2020 June 29 [cited 2020 Nov 27] Available from: https://www.who.int/news/item/29-06-2020-covidtimeline

5. European Centre for Disease Prevention and Control. COVID-19 situation update worldwide, as of 27 November 2020 [Internet]. European Centre for Disease Prevention and Control. 2020 June 29 [cited 2021 Nov 25] Available from: https://www.ecdc.europa.eu/en/geographical-distribution-2019-ncov-cases.

6. British Broadcasting Corporation. COVID vaccine: First 'milestone' vaccine offers 90% protection. [Internet]. 2020 Nov 9 [cited 2020 Nov 27] Available from: https://www.bbc.com/news/health-54873105.

7. Kommenda N, Jones FH. COVID vaccine tracker: when will a coronavirus vaccine be ready? [Internet]. The Guardian. 2020 Nov 10 [cited 2020 Nov 27] Available from:

https://www.theguardian.com/world/ng-interactive/2020/nov/10/covid-vaccine-tracker-when-will-a-coronavirus-vaccine-be-ready

8. Ahsan W, Javed S, Al Bratty M, Alhazmi HA, Najmi A. Treatment of SARS-CoV-2: How far have we reached? *Drug Discov Ther*. 2020; 14(2):67-72. doi: 10.5582/ddt.2020.03008

9. Kupferschmidt K, Cohen J. Race to find COVID-19 treatments accelerates. *Science*. 2020; 367(6485): 1412-1413. doi: 10.1126/science.367.6485.1412

10. World Health Organization (WHO). Solidarity clinical trial for COVID-19 treatments [Internet]. World Health Organization 2019. [cited 2020 Nov 27] Available from: https://www.who.int/emergencies/diseases/novel-coronavirus-2019/global-research-on-novel-coronavirus-2019-ncov/solidarity-clinical-trial-for-covid-19-treatments

11. Zarocostas J. How to fight an infodemic. *The Lancet*. 2020; *395*(10225):676. doi: 10.1016/S0140-6736(20)30461-X

12. Tuccori M, Convertino I, Ferraro S, Cappello E, Valdiserra G, Focosi D et al. The Impact of the COVID-19 "Infodemic" on Drug-Utilization Behaviors: Implications for Pharmacovigilance. *Drug Saf*. 2020;43:699–709 doi:10.1007/s40264-020-00965-w

13. Rosa SGV, Santos WC. Clinical trials on drug repositioning for COVID-19 treatment. *Rev Panam Salud Publica*. 2020; 44: e40 doi: 10.26633/RPSP.2020.40

14. Sahraei Z, Shabani M, Shokouhi S, Saffaei A. Aminoquinolines against coronavirus disease 2019 (COVID-19): chloroquine or hydroxychloroquine. *Int J Antimicrob Agents*. 2020; *105945*(10.1016) doi:10.1016/j.ijantimicag.2020.105945

15. White NJ, Watson JA, Hoglund RM, Chan XHS, Cheah PY, Tarning J. COVID-19 prevention and treatment: A critical analysis of chloroquine and hydroxychloroquine clinical pharmacology. PLoS Med. 2020; 17(9): e1003252. doi:10.1371/journal.pmed.1003252

16. Liu J, Cao R, Xu M, Wang X, Zhang H, Hu H. et al. Hydroxychloroquine, a less toxic derivative of chloroquine, is effective in inhibiting SARS-CoV-2 infection in vitro. Cell Discov. 2020; 6(16) doi:10.1038/s41421-020-0156-0 .

17. Wang M, Cao R, Zhang L, Yang X, Liu J, Xu M. et.al. Remdesivir and chloroquine effectively inhibit the recently emerged novel coronavirus (2019-nCoV) in vitro. *Cell Res*. 2020; 30:269–271. doi:10.1038/s41422-020-0282-0

18. Davidescu EI, Odajiu I, Bunea T, Sandu G, Stratan L, Aramă V. et.al. Treatment with hydroxychloroquine in patients with covid-19. Experience of a neurology department. *Farmacia*. 2020; *68*(4): 597-605. doi:10.31925/farmacia.2020.4.3

19. Gautret P, Lagier JC, Parola P, Meddeb L, Mailhe M, Doudier B. et.al. Hydroxychloroquine and azithromycin as a treatment of COVID-19: results of an open-label non-randomized clinical trial. *Int. J. Antimicrob. Agents*. 2020; 105949. doi:10.1016/j.ijantimicag.2020.105949.

20. Thomson K, Nachlis H. Emergency Use Authorizations During the COVID-19 Pandemic: Lessons From Hydroxychloroquine for Vaccine Authorization and Approval. *JAMA.* 2020; 324(13):1282–1283. doi:10.1001/jama.2020.16253.

21. U.S Food & Drug Administration. FDA cautions against use of hydroxychloroquine or chloroquine for COVID-19 outside of the hospital setting or a clinical trial due to risk of heart rhythm problems. [Internet]. Food and Drug Administration. 2020 Jul 1 [cited 2020 Nov 27]

Available from: https://www.fda.gov/drugs/drug-safety-and-availability/fda-cautions-against-use-hydroxychloroquine-or-chloroquine-covid-19-outside-hospital-setting-or .

22. Recovery. No clinical benefit from use of hydroxychloroquine in hospitalized patients with COVID-19. [Internet]. Recovery 2020 June 5 [cited 2020 Nov 27] Available from: https://www.recoverytrial.net/news/statement-from-the-chief-investigators-of-the-randomised-evaluation-of-covid-19-therapy-recovery-trial-on-hydroxychloroquine-5-june-2020-no-clinical-benefit-from-use-of-hydroxychloroquine-in-hospitalised-patients-with-covid-19 .

23. Naveed M, Uddin S, Abdullah KS, Ishaq SE, Ahmad T. Various Evidence-Based Hypothetical and Experimental Treatment Approaches and Their Effectiveness against COVID-19 Worldwide: A Comprehensive Literature Review. *EJMO* 2020; 4(4):265–285. doi: 10.14744/ejmo.2020.52538

24. Chu C M, Cheng VCC, Hung IFN, Wong MML, Chan KH, Chan KS, et.al. Role of lopinavir/ritonavir in the treatment of SARS: initial virological and clinical findings. *Thorax*. 2004; 59(3):252-256. doi:10.1136/thorax.2003.012658

25. Cao B, Wang Y, Wen D, Liu W, Wang J, Fan G, et al. A trial of lopinavir-ritonavir in adults hospitalized with severe covid-19. *N. Engl . J. Med.* 2020; 382(19): 1787-1799. doi:10.1056/NEJMoa2001282

26. Vosu J, Britton P, Howard-Jones A, Isaacs D, Kesson A, Khatami A, et al. Is the risk of ibuprofen or other non-steroidal anti-inflammatory drugs increased in COVID-19?. *J Paediatr Child Health*. 2020; *56*(10): 1645-1646. doi:10.1111/jpc.15159

27. Fang L, Karakiulakis G, Roth M. Are patients with hypertension and diabetes mellitus at increased risk for COVID-19 infection? *Lancet Respir Med*. 2020; 8(4):e21.8 doi:10.1016/S2213-2600(20)30116-8

28. Science alert. Updated: WHO Now Doesn't Recommend Avoiding Ibuprofen For COVID-19 Symptoms [Internet]. Science alert 2020 Mar 17 [cited 2020 Nov 27]. Available from: https://www.sciencealert.com/who-recommends-to-avoid-taking-ibuprofen-for-covid-19-symptoms

29. Esba LCA, Alqahtani RA, Thomas A, Shamas N, Alswaidan L, Mardawi G. Ibuprofen and NSAID Use in COVID-19 Infected Patients Is Not Associated with Worse Outcomes: A Prospective Cohort Study. *Infect Dis Ther*. 2020; 1-16. doi:10.1007/s40121-020-00363-w

30. Rinott E, Kozer E, Shapira Y, Bar-Haim A, Youngster I. Ibuprofen use and clinical outcomes in COVID-19 patients. *Clin Microbiol Infect*. 2020; 26(9):1259.e5-1259.e7. doi:10.1016/j.cmi.2020.06.003

31. Roberts M. Coronavirus: Dexamethasone proves first life-saving drug. [Internet]. British Broadcasting Corporation 2020 June 16 [cited 2020 Nov 27] Available from: https://www.bbc.com/news/health-53061281.

32. Horby P, Lim WS, Emberson JR, Mafham M, Bell JL, Linsell L, et al.. Dexamethasone in hospitalized patients with Covid-19-preliminary report. *N. Engl. J. Med*. 2020. doi:10.1056/nejmoa2021436

33. Lu CC, Chen MY, Lee WS, Chang YL. Potential therapeutic agents against COVID-19: What we know so far. *J Chin Med Assoc*. 2020; 83(6):534-536. doi: 10.1097/JCMA.0000000000000318

34. Wang Y, Zhang D, Du G, Du R, Zhao J, Jin Y, et al. Remdesivir in adults with severe COVID-19: a randomised, double-blind, placebo-controlled, multicentre trial. *The Lancet*. 2020; 395(10236):1569-1578. doi:10.1016/S0140-6736(20)31022-9

35. Beigel JH, Tomashek KM, Dodd LE, Mehta AK, Zingman BS, Kalil AC. et al. Remdesivir for the treatment of Covid-19. *N. Engl. J. Med*. 2020. https://www.nejm.org/doi/10.1056/NEJMoa2007764

36. U.S Food and Drug Administration. FDA Approves First Treatment for COVID-19 [Internet]. Food and Drug Administration 2020 Oct 22 [cited 2020 Nov 27] Available from: https://www.fda.gov/news-events/press-announcements/fda-approves-first-treatment-covid-19.

37. U.S Food and Drug Administration. Coronavirus (COVID-19) Update: November 20, 2020 [Internet]. Food and Drug Administration 2020 Nov 20 [cited 2020 Nov 27] Available from: https://www.fda.gov/news-events/press-announcements/coronavirus-covid-19-update-november-20-2020.

38. Glasdam S, Stjernswärd S. Information about the COVID-19 pandemic–a thematic analysis of different ways of perceiving true and untrue information. *SSHOP.* 2020; (2)1: 100090. https://doi.org/10.1016/j.ssaho.2020.100090

39. Caleb TC, Hayes RA. Social Media: Defining, Developing, and Divining. *Atl J Commun.*2015; 23:1: 46-65. https://doi.org/10.1080/15456870.2015.972282

40. Boyd DM, Ellison NB.Social network sites: Definition, history, and scholarship. *J Comput Mediat Commun.* 2007; *13*(1): 210-230. https://doi.org/10.1111/j.1083-6101.2007.00393.x

41. Reuter C, Stieglitz S, Imran M.Social media in conflicts and crisesBehav. *Inf. Technol*. 2020; *39*(3): 241-251. https://doi.org/10.1080/0144929X.2019.1629025

42. Vasconcellos-Silva PR, Castiel LD. COVID-19, fake news, and the sleep of communicative reason producing monsters: the narrative of risks and the risks of narratives. *Cad Saude Publica*. 2020); *36*(7): e00101920. https://doi.org/10.1590/0102-311x00101920

43. Pulido CM, Ruiz-Eugeni L, Redondo-Sama G, Villarejo-Carballido B. A New Application of Social Impact in Social Media for Overcoming Fake News in Health. *Int. J. Environ. Res. Public Health*. 2020; *17*(7):2430. **https://doi.org/10.3390/ijerph17072430**

44. Al-Dmour H, Salman A, Abuhashesh M, Al-Dmour R.Influence of social media platforms on public health protection against the COVID-19 pandemic via the mediating effects of public health awareness and behavioral changes: integrated model. *J. Medical Internet Res*. 2020; *22*(8):e19996.

45. Wong JEL, Leo YS, Tan CC. COVID-19 in Singapore—Current Experience: Critical Global Issues That Require Attention and Action. *JAMA.* 2020; 323(13):1243–1244. doi:10.1001/jama.2020.2467

46. Lazer DM, Baum MA, Benkler Y, Berinsky AJ, Greenhill KM, Menczer F, et al. The science of fake news. *Science*. 2018; *359*(6380):1094-1096. DOI: 10.1126/science.aao2998

47. Moscadelli A, Albora G, Biamonte MA, Giorgetti D, Innocenzio M, Paoli S, et al. Fake News and Covid-19 in Italy: Results of a Quantitative Observational Study. *Int. J. Environ. Res. Public Health.* 2020; *17*:5850

48. Mian A, Khan S. Coronavirus: The spread of misinformation. *BMC Medicine*. 2020; 18(1):1-2 doi:10.1186/s12916-020-01556-3 \

49. Pan American Health Organization. Understanding the infodemic and misinformation in the fight against covid-19. [Internet]. Pan American Health Organization 2020 May 1 [cited 2020 Nov 27] Available from: https://www.paho.org/en/documents/understanding-infodemic-and-misinformation-fight-against-covid-19.

50. Bowles J, Larreguy H, Liu, S. Countering misinformation via WhatsApp: Preliminary evidence from the COVID-19 pandemic in Zimbabwe. *PloS one*. 2020; *15*(10): e0240005. https://doi.org/10.1371/journal.pone.0240005

51. Ittefaq M, Hussain SA, Fatima M. COVID-19 and social-politics of medical misinformation on social media in Pakistan. *Media Asia*. 2020; *47*(1-2): 75-80. https://doi.org/10.1080/01296612.2020.1817264

52. The National Law Review. There's a Fake News Pandemic. Could COVID-19 and Trademarks be the Cure? [Internet]. The National Law Review 2020 Jul 7 [cited 2020 Nov 27] Available from: https://www.natlawreview.com/article/there-s-fake-news-pandemic-could-covid-19-and-trademarks-be-cure

53. O'connor C, Murphy M. Going Viral: Doctors Must Combat Fake News in the Fight against Covid-19. *Ir Med J*. 2020; 113(5): 85-85.

54. Pew Research Center. Nearly three-in-ten Americans believe COVID-19 was made in a lab. [Internet]. Pew research center 2020 April 8 [cited 2020 Nov 27] Available from: https://www.pewresearch.org/fact-tank/2020/04/08/nearly-three-in-ten-americans-believe-covid-19-was-made-in-a-lab/

55. Health Analytics Asia. 50 Fake 'frequently forwarded' COVID-19 WhatsApp messages. [Internet]. Health Analytics Asia 2020 April 2 [cited 2020 Nov 27] Available from: https://www.ha-asia.com/50-fake-frequently-forwarded-covid-19-whatsapp-messages/

56. Brennen JS, Simon F, Howard PN, Nielsen RK. Types, sources, and claims of COVID-19 misinformation. *Reuters Institute*. 2020; *7*: 1-13.

57. Pennycook G, McPhetres J, Zhang Y, Lu JG, Rand DG. Fighting COVID-19 misinformation on social media: Experimental evidence for a scalable accuracy-nudge intervention. *Psychol*. 2020; *31*(7): 770-780. https://doi.org/10.1177/0956797620939054

58. Barua Z, Barua S, Aktar S, Kabir N, Li M. Effects of misinformation on COVID-19 individual responses and recommendations for resilience of disastrous consequences of misinformation. *Prog Disaster Science*. 2020; *8*: 100119. doi:10.1016/j.pdisas.2020.100119

59. Ahmed N, Shahbaz T, Shamim A, Khan KS, Hussain SM, Usman A. The COVID-19 Infodemic: A Quantitative Analysis Through Facebook. *Cureus.* 2020; 12(11): e11346. doi: 10.7759/cureus.11346

60. Nagler RH, Vogel RI, Gollust SE, Rothman AJ, Fowler EF, Yzer MC. Public perceptions of conflicting information surrounding COVID-19: Results from a nationally representative survey of US adults. *PloS one*. 2020; *15*(10): e0240776. doi:10.1371/journal.pone.0240776

61. Orso D, Federici N, Copetti R, Vetrugno L, Bove T. Infodemic and the spread of fake news in the COVID-19-era. *Eur J Emerg Med*. 2020; 27(5):327-328. doi:10.1097/MEJ.0000000000000713

62. McNab C. What social media offers to health professionals and citizens. *Bull World Health Organ*. 2009; 87(8):566. doi:10.2471/blt.09.066712

63. Ma X, Vervoort D, Luc JG. When misinformation goes viral: access to evidence-based information in the COVID-19 pandemic. *J. Glob. Health. Sci*. 2020; *2*(1):e13 doi:10.35500/jghs.2020.2.e13

64. Tasnim S, Hossain MM, Mazumder H. Impact of rumors and misinformation on COVID-19 in social media. *J Prev Med Public Health*. 2020; *53*(3):171-174.

65. Bode L, Vraga EK.See something, say something: Correction of global health misinformation on social media. J Health Commu. 2018; *33*(9):1131-1140. doi:10.1080/10410236.2017.1331312

66. Chou WYS, Oh A, Klein WM. Addressing health-related misinformation on social media. *Jama*. 2018; *320*(23): 2417-2418. doi:10.1001/jama.2018.16865.

67. Stasiuk K, Polak M, Dolinski D, Maciuszek J. The credibility of health information sources as predictors of attitudes toward vaccination—the results from a longitudinal study in Poland. *Vaccines*. 2021; *9*(8):933 doi: 10.3390/vaccines9080933

68. Cernicova-Buca M, Palea A. An appraisal of communication practices demonstrated by romanian district public health authorities at the outbreak of the COVID-19 pandemic. *Sustainability*. 2021; *13*(5): 1-19, doi: 10.3390/su13052500

69. Tagliacozzo S, Albrecht F, Ganapati NE. International Perspectives on COVID-19 Communication Ecologies: Public Health Agencies' Online Communication in Italy, Sweden, and the United States. *Am Behav Sci*. 2021; *65*(7), 934-955 doi: 10.1177/0002764221992832

70. Saechang O, Yu J, Li Y. Public trust and policy compliance during the COVID-19 pandemic: The role of professional trust. *Healthcare*. 2021; 9 (2):1-13 doi: 10.3390/healthcare9020151.

71. Lewandowski R, Goncharuk AG, Cirella GT. Restoring patient trust in healthcare: medical information impact case study in Poland. *BMC Health Serv. Res.* 2021; *21*(1):1-11 doi: 10.1186/s12913-021-06879-2

72. Soveri A, Karlsson LC, Antfolk J, Lindfelt M, Lewandowsky S. Unwillingness to engage in behaviors that protect against COVID-19: the role of conspiracy beliefs, trust, and endorsement of complementary and alternative medicine. *BMC Public Health*. 2021; *21*(1): 1-12 doi: 10.1186/s12889-021-10643-w

73. Antinyan A, Bassetti T, Corazzini L, Pavesi F. Trust in the health system and COVID-19 treatment. Front. Psychol.. 2021; *12*:1-14 doi:10.3389/fpsyg.2021.643758

74. Law RW, Kanagasingam S, Choong KA. Sensationalist social media usage by doctors and dentists during Covid-19. *Digit. Health*. 2021; *7*:1-12 doi: 10.1177/20552076211028034

75. Leonard MB, Pursley DM, Robinson LA, Abman SH, Davis JM. The importance of trustworthiness: lessons from the COVID-19 pandemic. Pediatr. Res. 2021; 1-4 doi:10.1038/s41390-021-01866-z

76. Wood JL, Lee GY, Stinnett SS, Southwell BG. A Pilot Study of Medical Misinformation Perceptions and Training Among Practitioners in North Carolina (USA). *INQUIRY: The Journal of Health Care Organization, Provision, and Financing*, 2021; *58*:1-6 doi: 10.1177/00469580211035742

77. Gopichandran V, Sakthivel K. Doctor-patient communication and trust in doctors during COVID 19 times—A cross sectional study in Chennai, India. *Plos One*. 2021: *16*(6), 1-11 doi: 10.1371/journal.pone.0253497

78. Nwoga HO, Ajuba, MO, Ezeoke UE. Effect of COVID-19 on doctor-patient relationship. *Int J Community Med Public Health*. 2020; *7(12)*: 2394-6040, doi: 10.18203/2394-6040.ijcmph20205136

79. Rocha YM, de Moura GA, Desidério GA, de Oliveira CH, Lourenço FD, de Figueiredo NLD. The impact of fake news on social media and its influence on health during the COVID-19 pandemic: A systematic review. *J. Public Health*. 2021; 1-10 doi:10.1007/s10389-021-01658-z

80. Greene CM, Murphy G. Quantifying the effects of fake news on behavior: Evidence from a study of COVID-19 misinformation. *J Exp Psychol Appl*. 2021 Dec;27(4):773-784. doi: 10.1037/xap0000371.

81.Rogozea LM, Sechel G, Bularca MC, Coman C, Cocuz ME. Who's Getting Shots First? Dealing With the Ethical Responsibility for Prioritizing Population Groups in Vaccination. *Am J Ther*. 2021 Jun 22;28(4):e478-e487. doi: 10.1097/MJT.0000000000001400. PMID: 34228653.

82. Rogozea L, Purcaru D, Leaşu F, Nemet C. Biomedical research - opportunities and ethical challenges. *Rom J Morphol Embryol.* 2014;55(2 Suppl):719-22. PMID: 25178352.

83. Olimid AP, Rogozea LM, Olimid DA. Ethical approach to the genetic, biometric and health data protection and processing in the new EU General Data Protection Regulation (2018). *Rom J Morphol Embryol*. 2018;59(2):631-636. PMID: 30173275.

Supporting information

S1 Appendix English version of the questionnaire

(docx)

S2 Appendix Romanian version of the questionnaire

(docx)

Click here to access/download

Supporting Information - Compressed/ZIP File Archive

Supporting information.zip

Challenges in the communication process during the COVID-19 pandemic- a perspective of medical staff

Claudiu Coman[1#a*], Maria Cristina Bularca[1], Angela Repanovici[2], Liliana Rogozea[3]

1 Department of Social Sciences and Communication, Faculty of Sociology and Communication, Transilvania University of Brasov, Brasov, Romania;

2 Department of Product Design, Mechatronics and Environment, Faculty of Product Design and Environment, Transilvania University of Brasov, Brasov, Romania

3 Basic, Preventive and Clinical Sciences Department, Transilvania University of Brasov, Brasov, Romania;

[#a] Current address: Department of Social Sciences and Communication, Faculty of Sociology and Communication, Transilvania University of Braşov, Brasov, România

[*] Corresponding author

E-mail: claudiu.coman@unitbv.ro (CC)

Abstract

Background. Healthcare professionals had to face numerous challenges during the pandemic, their professional activity being influenced not only by the virus, but also by the spread of medical misinformation. In this regard, we aimed to analyze, from the perspective of medical staff, the way medical and non - medical information about the virus was communicated during the pandemic in order to raise awareness about the way misinformation affected the medical staff.

Methods and findings. The study was conducted on Romanian healthcare professionals including doctors, nurses and medical students. They were asked to answer to a questionnaire and the sample of the research includes 536 respondents. The findings revealed that most respondents stated that information about alternative treatments against the virus affected the credibility of health professionals, and that younger professionals believed to a greater extent that trust in doctors was affected. The research also showed that respondents were well informed about the drugs used in clinical trials in order to treat the virus, and that younger respondents believed that social media should be used to send official information. Among the main limitations of our study we mention the fact that we used only quantitative methods and the fact we focused only on Romanian healthcare professionals.

Conclusions. Healthcare professionals declared that the spread of misinformation regarding alternative treatments, affected their credibility and the relationship with their patients. Healthcare professionals had knowledge about the drugs used in clinical trials, and they acknowledged the role of social media in spreading medical misinformation. However, younger professionals also believed that social media could be used to share official information about

the virus. A future research should focus on studying the opinion of Romanian and international doctors, it should use qualitative methods too and should address the issue of social media being an appropriate environment for sending official information.

Introduction

The COVID 19 pandemic generated multiple changes in the way today's society members carry out their daily activities. While many domains were affected by the spread of the virus, such as the educational system or the cultural sector, the health sector was the one that faced the most challenges, the pandemic managing to generate a tremendous global public health crisis [1].

Caused by severe acute respiratory syndrome coronavirus 2 (SARS-CoV-2) [2], the disease was firstly detected in December 2019, in Wuhan, China [3], and it fastly spread all over the world. The World Health Organization was informed about a pneumonia outbreak in Wuhan on December 31 2019, the number of cases continued to increase, and on March 11 2020 the World Health Organization characterized COVID 19 as a pandemic [4]. Being highly contagious, the virus affected a large number of people, and as of November 27 over 61 million cases were reported [5]. Even though many companies and institutions are struggling to develop a vaccine, Pfizer, Gamaleya Research Institute, University of Oxford, and a preliminary analysis of the vaccine proposed by Pfizer showed that the vaccine is able to prevent more than 90% of people from getting infected with COVID 19 [6], so far no vaccine was approved as a general and universal vaccine against COVID 19 [7].

Ever since the pandemic was declared, finding the right treatment for the virus has become a priority for researchers and doctors from all over the world. In this regard, large

number of trials started to be conducted, and in order to find an efficient drug treatment against the virus, one method that was adopted was testing and administrating to patients, drugs that were previously used for curing other viruses [8]. Thus, on March 20 2020, The World Health Organization launched the SOLIDARITY clinical trial, a trial that monitored the effects on patients infected with COVID 19, of specific drugs that proven to be effective in the treatment of other diseases: remdesivir, interferon beta, chloroquine and hydroxychloroquine - previously used for Malaria, as well as drugs used on HIV patients: lopinavir and ritonavir [9]. However, according to the interim results published on October 15 2020 by WHO, even though those drugs were taught to have positive effects on treating COVID 19, they had little influence or no influence at all on mortality in general, on the need and initiation of ventilation and on the recovery process [10].

With the development of many trials and programs meant to find a cure for COVID 19 and with the use of diverse drug combinations, another major problem arose: misinformation and fake news about the virus, its treatment or methods to combat it. In this regard, along with the pandemic, people also had to face an epidemic of information, described by the general director of WHO as an „infodemic" [11]. In other words, information about COVID 19 began to be spread by people on every available communication channel, both in the online and offline environment. However, very often and especially on social media, the information was poorly communicated, it was distorted and there usually wasn't enough scientific evidence to demonstrate its validity [12].

Taking into account the previously mentioned aspects the paper addresses the issues of drugs tested and used for the treatment of COVID 19 and how information about COVID 19 was

communicated in the offline and online environment. The purpose of the paper is to analyze, from the perspective of medical staff, the way medical and non - medical information about the virus was communicated during the pandemic in order to raise awareness about the way misinformation affected medical staff. Thus, the paper aims at finding an answer to three research questions: (1) to what extent information about alternative treatments affected the credibility of medical staff? (2) What is the knowledge of medical staff about the type of drugs that had positive effects on treating the disease and about alternative treatments? (3) How satisfied is the medical staff with the way medical and non-medical information was communicated online and offline during the pandemic? (4) What is the perception of medical staff about the role of social media in spreading misinformation about the virus? (5) What aspects of the professional activity of the medical staff were affected most by the COVID – 19 pandemic?

Hence, considering the purpose of our paper and the research questions, we believed it was necessary to analyze the literature on the drugs used to treat COVID – 19, on the role of social media platforms in spreading fake information about the virus and potential treatments, and on the way the pandemic influenced the credibility of doctors and their relationship with their patients.

Literature review

Information on drugs used to treat COVID 19

Before analyzing the way information about the virus was communicated in the online environment, it is important to take a look at the drugs used to treat the disease. Hence, one of the most important issues that appeared with the COVID 19 pandemic, was finding the right

treatment for the virus. In this regard, researchers started to develop many experimental trials and used diversified drug combinations in order to treat patients with COVID 19. However, information that was communicated about the effectiveness of certain drugs was often contradictory.

Chloroquine and hydroxychloroquine are two drugs that were tested and included in many trials. Both drugs were previously used to treat malaria but they also have antiviral effects on viruses like HIV since they have the ability to prevent the virus to enter in the host cells [13]. Even though they have similar compounds, chloroquine is taught to have more negative effects than hydroxychloroquine [14], and hydroxychloroquine is considered safer due to the fact that it can be tolerated better for a longer period of time [15].

While some studies show positive effects of hydroxychloroquine in inhibiting the infection with the virus in vitro [16, 17], other studies found no influence of the drug on mortality rate or time spent by patients in the hospital [18]. However, when hydroxychloroquine was combined with other drugs such as azithromycin, it showed beneficial effects in treating patients with COVID 19 [19].

Nonetheless the findings regarded the effectiveness of these drugs were contrasting. For example, on March 28 2020 the Food and Drug Administration (FDA) issued an Emergency Use Authorization for using hydroxychloroquine in treating people suffering from COVID 19 [20], and in June 15 2020, the FDA retracted the authorization stating that the trials in which the drug was involved showed that the drug had no effect on the faster recovery of patients or on decreasing chances of death [21]. Even more, on 5th June 2020 the UK trial, Randomised Evaluation of COVID 19 THERAPY (RECOVERY), also stopped testing the drug on patients

because the results showed no benefits in improving the conditions of hospitalized patients with COVID 19 [22].

Studies were carried out with other drugs such as lopinavir/ritonavir, an antiviral drug used in the treatment of HIV [23]. While in concentration of 4 μg/ml and 50 μg/ml, the drug showed positive effects against the virus in vitro [24], a study on 199 patients, from which 99 received the drug and the other 100 did not receive the drug, revealed that lopinavir/ritonavir had no benefits when it comes to diminishing mortality or improving the state of patients with severe symptoms [25].

Controversial discussions also involved the use of Ibuprofen, a Non-steroidal anti-inflammatory drug that is used to treat fever, or inflammation [26]. Since the pandemic was declared there has been a preoccupation regarding ibuprofen and its role in making people more vulnerable to contacting the virus. Thus, right after the declaration of the pandemic, in a letter addressed to The Lancer Journal, researchers pointed out that ibuprofen could make people with diabetes, cardiac disease or hypertension more likely to get infected with virus and have severe symptoms [27]. However, while firstly, WHO recommended people who are infected with the virus not to take ibuprofen, only one day after that recommendation, on 18 March 2020, WHO corrected its statement and mentioned that it ”does not recommend against ibuprofen” [28]. Even more, a study focusing on the use of ibuprofen showed that the drug does not make patients feel worse [29] and another study that analyzed the use of ibuprofen and paracetamol of 403 COVID 19 confirmed patients revealed that compared to paracetamol, ibuprofen did not aggravated the clinical state of the patients [30].

While other drugs failed to show beneficial effects on the treatment of COVID 19, drugs like dexamethasone, which is included in the UK RECOVERY trial, revealed positive effects on people suffering from COVID 19: the drug lowered the risk of death in patients on ventilators from 40% to 28% and in patients who were in need of oxygen, from 25% to 20%, but did not influence the state of patients who did not need oxygen [31, 32].

Another highly tested drug was Remdesivir, an antiviral drug produced by Gilead Sciences that was previously used in treating Ebola [33]. The information regarding its positive effects on treating COVID 19 is also contradictory. A study conducted from February 6 2020 until March 12 2020, on 237 patients, showed that the drug did not bring any benefits for people that had severe symptoms of COVID 19 [34], while a more recent study revealed that Remdesivir had a more positive effect in reducing the time of recovery in patients with COVID 19 that showed signs of respiratory issues, than it had the placebo effect [35]. However, the FDA approved on October 22 2020, the use of Remdesivir in the case of adults and also children aged 12 or older who have at least 44 kilograms, who are infected with the virus and need to be treated in the hospital [36], and as of November 20 2020, FDA allows, in emergency cases, the use of Remdesivir in combination with Baricitinib, for adults and children aged two or older that require oxygen and treatment in the hospital [37].

Social media and COVID 19 misinformation

Together with the health crisis, the COVID 19 pandemic generated an information crisis, often described as an infodemic, that is represented by the spread of fake news, misguided and false information, especially in the online environment [38].

In this context, social media plays an essential role in disseminating information. Social media consists of internet based channels that provide people with the opportunity to interact, communicate in asynchronous way and in real time, with either small or large audiences where value is derived from user generated content [39]. Social media comprises multiple social networks, which according to Boyd and Ellison, offer users the possibility to create profiles that are public, or semi-public, to create a list of people with whom they can interact and share information and to view the list of connections that other users make [40].

Social media channels are often used in time of crisis not only by citizen, but also by official authorities, emergency services, because they can facilitate communication and the spread of valuable information that can contribute to surpassing the crisis [41]. Social networks like Facebook, Whatsapp, Twitter, Instagram can function as sources that have the ability to confirm or complete the information communicated by the authorities, while also receiving feedback from the public [42]. Thus, sending messages through social media channels is a strategy that can help authorities obtain feedback on certain proposals regarding public health policies [43]. Even more, a study regarding the influence of social media on the way people protect their health during the pandemic, showed that social media can have positive impact on increasing awareness about public health and protection against the virus [44].

However, during the pandemic, while authorities can use social media to keep the public informed, a major issue generated by social media, that public health representatives have to face, is the spread of fake news [45].

Fake news are represented by fabricated information designed in the form of news communicated by the media that do not share the same process of organization and do not

have the same intent, and fake news are related to misinformation: information that is false or misleading, and disinformation: a type of false information whose aim is to deceive people [46].

Thus, the internet became a favorable environment for spreading conspiracy theories or false information about alternative treatment for the virus. Since people were stressed and frightened by the uncertainty of the situation, they started to consider reasonable and valid any information that presented explanations in regards to the virus [47]. Thus, when referring to health information, false news often undermine the credibility of official sources, they create confusion among people and favor the faster spread of the virus [48].

Misinformation during the pandemic can negatively influence peoples' health because false information is not easy to recognize, because it can determine people to change their behavior in a way that is harmful to their health and those around them. Thus, since the pandemic was declared, false information has been spread about the origin of the virus, about what caused it, how it spreads and what treatment is efficient for eliminating it [49]. However, a study focusing on the WhatsApp platform showed that when the information on social media is shared by trusted sources, it can increase knowledge about the virus and encourage people to adopt preventive behavior [50].

During the time of crisis, on platforms like WhatsApp or Facebook, more and more false news and unverified information about the virus began to be shared. With millions of users worldwide, WhatsApp became one of the platforms where most fake news were shared by forwarding messages to many users [51], while Facebook was characterized as the core, epicenter of misinformation [52].

When it comes to health misinformation on social media, the most discussed subjects are alternative cures involving certain food or drinks, hygiene related actions and treatment drugs. Thus, among the most “recommended” practices for preventing or curing COVID were drinking hot water every 15 minutes in order for the virus to go into the stomach, eating garlic, taking vitamin C or even pointing a hairdryer to the nostrils because the heat could eliminate the virus [53].

False news that circulated on social media regarding the virus also involve the idea that the virus was created on purpose in a lab, three in ten Americans considering true this information [54].

However, many other unverified methods were shared and the most forwarded messages on WhatsApp presented information about the fact that if people hold their breath for ten seconds without coughing then they are not infected with the virus, about the idea that at temperatures of 30-35 Celsius degrees the virus will die, messages about the release of the vaccine or about drugs allegedly recommended by Chinese doctors that could be efficient in eliminating the virus [55].

Nonetheless, misinformation became a major issue in the context of the pandemic, but also a subject of interest for researchers. A study focusing on the spread of fake news showed that most news reconfigure and twist the original information thus creating a different context, and that most of them contain false information about public authorities and health organizations [56].

Another study found that people who tend to rely on their intuition or who possess little scientific knowledge about certain subjects, encountered difficulties in differentiating true and

false information [57]. Thus, misleading or unverified information can negatively influence the way people behave. For example, people in USA who died after they consumed chloroquine may have used the drug because news about it mentioned that it could treat and eliminate the virus [58]. Even more, a study concerning misinformation on Facebook revealed that posts made from verified accounts contained more false information than the accounts that were not verified [59], while other study conducted from 23 April 2020 to 27 April 2020, focused on perception about contradictory information and stated that 73% of participants mentioned they observed or were exposed to contrasting messages usually communicated by politicians or health experts [60].

Apart from influencing peoples' beliefs or health practices, COVID 19 fake news also influenced the activity of health professionals. Social media managed to increase the level of trust in information that comes from people's personal opinions rather than professionals [61], and doctor's credibility is often affected. In order to improve these situations, doctors must be willing to use social media not just to send messages, but to actively communicate with people, to offer feedback, to share their experiences and rectify and clarify the fake news presented on social media [62].

Among action from health professionals, in order to combat COVID 19 fake news, social media networks as well as public authorities must implement some strategies. For example, the government of United Kingdom developed collaboration programs between its rapid response teams and social media platforms, and Taiwan introduced greater fines for news that were proven to be false [63]. Moreover, even though some social networks such as Facebook or Twitter already implemented algorithms to identity and remove fake accounts [64], or to

correct information [65], they should further develop efficient strategies in order to validate the information that people share [66].

The influence of the pandemic on doctors' credibility and relationship with patients

The way information regarding the virus was communicated online and offline during the pandemic played an essential role in the process of maintaining trust in health professionals. In this regard, a previous longitudinal study conducted in Poland revealed that trust in physicians has declined from 2018 – 2020, and emphasized the idea that the decrease may be caused by the health problems that people had to cope with during the pandemic and the problems with the healthcare system of the country [67]. In Romanian context, a previous study showed that the communication process of the healthcare system was poor and confusing, and that public health authorities at national level focused more on global information about the virus, while local authorities failed to succeed in providing their "share of information" [68]. Another study, which focused on analyzing the online communication of Public Health Agencies from Italy, United States and Sweden, revealed that compared to Sweden and the United States, agencies from Italy collaborated more with other organizations, and that overall, the communication process of the agencies was coordinated by their members, that agencies also communicated with governments, but they rarely collaborated with political or non-governmental organizations [69]. Hence, while trust in the government and communication from authorized organizations is essential, the importance of trusting the professionals is highlighted by a study conducted in Thailand, which showed that in the cases in which people have low levels of trust

in the government, trust in professionals can have a positive influence on the adoption of protective measures at the individual level [70].

Furthermore, another previous study conducted in Poland, revealed that information can have the power to influence the level of trust that people have in the healthcare system and in healthcare professionals, suggesting that an increase of trust in hospitals, may be associated with a decrease of trust in physicians [71].

While focusing on studying people's response to non- pharmaceutical interventions, conspiracy theories and alternative treatments, a study conducted in Finland showed that the level of trust people have in the system implemented in order to provide information about the virus, has an essential role in the way people react to the official measures recommended. Hence, most participants in the study were between 40 and 60 years of age, and the study emphasized that people who were less willing to comply with the non-pharmaceutical interventions implemented by the government, tended to believe more in conspiracies and had low levels of trust in the sources which provided information about the virus [72].

Another study, which focused on examining the relationship between trust in the healthcare system and people's choice of seeking medical help when they experienced COVID – 19 symptoms, concluded that high levels of trust in the healthcare system can increase the probability of asking for medical help when people first notice COVID – 19 symptoms [73].

Taking into account the aspects mentioned above, we can infer that peoples' trust in doctors was affected during the pandemic. In this regard, in the context of misinformation, one of the reasons why people lost trust in doctors may be the fact that, besides using social media for communicating information, for networking or for interacting with patients, many medical

or dental practitioners used social media to express their professional opinions about the virus, opinions which were not validated and which later proven to be inaccurate [74]. In other words, health professionals may have contributed to the spread of misinformation, and such behavior can contribute to the decrease of trust in medical processes and in healthcare professionals [75]. Other researchers who focused on examining medical misinformation, found that most doctors (94.2%) stated that patients had medical misinformation, and the subjects about they had the most inaccurate information were represented by COVID – 19 vaccines, COVID – 19 origin, treatment or essential oils [76]. Furthermore, a previous study discovered that trust in doctors increased with age, and communication difficulties decreased, and that trust in doctors decreased while the level of education and communication difficulties increased [77].

Hence, while acknowledging that the pandemic influenced the trust in medical professionals, another aspect that was negatively influenced was the relationships between doctors and their patients. A study which focused on examining the doctor – patient interaction from the perspective of both groups of people, revealed differences in the respondents' opinions. Thus, most doctors stated that they still make eye contact (72%) and that they still show patients empathy, but only few patients declared that their doctors made eye contact (56,8%) or showed them empathy (43,2%) [78].

Methods and materials

Sampling and data collection procedures

The present study was conducted on Romanian healthcare professionals including doctors, nurses and medical students. The questionnaire was administered online, the data was collected through the help of Google forms, and was disseminated on groups of healthcare

professionals and students on platforms such as Facebook and WhatsApp, during the period April 2021– June 2021. The data we collected was firstly exported to Microsoft Excel, and then it was analyzed with IBM Statistical Package for the Social Sciences, version 20. At the beginning of the questionnaire, the respondents were informed about the purpose of the study, about the fact that they were allowed to withdraw at any time, and they were asked to give their consent for participating in the study. The average time needed to complete the questionnaire was 15 minutes, and the research received approval from The Council of the Faculty of Sociology and Communication, approval request Nr.378/30.03.2021.

The sample of our study comprises 536 respondents. Out of the 536 respondents, 460 (85.8%) were female and 76 (14.2%) were male. A total of 411 respondents live in the urban area (76.7%), while 125 (23.3%) live in the rural area. Most respondents (286, 53.4%) are between 18 and 35 years of age, 142 respondents (26.5%) are between 36 and 50 years of age, 102 respondents (19.0%) are between 51 and 65 years of age, and 6 of them (1.1) are over 65 years of age. When it comes to the professional degree of the respondents, most of them are students at a university nursing program (122, 22.8%), and medical students (120, 22.4%). However, a total of 102 respondents (19.0%) are senior specialists medical – doctors, and 70 (13.1%) are nurses who have a higher education diploma. When it comes to the respondents field of specialization, most of them (70.5%) operate in the field of general medicine, while others are family doctors (10.4%), pediatricians (3%), dentists or oncologists (1.9%), surgeons of doctors who are specialized in internal medicine (1.5%), or infectious disease doctors, radiologists or cardiologists (1.1%). Furthermore, most of the respondents (77.2%) stated that they did not work a unit with COVID – 19 patients while few of them (22.8%) stated that they

worked in such a unit at the time the research was conducted. Thus, all the characteristics of the sample are presented in Table 1.

Table 1. Sample characteristics (n = 536).

	Category	Count	Percentage
Gender	Female	460	88.8%
	Male	76	14.2%
Living environment	Urban	411	76.7%
	Rural	125	23.3%
Age	18-35 years old	286	53.4%
	36-50 years old	142	26.5%
	51 -65 years old	102	19.0%
	Over 65 years old	6	1.1%
Professional degree	Senior specialist medical - doctor	102	19.0%
	Specialist medical - doctor	46	8.6%
	Resident	28	5.2%
	Nurse with higher education diploma	70	13.1%
	Nurse with other studies than higher education	48	9.0%
	Medical student	120	22.4%
	Student at university nursing program	122	22.8%
Field of specialization	General medicine	378	70.5%
	Family doctor	56	10.4%
	Pediatrics	16	3%
	Stomatology	10	1.9%
	Oncology	10	1.9%
	Surgery	8	1.5%
	Internal medicine	8	1.5%
	Virology/ infectious disease doctor	6	1.1%
	Cardiology	6	1.1%
	Radiology	6	1.1%

	Other	32	6%
Works in a unit with COVID – 19 patients	Yes	122	22.8%
	No	414	77.2%

The research instrument

In order to conduct the research we used a quantitative method while having a questionnaire as an instrument. In this regard, we developed a questionnaire which comprises four sections: A. Influence of the pandemic on the professional activity of medical staff (items A1 to A4), B. Perception about the authorities' communication process (items B1 to B11), C. Perception about the communication of non- validated treatments (items C1 to C20), and D. Sociodemographic questions (items D1 – D9), such as: gender, age, living environment, professional degree, field of specialization. The sociodemographic questions were used in order to identify different or similar attitudes between specific groups. The questionnaire can be found in "S1.Appendix English version of the questionnaire", and in "S2. Appendix Romanian version of the questionnaire." Before disseminating the questionnaire, the instrument was tested on 30 doctors who work in the field of cardiology and general medicine. The respondents understood clearly the questions and did not report any issue in the process of answering them. Hence, the questionnaire comprises close ended and open ended questions (Items A1, A4,B3, B11, C19, C20, D2, D5, D6,) dihotomic questions as well as questions whose answers were measured on a 7 point Likert scale. For example, item A2 measured the extent to which the respondents considered that the pandemic influenced the way they carried out their

professional activity (1- “to an extremely little extent, 7 “to an extremely great extent”), or item B2 measure the respondents’ level of agreement with statements regarding the way authorities communicated during the pandemic (1 – “strongly disagree, 7-“ strongly agree”).

Data analysis

Data was analyzed with IBM Statistical Package for the Social Sciences, version 20. In order to analyze the data and identify differences and similarities between the attitudes of certain groups, t tests for independent samples were performed. The t test were performed among groups: male/female, working in unit with COVID – 19 patients/ not working in unit with COVID – 19 patients, urban/rural area, and professional degree: medical staff/students. Hence, in order to be able to analyze the results depending on professional degree, we computed the variable of professional degree which had the following values: senior specialist medical – doctor, specialist medical – doctor, resident, nurse with higher education diploma, nurse with other studies than higher education, medical student, student at university nursing program, in a new variable. Thus, doctors, nurses and residents, were integrated in a new group called “medical staff”, while medical students and students at university nursing programs were integrated in the group “students”. Moreover, for a better understanding of the way some variables correlate with each other, (for example: respondents satisfaction with the way authorities communicated during the pandemic and age, respondents’ opinion about the way misinformation about alternative treatments influenced doctors’ credibility and age), we also calculated the Pearson coefficient.

Results

1) To what extent information about alternative treatments affected the credibility of medical staff?

The results of our research revealed that respondents were of the opinion that information about alternative treatments for COVID -19 affected the credibility of healthcare professionals. Hence, most respondents (32.5%), stated that trust in healthcare professionals was affected to a an extremely great extent by the information about alternative treatments, many of them declared that credibility was affected to a very great extent (23.1%), and to a great extent (21.3%) (Table 1).

Table 1. The extent to which information about alternative treatments affected trust in physicians

		Frequency	Percent	Valid Percent	Cumulative Percent
Valid	to an extremely little extent	14	2.6	2.6	2.6
	to a very little extent	10	1.9	1.9	4.5
	to a little extent	42	7.8	7.8	12.3
	nor to a little, neither to a great extent	58	10.8	10.8	23.1
	to a great extent	114	21.3	21.3	44.4
	to a very great extent	124	23.1	23.1	67.5
	to an extremely great extent	174	32.5	32.5	100.0
	Total	536	100.0	100.0	

Furthermore, the Pearson correlation performed between the extent to which respondents believed that information about alternative treatments affected people's trust in doctors and the age of the respondents, revealed a weak, negative and statistically significant correlation between the two variables ($r(534) = -.155$, $p=0.001$) (Table 2). Hence, as the age of

the medical staff decreases, the extent to which they believe the credibility of doctors was affected increases. In other words, compared to older healthcare professionals, younger healthcare professionals tend to believe more that information about alternative treatments affected trust in doctors. One possible explanation for this result can be that younger people tend to be fonder of keeping up with trends and being up to date, and in this context, it is possible that they came into contact more frequently with information about certain alternative treatments for COVID – 19, this making them more aware about the way such treatments can undermine doctor's credibility.

Table 2. Pearson correlation between information about alternative treatments and age

		C14. The extent to which information about alternative treatments affected trust in physicians	D2. Age
C14. The extent to which information about alternative treatments affected trust in physicians	Pearson Correlation	1	-.155**
	Sig. (2-tailed)		.000
	N	536	536
D2. Age	Pearson Correlation	-.155**	1
	Sig. (2-tailed)	.000	
	N	536	536

**. Correlation is significant at the 0.01 level (2-tailed).

In order to observe if there any differences in the opinion of the respondents depending on certain variables including, age, gender, or living environment, we performed t tests for independent samples. The results of the significant t tests (Table 3), showed that students believed to a greater extent (M= 5.60, SD=1.49), that information about alternative treatments negatively affects the credibility of doctors, than the medical staff (M=5.33, SD=1.54). Also, respondents who declared they worked in a unit without COVID – 19 patients (M=5.53,

SD=1.49), were more of the opinion that information about alternative cures affected trust in health professionals, than respondents who worked in a unit with COVID – 19 patients (M=5.19, SD=1.61). One possible explanation would be that, doctors who interacted with COVID – 19 patients may have observed that when being put in the situation to receive medical care in the hospital, patients still had faith and trust in doctors. Moreover, another explanation is that respondents who did not come into contact with COVID – 19 patients were not that close with the situation and thus they might have had a more distorted perception about the situation than those professionals who interacted with COVID – 19 patients. Moreover, the results of the research also showed that female respondents (M=5.51, SD=1.48), believed more than male respondents (M=5.10, SD=1.70), that trust in healthcare professionals was affected by the information about alternative treatments.

Table 3. Significant t-test results: comparisons between variables

					t-test for Equality of Means						
	Group	N	Mean	S. D.	t	df	p	Mean Difference	Std. Error Difference	CI4 Lower	CI4 Upper
Information about alternative treatments _ Professional degree[1]	Medical staff	294	5.33	1.54	-2.04	534	.04	-.27	.13	-.52	-.01
	Student	242	5.60	1.49							
Information about alternative treatments _working unit	Unit with COVID -19 patients	122	5.19	1.61	-2.13	534	.03	-.33	.15	-.64	-.02
	Unit without COVID 19 patients	414	5.53	1.49							
Information about alternative treatments _gender	Male	76	5.10	1.70	-2.16	534	.03	-.40	.18	-.77	-.03
	Female	460	5.51	1.48							

[1]Index variable from the professional degrees of respondents. Student: medical student and student at university nursing program, Medical Staff: Senior specialist medical – doctor, Specialist medical – doctor, Resident, Nurse with higher education diploma, Nurse with other studies than higher education

2) What is the knowledge of medical staff about the type of drugs that had positive effects on treating the disease and about alternative treatments?

Considering the type of drugs which were known to have positive effects on treating the virus, the research revealed that type of drug about which the respondents have heard it had positive effects against the virus was Dexamethasone (46.6%), closely followed by Remdesivir (40.5%) and Azithromicin (38.4%). However, some of the respondents also mentioned Chloroquine, Hydroxychloroquine (23.1%), Ibuprofen (19.8%), Tocilizumab (15.9%), and Favipiravir (13.8%) as drugs known to have positive effects when dealing with COVID – 19 (Table 4). Hence, the research showed that the medical staff had knowledge about the type of drugs tested or used against the virus, which were taught to be efficient in treating the disease.

Table 4. Drugs known to have positive effects in treating the virus: the perception of medical staff

	**Frequency**	**Valid percent**
Amoxicillin	36	6.7%
Azithromicin	206	38.4%
Chloroquine, Hydroxychloroquine	124	23.1%
Dexamethasone	250	46.6%
Doxycycline	32	6.0%
Favipiravir	74	13.8%
Ibuprofen	106	19.8%
Lopinavir/Ritonavir	56	10.4%
Oseltamivir, Peramivir or Zanamivir	32	6.0%
Remdesivir	217	40.5%
Tocilizumab	85	15.9%

Umifenovir	17	3.2%

In the context of respondents' perception about alternative methods of preventing and treating the virus, the findings show that, most of them stated that they heard about the fact that alcohol consumption can prevent the infection with the virus (24.3%), that drinking warm water every 15 minutes may help eliminate the virus (21.3%), but also that pointing the hot air of the hairdryer to the nostrils leads to the elimination of the virus (16.8%) (Table 5).

Table 5. Medical staff's knowledge about alternative methods of preventing and treating the virus

		Frequency	Percent	Valid Percent	Cumulative Percent
Valid	drinking alcohol helps you eliminate the virus	79	14.7	14.7	14.7
	drinking alcohol prevents the infection with the virus	130	24.3	24.3	39.0
	rinsing the nostrils with disinfectant eliminates the virus	81	15.1	15.1	54.1
	drinking hot water every 15 minutes eliminates the virus	114	21.3	21.3	75.4
	pointing hot air to the nostrils leads to the elimination of the virus	90	16.8	16.8	92.2
	other	42	7.8	7.8	100.0
	Total	536	100.0	100.0	

3) How satisfied is the medical staff with the way medical and non-medical information was communicated during the pandemic?

The findings of the study revealed that respondents were mostly dissatisfied with the way medical and non – medical information was communicated during the pandemic. Hence, the

sum of the responses with negative valences of the study participants (extremely dissatisfied, very dissatisfied and dissatisfied), showed that 238 of them, (44.4%) were dissatisfied with the process of sending medical and non- medical information, while the sum of the positive responses (satisfied, very satisfied, extremely satisfied) showed that 162 of them (30.2%), were satisfied with the communication process (Table 6). In other words, the study highlighted that respondents registered mostly low level of satisfaction with the way information was sent during the pandemic.

Table 6. The level of satisfaction with the way information about drugs used to treat the virus were communicated at national level

		Frequency	Percent	Valid Percent	Cumulative Percent
Valid	extremely dissatisfied	52	9.7	9.7	9.7
	very dissatisfied	76	14.2	14.2	23.9
	dissatisfied	110	20.5	20.5	44.4
	Nor dissatisfied, neither satisfied	136	25.4	25.4	69.8
	satisfied	108	20.1	20.1	89.9
	very satisfied	30	5.6	5.6	95.5
	Extremely satisfied	24	4.5	4.5	100.0
	Total	536	100.0	100.0	

Furthermore, in the context of the medical staff's satisfaction with the way information about drugs used to treat the virus was communicated at national level, the research showed that as age of the respondents decreases, the level of satisfaction increases ($r(534)= -.091$, $p=0.035$) (Table 7). Thus, according to this result, it can be inferred that younger people were more satisfied than older people, with how information about drugs used to treat the virus was communicated.

Table 7. Pearson Correlation: satisfaction with the way information about drugs used to treat the virus was communicated and age

		B10. Satisfaction with the way information about drugs used to treat the virus was communicated	D2. Age
B10. Satisfaction with the way information about drugs used to treat the virus was communicated	Pearson Correlation	1	-.091*
	Sig. (2-tailed)		.035
	N	536	536
D2. Age	Pearson Correlation	-.091*	1
	Sig. (2-tailed)	.035	
	N	536	536

*. Correlation is significant at the 0.05 level (2-tailed).

Moreover, when asked to evaluate the efficiency of the communication strategies adopted by authorities in order to send information about the virus, most respondents stated that the strategies were effective. Thus, the sum of the responses with negative valences shows that 144 of them (26, 9%) described the communication strategies as inefficient, while 266 of them (49, 6%) described them as efficient (Table 8). One interesting result of the analysis, was that, when trying to examine if the responses of the study participants about the efficiency of communication strategies differ depending on certain variables such as working unit, gender, working unit, living environment, the analysis found no differences between the responses of males and females, of people working in units without COVID – 19 patients and people not working in units with COVID – 19 patients, or in people from the rural and urban area.

Table 8. Perception about the efficiency of communication strategies adopted by authorities

		Frequency	Percent	Valid Percent	Cumulative Percent
Valid	Extremely inefficient	22	4.1	4.1	4.1
	very inefficient	38	7.1	7.1	11.2
	inefficient	84	15.7	15.7	26.9
	nor efficient, neither inefficient	126	23.5	23.5	50.4
	efficient	134	25.0	25.0	75.4
	very efficient	80	14.9	14.9	90.3
	extremely efficient	52	9.7	9.7	100.0
	Total	536	100.0	100.0	

In the context of the information about drugs tested and used in the treatment against COVID – 19, the results showed that students believe to a greater extent that such information was communicated in a coherent manner (M=4.05, SD=1.63), than the medical staff (M=3.79, SD=1.53) (t(534)= -2.05, p<0.05) (Table 9.). Hence, one possible explanation for this result would be that, due the experience and knowledge of the medical staff, people who were already working in the healthcare system, such people have greater expectations from authorities when it comes to sending medical information, than medical students.

Table 9. Significant t test for information about drugs used to treat the virus and professional degree

	t-test for Equality of Means										
	Group	N	Mean	S. D.	t	df	p	Mean Difference	Std. Error Difference	CI4 Lower	CI4 Upper
Information about drugs tested and used to treat the disease[1] _ Professional degree[2]	Medical staff	294	3.79	1.53	-2.05	534	.03	-.28	.13	-.55	-.01
	Student	242	4.05	1.63							

[1] The extent to which respondents believe that information about drugs tested and used to treat the virus was communicated in a coherent manner

[2] Index variable from the professional degrees of respondents. Student: medical student and student at university nursing program, Medical Staff: Senior specialist medical – doctor, Specialist medical – doctor, Resident, Nurse with higher education diploma, Nurse with other studies than higher education

(4) What is the perception of medical staff about the role of social media in spreading misinformation about the virus?

The results of the research revealed that respondents were inclined to believe more that social media was a proper environment for spreading fake medical information during the pandemic. By analyzing the information from Table 10, it can be observed that the sum of the responses with negative valences (4.5%) (to an extremely little extent, to a very little extent and to a little extent) is much lower than the sum of the responses with positive valences (89.9%) (to an extremely great extent, to a very great extent, to a great extent). Hence, most participants of the study believe that social media platforms favored the transmission of fake medical news during the pandemic. Furthermore, when trying to find differences in the responses of the participants depending on age, gender, living environment, professional degree or working unit (with COVID – 19 patients or without COVID – 19 patients), we observed that their responses did not differ depending on such variables. Thus, it can be inferred that, regardless of age, gender, living environment, professional degree or working unit, respondents' perception was that social media had a role in spreading fake medical information.

Table 10. Perception about the extent to which social media contributed to the spread of medical fake news

		Frequency	Percent	Valid Percent	Cumulative Percent
Valid	to an extremely little extent	2	.4	.4	.4
	to a very little extent	10	1.9	1.9	2.2
	to a little extent	12	2.2	2.2	4.5

	nor to a little, neither to a great extent	30	5.6	5.6	10.1
	to a great extent	62	11.6	11.6	21.6
	to a very great extent	88	16.4	16.4	38.1
	to an extremely great extent	332	61.9	61.9	100.0
	Total	536	100.0	100.0	

However, even though respondents were of the opinion that social media was an environment in which was sent fake medical information, some of them still believe that social media platforms are appropriate for sending official information about the virus. Thus, considering the results from Table 11, the sum of responses with positive valences (40.3%) is almost equal to the sum of responses with negative valences (45.1%) meaning that the opinions of the study participants were divided when it comes to sending official information about the virus on social media.

Table 11. Perception about the extent to which social media represents an appropriate environment for sharing official COVID – 19 information

		Frequency	Percent	Valid Percent	Cumulative Percent
Valid	to an extremely little extent	86	16.0	16.0	16.0
	to a very little extent	78	14.6	14.6	30.6
	to a little extent	52	9.7	9.7	40.3
	nor to a little, neither to a great extent	78	14.6	14.6	54.9
	to a great extent	72	13.4	13.4	68.3
	to a very great extent	74	13.8	13.8	82.1
	to an extremely great extent	96	17.9	17.9	100.0
	Total	536	100.0	100.0	

A factor which showed a weak but statistically significant influence on respondents' opinion about sending COVID – 19 official information on social media was age. Hence, the results of

the Pearson correlation (r (534) = -.175, p=0.000), showed that as age decreases, the extent to which respondents believed that social media is an environment in which official information about the virus should be communicated decreases (Table 12). In other words, younger respondents believed to a greater extent than older respondents that official information should also be communicated on social media. One possible explanation for this results would be that young people gather most of their information from online sources, and they also engage more with social media platforms, and thus it is possible that they would also like to see official and important information on such platforms.

Table 12. Person correlation between the extent to which social media represents an appropriate environment for sharing official COVID – 19 info and age

		C1. The extent to which social media represents an appropriate environment for sharing official COVID – 19 info	D2. Age
C1. The extent to which social media represents an appropriate environment for sharing official COVID – 19 info	Pearson Correlation	1	-.175**
	Sig. (2-tailed)		.000
	N	536	536
D2. Age	Pearson Correlation	-.175**	1
	Sig. (2-tailed)	.000	
	N	536	536

**. Correlation is significant at the 0.01 level (2-tailed).

Furthermore, when dividing the study participants in medical staff (doctors, nurses) and students (medical students or students at the university nursing programs), we found that students (M=4.31, SD=2.11) believed to a greater extent than the medical staff (M= 3.88, SD=2.07) that official information about the virus should also be sent on social media (t (534) = -

2.36, p< 0.05) (Table 13). Next, when dividing the sample by living environment, participants living in the urban area (M=4.19, SD=2.10) were inclined more than those living in the rural area (M=3.72, SD=2.05), to believe that official information could also be sent on social media (t (534) = 2.23, p< 0.05) (Table 13).

Table 13. Significant t tests for sharing official information on social media professional degree and living environment

	t-test for Equality of Means										
	Group	N	Mean	S. D.	t	df	p	Mean Difference	Std. Error Difference	CI4 Lower	CI4 Upper
Official information on social media _ Professional degree[1]	Medical staff	294	3.88	2.07	-2.36	534	.01	-.42	.18	-.78	-.07
	Student	242	4.31	2.11							
Official information on social media _living environment	Urban area	411	4.19	2.10	2.23	534	.02	.47	.21	.05	.89
	Rural area	125	3.71	2.05							

[1]Index variable from the professional degrees of respondents. Student: medical student and student at university nursing program, Medical Staff: Senior specialist medical – doctor, Specialist medical – doctor, Resident, Nurse with higher education diploma, Nurse with other studies than higher education

(5) What aspects of the professional activity of the medical staff were affected most by the COVID – 19 pandemic?

The findings of our research showed that most respondents stated that the patient – doctor relationship was most affected by the pandemic (38.4%). However, a smaller percent of respondents declared that the working schedule was the most affected (26.9%), or the collaboration with their peers (23.9%) (Table 14).

Table 14. The aspect of professional life which was most influenced by the pandemic

	Frequency	Percent	Valid Percent	Cumulative Percent

Valid	patient – doctor relationship	206	38.4	38.4	38.4
	work schedule	144	26.9	26.9	65.3
	collaboration with peers	128	23.9	23.9	89.2
	other	58	10.8	10.8	100.0
	Total	536	100.0	100.0	

Furthermore, taking into account the group of medical staff (doctors, nurses) and the group of students (medical students and students at university nursing program), the results revealed that the most respondents who stated that the patient- doctor relationship was affected most by the pandemic was the group of medical staff (144 compared to 62) (Table 15). One possible explanation for this result is that, by being in constant contact with their patients, doctors and nurses were more inclined to perceive that the relation with their patients has deteriorated during the pandemic.

Table 15. Main aspect of professional life influenced by the pandemic * professional degree - Cross tabulation

		Professional degree[1]		Total
		Medical staff	Student	
A3. Main aspect of professional life influenced by the pandemic	patient – doctor relationship	144	62	206
	work schedule	70	74	144
	collaboration with peers	62	66	128
	other	18	40	58
Total		294	242	536

[1]Index variable from the professional degrees of respondents. Student: medical student and student at university nursing program, Medical Staff: Senior specialist medical – doctor, Specialist medical – doctor, Resident, Nurse with higher education diploma, Nurse with other studies than higher education

Discussion

During the COVID – 19 pandemic, one of the major issues people had to face, was the spread of misinformation about the virus, its origins and its treatment. In this regard, we analyzed the perception of medical staff (including doctors, nurses, medical students and students in the university nursing program) about the way medical and non – medical information was communicated during the pandemic. In the context of the so called infodemic [11], and the effects of misinformation on people's trust in doctors, most participants of our study declared that the information about alternative treatments for the virus affected the credibility of health professionals. Hence, from this point of view, our study is in line with previous studies which highlighted the fact that lately, trust in physician decreased [67], and which suggested that social media managed to determine people to trust the personal opinions of other people rather than the opinion of the professionals [61]. Furthermore, since other researchers pointed out that many medical practitioners used social media to express professional opinions that were later found inaccurate [74], and thus they may have contributed to the spread of misinformation [75], we argue that the credibility of physicians might have also been affected by this type of behavior.

An interesting result of our research showed that as the age of medical staff decreases, the extent to which they believe that information about alternative treatments affects doctors' credibility increases. Hence, younger healthcare professionals believed to a greater extent than older healthcare professionals, that information about alternative treatments affected negatively people's trust in doctors. This results might have as possible explanation, the fact that younger people tend to spend more time on social media platforms, and they may have

interacted more than older professionals, with misinformation about the virus, this making them more able to be aware of the negative effects of fake news. Moreover, the type of unit in which the respondents worked, was a factor which influenced the opinion of the respondents, our findings showing that, the medical staff who did not work in unit with COVID -19 patients, believed to a greater extent than those who worked in such units, that information about alternative treatments negatively influenced doctors' credibility. Given this result we argue that is it possible for those professionals who did not interact with COVID -19 patients, and who thus were more distant from the situation, to have a more distorted image regarding the way people's levels of trust in them changed in the context of the pandemic.

Considering the role of social media in spreading misinformation, our study is in line with previous studies which support the idea that such channels favored the communication of fake news during the pandemic [49, 50, 51]. In this regard, regardless of age, professional degree or living environment, most healthcare professionals who participated in our study were of the opinion that social media contributed to the spread of misinformation. However, our study also showed that when it comes to communicating official information on social media, younger respondents (students) believed to a greater extent than older respondents (doctors, nurses), that such channels should be used to send official information about the virus. Taking into account these results, the fact that healthcare professionals acknowledge that social media favors the spread of misinformation, and that many of them still believe they should be used in order to communicate official information, shows that at personal level, professionals were not affected that much by misinformation, them being able to differentiate more easily between real and fake news. In other words, we argue that while people in general were negatively

influenced by the fake news they read on social media, as it was shown in previous studies which highlighted that people trusted the information on social networks, they shared un-validated information and had trouble with differentiating real from fake news [57, 79] or that exposure to health misinformation may influence people's intention to engage in certain behaviors [80], healthcare professionals may be less influenced by fake news, due to their knowledge.

Considering the knowledge of medical staff about the type of drugs that had positive effects on treating the virus, the findings of the research showed that the respondents had opinions which were in line with the results found in other studies. Hence, according to the research, most respondents stated that the drug which was known to have positive effects against the virus was Dexamethasone (46.6%), it being followed by Remdesivir (40.5%). Thus, positive effects of Dexamethasone were also highlighted by studies [31, 32], while study [35] showed positive effects of Remdesivir.

In the context of medical staff's knowledge about alternative treatments, most respondents declared they had heard about the fact that alcohol can prevent the infection, that warm water drunk every 15 minutes, and the hot air from the hairdryer pointed to the nostrils can help eliminate the virus. From this point of view, our study is in line with a previous study [53], which also described these methods.

When it comes to the respondents' level of satisfaction about the way medical and non – medical information was communicated during the pandemic, generally, the research revealed that most respondents were dissatisfied with the communication process. In the case of communication strategies adopted by authorities, the results showed that most respondents

were satisfied with them. However, in the context of sending information about the drugs used to treat the disease, the research showed that younger healthcare professionals were more satisfied with the communication process than older healthcare professionals. This results might be due to the fact that physicians with more experience have higher expectations from authorities than students.

Another area on which we focused our research was the professional activity of the medical staff during the pandemic. In this regard, our findings revealed that, according to the respondents of our study, the aspect that was mostly affected by the pandemic was the doctor-patient relationship. Hence, our research is in line with other studies [78], which showed that the pandemic affected the way doctors interacted with their patients.

Furthermore, on the basis of the results of our study we argue that not only the process of vaccination created ethical issues, but also the process of communication [81]. Thus, these ethical issues were perceived by the medical staff and they would require a further examination in order to be able to create communication guides which can be regarded as essential instruments not only for the research process of the medical staff and healthcare professionals with management positions, but also for their current medical activity [82,83].

Conclusions

During the pandemic, healthcare professionals did not have to deal only with challenges regarding their health and the health of their patients, but also with the problems created by the spread of medical misinformation. In this regard, besides fighting the pandemic, physician also had to fight the so called infodemic. Fake news spread on social media about various alternative treatments for the virus and the opinions of certain professionals about treatment

methods which later proven to be inaccurate negatively influenced the credibility of doctors. Hence, according to the results of our research, generally, the medical staff (doctors, nurses, medical students, students at university nursing program), believed that information about alternative treatments affected people's trust in doctors, but younger healthcare professionals and those working in units without COVID - 19 patients believed to a greater extent than older healthcare professionals and people working in units with COVID – 19 patients that fake news about treatments for the virus affected the credibility of doctors.

Furthermore, regardless of age, age, gender, living environment, professional degree or working unit, the medical staff acknowledged the role of social media in spreading fake news, but when it comes to using social media in order to communicate official information, younger healthcare professionals were more inclined to believe that such networks were appropriate for the communication of official information. This results can suggest that while professionals were aware of the role of social media in spreading medical misinformation and in affecting trust in doctors, due to their knowledge, at personal level they were less affected by that type of information, many of them believing that social media should also be used for sending official information.

In the context of the drugs used to treat the virus, the results pointed out that the medical staff had knowledge about the drugs known to have positive effects in treating the virus, their perception being in line with previous studies which focused on this matter. Moreover, the medical staff was aware of the alternative treatments which were promoted on social media, the method of drinking alcohol in order to prevent the infection being the method that most of the respondents have heard about.

When it comes to the influence of the pandemic on the professional activity of the medical staff, the respondents declared that the aspect which was most affected was the doctor – patient relationship. In this regard, we argue that, by influencing peoples' trust in doctors, the medical fake news spread during the pandemic, implicitly had a role in deteriorating the relation between doctors and their patients.

Therefore, the healthcare professionals were generally dissatisfied with the way medical and non – medical information was communicated during the pandemic, but younger professionals were satisfied than older professionals. Overall, the medical staff believed that fake news managed to undermine doctors' credibility that social media platforms favor the spread of such news, and they had knowledge about the drugs which were known to have positive effects on the virus and about the alternative treatments.

Taking into account the results of the research, the paper has some theoretical and practical implications. From a theoretical point of view, the paper contributes to the literature on the matter of fake news and its influence on the trust of healthcare professionals, a strength of the paper being the fact that it analyzed the opinions of medical staff (doctors, nurses, medical students and students at university nursing program). From a practical point of view, the paper brings awareness to the phenomenon of fake news regarding medical treatments and the negative influence it has on doctors' credibility. Another practical implication refers to the fact that the paper brings attention to the issue of using social media as a mean to communicate official information, many healthcare professionals, especially the younger ones, stating that such networks could be appropriate for sharing official information. Furthermore, by highlighting that the most affected aspect of the professional activity of doctors was the

relationship with their patients, the study also shows that actions need to be taken in order to restore people's trust in doctors and improve the process of communication between them. Hence, on the basis of the findings and implications of the study, we further discuss limitations and future research directions.

Limitations and future research directions

While our study proved relevant information regarding the perception of healthcare professionals about the way medical and non – medical information was communicated in time of the pandemic, it also has some limitations.

One limitation is represented by the fact that the perception of healthcare professionals was studied only by using quantitative methods. In this regard, a future research should focus on obtaining information from doctors while using qualitative methods too. Next, the study was conducted only on Romanian healthcare professionals, and thus, a future research should take into consideration a comparison between the opinions of professionals from different countries. Another limitation is represented by the fact that we only asked respondents to state the aspect which was most influenced by the pandemic, but we did not asked them to offer detail about other type of challenges encountered. Thus, a future research should focus on analyzing the extent to which aspects of the professional activity of doctors were affected, and on analyzing more deeply the challenges they had to face during the pandemic.

Furthermore, since our research revealed that many respondents believed that social media platforms could be appropriate for sharing official information, we draw attention to a problem that can arise in this context. Since people know that such platforms favor the spread

of fake news, if we encourage the use of social media in order to communicate official information, don't we risk to discredit that information as it is possible for people to consider that such information is fake too? We believe that this issue should be taken into account and studied in a future research.

Author Contributions

Conceptualization: Claudiu Coman, Maria Cristina Bularca

Data curation: Claudiu Coman, Liliana Rogozea, Angela Repanovici, Maria Cristina Bularca

Formal analysis: Maria Cristina Bularca, Claudiu Coman

Investigation: Claudiu Coman, Maria Cristina Bularca, Angela Repanovici, Liliana Rogozea

Methodology: Maria Cristina Bularca, Claudiu Coman

Project administration: Claudiu Coman, Liliana Rogozea, Angela Repanovici

Resources: Maria Cristina Bularca, Liliana Rogozea, Angela Repanovici

Supervision: Claudiu Coman, Liliana Rogozea, Angela Repanovici

Writing – original draft: Maria Cristina Bularca, Claudiu Coman

Writing – review & editing: Maria Cristina Bularca, Claudiu Coman, Liliana Rogozea, Angela Repanovici

References

1. Sanders JM, Monogue ML, Jodlowski TZ, Cutrell J. B.Pharmacologic Treatments for Coronavirus Disease 2019 (COVID-19): A Review. *JAMA.* 2020; 323(18):1824–1836. doi:10.1001/jama.2020.6019

2. Shereen MA, Khan S, Kazmi A, Bashir N, Siddique R. COVID-19 infection: Origin, transmission, and characteristics of human coronaviruses. *J Adv Res*. 2020; 24:91-98 doi: 10.1016/j.jare.2020.03.005

3. Kristina SA, Herliana N, Hanifah S. The perception of role and responsibilities during covid-19 pandemic: A survey from Indonesian pharmacists. *Int J Pharm Res* , 2020; *12*(2). doi: 10.31838/ijpr/2020.SP2.369

4. World Health Organization (WHO). Timeline of WHO's response to COVID-19. [Internet]. World Health Organization. 2020 June 29 [cited 2020 Nov 27] Available from: https://www.who.int/news/item/29-06-2020-covidtimeline

5. European Centre for Disease Prevention and Control. COVID-19 situation update worldwide, as of 27 November 2020 [Internet]. European Centre for Disease Prevention and Control. 2020 June 29 [cited 2021 Nov 25] Available from: https://www.ecdc.europa.eu/en/geographical-distribution-2019-ncov-cases.

6. British Broadcasting Corporation. COVID vaccine: First 'milestone' vaccine offers 90% protection. [Internet]. 2020 Nov 9 [cited 2020 Nov 27] Available from: https://www.bbc.com/news/health-54873105.

7. Kommenda N, Jones FH. COVID vaccine tracker: when will a coronavirus vaccine be ready? [Internet]. The Guardian. 2020 Nov 10 [cited 2020 Nov 27] Available from:

https://www.theguardian.com/world/ng-interactive/2020/nov/10/covid-vaccine-tracker-when-will-a-coronavirus-vaccine-be-ready

8. Ahsan W, Javed S, Al Bratty M, Alhazmi HA, Najmi A. Treatment of SARS-CoV-2: How far have we reached? *Drug Discov Ther*. 2020; 14(2):67-72. doi: 10.5582/ddt.2020.03008

9. Kupferschmidt K, Cohen J. Race to find COVID-19 treatments accelerates. *Science*. 2020; 367(6485): 1412-1413. doi: 10.1126/science.367.6485.1412

10. World Health Organization (WHO). Solidarity clinical trial for COVID-19 treatments [Internet]. World Health Organization 2019. [cited 2020 Nov 27] Available from: https://www.who.int/emergencies/diseases/novel-coronavirus-2019/global-research-on-novel-coronavirus-2019-ncov/solidarity-clinical-trial-for-covid-19-treatments

11. Zarocostas J. How to fight an infodemic. *The Lancet*. 2020; *395*(10225):676. doi: 10.1016/S0140-6736(20)30461-X

12. Tuccori M, Convertino I, Ferraro S, Cappello E, Valdiserra G, Focosi D et al. The Impact of the COVID-19 "Infodemic" on Drug-Utilization Behaviors: Implications for Pharmacovigilance. *Drug Saf*. 2020;43:699–709 doi:10.1007/s40264-020-00965-w

13. Rosa SGV, Santos WC. Clinical trials on drug repositioning for COVID-19 treatment. *Rev Panam Salud Publica*. 2020; 44: e40 doi: 10.26633/RPSP.2020.40

14. Sahraei Z, Shabani M, Shokouhi S, Saffaei A. Aminoquinolines against coronavirus disease 2019 (COVID-19): chloroquine or hydroxychloroquine. *Int J Antimicrob Agents*. 2020; *105945*(10.1016) doi:10.1016/j.ijantimicag.2020.105945

15. White NJ, Watson JA, Hoglund RM, Chan XHS, Cheah PY, Tarning J. COVID-19 prevention and treatment: A critical analysis of chloroquine and hydroxychloroquine clinical pharmacology. PLoS Med. 2020; 17(9): e1003252. doi:10.1371/journal.pmed.1003252

16. Liu J, Cao R, Xu M, Wang X, Zhang H, Hu H. et al. Hydroxychloroquine, a less toxic derivative of chloroquine, is effective in inhibiting SARS-CoV-2 infection in vitro. Cell Discov. 2020; 6(16) doi:10.1038/s41421-020-0156-0 .

17. Wang M, Cao R, Zhang L, Yang X, Liu J, Xu M. et.al. Remdesivir and chloroquine effectively inhibit the recently emerged novel coronavirus (2019-nCoV) in vitro. *Cell Res*. 2020; 30:269–271. doi:10.1038/s41422-020-0282-0

18. Davidescu EI, Odajiu I, Bunea T, Sandu G, Stratan L, Aramă V. et.al. Treatment with hydroxychloroquine in patients with covid-19. Experience of a neurology department. *Farmacia*. 2020; *68*(4): 597-605. doi:10.31925/farmacia.2020.4.3

19. Gautret P, Lagier JC, Parola P, Meddeb L, Mailhe M, Doudier B. et.al. Hydroxychloroquine and azithromycin as a treatment of COVID-19: results of an open-label non-randomized clinical trial. *Int. J. Antimicrob. Agents*. 2020; 105949. doi:10.1016/j.ijantimicag.2020.105949.

20. Thomson K, Nachlis H. Emergency Use Authorizations During the COVID-19 Pandemic: Lessons From Hydroxychloroquine for Vaccine Authorization and Approval. *JAMA.* 2020; 324(13):1282–1283. doi:10.1001/jama.2020.16253.

21. U.S Food & Drug Administration. FDA cautions against use of hydroxychloroquine or chloroquine for COVID-19 outside of the hospital setting or a clinical trial due to risk of heart rhythm problems. [Internet]. Food and Drug Administration. 2020 Jul 1 [cited 2020 Nov 27]

Available from: https://www.fda.gov/drugs/drug-safety-and-availability/fda-cautions-against-use-hydroxychloroquine-or-chloroquine-covid-19-outside-hospital-setting-or .

22. Recovery. No clinical benefit from use of hydroxychloroquine in hospitalized patients with COVID-19. [Internet]. Recovery 2020 June 5 [cited 2020 Nov 27] Available from: https://www.recoverytrial.net/news/statement-from-the-chief-investigators-of-the-randomised-evaluation-of-covid-19-therapy-recovery-trial-on-hydroxychloroquine-5-june-2020-no-clinical-benefit-from-use-of-hydroxychloroquine-in-hospitalised-patients-with-covid-19 .

23. Naveed M, Uddin S, Abdullah KS, Ishaq SE, Ahmad T. Various Evidence-Based Hypothetical and Experimental Treatment Approaches and Their Effectiveness against COVID-19 Worldwide: A Comprehensive Literature Review. *EJMO* 2020; 4(4):265–285. doi: 10.14744/ejmo.2020.52538

24. Chu C M, Cheng VCC, Hung IFN, Wong MML, Chan KH, Chan KS, et.al. Role of lopinavir/ritonavir in the treatment of SARS: initial virological and clinical findings. *Thorax*. 2004; 59(3):252-256. doi:10.1136/thorax.2003.012658

25. Cao B, Wang Y, Wen D, Liu W, Wang J, Fan G, et al. A trial of lopinavir-ritonavir in adults hospitalized with severe covid-19. *N. Engl . J. Med.* 2020; 382(19): 1787-1799. doi:10.1056/NEJMoa2001282

26. Vosu J, Britton P, Howard-Jones A, Isaacs D, Kesson A, Khatami A, et al. Is the risk of ibuprofen or other non-steroidal anti-inflammatory drugs increased in COVID-19?. *J Paediatr Child Health*. 2020; *56*(10): 1645-1646. doi:10.1111/jpc.15159

27. Fang L, Karakiulakis G, Roth M. Are patients with hypertension and diabetes mellitus at increased risk for COVID-19 infection? *Lancet Respir Med*. 2020; 8(4):e21.8 doi:10.1016/S2213-2600(20)30116-8

28. Science alert. Updated: WHO Now Doesn't Recommend Avoiding Ibuprofen For COVID-19 Symptoms [Internet]. Science alert 2020 Mar 17 [cited 2020 Nov 27]. Available from: https://www.sciencealert.com/who-recommends-to-avoid-taking-ibuprofen-for-covid-19-symptoms

29. Esba LCA, Alqahtani RA, Thomas A, Shamas N, Alswaidan L, Mardawi G. Ibuprofen and NSAID Use in COVID-19 Infected Patients Is Not Associated with Worse Outcomes: A Prospective Cohort Study. *Infect Dis Ther*. 2020; 1-16. doi:10.1007/s40121-020-00363-w

30. Rinott E, Kozer E, Shapira Y, Bar-Haim A, Youngster I. Ibuprofen use and clinical outcomes in COVID-19 patients. *Clin Microbiol Infect*. 2020; 26(9):1259.e5-1259.e7. doi:10.1016/j.cmi.2020.06.003

31. Roberts M. Coronavirus: Dexamethasone proves first life-saving drug. [Internet]. British Broadcasting Corporation 2020 June 16 [cited 2020 Nov 27] Available from: https://www.bbc.com/news/health-53061281.

32. Horby P, Lim WS, Emberson JR, Mafham M, Bell JL, Linsell L, et al.. Dexamethasone in hospitalized patients with Covid-19-preliminary report. *N. Engl. J. Med*. 2020. doi:10.1056/nejmoa2021436

33. Lu CC, Chen MY, Lee WS, Chang YL. Potential therapeutic agents against COVID-19: What we know so far. *J Chin Med Assoc*. 2020; 83(6):534-536. doi: 10.1097/JCMA.0000000000000318

34. Wang Y, Zhang D, Du G, Du R, Zhao J, Jin Y, et al. Remdesivir in adults with severe COVID-19: a randomised, double-blind, placebo-controlled, multicentre trial. *The Lancet*. 2020; 395(10236):1569-1578. doi:10.1016/S0140-6736(20)31022-9

35. Beigel JH, Tomashek KM, Dodd LE, Mehta AK, Zingman BS, Kalil AC. et al. Remdesivir for the treatment of Covid-19. *N. Engl. J. Med*. 2020. https://www.nejm.org/doi/10.1056/NEJMoa2007764

36. U.S Food and Drug Administration. FDA Approves First Treatment for COVID-19 [Internet]. Food and Drug Administration 2020 Oct 22 [cited 2020 Nov 27] Available from: https://www.fda.gov/news-events/press-announcements/fda-approves-first-treatment-covid-19.

37. U.S Food and Drug Administration. Coronavirus (COVID-19) Update: November 20, 2020 [Internet]. Food and Drug Administration 2020 Nov 20 [cited 2020 Nov 27] Available from: https://www.fda.gov/news-events/press-announcements/coronavirus-covid-19-update-november-20-2020.

38. Glasdam S, Stjernswärd S. Information about the COVID-19 pandemic–a thematic analysis of different ways of perceiving true and untrue information. *SSHOP.* 2020; (2)1: 100090. https://doi.org/10.1016/j.ssaho.2020.100090

39. Caleb TC, Hayes RA. Social Media: Defining, Developing, and Divining. *Atl J Commun.*2015; 23:1: 46-65. https://doi.org/10.1080/15456870.2015.972282

40. Boyd DM, Ellison NB.Social network sites: Definition, history, and scholarship. *J Comput Mediat Commun.* 2007; *13*(1): 210-230. https://doi.org/10.1111/j.1083-6101.2007.00393.x

41. Reuter C, Stieglitz S, Imran M.Social media in conflicts and crisesBehav. *Inf. Technol*. 2020; *39*(3): 241-251. https://doi.org/10.1080/0144929X.2019.1629025

42. Vasconcellos-Silva PR, Castiel LD. COVID-19, fake news, and the sleep of communicative reason producing monsters: the narrative of risks and the risks of narratives. *Cad Saude Publica*. 2020); *36*(7): e00101920. https://doi.org/10.1590/0102-311x00101920

43. Pulido CM, Ruiz-Eugeni L, Redondo-Sama G, Villarejo-Carballido B. A New Application of Social Impact in Social Media for Overcoming Fake News in Health. *Int. J. Environ. Res. Public Health*. 2020; *17*(7):2430. **https://doi.org/10.3390/ijerph17072430**

44. Al-Dmour H, Salman A, Abuhashesh M, Al-Dmour R.Influence of social media platforms on public health protection against the COVID-19 pandemic via the mediating effects of public health awareness and behavioral changes: integrated model. *J. Medical Internet Res*. 2020; *22*(8):e19996.

45. Wong JEL, Leo YS, Tan CC. COVID-19 in Singapore—Current Experience: Critical Global Issues That Require Attention and Action. *JAMA.* 2020; 323(13):1243–1244. doi:10.1001/jama.2020.2467

46. Lazer DM, Baum MA, Benkler Y, Berinsky AJ, Greenhill KM, Menczer F, et al. The science of fake news. *Science*. 2018; *359*(6380):1094-1096. DOI: 10.1126/science.aao2998

47. Moscadelli A, Albora G, Biamonte MA, Giorgetti D, Innocenzio M, Paoli S, et al. Fake News and Covid-19 in Italy: Results of a Quantitative Observational Study. *Int. J. Environ. Res. Public Health.* 2020; *17*:5850

48. Mian A, Khan S. Coronavirus: The spread of misinformation. *BMC Medicine*. 2020; 18(1):1-2 doi:10.1186/s12916-020-01556-3 \

49. Pan American Health Organization. Understanding the infodemic and misinformation in the fight against covid-19. [Internet]. Pan American Health Organization 2020 May 1 [cited 2020 Nov 27] Available from: https://www.paho.org/en/documents/understanding-infodemic-and-misinformation-fight-against-covid-19.

50. Bowles J, Larreguy H, Liu, S. Countering misinformation via WhatsApp: Preliminary evidence from the COVID-19 pandemic in Zimbabwe. *PloS one*. 2020; *15*(10): e0240005. https://doi.org/10.1371/journal.pone.0240005

51. Ittefaq M, Hussain SA, Fatima M. COVID-19 and social-politics of medical misinformation on social media in Pakistan. *Media Asia*. 2020; *47*(1-2): 75-80. https://doi.org/10.1080/01296612.2020.1817264

52. The National Law Review. There's a Fake News Pandemic. Could COVID-19 and Trademarks be the Cure? [Internet]. The National Law Review 2020 Jul 7 [cited 2020 Nov 27] Available from: https://www.natlawreview.com/article/there-s-fake-news-pandemic-could-covid-19-and-trademarks-be-cure

53. O'connor C, Murphy M. Going Viral: Doctors Must Combat Fake News in the Fight against Covid-19. *Ir Med J*. 2020; 113(5): 85-85.

54. Pew Research Center. Nearly three-in-ten Americans believe COVID-19 was made in a lab. [Internet]. Pew research center 2020 April 8 [cited 2020 Nov 27] Available from: https://www.pewresearch.org/fact-tank/2020/04/08/nearly-three-in-ten-americans-believe-covid-19-was-made-in-a-lab/

55. Health Analytics Asia. 50 Fake ‘frequently forwarded’ COVID-19 WhatsApp messages. [Internet]. Health Analytics Asia 2020 April 2 [cited 2020 Nov 27] Available from: https://www.ha-asia.com/50-fake-frequently-forwarded-covid-19-whatsapp-messages/

56. Brennen JS, Simon F, Howard PN, Nielsen RK. Types, sources, and claims of COVID-19 misinformation. *Reuters Institute*. 2020; *7*: 1-13.

57. Pennycook G, McPhetres J, Zhang Y, Lu JG, Rand DG. Fighting COVID-19 misinformation on social media: Experimental evidence for a scalable accuracy-nudge intervention. *Psychol*. 2020; *31*(7): 770-780. https://doi.org/10.1177/0956797620939054

58. Barua Z, Barua S, Aktar S, Kabir N, Li M. Effects of misinformation on COVID-19 individual responses and recommendations for resilience of disastrous consequences of misinformation. *Prog Disaster Science*. 2020; *8*: 100119. doi:10.1016/j.pdisas.2020.100119

59. Ahmed N, Shahbaz T, Shamim A, Khan KS, Hussain SM, Usman A. The COVID-19 Infodemic: A Quantitative Analysis Through Facebook. *Cureus.* 2020; 12(11): e11346. doi: 10.7759/cureus.11346

60. Nagler RH, Vogel RI, Gollust SE, Rothman AJ, Fowler EF, Yzer MC. Public perceptions of conflicting information surrounding COVID-19: Results from a nationally representative survey of US adults. *PloS one*. 2020; *15*(10): e0240776. doi:10.1371/journal.pone.0240776

61. Orso D, Federici N, Copetti R, Vetrugno L, Bove T. Infodemic and the spread of fake news in the COVID-19-era. *Eur J Emerg Med*. 2020; 27(5):327-328. doi:10.1097/MEJ.0000000000000713

62. McNab C. What social media offers to health professionals and citizens. *Bull World Health Organ*. 2009; 87(8):566. doi:10.2471/blt.09.066712

63. Ma X, Vervoort D, Luc JG. When misinformation goes viral: access to evidence-based information in the COVID-19 pandemic. *J. Glob. Health. Sci*. 2020; *2*(1):e13 doi:10.35500/jghs.2020.2.e13

64. Tasnim S, Hossain MM, Mazumder H. Impact of rumors and misinformation on COVID-19 in social media. *J Prev Med Public Health*. 2020; *53*(3):171-174.

65. Bode L, Vraga EK.See something, say something: Correction of global health misinformation on social media. J Health Commu. 2018; *33*(9):1131-1140. doi:10.1080/10410236.2017.1331312

66. Chou WYS, Oh A, Klein WM. Addressing health-related misinformation on social media. *Jama*. 2018; *320*(23): 2417-2418. doi:10.1001/jama.2018.16865.

67. Stasiuk K, Polak M, Dolinski D, Maciuszek J. The credibility of health information sources as predictors of attitudes toward vaccination—the results from a longitudinal study in Poland. *Vaccines*. 2021; *9*(8):933 doi: 10.3390/vaccines9080933

68. Cernicova-Buca M, Palea A. An appraisal of communication practices demonstrated by romanian district public health authorities at the outbreak of the COVID-19 pandemic. *Sustainability*. 2021; *13*(5): 1-19, doi: 10.3390/su13052500

69. Tagliacozzo S, Albrecht F, Ganapati NE. International Perspectives on COVID-19 Communication Ecologies: Public Health Agencies' Online Communication in Italy, Sweden, and the United States. *Am Behav Sci*. 2021; *65*(7), 934-955 doi: 10.1177/0002764221992832

70. Saechang O, Yu J, Li Y. Public trust and policy compliance during the COVID-19 pandemic: The role of professional trust. *Healthcare*. 2021; 9 (2):1-13 doi: 10.3390/healthcare9020151.

71. Lewandowski R, Goncharuk AG, Cirella GT. Restoring patient trust in healthcare: medical information impact case study in Poland. *BMC Health Serv. Res.* 2021; *21*(1):1-11 doi: 10.1186/s12913-021-06879-2

72. Soveri A, Karlsson LC, Antfolk J, Lindfelt M, Lewandowsky S. Unwillingness to engage in behaviors that protect against COVID-19: the role of conspiracy beliefs, trust, and endorsement of complementary and alternative medicine. *BMC Public Health*. 2021; *21*(1): 1-12 doi: 10.1186/s12889-021-10643-w

73. Antinyan A, Bassetti T, Corazzini L, Pavesi F. Trust in the health system and COVID-19 treatment. Front. Psychol.. 2021; *12*:1-14 doi:10.3389/fpsyg.2021.643758

74. Law RW, Kanagasingam S, Choong KA. Sensationalist social media usage by doctors and dentists during Covid-19. *Digit. Health*. 2021; *7*:1-12 doi: 10.1177/20552076211028034

75. Leonard MB, Pursley DM, Robinson LA, Abman SH, Davis JM. The importance of trustworthiness: lessons from the COVID-19 pandemic. Pediatr. Res. 2021; 1-4 doi:10.1038/s41390-021-01866-z

76. Wood JL, Lee GY, Stinnett SS, Southwell BG. A Pilot Study of Medical Misinformation Perceptions and Training Among Practitioners in North Carolina (USA). *INQUIRY: The Journal of Health Care Organization, Provision, and Financing*, 2021; *58*:1-6 doi: 10.1177/00469580211035742

77. Gopichandran V, Sakthivel K. Doctor-patient communication and trust in doctors during COVID 19 times—A cross sectional study in Chennai, India. *Plos One*. 2021: *16*(6), 1-11 doi: 10.1371/journal.pone.0253497

78. Nwoga HO, Ajuba, MO, Ezeoke UE. Effect of COVID-19 on doctor-patient relationship. *Int J Community Med Public Health*. 2020; *7(12)*: 2394-6040, doi: 10.18203/2394-6040.ijcmph20205136

79. Rocha YM, de Moura GA, Desidério GA, de Oliveira CH, Lourenço FD, de Figueiredo NLD. The impact of fake news on social media and its influence on health during the COVID-19 pandemic: A systematic review. *J. Public Health*. 2021; 1-10 doi:10.1007/s10389-021-01658-z

80. Greene CM, Murphy G. Quantifying the effects of fake news on behavior: Evidence from a study of COVID-19 misinformation. *J Exp Psychol Appl*. 2021 Dec;27(4):773-784. doi: 10.1037/xap0000371.

81.Rogozea LM, Sechel G, Bularca MC, Coman C, Cocuz ME. Who's Getting Shots First? Dealing With the Ethical Responsibility for Prioritizing Population Groups in Vaccination. *Am J Ther*. 2021 Jun 22;28(4):e478-e487. doi: 10.1097/MJT.0000000000001400. PMID: 34228653.

82. Rogozea L, Purcaru D, Leaşu F, Nemet C. Biomedical research - opportunities and ethical challenges. *Rom J Morphol Embryol.* 2014;55(2 Suppl):719-22. PMID: 25178352.

83. Olimid AP, Rogozea LM, Olimid DA. Ethical approach to the genetic, biometric and health data protection and processing in the new EU General Data Protection Regulation (2018). *Rom J Morphol Embryol*. 2018;59(2):631-636. PMID: 30173275.

Supporting information

S1 Appendix English version of the questionnaire

(docx)

S2 Appendix Romanian version of the questionnaire

(docx)
