# Supplementary material for: Misinformation about medication during the COVID– 19 pandemic: A perspective of medical staff
Source: PLoS One. 2022 Oct 27;17(10):e0276693. doi: 10.1371/journal.pone.0276693 (PMC9612566; doi:10.1371/journal.pone.0276693)
Supplement: Attachment — Submitted filename: PONE-D-22-09134_R2 reviewed.pdf [file pone.0276693.s009.pdf]

PLOS ONE

Challenges in the communication process during the COVID-19 pandemic- a perspective of medical staff

--Manuscript Draft--

**Manuscript Number:**	PONE-D-22-09134R2
**Article Type:**	Research Article
**Full Title:**	Challenges in the communication process during the COVID-19 pandemic- a perspective of medical staff
**Short Title:**	Challenges in the communication process during the COVID-19 pandemic
**Corresponding Author:**	Claudiu Coman<br>Universitatea Transilvania din Brasov<br>Brasov, ROMANIA
**Keywords:**	Miscommunication; healtcare professionals; trust; Covid - 19 pandemic
**Abstract:**	Background Healthcare professionals had to face numerous challenges during the pandemic, their professional activity being influenced not only by the virus, but also by the spread of medical misinformation. In this regard, we aimed to analyze, from the perspective of medical staff, the way medical and non - medical information about the virus was communicated during the pandemic in order to raise awareness about the way misinformation affected the medical staff. Methods and findings. The study was conducted on Romanian healthcare professionals including doctors, nurses and medical students. They were asked to answer to a questionnaire and the sample of the research includes 536 respondents. The findings revealed that most respondents stated that information about alternative treatments against the virus affected the credibility of health professionals, and that younger professionals believed to a greater extent that trust in doctors was affected. The research also showed that respondents were well informed about the drugs used in clinical trials in order to treat the virus, and that younger respondents believed that social media should be used to send official information. Among the main limitations of our study we mention the fact that we used only quantitative methods and the fact we focused only on Romanian healthcare professionals. Conclusions Healthcare professionals declared that the spread of misinformation regarding alternative treatments, affected their credibility and the relationship with their patients. Healthcare professionals had knowledge about the drugs used in clinical trials, and they acknowledged the role of social media in spreading medical misinformation. However, younger professionals also believed that social media could be used to share official information about the virus. A future research should focus on studying the opinion of Romanian and international doctors, it should use qualitative methods too and should address the issue of social media being an appropriate environment for sending official information.
**Order of Authors:**	Claudiu Coman
	Maria Cristina Bularca
	Angela Repanovici
	Liliana Rogozea
**Response to Reviewers:**	*For a more proper view of our Response to reviewers, we kindly ask you to check the Word document entitled Response to reviewers.<br><br>Dear Sir/Madam<br>With this cover letter we submit the revised manuscript, initially entitled” Challenges in the communication process during the COVID-19 pandemic- a perspective of medical staff”, and after complying with the suggestions of the reviewers, entitled “Misinformation about medication during the COVID – 19 pandemic: a perspective of medical staff” by Claudiu Coman, Maria Cristina Bularca, Angela Repanovici and Liliana Rogozea for publication in PLOS ONE.<br>We revised the manuscript according to the suggestions and recommendation made by the reviewers. We would like to thank the reviewers for taking time to review our paper and for providing such useful suggestions. We also thank the academic editor

Powered by Editorial Manager® and ProduXion Manager® from Aries Systems Corporation

for reviewing our paper. We tried to comply with all the suggestions and recommendations made by the reviewers, and in this letter, we describe the changes we made to the text according to the recommendations of the reviewers.
Our manuscript needed major revisions. The changes were made while having active the “Track changes” function from Microsoft Word and the lines where the text was changed can be best viewed while having active the “All markup” option. Moreover, in order for our changes to be best seen, we will also provide in this cover letter, the lines from the revised manuscript with the “Track changes” function, and “All markup” option active. With regards to our response to Reviewer 1, the reviewer made a series of suggestions directly in the PDF version of our initial manuscript, but also provided a summary of those suggestions in the e-mail which was sent by the journal to the corresponding author. In this regard, we responded first to the comments highlighted in the summary from the e-mail, and then we responded to each point made by Reviewer 1 in the PDF version of our initial manuscript. Next, we responded to each point raised by Reviewer 2.
Our response to Reviewer 1:
We firstly thank the reviewer for taking time to review our manuscript and provide suggestions in order to improve it. We addressed all the suggestions made by the reviewer. When we describe how the text was changed, we also provide the lines where the text can be found in the revised manuscript with the option “Track changes” active. In this way, the changes can be viewed completely (the text we deleted, and the text we inserted). Next, we will firstly describe our answers to the comments which were summarized in the e-mail received by the corresponding author, and then we will present our responses to the comments made by the reviewer in the PDF version of our manuscript.

Reviewer 1 comments- as summarized in the email received by the corresponding author
Reviewer 1 point 1: the review comments attached. The required modifications can be summarized as following and the authors will find it in details in the attached file: the authors should review the journal guidelines and abide by it in manuscript preparation.
Response 1: We are grateful to the reviewer for the suggestion. We reviewed the guidelines of PLOS ONE journal again and we made sure our manuscript is prepared in accordance to the author guidelines which can be found on the journal’s official website. We also checked the pdf files entitled “Download sample title, author list, and affiliation page” and “Download sample manuscript body”, in order to make sure our manuscript is correctly formatted. Thus, we looked again at the guidelines for the sections which have to be included in the manuscript, the font and sizes for headings, table captions, referencing rules, etc., and we made sure our manuscript respects the guidelines of the journal.
Reviewer 1 point 2: the introduction section is too long and need to be summarized.
Response 2: We thank the reviewer for the useful suggestion. In order to comply with it, we tried to summarize our introduction. Thus, we would like to mention that we also took into account the comments the reviewer made in the pdf version of the manuscript. In this regard, there the reviewer recommended us to rephrase the first paragraph of our paper because the paragraph was not about the communication process: “the introductory paragraph is not related to communication process”. We rephrased the paragraph and we added information in which we highlighted the fact that the COVID – 19 pandemic negatively influenced the communication process. The changes we made, the text deleted, added or rephrased can be best seen while having active the “Track changes” function and the “All markup” option provided by Microsoft Word. Thus, in the Introduction section of the paragraph, page 4 of the manuscript, lines 71-77, we made changes to the text, and the new introductory paragraph also addresses the subject of communication:
“The COVID 19 pandemic generated multiple changes in the way today’s society members carry out their daily activities. One of the processes which was mostly affected by the pandemic was the communication process between institutions and the public, as well as between individuals. In this regard, from this perspective, while many domains were affected by the spread of the virus, such as the educational system or the cultural sector, the health sector was the one that faced the most challenges [1].”
Next, in the pdf version of our manuscript, the reviewer suggested that the details we gave regarding the virus could be summarized in one paragraph: “the history of covid-19 can be summarized in a single paragraph”. In order to comply with the request, in

Powered by Editorial Manager® and ProduXion Manager® from Aries Systems Corporation

the Introduction section, at page 4 of the manuscript, we summarized the text indicated by the reviewer.
The text the reviewer suggested us to summarize:
"Caused by severe acute respiratory syndrome coronavirus 2 (SARS-CoV-2) [2], the disease was firstly detected in December 2019, in Wuhan, China [3], and it fastly spread all over the world. The World Health Organization was informed about a pneumonia outbreak in Wuhan on December 31 2019, the number of cases continued to increase, and on March 11 2020 the World Health Organization characterized COVID 19 as a pandemic [4]. Being highly contagious, the virus affected a large number of people, and as of November 27 over 61 million cases were reported [5]. Even though many companies and institutions are struggling to develop a vaccine, Pfizer, Gamaleya Research Institute, University of Oxford, and a preliminary analysis of the vaccine proposed by Pfizer showed that the vaccine is able to prevent more than 90% of people from getting infected with COVID 19 [6], so far no vaccine was approved as a general and universal vaccine against COVID 19 [7]. Ever since the pandemic was declared, finding the right treatment for the virus has become a priority for researchers and doctors from all over the world. In this regard, large number of trials started to be conducted, and in order to find an efficient drug treatment against the virus, one method that was adopted was testing and administrating to patients, drugs that were previously used for curing other viruses [8]. Thus, on March 20 2020, The World Health Organization launched the SOLIDARITY clinical trial, a trial that monitored the effects on patients infected with COVID 19, of specific drugs that proven to be effective in the treatment of other diseases: remdesivir, interferon beta, chloroquine and hydroxychloroquine -previously used for Malaria, as well as drugs used on HIV patients: lopinavir and ritonavir [9]. However, according to the interim results published on October 15 2020 by WHO, even though those drugs were taught to have positive effects on treating COVID 19, they had little influence or no influence at all on mortality in general, on the need and initiation of ventilation and on the recovery process [10]."
The way we summarized the text can be seen at lines 102-114- in the revised version of our manuscript (The full change, the text deleted and the text summarizes is visible at lines 78-114).
The text we summarized (lines 102 -114 with the "Track changes" and "All Markup" option active:
"Caused by severe acute respiratory syndrome coronavirus 2 [2], the disease was firstly detected in December 2019, in Wuhan, China [3]. Due to the evolution of the virus, the World Health Organization declared the pandemic in March 2020 [4], and as of November 27 over 61 million cases were reported [5]. In this regard, although several companies are struggling to develop a vaccine, and some of the proposed vaccines showed promising results [6], so far no vaccine was approved in order to be administrated to the entire population [7]. Ever since the pandemic was declared, many companies started to be preoccupied with finding a treatment, and one method used that was adopted was administrating to patients, drugs that were previously used for curing other viruses [8]. Thus, one of the most well - known trials started was the SOLIDARITY trial, which focused on using various drugs including chloroquine and hydroxychloroquine, lopinavir or ritonavir [9]. However, even if those drugs were taught to have positive effects on treating the virus, they did not have a significant influence on preventing mortality in general [10]".
Next, in order to reduce the information written in the Introduction section, as the reviewer suggested, we also deleted the last paragraph of the Introduction section, paragraph in which we provided details about the concepts that we addressed next in the Literature review section. Thus, at lines 118 – 122 in the revised manuscript with "Track changes" and "All Markup" option active, we deleted the following text:
"Hence, considering the purpose of our paper and the research questions, we believed it was necessary to analyze the literature on the drugs used to treat COVID – 19, on the role of social media platforms in spreading fake information about the virus and potential treatments, and on the way the pandemic influenced the credibility of doctors and their relationship with their patients."

Reviewer 1 point 3: the section titles need to be reviewed and fixed.
Response 3: We thank the reviewer for the useful suggestion. We checked again the author guidelines provided by the journal on its official website, regarding sections of the manuscript. In this regard, we corrected the section which was entitled "Methods and materials" in the initial version of our manuscript, with the correct form, which is

Powered by Editorial Manager® and ProduXion Manager® from Aries Systems Corporation

"Materials and methods". The change can be seen in the revised manuscript at page 17, line 364, while having active the "Track changes" and "All markup" options from Microsoft Word. We reviewed all of our section titles and made sure they are correct.
Reviewer 1 point 4: the resuklts section include too much tables need to be focusing on the most significant tables and attach the other tablesas supplementary tables.
Response 4: We are grateful to the reviewer for such useful suggestion. We addressed the suggestion, we looked at the tables included in the Results section and we integrated in the section only the most significant tables. The other tables were deleted from the text and added to supplementary information. Thus, we created Word documents with supplementary information for each of our research questions. In this regard in S3_Tables with results to the 1st research question we included Table 2 ; in S4_Tables with results to the 2nd research question we included Table 5 and Table 6; in S5_Tables with results to the 3rd research question we included Table 7 and Table 9; in S6_Tables with results to the 4th research question we included Table 11 and Table 12; in S7_Tables with results to the 5th research question we included Table 15 and Table 16.
Reviewer 1 point 5: the methods section is missing the research design, sampling method and the calculation of the study sample and the validity and reliability section.
Response 5: We are very grateful to the reviewer for suggesting us to improve the methods section of our paper. With regards to the research design section, we added this section to our manuscript and we explained in detail the research design. Even more, we deleted some information from the Sampling and data collection procedures and we added it to the research design section because it was more suitable there. In this regard, at pages 17-18 of the manuscript, between lines 365- 385 can be found the Research design section of our paper, which comprises the following text:
"The present study was conducted on Romanian healthcare professionals including doctors, nurses and medical students. The method used is quantitative. The questionnaire was administrated online, the data was collected through the help of Google forms, and was disseminated on groups of healthcare professionals and students on platforms such as Facebook and WhatsApp, during the period April 2021–June 2021. The data we collected was firstly exported to Microsoft Excel, and then it was analyzed with IBM Statistical Package for the Social Sciences, version 20. The respondents were informed about the purpose of the study, about the fact that they were allowed to withdraw at any time, and they were asked to give their consent for participating in the study. The average time needed to complete the questionnaire was 15 minutes. Considering the validity of our research, we took into account the theoretical information from the literature regarding the development of a questionnaire. Our team of researchers together with health specialists have configured the dimensions, and operationalized the concepts in accordance with the theoretical approaches identified at the current stage of the research. Even more, we pre-tested the questionnaire before disseminating in order to guarantee the validity of the instrument. Thus, the questionnaire was completed by 50 respondents in the pre-testing stage. Considering the reliability of the research, we used split half reliability method. We split our sample in half, and we checked the variables in from our sub-samples in order to see if the variables provided convergent results. The convergent results we obtained by applying the split half method showed that we obtained a high fidelity measurement.
In order to create the research design section and to also improve the way our paper is structured, we made changes to the section "Sampling and data collection procedures". In this regard, we deleted some text and we reformulated some phrases. The section comprises the following text, which can be found at pages 17-18 of the revised manuscript with "Track changes" and "All markup" option active, lines 419-427:
"In order to conduct the research we used a quantitative method while having as an instrument a questionnaire. The responses were collected online, with the help of Google forms, and the questionnaire was self – administrated. The research received approval from The Council of the Faculty of Sociology and Communication, approval request Nr.378/30.03.2021. Taking into account the sampling method and the calculation of the study sample, we used random, probabilistic sampling method. We took into consideration specialists, physicians, and medical students from Brasov, and we applied the snowballing method in order to disseminate the questionnaire. The sample of our study comprises 536 respondents, and included doctors, nurses as well as medical students from Romania."
With regards to the sampling method, we would like to thank the reviewer for pointing out that we should give more information about the sampling procedure. Even though

in the initial version of our manuscript we described the sample of our research, how the questionnaire was distributed and to whom, we added more specific information about the sampling method. Hence, at page 20 of the manuscript, lines 423 - 426, we explained that we used a random, probabilistic sampling method:
"Taking into account the sampling method and the calculation of the study sample, we used random, probabilistic sampling method. We took into consideration specialists, physicians and medical students from Brasov, and we applied the snowballing method in order to disseminate the questionnaire."
Reviewer 1 point 6: the conclusion section need to be summarized and conclude the main study findings and its significance.
Response 6: We are grateful to the reviewer for the suggestion. In order to comply with it we tried to summarize our Conclusions section, to highlight again the main findings of the research and the significance of our study. In this regard, the text which was written in Conclusions in the initial version of our manuscript was improved. In this regard, we deleted some of the redundant information which was written in this section. The information we deleted:
"In this regard, besides fighting the pandemic, physician also had to fight the so called infodemic. Fake news spread on social media about various alternative treatments for the virus and the opinions of certain professionals about treatment methods which later proven to be inaccurate negatively influenced the credibility of doctors." (Lines 789-792)
"This results can suggest that while professionals were aware of the role of social media in spreading medical misinformation and in affecting trust in doctors, due to their knowledge, at personal level they were less affected by that type of information, many of them believing that social media should also be used for sending official information" (lines 803-807)
"Moreover, the medical staff was aware of the alternative treatments which were promoted on social media, the method of drinking alcohol in order to prevent the infection being the method that most of the respondents have heard about" (lines 811-813).
"Hence, on the basis of the findings and implications of the study, we further discuss limitations and future research directions." (Lines 838-839).
Next, we took into account the recommendation of the reviewer and we started the section by presenting the main findings of our research. Since we had several research questions, we presented our main findings in relation to those research questions.
Next, the reviewer recommended us to explain the significance of our study. Thus, in the paper we had already written the theoretical and practical implication of our paper. In this regard, we did not delete the implications because we consider that the implications emphasize why the study conducted is important and how it can be further taken into consideration. Next, we did not delete the limitations and future research directions either, because we considered necessary to highlight how and why our study has limitations but also how it could be further developed or extended.
Reviewer 1 point 7: the references are too much need to be filtered and summarized to 30 or 40 refrences maximum. Regards
Response 7: We are very grateful to the reviewer for this recommendation and we appreciated the interest in improving our paper. However, when we started to write the article, we wanted to make sure our paper will be well documented and that it will address all the theoretical concepts and aspects needed. In this regard, we made a thorough research and literature review on the medication used in order to treat the virus, on the way social media contributed to the spread of misinformation about the virus, and on the way trust in doctors and the doctor- patient relation was affected during the pandemic. Thus, we read many research paper because we wanted for our paper to provide an overall view on the subject addressed. In this regard, we consider that all the references we used are relevant for the subject approached and for the research that we conducted, and therefore we could not delete more than half of them. In other words, through the references cited we support and sustain our arguments, we show how other researchers approached similar matters and thus we could not delete more than half of our references because we considered that by deleting them we could no longer have a strong and well consolidated theoretical background and we could not properly explain how we wanted to address the matted of medical misinformation and its effects from the perspective of medical staff. Even more, the journal does not have a limitation regarding the length of the article or the number of references: "Manuscripts can be any length. There are no restrictions on word count, number of figures, or amount of supporting information". In addition, we have seen

articles which addressed subjects related to health and the COVID – 19 pandemic, and which were published in PLOS ONE, that have more than 40 references. For example, one article entitled “Severity of infection with the SARS- CoV -2 B1.1.7 lineage among hospitalized COVID – 19 patients in Belgium” (https://journals.plos.org/plosone/article?id=10.1371/journal.pone.0269138), has 76 references, and another article, entitled “The coronavirus disease 2019 (COVID -19) vaccination psychological antecedent assessment using the ARABIC 5c validated tool: An online survey in 13 Arab countries” (https://journals.plos.org/plosone/article?id=10.1371/journal.pone.0260321) has 71 references.

Reviewer 1 comments- as pointed by the reviewer in the PDF version of our manuscript

Reviewer 1 point 1: A perspective of medical staff

Response 1: We thank the reviewer for the suggestion. We put “:” instead of “-“in our title, before the phrase “a perspective of medical staff”. The change can be seen at line 2 of the revised manuscript.

Reviewer 1 point 2: the abstract need to be summarized to 250 to 300 words by the main important information in each part ....it is recommended to avoid long paragraphs and to paraphraze and summarize the ideas in short paragraphs.

Response 2: We are grateful to the reviewer for the recommendation. In order to comply with it we summarized our abstract to 219 words. In this regard, we deleted the text which was written in the Abstract section, and instead, at page 3 of the revised manuscript with “Track changes” and “All markup” option on, at lines 50 –68 we inserted the following text:

“Background. Healthcare professionals had to face numerous challenges during the pandemic, their professional activity being influenced not only by the virus, but also by the spread of medical misinformation. In this regard, we aimed to analyze, from the perspective of medical staff, the way medical and non - medical information about the virus was communicated during the pandemic in order to raise awareness about the way misinformation affected the medical staff.

Methods and findings. The study was conducted on Romanian healthcare professionals. They were asked to answer to a questionnaire and the sample of the research includes 536 respondents. The findings revealed that most respondents stated that information about alternative treatments against the virus affected the credibility of health professionals, and that younger professionals believed to a greater extent that trust in doctors was affected. The research also showed that respondents were well informed about the drugs used in clinical trials in order to treat the virus.

Conclusions. Healthcare professionals declared that the spread of misinformation regarding alternative treatments, affected their credibility and the relationship with their patients. Healthcare professionals had knowledge about the drugs used in clinical trials, and they acknowledged the role of social media in spreading medical misinformation. However, younger professionals also believed that social media could be used to share official information about the virus.”

Reviewer 1 point 3: the introductory paragraph is not related to communication process.

Response 3: We thank the reviewer for pointing this out. We explained how we addressed this point above in this Cover letter, in point 2 raised by the reviewer in the summary which was written in the e-mail sent to the corresponding author. However, we will present again the way we changed the introductory paragraph in order for it to be related to communication process. In this regards, in the Introduction section of the paragraph, page 4 of the manuscript with “Track changes” and “All markup active”, lines 71-77, we made changes to the text, and the new introductory paragraph also addresses the subject of communication:

“The COVID 19 pandemic generated multiple changes in the way today’s society members carry out their daily activities. One of the processes which was mostly affected by the pandemic was the communication process between institutions and the public, as well as between individuals. In this regard, from this perspective, while many domains were affected by the spread of the virus, such as the educational system or the cultural sector, the health sector was the one that faced the most challenges [1].”

Reviewer 1 point 4: the history of covid-19 can be summarized in a single paragraph.

Response 4: We are very grateful to the reviewer for the recommendation. We tried to comply with it and we summarized the history of COVID -19. Earlier in this cover letter we explained how we addressed this point because the reviewer also mentioned it in

the summary which was written in the e-mail sent to the corresponding author. In this regard, we summarized the indicated text, and at page 5 of the manuscript with "Track changes" and "All markup" option active, lines 102- 114 we added the following text: "Caused by severe acute respiratory syndrome coronavirus 2 [2], the disease was firstly detected in December 2019, in Wuhan, China [3]. Due to the evolution of the virus, the World Health Organization declared the pandemic in March 2020 [4], and as of November 27 over 61 million cases were reported [5]. In this regard, although several companies are struggling to develop a vaccine, and some of the proposed vaccines showed promising results [6], so far no vaccine was approved in order to be administrated to the entire population [7]. Ever since the pandemic was declared, many companies started to be preoccupied with finding a treatment, and one method used that was adopted was administrating to patients, drugs that were previously used for curing other viruses [8]. Thus, one of the most well - known trials started was the SOLIDARITY trial, which focused on using various drugs including chloroquine and hydroxychloroquine, lopinavir or ritonavir [9]. However, even if those drugs were taught to have positive effects on treating the virus, they did not have a significant influence on preventing mortality in general [10]".

Reviewer 1 point 5: the stydy aim is to assess the perception and this other aim is not included as an intervention, so it is better to rephrased as to recommend future researches or interventions to raise......

Response 5: We thank the reviewer for the useful suggestion. We tried our best in addressing the recommendation. In this regard, we rephrased the part of the purpose indicated by the reviewer. In other words, the reviewer suggested us to rephrase the last part of our purpose, to rephrase the expression "in order to raise awareness about the way misinformation affected medical staff". Hence, at page 6 of the manuscript with "Track changes" and "All Markup" option active, lines 129 –133 we rephrased the purpose and added the following text:

"The purpose of the paper is to analyze, from the perspective of medical staff, the way medical and non - medical information about the virus was communicated during the pandemic to encourage the development of future research or interventions in order to raise awareness about the way misinformation affected medical staff."

Due to the suggestion of the reviewer, we had to change the way we described the purpose of our paper in other sections of our manuscript too. Thus, the purpose of the paper was changed in the way recommended by the reviewer, also at lines: 52 -55 (in the Abstract section).

Reviewer 1 point 6: please to consider the restructuring of the manuscript as per the journal guidelines and the title of each section. Also, the literature review section is very long and it should be fixed to bo not more than 2 to 2 and half pages summarizing the main ideas.

Response 6: We are very grateful to the reviewer for suggesting us to check again the guidelines of the journal. As we previously explained in this Cover letter, (due to the fact that the same point was also highlighted by the reviewer in the summary which was written in the e-mail sent to the corresponding author), we checked again the guidelines and made sure our manuscript is formatted according to the guidelines. We also checked again the titles of the section which should be included in the manuscript, and at page 17 of the revised manuscript with "Track changes" and "All markup" option active, line 364 we changed "Methods and materials" to "Materials and methods".

With regards to summarizing our Literature review and deleting references from our paper, we present again the explanation we gave earlier in the Cover letter, at point 7 made by the reviewer in the e-mail sent to the corresponding author:

We are very grateful to the reviewer for this recommendation and we appreciated the interest in improving our paper. However, when we started to write the article, we wanted to make sure our paper will be well documented and that it will address all the theoretical concepts and aspects needed. In this regard, we made a thorough research and literature review on the medication used in order to treat the virus, on the way social media contributed to the spread of misinformation about the virus, and on the way trust in doctors and the doctor- patient relation was affected during the pandemic. Thus, we read many research paper because we wanted for our paper to provide an overall view on the subject addressed. In this regard, we consider that all the references we used are relevant for the subject approached and for the research that we conducted, and therefore we could not delete more than half of them. In other words, through the references cited we support and sustain our arguments, we show how other researchers approached similar matters and thus we could not delete more

Powered by Editorial Manager® and ProduXion Manager® from Aries Systems Corporation

than half of our references because we considered that by deleting them we could no longer have a strong and well consolidated theoretical background and we could not properly explain how we wanted to address the matted of medical misinformation and its effects from the perspective of medical staff. Even more, the journal does not have a limitation regarding the length of the article or the number of references: “Manuscripts can be any length. There are no restrictions on word count, number of figures, or amount of supporting information”. In addition, we have seen articles which addressed subjects related to health and the COVID – 19 pandemic, and which were published in PLOS ONE, that have more than 40 references. For example, one article entitled “Severity of infection with the SARS- CoV -2 B1.1.7 lineage among hospitalized COVID – 19 patients in Belgium” (https://journals.plos.org/plosone/article?id=10.1371/journal.pone.0269138), has 76 references, and another article, entitled “The coronavirus disease 2019 (COVID -19) vaccination psychological antecedent assessment using the ARABIC 5c validated tool: An online survey in 13 Arab countries” (https://journals.plos.org/plosone/article?id=10.1371/journal.pone.0260321) has 71 references.

Reviewer 1 point 7: Research Design (please to review examples of the journal manuscript preparation)

Response 7: We thank the reviewer for pointing out that we should described more thoroughly the Research design of our paper. We explained how we addressed this suggestion earlier in this Cover letter, because the reviewer highlighted the suggestion in the summary from the e-mail sent to the corresponding author too. However, we will present again the way we complied with the suggestion. We did review examples of the journal manuscript preparation, and after we had done so, we deleted some text from the section “Sampling and data collection procedures” and moved it to the new section created. In this regard, at pages 17-18 of the revised manuscript with “Track changes” and “All markup” option active, lines 365-385, we inserted a sub-section entitled “Research design” which comprises the following text:

“The present study was conducted on Romanian healthcare professionals including doctors, nurses and medical students. The method used is quantitative. The questionnaire was administrated online, the data was collected through the help of Google forms, and was disseminated on groups of healthcare professionals and students on platforms such as Facebook and WhatsApp, during the period April 2021–June 2021. The data we collected was firstly exported to Microsoft Excel, and then it was analyzed with IBM Statistical Package for the Social Sciences, version 20. The respondents were informed about the purpose of the study, about the fact that they were allowed to withdraw at any time, and they were asked to give their consent for participating in the study. The average time needed to complete the questionnaire was 15 minutes. Considering the validity of our research, we took into account the theoretical information from the literature regarding the development of a questionnaire. Our team of researchers together with health specialists have configured the dimensions, and operationalized the concepts in accordance with the theoretical approaches identified at the current stage of the research. Even more, we pre-tested the questionnaire before disseminating in order to guarantee the validity of the instrument. Thus, the questionnaire was completed by 50 respondents in the pre-testing stage. Considering the reliability of the research, we used split half reliability method. We split our sample in half, and we checked the variables in from our sub-samples in order to see if the variables provided similar results. The convergent results we obtained by applying the split half method showed that we obtained a high fidelity measurement.”

Reviewer 1 point 8: methods and data (please to review the journal authors guideline).Also the reserch design is missed, please to clarify the research design used.

Response 8: We thank the reviewer for the suggestion. We reviewed again the journal author guidelines. Also, we added a research design section and the text contained in the section can be found at lines 365-385 of the manuscript with the “Track changes” and “All markup” option active.

Reviewer 1 point 9: start new sentence (line 333) in the PDF version of our manuscript

Response 9: We thank the reviewer for the recommendation. We complied with it and we started a new sentence, at page 17 of the manuscript with “Track changes” and “All markup” option active, lines 372 we deleted the words “At the beginning of the questionnaire”, and we started a new sentence with “The respondents were

Powered by Editorial Manager® and ProduXion Manager® from Aries Systems Corporation

informed…”.
Reviewer 1 point 10: Also this section should not include the data interpretation or analysis. it should include only description.
Response 10: The reviewer referred to the “Sample and data collection procedure” section. We are grateful to the reviewer for the suggestion and in order to comply with it we made some changes to the text which was written in this section. In this regard, the data interpretation and analysis was removed from the section, and was moved to the “Results” section of our paper. The deleted text together with the table can be seen at lines 427 –444 of the revised manuscript with “Track changes” and “All markup” option active. The text we inserted in the “Results” section can be seen at lines 486-501 of the manuscript:
“Out of the 536 respondents, 460 (85.8%) were female and 76 (14.2%) were male. A total of 411 respondents live in the urban area (76.7%), while 125 (23.3%) live in the rural area. Most respondents (286, 53.4%) are between 18 and 35 years of age, 142 respondents (26.5%) are between 36 and 50 years of age, 102 respondents (19.0%) are between 51 and 65 years of age, and 6 of them (1.1) are over 65 years of age. When it comes to the professional degree of the respondents, most of them are students at a university nursing program (122, 22.8%), and medical students (120, 22.4%). However, a total of 102 respondents (19.0%) are senior specialists medical – doctors, and 70 (13.1%) are nurses who have a higher education diploma. When it comes to the respondents field of specialization, most of them (70.5%) operate in the field of general medicine, while others are family doctors (10.4%), pediatricians (3%), dentists or oncologists (1.9%), surgeons of doctors who are specialized in internal medicine (1.5%), or infectious disease doctors, radiologists or cardiologists (1.1%). Furthermore, most of the respondents (77.2%) stated that they did not work a unit with COVID – 19 patients while few of them (22.8%) stated that they worked in such a unit at the time the research was conducted. Thus, all the characteristics of the sample are presented in Table 1.
Table 1. Sample characteristics (n = 536).
Category CountPercentage
GenderFemale46088.8%
Male7614.2%
Living environmentUrban 41176.7%
Rural 12523.3%
Age18-35 years old28653.4%
36-50 years old14226.5%
51 -65 years old10219.0%
Over 65 years old61.1%
Professional degreeSenior specialist medical - doctor10219.0%
Specialist medical - doctor468.6%
Resident285.2%
Nurse with higher education diploma7013.1%
Nurse with other studies than higher education489.0%
Medical student12022.4%
Student at university nursing program
12222.8%
Field of specialization General medicine37870.5%
Family doctor5610.4%
Pediatrics163%
Stomatology 101.9%
Oncology101.9%
Surgery81.5%
Internal medicine81.5%
Virology/ infectious disease doctor61.1%
Cardiology61.1%
Radiology61.1%
Other326%
Works in a unit with COVID – 19 patientsYes 12222.8%
No 41477.2%

”
Reviewer 1 point 11: please to explain how you calculated the sample size and the type of sampling that you used.
Response 11: We thank the reviewer for the suggestion. We offered an explanation for

this point, which was also mentioned by the reviewer in the summary provided in the e-mail sent to the corresponding author. However, we will present again the explanation, which can be found at lines 413-416 of the manuscript with “Track changes” and “All markup” option active:
“Taking into account the sampling method and the calculation of the study sample, we used random, probabilistic sampling method. We took into consideration specialists, physicians and medical students from Brasov, and we applied the snowballing method in order to disseminate the questionnaire.”
Reviewer 1 point 12: this section should be trasfered before data presentation and analysis with the methods part before data analysis
Response 12: The reviewer was referring to “The research instrument” section. We thank the reviewer for the suggestion. Since the section was already written before the “Data analysis” section, we moved the section before “Sampling and data collection procedures”. The deleted text can be seen at lines 446-465 in the revised the manuscript with “Track changes” and “All markup” option active. The section was moved and so, the following text can be found in the revised manuscript at lines 387-406:
“In order to conduct the research we used a quantitative method while having a questionnaire as an instrument. In this regard, we developed a questionnaire which comprises four sections: A. Influence of the pandemic on the professional activity of medical staff (items A1 to A4), B. Perception about the authorities’ communication process (items B1 to B11), C. Perception about the communication of non- validated treatments (items C1 to C20), and D. Sociodemographic questions (items D1 – D9), such as: gender, age, living environment, professional degree, field of specialization. The sociodemographic questions were used in order to identify different or similar attitudes between specific groups. The questionnaire can be found in “S1.Appendix English version of the questionnaire”, and in “S2. Appendix Romanian version of the questionnaire.” Before disseminating the questionnaire, the instrument was tested on 30 doctors who work in the field of cardiology and general medicine. The respondents understood clearly the questions and did not report any issue in the process of answering them. Hence, the questionnaire comprises close ended and open ended questions (Items A1, A4, B3, B11, C19, C20, D2, D5, D6,) dihotomic questions as well as questions whose answers were measured on a 7 point Likert scale. For example, item A2 measured the extent to which the respondents considered that the pandemic influenced the way they carried out their professional activity (1- “to an extremely little extent, 7 “to an extremely great extent”), or item B2 measure the respondents’ level of agreement with statements regarding the way authorities communicated during the pandemic (1 – “strongly disagree, 7-“strongly agree”).”

Reviewer 1 point 13: the validity and reliabity section is missed , please to discuss it clearly Response 13: We thank the reviewer for the recommendation. In order to address the recommendation, we inserted into our manuscript information about the validity and reliability of our research in the “Research design” section. In this regard, at page 18 of the manuscript with “Track changes” and “All markup” option active, lines 376 – 385, we inserted the following explanation:
“Considering the validity of our research, we took into account the theoretical information from the literature regarding the development of a questionnaire. Our team of researchers together with health specialists have configured the dimensions, and operationalized the concepts in accordance with the theoretical approaches identified at the current stage of the research. Even more, we pre-tested the questionnaire before disseminating in order to guarantee the validity of the instrument. Thus, the questionnaire was completed by 50 respondents in the pre-testing stage.
Considering the reliability of the research, we used split half reliability method. We split our sample in half, and we checked the variables in from our sub-samples in order to see if the variables provided similar results. The convergent results we obtained by applying the split half method showed that we obtained a high fidelity measurement.”
Reviewer 1 point 14: you have two tables number by number 1 two times. please to review the tables numbering and indexing in the maneuscript.
Response 14: We are very grateful to the reviewer for pointing this out. We checked again all the numbers of the tables and corrected all the mistakes. Now in the revised manuscript, all the tables are correctly numbered.
Reviewer 1 point 15: these codes need to be interpretted (to give its full interpretaion under each table)
Response 15: We thank the reviewer for the suggestion. The reviewer was referring to

the numbers of the questions which appear in the tables with correlations and t tests. Those numbers represent the number of the questions from the questionnaires which were included in the t tests or in the correlations. In other words, the numbers refer to the variables used in order to make the tests and the correlations. For example, in Table 3, C14 means, the question 14 from the questionnaire, which belongs to section C. Section C refers to Perception about the communication of non- validated treatments. So, under each table from our manuscript (including the tables which we put in supplementary information) we added an explanation of the codes (numbers).
We would like to mention that the numbers of our tables changed, because in the initial manuscript we had two tables numbered 1, so now we corrected the mistake. Thus, we further present the explanation we gave in the revised manuscript with "Track changes" and "All markup" option active, under each table:
Table 3 (which was table 2 in the initial manuscript). The following explanation was added under the table: "1 1 C14 – refers to the question 14 from the section C of the manuscript (The extent to which information about alternative treatments affected trust in physicians), section which refers to Perception about the communication of non-validated treatments; 2D2 - refers to question 2 from the D section of the manuscript (age), which refers to Sociodemographic characteristics of the respondents
Table 8 (which was Table 7 in the initial manuscript). The following explanation was added under the table "1 B10- refers to the question 10 from the section B of the manuscript (Satisfaction with the way information about drugs used to treat the virus was communicated) section which refers to Perception about the authorities' communication process; 2D2 - refers to question 2 from the D section of the manuscript (age), which refers to Sociodemographic characteristics of the respondents."
Table 13 (which was Table 12 in the initial manuscript). The following explanation was added under the table "1 C1 – refers to question 1 from the section C of the manuscript (The extent to which social media represents an appropriate environment for sharing official COVID – 19 info), section which refers to Perception about the communication of non- validated treatments; 2D2 - refers to question 2 from the D section of the manuscript (age), which refers to Sociodemographic characteristics of the respondents.
Table 16 (which was Table 15 in the initial manuscript and which is in Supplementary information - S7 Tables with results to the 5th research question). The following explanation was added under the table "2A3 – refers to question 3 from the section A of the manuscript (Main aspect of professional life influenced by the pandemic), section which refers to Influence of the pandemic on the professional activity of medical staff; The explanation for 1 professional degree was already written under the table in the initial version of our manuscript.
Reviewer 1 point 16: the variables need to be clear on the table
Response 16: We thank the reviewer for pointing this out. The reviewer was referring to the variables from the table which had the number 3 in the initial version of our manuscript. The table now has the number 4, because we corrected the way we numbered the tables. Hence, in order to be clear which the variables in the table are, we put the word "variables" in front of the variables which were tested. The changes to the table can be seen in the revised version of our manuscript with "Track changes" and "All markup" option active at page 29:
"Table 4. Significant t-test results: comparisons between variables
t-test for Equality of Means
GroupNMean S. D.tdfp Mean DifferenceStd. Error DifferenceCI4
LowerUpper
Variables: Information about alternative treatments _ Professional degree1Medical staff2945.331.54-2.04534.04-.27.13-.52-.01
Student2425.601.49
Variables: Information about alternative treatments _working unitUnit with COVID -19 patients1225.191.61-2.13534.03-.33.15-.64-.02
Unit without COVID 19 patients4145.531.49
Variables: Information about alternative treatments _genderMale765.101.70-2.16534.03-.40.18-.77-.03
Female4605.511.48
1Index variable from the professional degrees of respondents. Student: medical student and student at university nursing program, Medical Staff: Senior specialist medical – doctor, Specialist medical – doctor, Resident, Nurse with higher education diploma, Nurse with other studies than higher education"

Powered by Editorial Manager® and ProduXion Manager® from Aries Systems Corporation

	Reviewer 1 point 17: there keys need to be written in full interpretaion under each table.<br>Response 17: We thank the reviewer for the recommendation. We complied with it, and as we explained at one of the previous points of the reviewer, the keys (or codes) refer to the number of the question from the questionnaire, and the letter refers to the section of the questionnaire. Hence, the reviewer referred to the table which had the number 7 in the initial version of our manuscript. The table has the number 8 in the revised version of our manuscript with “Track changes” and “All markup” option active, because we corrected the way we numbered the tables. Under table 8, at page 33 of the manuscript we added the following explanation:<br>“1 B10- refers to the question 10 from the section B of the manuscript (Satisfaction with the way information about drugs used to treat the virus was communicated) section which refers to Perception about the authorities’ communication process; 2D2 - refers to question 2 from the D section of the manuscript (age), which refers to Sociodemographic characteristics of the respondents.”<br>Reviewer 1 point 18: the tables are too much, please to focus on the highly significant tables and add the others as a supplementary tables. it is recommended to reduce the number of tables to 5 or 6 tables<br>Response 18: We are very grateful to the reviewer for the useful suggestion. We complied with the suggestion and we deleted some tables from the manuscript and added them as supplementary information. Early in this Cover letter we provided an explanation for the tables, because this point was also included in the summary provided by the reviewer in the e-mail sent to the corresponding author. We let in the manuscript only the important tables: the tables with correlations and t tests, and the table with sociodemographic ch...
**Additional Information:**	
**Question**	**Response**
**Financial Disclosure**<br><br>Enter a financial disclosure statement that describes the sources of funding for the work included in this submission. Review the submission guidelines for detailed requirements. View published research articles from *PLOS ONE* for specific examples.<br><br>This statement is required for submission and **will appear in the published article** if the submission is accepted. Please make sure it is accurate.	The authors received no specific funding for this work.

Powered by Editorial Manager® and ProduXion Manager® from Aries Systems Corporation

**Unfunded studies**<br>Enter: *The author(s) received no specific funding for this work.*<br><br>**Funded studies**<br>Enter a statement with the following details:<br>• Initials of the authors who received each award<br>• Grant numbers awarded to each author<br>• The full name of each funder<br>• URL of each funder website<br>• Did the sponsors or funders play any role in the study design, data collection and analysis, decision to publish, or preparation of the manuscript?<br>• **NO** - Include this sentence at the end of your statement: *The funders had no role in study design, data collection and analysis, decision to publish, or preparation of the manuscript.*<br>• **YES** - Specify the role(s) played.<br><br>* typeset	
**Competing Interests**<br><br>Use the instructions below to enter a competing interest statement for this submission. On behalf of all authors, disclose any competing interests that could be perceived to bias this work—acknowledging all financial support and any other relevant financial or non-financial competing interests.<br><br>This statement is required for submission and **will appear in the published article** if the submission is accepted. Please make sure it is accurate and that any funding sources listed in your Funding Information later in the submission form are also declared in your Financial Disclosure statement.<br><br>View published research articles from *PLOS ONE* for specific examples.	The authors have declared that no competing interests exist.

**NO authors have competing interests**<br><br>Enter: *The authors have declared that no competing interests exist.*<br><br>**Authors with competing interests**<br><br>Enter competing interest details beginning with this statement:<br><br>*I have read the journal's policy and the authors of this manuscript have the following competing interests: [insert competing interests here]*<br><br>* typeset	
**Ethics Statement**<br><br>Enter an ethics statement for this submission. This statement is required if the study involved:<br><br>• Human participants<br>• Human specimens or tissue<br>• Vertebrate animals or cephalopods<br>• Vertebrate embryos or tissues<br>• Field research<br><br>Write "N/A" if the submission does not require an ethics statement.<br><br>General guidance is provided below. Consult the submission guidelines for detailed instructions. **Make sure that all information entered here is included in the Methods section of the manuscript.**	APPROVAL OF THE COUNCIL OF THE FACULTY OF SOCIOLOGY AND COMMUNICATION<br>Approval request: Nr.378/30.03.2021<br>Form of consent obtained: oral

Powered by Editorial Manager® and ProduXion Manager® from Aries Systems Corporation

**Format for specific study types**<br><br>**Human Subject Research (involving human participants and/or tissue)**<br>• Give the name of the institutional review board or ethics committee that approved the study<br>• Include the approval number and/or a statement indicating approval of this research<br>• Indicate the form of consent obtained (written/oral) or the reason that consent was not obtained (e.g. the data were analyzed anonymously)<br><br>**Animal Research (involving vertebrate animals, embryos or tissues)**<br>• Provide the name of the Institutional Animal Care and Use Committee (IACUC) or other relevant ethics board that reviewed the study protocol, and indicate whether they approved this research or granted a formal waiver of ethical approval<br>• Include an approval number if one was obtained<br>• If the study involved *non-human primates*, add *additional details* about animal welfare and steps taken to ameliorate suffering<br>• If anesthesia, euthanasia, or any kind of animal sacrifice is part of the study, include briefly which substances and/or methods were applied<br><br>**Field Research**<br><br>Include the following details if this study involves the collection of plant, animal, or other materials from a natural setting:<br>• Field permit number<br>• Name of the institution or relevant body that granted permission	
**Data Availability**<br><br>Authors are required to make all data underlying the findings described fully available, without restriction, and from the time of publication. PLOS allows rare exceptions to address legal and ethical concerns. See the PLOS Data Policy and FAQ for detailed information.	Yes - all data are fully available without restriction

A Data Availability Statement describing where the data can be found is required at submission. Your answers to this question constitute the Data Availability Statement and **will be published in the article**, if accepted.<br><br>**Important:** Stating 'data available on request from the author' is not sufficient. If your data are only available upon request, select 'No' for the first question and explain your exceptional situation in the text box.<br><br>Do the authors confirm that all data underlying the findings described in their manuscript are fully available without restriction?	
**Describe where the data may be found in full sentences. If you are copying our sample text, replace any instances of XXX with the appropriate details.**<br><br>• If the data are **held or will be held in a public repository**, include URLs, accession numbers or DOIs. If this information will only be available after acceptance, indicate this by ticking the box below. For example: *All XXX files are available from the XXX database (accession number(s) XXX, XXX.*).<br>• If the data are all contained **within the manuscript and/or Supporting Information files**, enter the following: *All relevant data are within the manuscript and its Supporting Information files.*<br>• If neither of these applies but you are able to provide **details of access elsewhere**, with or without limitations, please do so. For example:<br><br>*Data cannot be shared publicly because of [XXX]. Data are available from the XXX Institutional Data Access / Ethics Committee (contact via XXX) for researchers who meet the criteria for access to confidential data.*<br><br>*The data underlying the results presented in the study are available from (include the name of the third party*	All relevant data are within the manuscript and its Supporting Information files.

and contact information or URL).<br>• This text is appropriate if the data are owned by a third party and authors do not have permission to share the data.<br><br>* typeset	
Additional data availability information:	

Powered by Editorial Manager® and ProduXion Manager® from Aries Systems Corporation

Manuscript revised Click here to access/download;Manuscript;Manuscript.revised.docx

Misinformation about medication during the COVID – 19 pandemic: a perspective of medical staff

Claudiu Coman[1#a*], Maria Cristina Bularca[1], Angela Repanovici[2], Liliana Rogozea[3]

1 Department of Social Sciences and Communication, Faculty of Sociology and Communication, Transilvania University of Brasov, Brasov, Romania;

2 Department of Product Design, Mechatronics and Environment, Faculty of Product Design and Environment, Transilvania University of Brasov, Brasov, Romania

3 Basic, Preventive and Clinical Sciences Department, Transilvania University of Brasov, Brasov, Romania;

[#a] Current address: Department of Social Sciences and Communication, Faculty of Sociology and Communication, Transilvania University of Braşov, Brasov, România

[*] Corresponding author

E-mail: claudiu.coman@unitbv.ro (CC)

Abstract

Background. Healthcare professionals had to face numerous challenges during the pandemic, their professional activity being influenced not only by the virus, but also by the spread of medical misinformation. In this regard, we aimed to analyze, from the perspective of medical staff, the way medical and non - medical information about the virus was communicated during the pandemic to encourage the development of future research or interventions in order to raise awareness about the way misinformation affected medical staff.

Methods and findings. The study was conducted on Romanian healthcare professionals. They were asked to answer to a questionnaire and the sample of the research includes 536 respondents. The findings revealed that most respondents stated that information about alternative treatments against the virus affected the credibility of health professionals, and that younger professionals believed to a greater extent that trust in doctors was affected. The research also showed that respondents were well informed about the drugs used in clinical trials in order to treat the virus.

Conclusions. Healthcare professionals declared that the spread of misinformation regarding alternative treatments, affected their credibility and the relationship with their patients. Healthcare professionals had knowledge about the drugs used in clinical trials, and they acknowledged the role of social media in spreading medical misinformation. However, younger professionals also believed that social media could be used to share official information about the virus.

Introduction

The COVID 19 pandemic generated multiple changes in the way today's society members carry out their daily activities. One of the processes which was mostly affected by the pandemic was the communication process between institutions and the public, as well as between individuals. In this regard, from this perspective, while many domains were affected by the spread of the virus, such as the educational system or the cultural sector, the health sector was the one that faced the most challenges, [1].

"Caused by severe acute respiratory syndrome coronavirus 2 [2], the disease was firstly detected in December 2019, in Wuhan, China [3]. Due to the evolution of the virus, the World Health Organization declared the pandemic in March 2020 [4], and as of November 27 over 61 million cases were reported [5]. In this regard, although several companies are struggling to develop a vaccine, and some of the proposed vaccines showed promising results [6], so far no vaccine was approved in order to be administrated to the entire population [7]. Ever since the pandemic was declared, many companies started to be preoccupied with finding a treatment, and one method used that was adopted was administrating to patients, drugs that were previously used for curing other viruses [8]. Thus, one of the most well - known trials started was the SOLIDARITY trial, which focused on using various drugs including chloroquine and hydroxychloroquine, lopinavir or ritonavir [9]. However, even if those drugs were taught to have positive effects on treating the virus, they did not have a significant influence on preventing mortality in general [10].

With the development of many trials and programs meant to find a cure for COVID 19 and with the use of diverse drug combinations, another major problem arose: misinformation

and fake news about the virus, its treatment or methods to combat it. In this regard, along with the pandemic, people also had to face an epidemic of information, described by the general director of WHO as an „infodemic” [11]. In other words, information about COVID 19 began to be spread by people on every available communication channel, both in the online and offline environment. However, very often and especially on social media, the information was poorly communicated, it was distorted and there usually wasn't enough scientific evidence to demonstrate its validity [12].

Taking into account the previously mentioned aspects the paper addresses the issues of drugs tested and used for the treatment of COVID 19 and how information about COVID 19 was communicated in the offline and online environment. The purpose of the paper is to analyze, from the perspective of medical staff, the way medical and non - medical information about the virus was communicated during the pandemic in order to encourage the development of future research or interversions in order to raise awareness about the way misinformation affected medical staff. Thus, the paper aims at finding an answer to three research questions: (1) to what extent information about alternative treatments affected the credibility of medical staff? (2) What is the knowledge of medical staff about the type of drugs that had positive effects on treating the disease and about alternative treatments? (3) How satisfied is the medical staff with the way medical and non-medical information was communicated online and offline during the pandemic? (4) What is the perception of medical staff about the role of social media in spreading misinformation about the virus? (5) What aspects of the professional activity of the medical staff were affected most by the COVID – 19 pandemic?

Literature review

Information on drugs used to treat COVID 19

Before analyzing the way information about the virus was communicated in the online environment, it is important to take a look at the drugs used to treat the disease. Hence, one of the most important issues that appeared with the COVID 19 pandemic, was finding the right treatment for the virus. In this regard, researchers started to develop many experimental trials and used diversified drug combinations in order to treat patients with COVID 19. However, information that was communicated about the effectiveness of certain drugs was often contradictory.

Chloroquine and hydroxychloroquine are two drugs that were tested and included in many trials. Both drugs were previously used to treat malaria but they also have antiviral effects on viruses like HIV since they have the ability to prevent the virus to enter in the host cells [13]. Even though they have similar compounds, chloroquine is taught to have more negative effects than hydroxychloroquine [14], and hydroxychloroquine is considered safer due to the fact that it can be tolerated better for a longer period of time [15].

While some studies show positive effects of hydroxychloroquine in inhibiting the infection with the virus in vitro [16, 17], other studies found no influence of the drug on mortality rate or time spent by patients in the hospital [18]. However, when hydroxychloroquine was combined with other drugs such as azithromycin, it showed beneficial effects in treating patients with COVID 19 [19].

Nonetheless the findings regarded the effectiveness of these drugs were contrasting. For example, on March 28 2020 the Food and Drug Administration (FDA) issued an Emergency Use Authorization for using hydroxychloroquine in treating people suffering from COVID 19

[20], and in June 15 2020, the FDA retracted the authorization stating that the trials in which the drug was involved showed that the drug had no effect on the faster recovery of patients or on decreasing chances of death [21]. Even more, on 5th June 2020 the UK trial, Randomised Evaluation of COVID 19 THERAPY (RECOVERY), also stopped testing the drug on patients because the results showed no benefits in improving the conditions of hospitalized patients with COVID 19 [22].

Studies were carried out with other drugs such as lopinavir/ritonavir, an antiviral drug used in the treatment of HIV [23]. While in concentration of 4 µg/ml and 50 µg/ml, the drug showed positive effects against the virus in vitro [24], a study on 199 patients, from which 99 received the drug and the other 100 did not receive the drug, revealed that lopinavir/ritonavir had no benefits when it comes to diminishing mortality or improving the state of patients with severe symptoms [25].

Controversial discussions also involved the use of Ibuprofen, a Non-steroidal anti-inflammatory drug that is used to treat fever, or inflammation [26]. Since the pandemic was declared there has been a preoccupation regarding ibuprofen and its role in making people more vulnerable to contacting the virus. Thus, right after the declaration of the pandemic, in a letter addressed to The Lancer Journal, researchers pointed out that ibuprofen could make people with diabetes, cardiac disease or hypertension more likely to get infected with virus and have severe symptoms [27]. However, while firstly, WHO recommended people who are infected with the virus not to take ibuprofen, only one day after that recommendation, on 18 March 2020, WHO corrected its statement and mentioned that it ”does not recommend against ibuprofen” [28]. Even more, a study focusing on the use of ibuprofen showed that the drug does not make

patients feel worse [29] and another study that analyzed the use of ibuprofen and paracetamol of 403 COVID 19 confirmed patients revealed that compared to paracetamol, ibuprofen did not aggravated the clinical state of the patients [30].

While other drugs failed to show beneficial effects on the treatment of COVID 19, drugs like dexamethasone, which is included in the UK RECOVERY trial, revealed positive effects on people suffering from COVID 19: the drug lowered the risk of death in patients on ventilators from 40% to 28% and in patients who were in need of oxygen, from 25% to 20%, but did not influence the state of patients who did not need oxygen [31, 32].

Another highly tested drug was Remdesivir, an antiviral drug produced by Gilead Sciences that was previously used in treating Ebola [33]. The information regarding its positive effects on treating COVID 19 is also contradictory. A study conducted from February 6 2020 until March 12 2020, on 237 patients, showed that the drug did not bring any benefits for people that had severe symptoms of COVID 19 [34], while a more recent study revealed that Remdesivir had a more positive effect in reducing the time of recovery in patients with COVID 19 that showed signs of respiratory issues, than it had the placebo effect [35]. However, the FDA approved on October 22 2020, the use of Remdesivir in the case of adults and also children aged 12 or older who have at least 44 kilograms, who are infected with the virus and need to be treated in the hospital [36], and as of November 20 2020, FDA allows, in emergency cases, the use of Remdesivir in combination with Baricitinib, for adults and children aged two or older that require oxygen and treatment in the hospital [37].

Social media and COVID 19 misinformation

Together with the health crisis, the COVID 19 pandemic generated an information crisis, often described as an infodemic, that is represented by the spread of fake news, misguided and false information, especially in the online environment [38].

In this context, social media plays an essential role in disseminating information. Social media consists of internet based channels that provide people with the opportunity to interact, communicate in asynchronous way and in real time, with either small or large audiences where value is derived from user generated content [39]. Social media comprises multiple social networks, which according to Boyd and Ellison, offer users the possibility to create profiles that are public, or semi-public, to create a list of people with whom they can interact and share information and to view the list of connections that other users make [40].

Social media channels are often used in time of crisis not only by citizen, but also by official authorities, emergency services, because they can facilitate communication and the spread of valuable information that can contribute to surpassing the crisis [41]. Social networks like Facebook, Whatsapp, Twitter, Instagram can function as sources that have the ability to confirm or complete the information communicated by the authorities, while also receiving feedback from the public [42]. Thus, sending messages through social media channels is a strategy that can help authorities obtain feedback on certain proposals regarding public health policies [43]. Even more, a study regarding the influence of social media on the way people protect their health during the pandemic, showed that social media can have positive impact on increasing awareness about public health and protection against the virus [44].

However, during the pandemic, while authorities can use social media to keep the public informed, a major issue generated by social media, that public health representatives have to face, is the spread of fake news [45].

Fake news are represented by fabricated information designed in the form of news communicated by the media that do not share the same process of organization and do not have the same intent, and fake news are related to misinformation: information that is false or misleading, and disinformation: a type of false information whose aim is to deceive people [46].

Thus, the internet became a favorable environment for spreading conspiracy theories or false information about alternative treatment for the virus. Since people were stressed and frightened by the uncertainty of the situation, they started to consider reasonable and valid any information that presented explanations in regards to the virus [47]. Thus, when referring to health information, false news often undermine the credibility of official sources, they create confusion among people and favor the faster spread of the virus [48].

Misinformation during the pandemic can negatively influence peoples' health because false information is not easy to recognize, because it can determine people to change their behavior in a way that is harmful to their health and those around them. Thus, since the pandemic was declared, false information has been spread about the origin of the virus, about what caused it, how it spreads and what treatment is efficient for eliminating it [49]. However, a study focusing on the WhatsApp platform showed that when the information on social media is shared by trusted sources, it can increase knowledge about the virus and encourage people to adopt preventive behavior [50].

During the time of crisis, on platforms like WhatsApp or Facebook, more and more false news and unverified information about the virus began to be shared. With millions of users worldwide, WhatsApp became one of the platforms where most fake news were shared by forwarding messages to many users [51], while Facebook was characterized as the core, epicenter of misinformation [52].

When it comes to health misinformation on social media, the most discussed subjects are alternative cures involving certain food or drinks, hygiene related actions and treatment drugs. Thus, among the most “recommended” practices for preventing or curing COVID were drinking hot water every 15 minutes in order for the virus to go into the stomach, eating garlic, taking vitamin C or even pointing a hairdryer to the nostrils because the heat could eliminate the virus [53].

False news that circulated on social media regarding the virus also involve the idea that the virus was created on purpose in a lab, three in ten Americans considering true this information [54].

However, many other unverified methods were shared and the most forwarded messages on WhatsApp presented information about the fact that if people hold their breath for ten seconds without coughing then they are not infected with the virus, about the idea that at temperatures of 30-35 Celsius degrees the virus will die, messages about the release of the vaccine or about drugs allegedly recommended by Chinese doctors that could be efficient in eliminating the virus [55].

Nonetheless, misinformation became a major issue in the context of the pandemic, but also a subject of interest for researchers. A study focusing on the spread of fake news showed

that most news reconfigure and twist the original information thus creating a different context, and that most of them contain false information about public authorities and health organizations [56].

Another study found that people who tend to rely on their intuition or who possess little scientific knowledge about certain subjects, encountered difficulties in differentiating true and false information [57]. Thus, misleading or unverified information can negatively influence the way people behave. For example, people in USA who died after they consumed chloroquine may have used the drug because news about it mentioned that it could treat and eliminate the virus [58]. Even more, a study concerning misinformation on Facebook revealed that posts made from verified accounts contained more false information than the accounts that were not verified [59], while other study conducted from 23 April 2020 to 27 April 2020, focused on perception about contradictory information and stated that 73% of participants mentioned they observed or were exposed to contrasting messages usually communicated by politicians or health experts [60].

Apart from influencing peoples' beliefs or health practices, COVID 19 fake news also influenced the activity of health professionals. Social media managed to increase the level of trust in information that comes from people's personal opinions rather than professionals [61], and doctor's credibility is often affected. In order to improve these situations, doctors must be willing to use social media not just to send messages, but to actively communicate with people, to offer feedback, to share their experiences and rectify and clarify the fake news presented on social media [62].

Among action from health professionals, in order to combat COVID 19 fake news, social media networks as well as public authorities must implement some strategies. For example, the government of United Kingdom developed collaboration programs between its rapid response teams and social media platforms, and Taiwan introduced greater fines for news that were proven to be false [63]. Moreover, even though some social networks such as Facebook or Twitter already implemented algorithms to identity and remove fake accounts [64], or to correct information [65], they should further develop efficient strategies in order to validate the information that people share [66].

The influence of the pandemic on doctors' credibility and relationship with patients

The way information regarding the virus was communicated online and offline during the pandemic played an essential role in the process of maintaining trust in health professionals. In this regard, a previous longitudinal study conducted in Poland revealed that trust in physicians has declined from 2018 – 2020, and emphasized the idea that the decrease may be caused by the health problems that people had to cope with during the pandemic and the problems with the healthcare system of the country [67]. In Romanian context, a previous study showed that the communication process of the healthcare system was poor and confusing, and that public health authorities at national level focused more on global information about the virus, while local authorities failed to succeed in providing their "share of information" [68]. Another study, which focused on analyzing the online communication of Public Health Agencies from Italy, United States and Sweden, revealed that compared to Sweden and the United States, agencies from Italy collaborated more with other organizations, and that overall, the communication

process of the agencies was coordinated by their members, that agencies also communicated with governments, but they rarely collaborated with political or non-governmental organizations [69]. Hence, while trust in the government and communication from authorized organizations is essential, the importance of trusting the professionals is highlighted by a study conducted in Thailand, which showed that in the cases in which people have low levels of trust in the government, trust in professionals can have a positive influence on the adoption of protective measures at the individual level [70].

Furthermore, another previous study conducted in Poland, revealed that information can have the power to influence the level of trust that people have in the healthcare system and in healthcare professionals, suggesting that an increase of trust in hospitals, may be associated with a decrease of trust in physicians [71].

While focusing on studying people's response to non- pharmaceutical interventions, conspiracy theories and alternative treatments, a study conducted in Finland showed that the level of trust people have in the system implemented in order to provide information about the virus, has an essential role in the way people react to the official measures recommended. Hence, most participants in the study were between 40 and 60 years of age, and the study emphasized that people who were less willing to comply with the non-pharmaceutical interventions implemented by the government, tended to believe more in conspiracies and had low levels of trust in the sources which provided information about the virus [72].

Another study, which focused on examining the relationship between trust in the healthcare system and people's choice of seeking medical help when they experienced COVID –

19 symptoms, concluded that high levels of trust in the healthcare system can increase the probability of asking for medical help when people first notice COVID – 19 symptoms [73].

Taking into account the aspects mentioned above, we can infer that peoples' trust in doctors was affected during the pandemic. In this regard, in the context of misinformation, one of the reasons why people lost trust in doctors may be the fact that, besides using social media for communicating information, for networking or for interacting with patients, many medical or dental practitioners used social media to express their professional opinions about the virus, opinions which were not validated and which later proven to be inaccurate [74]. In other words, health professionals may have contributed to the spread of misinformation, and such behavior can contribute to the decrease of trust in medical processes and in healthcare professionals [75]. Other researchers who focused on examining medical misinformation, found that most doctors (94.2%) stated that patients had medical misinformation, and the subjects about they had the most inaccurate information were represented by COVID – 19 vaccines, COVID – 19 origin, treatment or essential oils [76]. Furthermore, a previous study discovered that trust in doctors increased with age, and communication difficulties decreased, and that trust in doctors decreased while the level of education and communication difficulties increased [77].

Hence, while acknowledging that the pandemic influenced the trust in medical professionals, another aspect that was negatively influenced was the relationships between doctors and their patients. A study which focused on examining the doctor – patient interaction from the perspective of both groups of people, revealed differences in the respondents' opinions. Thus, most doctors stated that they still make eye contact (72%) and that they still

show patients empathy, but only few patients declared that their doctors made eye contact (56,8%) or showed them empathy (43,2%) [78].

Materials and methodsResearch design

The present study was conducted on Romanian healthcare professionals including doctors, nurses and medical students. The method used is quantitative. The questionnaire was administrated online, the data was collected through the help of Google forms, and was disseminated on groups of healthcare professionals and students on platforms such as Facebook and WhatsApp, during the period April 2021– June 2021. The data we collected was firstly exported to Microsoft Excel, and then it was analyzed with IBM Statistical Package for the Social Sciences, version 20. The respondents were informed about the purpose of the study, about the fact that they were allowed to withdraw at any time, and they were asked to give their consent for participating in the study. The average time needed to complete the questionnaire was 15 minutes.

Considering the validity of our research, we took into account the theoretical information from the literature regarding the development of a questionnaire. Our team of researchers together with health specialists have configured the dimensions, and operationalized the concepts in accordance with the theoretical approaches identified at the current stage of the research. Even more, we pre-tested the questionnaire before disseminating in order to guarantee the validity of the instrument. Thus, the questionnaire was completed by 50 respondents in the pre-testing stage. Considering the reliability of the research, we used split half reliability method. We split our sample in half, and we checked the variables in from our sub-samples in

order to see if the variables provided convergent results. The convergent results we obtained by applying the split half method showed that we obtained a high fidelity measurement.

The research instrument

In order to conduct the research we used a quantitative method while having a questionnaire as an instrument. In this regard, we developed a questionnaire which comprises four sections: A. Influence of the pandemic on the professional activity of medical staff (items A1 to A4), B. Perception about the authorities' communication process (items B1 to B11), C. Perception about the communication of non- validated treatments (items C1 to C20), and D. Sociodemographic questions (items D1 – D9), such as: gender, age, living environment, professional degree, field of specialization. The sociodemographic questions were used in order to identify different or similar attitudes between specific groups. The questionnaire can be found in "S1.Appendix English version of the questionnaire", and in "S2. Appendix Romanian version of the questionnaire." Before disseminating the questionnaire, the instrument was tested on 30 doctors who work in the field of cardiology and general medicine. The respondents understood clearly the questions and did not report any issue in the process of answering them. Hence, the questionnaire comprises close ended and open ended questions (Items A1, A4,B3, B11, C19, C20, D2, D5, D6,) dihotomic questions as well as questions whose answers were measured on a 7 point Likert scale. For example, item A2 measured the extent to which the respondents considered that the pandemic influenced the way they carried out their professional activity (1- "to an extremely little extent, 7 "to an extremely great extent"), or item

B2 measure the respondents' level of agreement with statements regarding the way authorities communicated during the pandemic (1 – "strongly disagree, 7-" strongly agree").

Sampling and data collection procedures

In order to conduct the research we used a quantitative method while having as an instrument a questionnaire. The responses were collected online, with the help of Google forms, and the questionnaire was self – administrated. The research received approval from The Council of the Faculty of Sociology and Communication, approval request Nr.378/30.03.2021. Taking into account the sampling method and the calculation of the study sample, we used random, probabilistic sampling method. We took into consideration specialists, physicians,and medical students from Brasov, and we applied the snowballing method in order to disseminate the questionnaire. The sample of our study comprises 536 respondents, and includeds doctors, nurses as well as medical students from Romania.

Data analysis

Data was analyzed with IBM Statistical Package for the Social Sciences, version 20. In order to analyze the data and identify differences and similarities between the attitudes of certain groups, t tests for independent samples were performed. The t test were performed among groups: male/female, working in unit with COVID – 19 patients/ not working in unit with COVID – 19 patients, urban/rural area, and professional degree: medical staff/students. Hence, in order to be able to analyze the results depending on professional degree, we computed the variable of professional degree which had the following values: senior specialist medical –

doctor, specialist medical – doctor, resident, nurse with higher education diploma, nurse with other studies than higher education, medical student, student at university nursing program, in a new variable. Thus, doctors, nurses and residents, were integrated in a new group called "medical staff", while medical students and students at university nursing programs were integrated in the group "students". Moreover, for a better understanding of the way some variables correlate with each other, (for example: respondents satisfaction with the way authorities communicated during the pandemic and age, respondents' opinion about the way misinformation about alternative treatments influenced doctors' credibility and age), we also calculated the Pearson coefficient.

Results

Out of the 536 respondents, 460 (85.8%) were female and 76 (14.2%) were male. A total of 411 respondents live in the urban area (76.7%), while 125 (23.3%) live in the rural area. Most respondents (286, 53.4%) are between 18 and 35 years of age, 142 respondents (26.5%) are between 36 and 50 years of age, 102 respondents (19.0%) are between 51 and 65 years of age, and 6 of them (1.1) are over 65 years of age. When it comes to the professional degree of the respondents, most of them are students at a university nursing program (122, 22.8%), and medical students (120, 22.4%). However, a total of 102 respondents (19.0%) are senior specialists medical – doctors, and 70 (13.1%) are nurses who have a higher education diploma. When it comes to the respondents field of specialization, most of them (70.5%) operate in the field of general medicine, while others are family doctors (10.4%), pediatricians (3%), dentists or oncologists (1.9%), surgeons of doctors who are specialized in internal medicine (1.5%), or

infectious disease doctors, radiologists or cardiologists (1.1%). Furthermore, most of the respondents (77.2%) stated that they did not work a unit with COVID – 19 patients while few of them (22.8%) stated that they worked in such a unit at the time the research was conducted. Thus, all the characteristics of the sample are presented in Table 1.

Table 1. Sample characteristics (n = 536).

	Category	Count	Percentage
Gender	Female	460	88.8%
	Male	76	14.2%
Living environment	Urban	411	76.7%
	Rural	125	23.3%
Age	18-35 years old	286	53.4%
	36-50 years old	142	26.5%
	51 -65 years old	102	19.0%
	Over 65 years old	6	1.1%
Professional degree	Senior specialist medical - doctor	102	19.0%
	Specialist medical - doctor	46	8.6%
	Resident	28	5.2%
	Nurse with higher education diploma	70	13.1%
	Nurse with other studies than higher education	48	9.0%
	Medical student	120	22.4%
	Student at university nursing program	122	22.8%
Field of specialization	General medicine	378	70.5%
	Family doctor	56	10.4%
	Pediatrics	16	3%
	Stomatology	10	1.9%
	Oncology	10	1.9%
	Surgery	8	1.5%
	Internal medicine	8	1.5%
	Virology/ infectious disease	6	1.1%

	doctor		
	Cardiology	6	1.1%
	Radiology	6	1.1%
	Other	32	6%
Works in a unit with COVID – 19 patients	Yes	122	22.8%
	No	414	77.2%

1) To what extent information about alternative treatments affected the credibility of medical staff?

The results of our research revealed that respondents were of the opinion that information about alternative treatments for COVID -19 affected the credibility of healthcare professionals. Hence, most respondents (32.5%), stated that trust in healthcare professionals was affected to a an extremely great extent by the information about alternative treatments, many of them declared that credibility was affected to a very great extent (23.1%), and to a great extent (21.3%) (S3_ tables with results for 1st research question -Table 2).

Furthermore, the Pearson correlation performed between the extent to which respondents believed that information about alternative treatments affected people's trust in

doctors and the age of the respondents, revealed a weak, negative and statistically significant correlation between the two variables (r(534)= -.155, p=0.001) (Table 3). Hence, as the age of the medical staff decreases, the extent to which they believe the credibility of doctors was affected increases. In other words, compared to older healthcare professionals, younger healthcare professionals tend to believe more that information about alternative treatments affected trust in doctors. One possible explanation for this result can be that younger people tend to be fonder of keeping up with trends and being up to date, and in this context, it is possible that they came into contact more frequently with information about certain alternative treatments for COVID – 19, this making them more aware about the way such treatments can undermine doctor's credibility.

Table 3. Pearson correlation between information about alternative treatments and age

		C14. The extent to which information about alternative treatments affected trust in physicians	D2. Age
C14.[1] The extent to which information about alternative treatments affected trust in physicians	Pearson Correlation	1	-.155**
	Sig. (2-tailed)		.000
	N	536	536
D2[2]. Age	Pearson Correlation	-.155**	1
	Sig. (2-tailed)	.000	
	N	536	536

**. Correlation is significant at the 0.01 level (2-tailed).

[1] C14 – refers to the question 14 from the section C of the manuscript (The extent to which information about alternative treatments affected trust in physicians), section which refers to Perception about the communication of non- validated treatments

[2] D2 - refers to question 2 from the D section of the manuscript (age), which refers to Sociodemographic characteristics of the respondents

In order to observe if there any differences in the opinion of the respondents depending on certain variables including, age, gender, or living environment, we performed t tests for independent samples. The results of the significant t tests (Table 4), showed that students believed to a greater extent (M= 5.60, SD=1.49), that information about alternative treatments negatively affects the credibility of doctors, than the medical staff (M=5.33, SD=1.54). Also, respondents who declared they worked in a unit without COVID – 19 patients (M=5.53, SD=1.49), were more of the opinion that information about alternative cures affected trust in health professionals, than respondents who worked in a unit with COVID – 19 patients (M=5.19, SD=1.61). One possible explanation would be that, doctors who interacted with COVID – 19 patients may have observed that when being put in the situation to receive medical care in the hospital, patients still had faith and trust in doctors. Moreover, another explanation is that respondents who did not come into contact with COVID – 19 patients were not that close with the situation and thus they might have had a more distorted perception about the situation than those professionals who interacted with COVID – 19 patients. Moreover, the results of the research also showed that female respondents (M=5.51, SD=1.48), believed more than male respondents (M=5.10, SD=1.70), that trust in healthcare professionals was affected by the information about alternative treatments.

Table 4. Significant t-test results: comparisons between variables

	t-test for Equality of Means										
	Group	N	Mean	S. D.	t	df	p	Mean Difference	Std. Error Difference	CI4 Lower	CI4 Upper
Variables:	Medical staff	294	5.33	1.54	-2.04	534	.04	-.27	.13	-.52	-.01

Information about alternative treatments _ Professional degree[1]	Student	242	5.60	1.49							
Variables: Information about alternative treatments _working unit	Unit with COVID -19 patients	122	5.19	1.61	-2.13	534	.03	-.33	.15	-.64	-.02
	Unit without COVID 19 patients	414	5.53	1.49							
Variables: Information about alternative treatments _gender	Male	76	5.10	1.70	-2.16	534	.03	-.40	.18	-.77	-.03
	Female	460	5.51	1.48							

[1]Index variable from the professional degrees of respondents. Student: medical student and student at university nursing program, Medical Staff: Senior specialist medical – doctor, Specialist medical – doctor, Resident, Nurse with higher education diploma, Nurse with other studies than higher education

2) What is the knowledge of medical staff about the type of drugs that had positive effects on treating the disease and about alternative treatments?

Considering the type of drugs which were known to have positive effects on treating the virus, the research revealed that type of drug about which the respondents have heard it had positive effects against the virus was Dexamethasone (46.6%), closely followed by Remdesivir (40.5%) and Azithromicin (38.4%). However, some of the respondents also mentioned Chloroquine, Hydroxychloroquine (23.1%), Ibuprofen (19.8%), Tocilizumab (15.9%), and Favipiravir (13.8%) as drugs known to have positive effects when dealing with COVID – 19 (S4_ Tables with results to the 2nd research question_Table 5). Hence, the research showed that the medical staff had knowledge about the type of drugs tested or used against the virus, which were taught to be efficient in treating the disease.

In the context of respondents’ perception about alternative methods of preventing and treating the virus, the findings show that, most of them stated that they heard about the fact that alcohol consumption can prevent the infection with the virus (24.3%), that drinking warm water every 15 minutes may help eliminate the virus (21.3%), but also that pointing the hot air of the hairdryer to the nostrils leads to the elimination of the virus (16.8%) (S4_ tables with results to the 2nd reseach question_Table 6).

3) How satisfied is the medical staff with the way medical and non-medical information was communicated during the pandemic?

The findings of the study revealed that respondents were mostly dissatisfied with the way medical and non – medical information was communicated during the pandemic. Hence, the sum of the responses with negative valences of the study participants (extremely dissatisfied, very dissatisfied and dissatisfied), showed that 238 of them, (44.4%) were dissatisfied with the process of sending medical and non- medical information, while the sum of the positive responses (satisfied, very satisfied, extremely satisfied) showed that 162 of them (30.2%), were satisfied with the communication process (S5_ Tables with results to the 3rd research question_Table 7). In other words, the study highlighted that respondents registered mostly low level of satisfaction with the way information was sent during the pandemic.

Furthermore, in the context of the medical staff's satisfaction with the way information about drugs used to treat the virus was communicated at national level, the research showed that as age of the respondents decreases, the level of satisfaction increases ($r(534) = -.091$, $p=0.035$) (Table 8). Thus, according to this result, it can be inferred that younger people were more satisfied than older people, with how information about drugs used to treat the virus was communicated.

Table 8. Pearson Correlation: satisfaction with the way information about drugs used to treat the virus was communicated and age

		B10. Satisfaction with the way information about drugs used to treat the virus was communicated	D2. Age
B10[1]. Satisfaction with the way information about drugs used to treat the virus was communicated	Pearson Correlation	1	-.091*
	Sig. (2-tailed)		.035
	N	536	536
D2[2]. Age	Pearson Correlation	-.091*	1
	Sig. (2-tailed)	.035	
	N	536	536

*. Correlation is significant at the 0.05 level (2-tailed).

[1] B10- refers to the question 10 from the section B of the manuscript (Satisfaction with the way information about drugs used to treat the virus was communicated) section which refers to Perception about the authorities' communication process

[2]D2 - refers to question 2 from the D section of the manuscript (age), which refers to Sociodemographic characteristics of the respondents.

Moreover, when asked to evaluate the efficiency of the communication strategies adopted by authorities in order to send information about the virus, most respondents stated that the strategies were effective. Thus, the sum of the responses with negative valences shows that 144 of them (26, 9%) described the communication strategies as inefficient, while 266 of them (49, 6%) described them as efficient (S5_ Tables with results to the 3rd research question_Table 9). One interesting result of the analysis, was that, when trying to examine if the responses of the study participants about the efficiency of communication strategies differ depending on certain variables such as working unit, gender, working unit, living environment, the analysis found no differences between the responses of males and females, of people working in units without COVID – 19 patients and people not working in units with COVID – 19 patients, or in people from the rural and urban area.

In the context of the information about drugs tested and used in the treatment against COVID – 19, the results showed that students believe to a greater extent that such information was communicated in a coherent manner (M=4.05, SD=1.63), than the medical staff (M=3.79, SD=1.53) ($t(534)= -2.05$, $p<0.05$) (Table 10.). Hence, one possible explanation for this result would be that, due the experience and knowledge of the medical staff, people who were already working in the healthcare system, such people have greater expectations from authorities when it comes to sending medical information, than medical students.

Table 10. Significant t test for information about drugs used to treat the virus and professional degree

	t-test for Equality of Means										
	Group	N	Mean	S. D.	t	df	p	Mean Difference	Std. Error Difference	CI4 Lower	CI4 Upper
Information about drugs tested and used to treat the disease[1] _ Professional degree[2]	Medical staff	294	3.79	1.53	-2.05	534	.03	-.28	.13	-.55	-.01
	Student	242	4.05	1.63							

[1] The extent to which respondents believe that information about drugs tested and used to treat the virus was communicated in a coherent manner

[2] Index variable from the professional degrees of respondents. Student: medical student and student at university nursing program, Medical Staff: Senior specialist medical – doctor, Specialist medical – doctor, Resident, Nurse with higher education diploma, Nurse with other studies than higher education

(4) What is the perception of medical staff about the role of social media in spreading misinformation about the virus?

The results of the research revealed that respondents were inclined to believe more that social media was a proper environment for spreading fake medical information during the pandemic. By analyzing the information from S6 Tables with results to the 4th research question_Table 11 , it can be observed that the sum of the responses with negative valences (4.5%) (to an extremely little extent, to a very little extent and to a little extent) is much lower than the sum of the responses with positive valences (89.9%) (to an extremely great extent, to a very great extent, to a great extent). Hence, most participants of the study believe that social media platforms favored the transmission of fake medical news during the pandemic. Furthermore, when trying to find differences in the responses of the participants depending on age, gender, living environment, professional degree or working unit (with COVID – 19 patients or without

COVID – 19 patients), we observed that their responses did not differ depending on such variables. Thus, it can be inferred that, regardless of age, gender, living environment, professional degree or working unit, respondents' perception was that social media had a role in spreading fake medical information.

However, even though respondents were of the opinion that social media was an environment in which was sent fake medical information, some of them still believe that social media platforms are appropriate for sending official information about the virus. Thus, considering the results from S6 Tables with results to the 4th research question_Table 12 , the sum of responses with positive valences (40.3%) is almost equal to the sum of responses with negative valences (45.1%) meaning that the opinions of the study participants were divided when it comes to sending official information about the virus on social media.

A factor which showed a weak but statistically significant influence on respondents' opinion about sending COVID – 19 official information on social media was age. Hence, the results of the Pearson correlation (r (534) = -.175, p=0.000), showed that as age decreases, the extent to which respondents believed that social media is an environment in which official information

about the virus should be communicated decreases (Table 13). In other words, younger respondents believed to a greater extent than older respondents that official information should also be communicated on social media. One possible explanation for this results would be that young people gather most of their information from online sources, and they also engage more with social media platforms, and thus it is possible that they would also like to see official and important information on such platforms.

Table 13. Person correlation between the extent to which social media represents an appropriate environment for sharing official COVID – 19 info and age

		C1. The extent to which social media represents an appropriate environment for sharing official COVID – 19 info	D2. Age
C1[1]. The extent to which social media represents an appropriate environment for sharing official COVID – 19 info	Pearson Correlation	1	-.175**
	Sig. (2-tailed)		.000
	N	536	536
D2[2]. Age	Pearson Correlation	-.175**	1
	Sig. (2-tailed)	.000	
	N	536	536

**. Correlation is significant at the 0.01 level (2-tailed).

[1] C1 – refers to question 1 from the section C of the manuscript (The extent to which social media represents an appropriate environment for sharing official COVID – 19 info), section which refers to Perception about the communication of non- validated treatments

[2] D2 - refers to question 2 from the D section of the manuscript (age), which refers to Sociodemographic characteristics of the respondents.

Furthermore, when dividing the study participants in medical staff (doctors, nurses) and students (medical students or students at the university nursing programs), we found that students (M=4.31, SD=2.11) believed to a greater extent than the medical staff (M= 3.88, SD=2.07) that official information about the virus should also be sent on social media (t (534) = -

2.36, p< 0.05) (Table 13). Next, when dividing the sample by living environment, participants living in the urban area (M=4.19, SD=2.10) were inclined more than those living in the rural area (M=3.72, SD=2.05), to believe that official information could also be sent on social media (t (534) = 2.23, p< 0.05) (Table 14).

Table 14. Significant t tests for sharing official information on social media professional degree and living environment

	t-test for Equality of Means										
	Group	N	Mean	S. D.	t	df	p	Mean Difference	Std. Error Difference	CI4 Lower	CI4 Upper
Official information on social media _ Professional degree[1]	Medical staff	294	3.88	2.07	-2.36	534	.01	-.42	.18	-.78	-.07
	Student	242	4.31	2.11							
Official information on social media _living environment	Urban area	411	4.19	2.10	2.23	534	.02	.47	.21	.05	.89
	Rural area	125	3.71	2.05							

[1]Index variable from the professional degrees of respondents. Student: medical student and student at university nursing program, Medical Staff: Senior specialist medical – doctor, Specialist medical – doctor, Resident, Nurse with higher education diploma, Nurse with other studies than higher education

(5) What aspects of the professional activity of the medical staff were affected most by the COVID – 19 pandemic?

The findings of our research showed that most respondents stated that the patient – doctor relationship was most affected by the pandemic (38.4%). However, a smaller percent of respondents declared that the working schedule was the most affected (26.9%), or the collaboration with their peers (23.9%) (S7 Tables with results to the 5th research question_Table 15).

Furthermore, taking into account the group of medical staff (doctors, nurses) and the group of students (medical students and students at university nursing program), the results revealed that the most respondents who stated that the patient- doctor relationship was affected most by the pandemic was the group of medical staff (144 compared to 62) (S7 Tables with results to the 5th research question_Table 16). One possible explanation for this result is that, by being in constant contact with their patients, doctors and nurses were more inclined to perceive that the relation with their patients has deteriorated during the pandemic.

Discussion

During the COVID – 19 pandemic, one of the major issues people had to face, was the spread of misinformation about the virus, its origins and its treatment. In this regard, we analyzed the perception of medical staff (including doctors, nurses, medical students and students in the university nursing program) about the way medical and non – medical information was communicated during the pandemic. In the context of the so called infodemic [11], and the effects of misinformation on people's trust in doctors, most participants of our study declared that the information about alternative treatments for the virus affected the credibility of health professionals. Hence, from this point of view, our study is in line with previous studies which highlighted the fact that lately, trust in physician decreased [67], and which suggested that social media managed to determine people to trust the personal opinions of other people rather than the opinion of the professionals [61]. Furthermore, since other researchers pointed out that many medical practitioners used social media to express professional opinions that

were later found inaccurate [74], and thus they may have contributed to the spread of misinformation [75], we argue that the credibility of physicians might have also been affected by this type of behavior.

An interesting result of our research showed that as the age of medical staff decreases, the extent to which they believe that information about alternative treatments affects doctors' credibility increases. Hence, younger healthcare professionals believed to a greater extent than older healthcare professionals, that information about alternative treatments affected negatively people's trust in doctors. This results might have as possible explanation, the fact that younger people tend to spend more time on social media platforms, and they may have interacted more than older professionals, with misinformation about the virus, this making them more able to be aware of the negative effects of fake news. Moreover, the type of unit in which the respondents worked, was a factor which influenced the opinion of the respondents, our findings showing that, the medical staff who did not work in unit with COVID -19 patients, believed to a greater extent than those who worked in such units, that information about alternative treatments negatively influenced doctors' credibility. Given this result we argue that is it possible for those professionals who did not interact with COVID -19 patients, and who thus were more distant from the situation, to have a more distorted image regarding the way people's levels of trust in them changed in the context of the pandemic.

Considering the role of social media in spreading misinformation, our study is in line with previous studies which support the idea that such channels favored the communication of fake news during the pandemic [49, 50, 51]. In this regard, regardless of age, professional degree or living environment, most healthcare professionals who participated in our study were

of the opinion that social media contributed to the spread of misinformation. However, our study also showed that when it comes to communicating official information on social media, younger respondents (students) believed to a greater extent than older respondents (doctors, nurses), that such channels should be used to send official information about the virus. Taking into account these results, the fact that healthcare professionals acknowledge that social media favors the spread of misinformation, and that many of them still believe they should be used in order to communicate official information, shows that at personal level, professionals were not affected that much by misinformation, them being able to differentiate more easily between real and fake news. In other words, we argue that while people in general were negatively influenced by the fake news they read on social media, as it was shown in previous studies which highlighted that people trusted the information on social networks, they shared un-validated information and had trouble with differentiating real from fake news [57, 79] or that exposure to health misinformation may influence people's intention to engage in certain behaviors [80], healthcare professionals may be less influenced by fake news, due to their knowledge.

Considering the knowledge of medical staff about the type of drugs that had positive effects on treating the virus, the findings of the research showed that the respondents had opinions which were in line with the results found in other studies. Hence, according to the research, most respondents stated that the drug which was known to have positive effects against the virus was Dexamethasone (46.6%), it being followed by Remdesivir (40.5%). Thus, positive effects of Dexamethasone were also highlighted by studies [31, 32], while study [35] showed positive effects of Remdesivir. Moreover, during the period in which we conducted our

research, (April – June 2021), among the drugs which were approved for administration against the virus were Remdesivir, Tocilizumab – which was authorized first in June 2021, drugs which were also acknowledged by the respondents of our research. Even more, one of the authors of the article (L.R.) is a doctor and was directly involved in the process of taking care of COVID – 19 patients, so the author can confirm that among the drugs which were in trial, or which were approved for administration against COVID-19 were also the drugs which were acknowledged by the respondents of our research.

In the context of medical staff's knowledge about alternative treatments, most respondents declared they had heard about the fact that alcohol can prevent the infection, that warm water drunk every 15 minutes, and the hot air from the hairdryer pointed to the nostrils can help eliminate the virus. From this point of view, our study is in line with a previous study [53], which also described these methods.

When it comes to the respondents' level of satisfaction about the way medical and non – medical information was communicated during the pandemic, generally, the research revealed that most respondents were dissatisfied with the communication process. In the case of communication strategies adopted by authorities, the results showed that most respondents were satisfied with them. However, in the context of sending information about the drugs used to treat the disease, the research showed that younger healthcare professionals were more satisfied with the communication process than older healthcare professionals. This results might be due to the fact that physicians with more experience have higher expectations from authorities than students.

Another area on which we focused our research was the professional activity of the medical staff during the pandemic. In this regard, our findings revealed that, according to the respondents of our study, the aspect that was mostly affected by the pandemic was the doctor-patient relationship. Hence, our research is in line with other studies [78], which showed that the pandemic affected the way doctors interacted with their patients.

Furthermore, on the basis of the results of our study we argue that not only the process of vaccination created ethical issues, but also the process of communication [81]. Thus, these ethical issues were perceived by the medical staff and they would require a further examination in order to be able to create communication guides which can be regarded as essential instruments not only for the research process of the medical staff and healthcare professionals with management positions, but also for their current medical activity [82,83].

Conclusions

During the pandemic, healthcare professionals did not have to deal only with challenges regarding their health and the health of their patients, but also with the problems created by the spread of medical misinformationAccording to the main findings of our research, generally, the medical staff (doctors, nurses, medical students, students at university nursing program), believed that information about alternative treatments affected people's trust in doctors, but younger healthcare professionals and those working in units without COVID - 19 patients believed to a greater extent than older healthcare professionals and people working in units with COVID – 19 patients that fake news about treatments for the virus affected the credibility of doctors.

Furthermore, regardless of age, age, gender, living environment, professional degree or working unit, the medical staff acknowledged the role of social media in spreading fake news, but when it comes to using social media in order to communicate official information, younger healthcare professionals were more inclined to believe that such networks were appropriate for the communication of official information.

In the context of the drugs used to treat the virus, the results pointed out that the medical staff had knowledge about the drugs known to have positive effects in treating the virus, their perception being in line with previous studies which focused on this matter.

When it comes to the influence of the pandemic on the professional activity of the medical staff, the respondents declared that the aspect which was most affected was the doctor – patient relationship. In this regard, we argue that, by influencing peoples' trust in doctors, the medical fake news spread during the pandemic, implicitly had a role in deteriorating the relation between doctors and their patients.

Therefore, the healthcare professionals were generally dissatisfied with the way medical and non – medical information was communicated during the pandemic, but younger professionals were satisfied than older professionals. Overall, the medical staff believed that fake news managed to undermine doctors' credibility that social media platforms favor the spread of such news, and they had knowledge about the drugs which were known to have positive effects on the virus and about the alternative treatments.

Taking into account the results of the research, the paper has some theoretical and practical implications. From a theoretical point of view, the paper contributes to the literature on the matter of fake news and its influence on the trust of healthcare professionals, a strength

of the paper being the fact that it analyzed the opinions of medical staff (doctors, nurses, medical students and students at university nursing program). From a practical point of view, the paper brings awareness to the phenomenon of fake news regarding medical treatments and the negative influence it has on doctors' credibility. Another practical implication refers to the fact that the paper brings attention to the issue of using social media as a mean to communicate official information, many healthcare professionals, especially the younger ones, stating that such networks could be appropriate for sharing official information. Furthermore, by highlighting that the most affected aspect of the professional activity of doctors was the relationship with their patients, the study also shows that actions need to be taken in order to restore people's trust in doctors and improve the process of communication between them.

Limitations and future research directions

While our study proved relevant information regarding the perception of healthcare professionals about the way medical and non – medical information was communicated in time of the pandemic, it also has some limitations.

One limitation is represented by the fact that the perception of healthcare professionals was studied only by using quantitative methods. In this regard, a future research should focus on obtaining information from doctors while using qualitative methods too. Next, the study was conducted only on Romanian healthcare professionals, and thus, a future research should take into consideration a comparison between the opinions of professionals from different countries. Another limitation is represented by the fact that we only asked respondents to state the aspect which was most influenced by the pandemic, but we did not asked them to offer

detail about other type of challenges encountered. Thus, a future research should focus on analyzing the extent to which aspects of the professional activity of doctors were affected, and on analyzing more deeply the challenges they had to face during the pandemic.

Furthermore, since our research revealed that many respondents believed that social media platforms could be appropriate for sharing official information, we draw attention to a problem that can arise in this context. Since people know that such platforms favor the spread of fake news, if we encourage the use of social media in order to communicate official information, don't we risk to discredit that information as it is possible for people to consider that such information is fake too? We believe that this issue should be taken into account and studied in a future research.

Author Contributions

Conceptualization: Claudiu Coman, Maria Cristina Bularca

Data curation: Claudiu Coman, Liliana Rogozea, Angela Repanovici, Maria Cristina Bularca

Formal analysis: Maria Cristina Bularca, Claudiu Coman

Investigation: Claudiu Coman, Maria Cristina Bularca, Angela Repanovici, Liliana Rogozea

Methodology: Maria Cristina Bularca, Claudiu Coman

Project administration: Claudiu Coman, Liliana Rogozea, Angela Repanovici

Resources: Maria Cristina Bularca, Liliana Rogozea, Angela Repanovici

Supervision: Claudiu Coman, Liliana Rogozea, Angela Repanovici

Writing – original draft: Maria Cristina Bularca, Claudiu Coman

Writing – review & editing: Maria Cristina Bularca, Claudiu Coman, Liliana Rogozea, Angela Repanovici

References

1. Sanders JM, Monogue ML, Jodlowski TZ, Cutrell J. B.Pharmacologic Treatments for Coronavirus Disease 2019 (COVID-19): A Review. JAMA. 2020; 323(18):1824–1836. doi:10.1001/jama.2020.6019

2. Shereen MA, Khan S, Kazmi A, Bashir N, Siddique R. COVID-19 infection: Origin, transmission, and characteristics of human coronaviruses. J Adv Res. 2020; 24:91-98 doi: 10.1016/j.jare.2020.03.005

3. Kristina SA, Herliana N, Hanifah S. The perception of role and responsibilities during covid-19 pandemic: A survey from Indonesian pharmacists. Int J Pharm Res , 2020; *12*(2). doi: 10.31838/ijpr/2020.SP2.369

4. World Health Organization (WHO). Timeline of WHO's response to COVID-19. [Internet]. World Health Organization. 2020 June 29 [cited 2020 Nov 27] Available from: https://www.who.int/news/item/29-06-2020-covidtimeline

5. European Centre for Disease Prevention and Control. COVID-19 situation update worldwide, as of 27 November 2020 [Internet]. European Centre for Disease Prevention and Control. 2020 June 29 [cited 2021 Nov 25] Available from: https://www.ecdc.europa.eu/en/geographical-distribution-2019-ncov-cases.

6. British Broadcasting Corporation. COVID vaccine: First 'milestone' vaccine offers 90% protection. [Internet]. 2020 Nov 9 [cited 2020 Nov 27] Available from: https://www.bbc.com/news/health-54873105.

7. Kommenda N, Jones FH. COVID vaccine tracker: when will a coronavirus vaccine be ready? [Internet]. The Guardian. 2020 Nov 10 [cited 2020 Nov 27] Available from: https://www.theguardian.com/world/ng-interactive/2020/nov/10/covid-vaccine-tracker-when-will-a-coronavirus-vaccine-be-ready

8. Ahsan W, Javed S, Al Bratty M, Alhazmi HA, Najmi A. Treatment of SARS-CoV-2: How far have we reached? *Drug Discov Ther*. 2020; 14(2):67-72. doi: 10.5582/ddt.2020.03008

9. Kupferschmidt K, Cohen J. Race to find COVID-19 treatments accelerates. *Science*. 2020; 367(6485): 1412-1413. doi: 10.1126/science.367.6485.1412

10. World Health Organization (WHO). Solidarity clinical trial for COVID-19 treatments [Internet]. World Health Organization 2019. [cited 2020 Nov 27] Available from: https://www.who.int/emergencies/diseases/novel-coronavirus-2019/global-research-on-novel-coronavirus-2019-ncov/solidarity-clinical-trial-for-covid-19-treatments

11. Zarocostas J. How to fight an infodemic. *The Lancet*. 2020; *395*(10225):676. doi: 10.1016/S0140-6736(20)30461-X

12. Tuccori M, Convertino I, Ferraro S, Cappello E, Valdiserra G, Focosi D et al. The Impact of the COVID-19 "Infodemic" on Drug-Utilization Behaviors: Implications for Pharmacovigilance. *Drug Saf*. 2020;43:699–709 doi:10.1007/s40264-020-00965-w

13. Rosa SGV, Santos WC. Clinical trials on drug repositioning for COVID-19 treatment. *Rev Panam Salud Publica*. 2020; 44: e40 doi: 10.26633/RPSP.2020.40

14. Sahraei Z, Shabani M, Shokouhi S, Saffaei A. Aminoquinolines against coronavirus disease 2019 (COVID-19): chloroquine or hydroxychloroquine. *Int J Antimicrob Agents*. 2020; *105945*(10.1016) doi:10.1016/j.ijantimicag.2020.105945

15. White NJ, Watson JA, Hoglund RM, Chan XHS, Cheah PY, Tarning J. COVID-19 prevention and treatment: A critical analysis of chloroquine and hydroxychloroquine clinical pharmacology. PLoS Med. 2020; 17(9): e1003252. doi:10.1371/journal.pmed.1003252

16. Liu J, Cao R, Xu M, Wang X, Zhang H, Hu H. et al. Hydroxychloroquine, a less toxic derivative of chloroquine, is effective in inhibiting SARS-CoV-2 infection in vitro. Cell Discov. 2020; 6(16) doi:10.1038/s41421-020-0156-0 .

17. Wang M, Cao R, Zhang L, Yang X, Liu J, Xu M. et.al. Remdesivir and chloroquine effectively inhibit the recently emerged novel coronavirus (2019-nCoV) in vitro. *Cell Res*. 2020; 30:269–271. doi:10.1038/s41422-020-0282-0

18. Davidescu EI, Odajiu I, Bunea T, Sandu G, Stratan L, Aramă V. et.al. Treatment with hydroxychloroquine in patients with covid-19. Experience of a neurology department. *Farmacia*. 2020; *68*(4): 597-605. doi:10.31925/farmacia.2020.4.3

19. Gautret P, Lagier JC, Parola P, Meddeb L, Mailhe M, Doudier B. et.al. Hydroxychloroquine and azithromycin as a treatment of COVID-19: results of an open-label non-randomized clinical trial. *Int. J. Antimicrob. Agents*. 2020; 105949. doi:10.1016/j.ijantimicag.2020.105949.

20. Thomson K, Nachlis H. Emergency Use Authorizations During the COVID-19 Pandemic: Lessons From Hydroxychloroquine for Vaccine Authorization and Approval. *JAMA.* 2020; 324(13):1282–1283. doi:10.1001/jama.2020.16253.

21. U.S Food & Drug Administration. FDA cautions against use of hydroxychloroquine or chloroquine for COVID-19 outside of the hospital setting or a clinical trial due to risk of heart rhythm problems. [Internet]. Food and Drug Administration. 2020 Jul 1 [cited 2020 Nov 27] Available from: https://www.fda.gov/drugs/drug-safety-and-availability/fda-cautions-against-use-hydroxychloroquine-or-chloroquine-covid-19-outside-hospital-setting-or .

22. Recovery. No clinical benefit from use of hydroxychloroquine in hospitalized patients with COVID-19. [Internet]. Recovery 2020 June 5 [cited 2020 Nov 27] Available from: https://www.recoverytrial.net/news/statement-from-the-chief-investigators-of-the-randomised-evaluation-of-covid-19-therapy-recovery-trial-on-hydroxychloroquine-5-june-2020-no-clinical-benefit-from-use-of-hydroxychloroquine-in-hospitalised-patients-with-covid-19 .

23. Naveed M, Uddin S, Abdullah KS, Ishaq SE, Ahmad T. Various Evidence-Based Hypothetical and Experimental Treatment Approaches and Their Effectiveness against COVID-19 Worldwide: A Comprehensive Literature Review. *EJMO* 2020; 4(4):265–285. doi: 10.14744/ejmo.2020.52538

24. Chu C M, Cheng VCC, Hung IFN, Wong MML, Chan KH, Chan KS, et.al. Role of lopinavir/ritonavir in the treatment of SARS: initial virological and clinical findings. *Thorax*. 2004; 59(3):252-256. doi:10.1136/thorax.2003.012658

25. Cao B, Wang Y, Wen D, Liu W, Wang J, Fan G, et al. A trial of lopinavir-ritonavir in adults hospitalized with severe covid-19. *N. Engl . J. Med.* 2020; 382(19): 1787-1799. doi:10.1056/NEJMoa2001282

26. Vosu J, Britton P, Howard-Jones A, Isaacs D, Kesson A, Khatami A, et al. Is the risk of ibuprofen or other non-steroidal anti-inflammatory drugs increased in COVID-19?. *J Paediatr Child Health*. 2020; *56*(10): 1645-1646. doi:10.1111/jpc.15159

27. Fang L, Karakiulakis G, Roth M. Are patients with hypertension and diabetes mellitus at increased risk for COVID-19 infection? *Lancet Respir Med*. 2020; 8(4):e21.8 doi:10.1016/S2213-2600(20)30116-8

28. Science alert. Updated: WHO Now Doesn't Recommend Avoiding Ibuprofen For COVID-19 Symptoms [Internet]. Science alert 2020 Mar 17 [cited 2020 Nov 27]. Available from: https://www.sciencealert.com/who-recommends-to-avoid-taking-ibuprofen-for-covid-19-symptoms

29. Esba LCA, Alqahtani RA, Thomas A, Shamas N, Alswaidan L, Mardawi G. Ibuprofen and NSAID Use in COVID-19 Infected Patients Is Not Associated with Worse Outcomes: A Prospective Cohort Study. *Infect Dis Ther*. 2020; 1-16. doi:10.1007/s40121-020-00363-w

30. Rinott E, Kozer E, Shapira Y, Bar-Haim A, Youngster I. Ibuprofen use and clinical outcomes in COVID-19 patients. *Clin Microbiol Infect*. 2020; 26(9):1259.e5-1259.e7. doi:10.1016/j.cmi.2020.06.003

31. Roberts M. Coronavirus: Dexamethasone proves first life-saving drug. [Internet]. British Broadcasting Corporation 2020 June 16 [cited 2020 Nov 27] Available from: https://www.bbc.com/news/health-53061281.

32. Horby P, Lim WS, Emberson JR, Mafham M, Bell JL, Linsell L, et al.. Dexamethasone in hospitalized patients with Covid-19-preliminary report. *N. Engl. J. Med*. 2020. doi:10.1056/nejmoa2021436

33. Lu CC, Chen MY, Lee WS, Chang YL. Potential therapeutic agents against COVID-19: What we know so far. *J Chin Med Assoc*. 2020; 83(6):534-536. doi: 10.1097/JCMA.0000000000000318

34. Wang Y, Zhang D, Du G, Du R, Zhao J, Jin Y, et al. Remdesivir in adults with severe COVID-19: a randomised, double-blind, placebo-controlled, multicentre trial. *The Lancet*. 2020; 395(10236):1569-1578. doi:10.1016/S0140-6736(20)31022-9

35. Beigel JH, Tomashek KM, Dodd LE, Mehta AK, Zingman BS, Kalil AC. et al. Remdesivir for the treatment of Covid-19. *N. Engl. J. Med*. 2020. https://www.nejm.org/doi/10.1056/NEJMoa2007764

36. U.S Food and Drug Administration. FDA Approves First Treatment for COVID-19 [Internet]. Food and Drug Administration 2020 Oct 22 [cited 2020 Nov 27] Available from: https://www.fda.gov/news-events/press-announcements/fda-approves-first-treatment-covid-19.

37. U.S Food and Drug Administration. Coronavirus (COVID-19) Update: November 20, 2020 [Internet]. Food and Drug Administration 2020 Nov 20 [cited 2020 Nov 27] Available from: https://www.fda.gov/news-events/press-announcements/coronavirus-covid-19-update-november-20-2020.

38. Glasdam S, Stjernswärd S. Information about the COVID-19 pandemic–a thematic analysis of different ways of perceiving true and untrue information. *SSHOP.* 2020; (2)1: 100090. https://doi.org/10.1016/j.ssaho.2020.100090

39. Caleb TC, Hayes RA. Social Media: Defining, Developing, and Divining. *Atl J Commun.*2015; 23:1: 46-65. https://doi.org/10.1080/15456870.2015.972282

40. Boyd DM, Ellison NB.Social network sites: Definition, history, and scholarship. *J Comput Mediat Commun.* 2007; *13*(1): 210-230. https://doi.org/10.1111/j.1083-6101.2007.00393.x

41. Reuter C, Stieglitz S, Imran M.Social media in conflicts and crisesBehav. *Inf. Technol*. 2020; *39*(3): 241-251. https://doi.org/10.1080/0144929X.2019.1629025

42. Vasconcellos-Silva PR, Castiel LD. COVID-19, fake news, and the sleep of communicative reason producing monsters: the narrative of risks and the risks of narratives. *Cad Saude Publica*. 2020); *36*(7): e00101920. https://doi.org/10.1590/0102-311x00101920

43. Pulido CM, Ruiz-Eugeni L, Redondo-Sama G, Villarejo-Carballido B. A New Application of Social Impact in Social Media for Overcoming Fake News in Health. *Int. J. Environ. Res. Public Health*. 2020; *17*(7):2430. **https://doi.org/10.3390/ijerph17072430**

44. Al-Dmour H, Salman A, Abuhashesh M, Al-Dmour R.Influence of social media platforms on public health protection against the COVID-19 pandemic via the mediating effects of public health awareness and behavioral changes: integrated model. *J. Medical Internet Res*. 2020; *22*(8):e19996.

45. Wong JEL, Leo YS, Tan CC. COVID-19 in Singapore—Current Experience: Critical Global Issues That Require Attention and Action. *JAMA.* 2020; 323(13):1243–1244. doi:10.1001/jama.2020.2467

46. Lazer DM, Baum MA, Benkler Y, Berinsky AJ, Greenhill KM, Menczer F, et al. The science of fake news. *Science*. 2018; *359*(6380):1094-1096. DOI: 10.1126/science.aao2998

47. Moscadelli A, Albora G, Biamonte MA, Giorgetti D, Innocenzio M, Paoli S, et al. Fake News and Covid-19 in Italy: Results of a Quantitative Observational Study. *Int. J. Environ. Res. Public Health.* 2020; *17*:5850

48. Mian A, Khan S. Coronavirus: The spread of misinformation. *BMC Medicine*. 2020; 18(1):1-2 doi:10.1186/s12916-020-01556-3 \

49. Pan American Health Organization. Understanding the infodemic and misinformation in the fight against covid-19. [Internet]. Pan American Health Organization 2020 May 1 [cited 2020 Nov 27] Available from: https://www.paho.org/en/documents/understanding-infodemic-and-misinformation-fight-against-covid-19.

50. Bowles J, Larreguy H, Liu, S. Countering misinformation via WhatsApp: Preliminary evidence from the COVID-19 pandemic in Zimbabwe. *PloS one*. 2020; *15*(10): e0240005. https://doi.org/10.1371/journal.pone.0240005

51. Ittefaq M, Hussain SA, Fatima M. COVID-19 and social-politics of medical misinformation on social media in Pakistan. *Media Asia*. 2020; *47*(1-2): 75-80. https://doi.org/10.1080/01296612.2020.1817264

52. The National Law Review. There's a Fake News Pandemic. Could COVID-19 and Trademarks be the Cure? [Internet]. The National Law Review 2020 Jul 7 [cited 2020 Nov 27] Available from: https://www.natlawreview.com/article/there-s-fake-news-pandemic-could-covid-19-and-trademarks-be-cure

53. O'connor C, Murphy M. Going Viral: Doctors Must Combat Fake News in the Fight against Covid-19. *Ir Med J*. 2020; 113(5): 85-85.

54. Pew Research Center. Nearly three-in-ten Americans believe COVID-19 was made in a lab. [Internet]. Pew research center 2020 April 8 [cited 2020 Nov 27] Available from: https://www.pewresearch.org/fact-tank/2020/04/08/nearly-three-in-ten-americans-believe-covid-19-was-made-in-a-lab/

55. Health Analytics Asia. 50 Fake 'frequently forwarded' COVID-19 WhatsApp messages. [Internet]. Health Analytics Asia 2020 April 2 [cited 2020 Nov 27] Available from: https://www.ha-asia.com/50-fake-frequently-forwarded-covid-19-whatsapp-messages/

56. Brennen JS, Simon F, Howard PN, Nielsen RK. Types, sources, and claims of COVID-19 misinformation. *Reuters Institute*. 2020; *7*: 1-13.

57. Pennycook G, McPhetres J, Zhang Y, Lu JG, Rand DG. Fighting COVID-19 misinformation on social media: Experimental evidence for a scalable accuracy-nudge intervention. *Psychol*. 2020; *31*(7): 770-780. https://doi.org/10.1177/0956797620939054

58. Barua Z, Barua S, Aktar S, Kabir N, Li M. Effects of misinformation on COVID-19 individual responses and recommendations for resilience of disastrous consequences of misinformation. *Prog Disaster Science*. 2020; *8*: 100119. doi:10.1016/j.pdisas.2020.100119

59. Ahmed N, Shahbaz T, Shamim A, Khan KS, Hussain SM, Usman A. The COVID-19 Infodemic: A Quantitative Analysis Through Facebook. *Cureus.* 2020; 12(11): e11346. doi: 10.7759/cureus.11346

60. Nagler RH, Vogel RI, Gollust SE, Rothman AJ, Fowler EF, Yzer MC. Public perceptions of conflicting information surrounding COVID-19: Results from a nationally representative survey of US adults. *PloS one*. 2020; *15*(10): e0240776. doi:10.1371/journal.pone.0240776

61. Orso D, Federici N, Copetti R, Vetrugno L, Bove T. Infodemic and the spread of fake news in the COVID-19-era. *Eur J Emerg Med*. 2020; 27(5):327-328. doi:10.1097/MEJ.0000000000000713

62. McNab C. What social media offers to health professionals and citizens. *Bull World Health Organ*. 2009; 87(8):566. doi:10.2471/blt.09.066712

63. Ma X, Vervoort D, Luc JG. When misinformation goes viral: access to evidence-based information in the COVID-19 pandemic. *J. Glob. Health. Sci*. 2020; *2*(1):e13 doi:10.35500/jghs.2020.2.e13

64. Tasnim S, Hossain MM, Mazumder H. Impact of rumors and misinformation on COVID-19 in social media. *J Prev Med Public Health*. 2020; *53*(3):171-174.

65. Bode L, Vraga EK.See something, say something: Correction of global health misinformation on social media. J Health Commu. 2018; *33*(9):1131-1140. doi:10.1080/10410236.2017.1331312

66. Chou WYS, Oh A, Klein WM. Addressing health-related misinformation on social media. *Jama*. 2018; *320*(23): 2417-2418. doi:10.1001/jama.2018.16865.

67. Stasiuk K, Polak M, Dolinski D, Maciuszek J. The credibility of health information sources as predictors of attitudes toward vaccination—the results from a longitudinal study in Poland. *Vaccines*. 2021; *9*(8):933 doi: 10.3390/vaccines9080933

68. Cernicova-Buca M, Palea A. An appraisal of communication practices demonstrated by romanian district public health authorities at the outbreak of the COVID-19 pandemic. *Sustainability*. 2021; *13*(5): 1-19, doi: 10.3390/su13052500

69. Tagliacozzo S, Albrecht F, Ganapati NE. International Perspectives on COVID-19 Communication Ecologies: Public Health Agencies' Online Communication in Italy, Sweden, and the United States. *Am Behav Sci*. 2021; *65*(7), 934-955 doi: 10.1177/0002764221992832

70. Saechang O, Yu J, Li Y. Public trust and policy compliance during the COVID-19 pandemic: The role of professional trust. *Healthcare*. 2021; 9 (2):1-13 doi: 10.3390/healthcare9020151.

71. Lewandowski R, Goncharuk AG, Cirella GT. Restoring patient trust in healthcare: medical information impact case study in Poland. *BMC Health Serv. Res.* 2021; *21*(1):1-11 doi: 10.1186/s12913-021-06879-2

72. Soveri A, Karlsson LC, Antfolk J, Lindfelt M, Lewandowsky S. Unwillingness to engage in behaviors that protect against COVID-19: the role of conspiracy beliefs, trust, and endorsement of complementary and alternative medicine. *BMC Public Health*. 2021; *21*(1): 1-12 doi: 10.1186/s12889-021-10643-w

73. Antinyan A, Bassetti T, Corazzini L, Pavesi F. Trust in the health system and COVID-19 treatment. Front. Psychol.. 2021; *12*:1-14 doi:10.3389/fpsyg.2021.643758

74. Law RW, Kanagasingam S, Choong KA. Sensationalist social media usage by doctors and dentists during Covid-19. *Digit. Health*. 2021; *7*:1-12 doi: 10.1177/20552076211028034

75. Leonard MB, Pursley DM, Robinson LA, Abman SH, Davis JM. The importance of trustworthiness: lessons from the COVID-19 pandemic. Pediatr. Res. 2021; 1-4 doi:10.1038/s41390-021-01866-z

76. Wood JL, Lee GY, Stinnett SS, Southwell BG. A Pilot Study of Medical Misinformation Perceptions and Training Among Practitioners in North Carolina (USA). *INQUIRY: The Journal of Health Care Organization, Provision, and Financing*, 2021; *58*:1-6 doi: 10.1177/00469580211035742

77. Gopichandran V, Sakthivel K. Doctor-patient communication and trust in doctors during COVID 19 times—A cross sectional study in Chennai, India. *Plos One*. 2021: *16*(6), 1-11 doi: 10.1371/journal.pone.0253497

78. Nwoga HO, Ajuba, MO, Ezeoke UE. Effect of COVID-19 on doctor-patient relationship. *Int J Community Med Public Health*. 2020; *7(12)*: 2394-6040, doi: 10.18203/2394-6040.ijcmph20205136

79. Rocha YM, de Moura GA, Desidério GA, de Oliveira CH, Lourenço FD, de Figueiredo NLD. The impact of fake news on social media and its influence on health during the COVID-19 pandemic: A systematic review. *J. Public Health*. 2021; 1-10 doi:10.1007/s10389-021-01658-z

80. Greene CM, Murphy G. Quantifying the effects of fake news on behavior: Evidence from a study of COVID-19 misinformation. *J Exp Psychol Appl*. 2021 Dec;27(4):773-784. doi: 10.1037/xap0000371.**81**.Rogozea LM, Sechel G, Bularca MC, Coman C, Cocuz ME. Who's Getting Shots First? Dealing With the Ethical Responsibility for Prioritizing Population Groups in Vaccination. *Am J Ther*. 2021 Jun 22;28(4):e478-e487. doi: 10.1097/MJT.0000000000001400. PMID: 34228653.

82. Rogozea L, Purcaru D, Leaşu F, Nemet C. Biomedical research - opportunities and ethical challenges. *Rom J Morphol Embryol.* 2014;55(2 Suppl):719-22. PMID: 25178352.

83. Olimid AP, Rogozea LM, Olimid DA. Ethical approach to the genetic, biometric and health data protection and processing in the new EU General Data Protection Regulation (2018). *Rom J Morphol Embryol*. 2018;59(2):631-636. PMID: 30173275.

Supporting information

S1 Appendix English version of the questionnaire

(docx)

S2 Appendix Romanian version of the questionnaire

(docx)

S3 Tables with results to the 1st research question

S4 Tables with results to the 2nd research question_

S5 Tables with results to the 3rd research question_

S6 Tables with results to the 4th research question_

S7 Tables with results to the 5th research question_

Click here to access/download
Supporting Information - Compressed/ZIP File Archive
Supporting information_revised manuscript.zip

~~Challenges in the communication process during the COVID-19 pandemic- a perspective of medical staff~~ Misinformation about medication during the COVID – 19 pandemic: a perspective of medical staff

Claudiu Coman[1#a*], Maria Cristina Bularca[1], Angela Repanovici[2], Liliana Rogozea[3]

1 Department of Social Sciences and Communication, Faculty of Sociology and Communication, Transilvania University of Brasov, Brasov, Romania;

2 Department of Product Design, Mechatronics and Environment, Faculty of Product Design and Environment, Transilvania University of Brasov, Brasov, Romania

3 Basic, Preventive and Clinical Sciences Department, Transilvania University of Brasov, Brasov, Romania;

[#a] Current address: Department of Social Sciences and Communication, Faculty of Sociology and Communication, Transilvania University of Braşov, Brasov, România

[*] Corresponding author

E-mail: claudiu.coman@unitbv.ro (CC)

Abstract

Background. Healthcare professionals had to face numerous challenges during the pandemic, their professional activity being influenced not only by the virus, but also by the spread of medical misinformation. In this regard, we aimed to analyze, from the perspective of medical staff, the way medical and non - medical information about the virus was communicated during the pandemic in order to raise awareness about the way misinformation affected the medical staff.

Methods and findings. The study was conducted on Romanian healthcare professionals including doctors, nurses and medical students. They were asked to answer to a questionnaire and the sample of the research includes 536 respondents. The findings revealed that most respondents stated that information about alternative treatments against the virus affected the credibility of health professionals, and that younger professionals believed to a greater extent that trust in doctors was affected. The research also showed that respondents were well informed about the drugs used in clinical trials in order to treat the virus, and that younger respondents believed that social media should be used to send official information. Among the main limitations of our study we mention the fact that we used only quantitative methods and the fact we focused only on Romanian healthcare professionals.

Conclusions. Healthcare professionals declared that the spread of misinformation regarding alternative treatments, affected their credibility and the relationship with their patients. Healthcare professionals had knowledge about the drugs used in clinical trials, and they acknowledged the role of social media in spreading medical misinformation. However, younger

professionals also believed that social media could be used to share official information about the virus. A future research should focus on studying the opinion of Romanian and international doctors, it should use qualitative methods too and should address the issue of social media being an appropriate environment for sending official information.

Background. Healthcare professionals had to face numerous challenges during the pandemic, their professional activity being influenced not only by the virus, but also by the spread of medical misinformation. In this regard, we aimed to analyze, from the perspective of medical staff, the way medical and non - medical information about the virus was communicated during the pandemic to encourage the development of future research or interventions in order to raise awareness about the way misinformation affected medical staff.

Methods and findings. The study was conducted on Romanian healthcare professionals. They were asked to answer to a questionnaire and the sample of the research includes 536 respondents. The findings revealed that most respondents stated that information about alternative treatments against the virus affected the credibility of health professionals, and that younger professionals believed to a greater extent that trust in doctors was affected. The research also showed that respondents were well informed about the drugs used in clinical trials in order to treat the virus.

Conclusions. Healthcare professionals declared that the spread of misinformation regarding alternative treatments, affected their credibility and the relationship with their patients. Healthcare professionals had knowledge about the drugs used in clinical trials, and they acknowledged the role of social media in spreading medical misinformation. However, younger

professionals also believed that social media could be used to share official information about the virus.

Introduction

The COVID 19 pandemic generated multiple changes in the way today's society members carry out their daily activities. One of the processes which was mostly affected by the pandemic was the communication process between institutions and the public, as well as between individuals. In this regard, from this perspective, ~~W~~while many domains were affected by the spread of the virus, such as the educational system or the cultural sector, the health sector was the one that faced the most challenges, ~~the pandemic managing to generate a tremendous global public health crisis~~ [1].

~~Caused by severe acute respiratory syndrome coronavirus 2 (SARS CoV 2) [2], the disease was firstly detected in December 2019, in Wuhan, China [3], and it fastly spread all over the world. The World Health Organization was informed about a pneumonia outbreak in Wuhan on December 31 2019, the number of cases continued to increase, and on March 11 2020 the World Health Organization characterized COVID 19 as a pandemic [4]. Being highly contagious, the virus affected a large number of people, and as of November 27 over 61 million cases were reported [5]. Even though many companies and institutions are struggling to develop a vaccine, Pfizer, Gamaleya Research Institute, University of Oxford, and a preliminary analysis of the vaccine proposed by Pfizer showed that the vaccine is able to prevent more than 90% of people from getting infected with COVID 19 [6], so far no vaccine was approved as a general and universal vaccine against COVID 19 [7].~~

Ever since the pandemic was declared, finding the right treatment for the virus has become a priority for researchers and doctors from all over the world. In this regard, large number of trials started to be conducted, and in order to find an efficient drug treatment against the virus, one method that was adopted was testing and administrating to patients, drugs that were previously used for curing other viruses [8]. Thus, on March 20 2020, The World Health Organization launched the SOLIDARITY clinical trial, a trial that monitored the effects on patients infected with COVID 19, of specific drugs that proven to be effective in the treatment of other diseases: remdesivir, interferon beta, chloroquine and hydroxychloroquine - previously used for Malaria, as well as drugs used on HIV patients: lopinavir and ritonavir [9]. However, according to the interim results published on October 15 2020 by WHO, even though those drugs were taught to have positive effects on treating COVID 19, they had little influence or no influence at all on mortality in general, on the need and initiation of ventilation and on the recovery process [10].

"Caused by severe acute respiratory syndrome coronavirus 2 [2], the disease was firstly detected in December 2019, in Wuhan, China [3]. Due to the evolution of the virus, the World Health Organization declared the pandemic in March 2020 [4], and as of November 27 over 61 million cases were reported [5]. In this regard, although several companies are struggling to develop a vaccine, and some of the proposed vaccines showed promising results [6], so far no vaccine was approved in order to be administrated to the entire population [7]. Ever since the pandemic was declared, many companies started to be preoccupied with finding a treatment, and one method used that was adopted was administrating to patients, drugs that were previously used for curing other viruses [8]. Thus, one of the most well - known trials started

was the SOLIDARITY trial, which focused on using various drugs including chloroquine and hydroxychloroquine, lopinavir or ritonavir [9]. However, even if those drugs were taught to have positive effects on treating the virus, they did not have a significant influence on preventing mortality in general [10].

With the development of many trials and programs meant to find a cure for COVID 19 and with the use of diverse drug combinations, another major problem arose: misinformation and fake news about the virus, its treatment or methods to combat it. In this regard, along with the pandemic, people also had to face an epidemic of information, described by the general director of WHO as an „infodemic” [11]. In other words, information about COVID 19 began to be spread by people on every available communication channel, both in the online and offline environment. However, very often and especially on social media, the information was poorly communicated, it was distorted and there usually wasn’t enough scientific evidence to demonstrate its validity [12].

Taking into account the previously mentioned aspects the paper addresses the issues of drugs tested and used for the treatment of COVID 19 and how information about COVID 19 was communicated in the offline and online environment. ~~The purpose of the paper is to analyze, from the perspective of medical staff, the way medical and non - medical information about the virus was communicated during the pandemic in order to raise awareness about the way misinformation affected medical staff.~~ The purpose of the paper is to analyze, from the perspective of medical staff, the way medical and non - medical information about the virus was communicated during the pandemic in order to encourage the development of future research or interversions in order to raise awareness about the way misinformation affected

medical staff. Thus, the paper aims at finding an answer to three research questions: (1) to what extent information about alternative treatments affected the credibility of medical staff? (2) What is the knowledge of medical staff about the type of drugs that had positive effects on treating the disease and about alternative treatments? (3) How satisfied is the medical staff with the way medical and non-medical information was communicated online and offline during the pandemic? (4) What is the perception of medical staff about the role of social media in spreading misinformation about the virus? (5) What aspects of the professional activity of the medical staff were affected most by the COVID – 19 pandemic?

Formatted: Font color: Text 1

~~Hence, considering the purpose of our paper and the research questions, we believed it was necessary to analyze the literature on the drugs used to treat COVID – 19, on the role of social media platforms in spreading fake information about the virus and potential treatments, and on the way the pandemic influenced the credibility of doctors and their relationship with their patients.~~

Literature review

Information on drugs used to treat COVID 19

Before analyzing the way information about the virus was communicated in the online environment, it is important to take a look at the drugs used to treat the disease. Hence, one of the most important issues that appeared with the COVID 19 pandemic, was finding the right treatment for the virus. In this regard, researchers started to develop many experimental trials and used diversified drug combinations in order to treat patients with COVID 19. However, information that was communicated about the effectiveness of certain drugs was often contradictory.

Chloroquine and hydroxychloroquine are two drugs that were tested and included in many trials. Both drugs were previously used to treat malaria but they also have antiviral effects on viruses like HIV since they have the ability to prevent the virus to enter in the host cells [13]. Even though they have similar compounds, chloroquine is taught to have more negative effects than hydroxychloroquine [14], and hydroxychloroquine is considered safer due to the fact that it can be tolerated better for a longer period of time [15].

While some studies show positive effects of hydroxychloroquine in inhibiting the infection with the virus in vitro [16, 17], other studies found no influence of the drug on mortality rate or time spent by patients in the hospital [18]. However, when hydroxychloroquine was combined with other drugs such as azithromycin, it showed beneficial effects in treating patients with COVID 19 [19].

Nonetheless the findings regarded the effectiveness of these drugs were contrasting. For example, on March 28 2020 the Food and Drug Administration (FDA) issued an Emergency Use Authorization for using hydroxychloroquine in treating people suffering from COVID 19 [20], and in June 15 2020, the FDA retracted the authorization stating that the trials in which the drug was involved showed that the drug had no effect on the faster recovery of patients or on decreasing chances of death [21]. Even more, on 5th June 2020 the UK trial, Randomised Evaluation of COVID 19 THERAPY (RECOVERY), also stopped testing the drug on patients because the results showed no benefits in improving the conditions of hospitalized patients with COVID 19 [22].

Studies were carried out with other drugs such as lopinavir/ritonavir, an antiviral drug used in the treatment of HIV [23]. While in concentration of 4 µg/ml and 50 µg/ml, the drug

showed positive effects against the virus in vitro [24], a study on 199 patients, from which 99 received the drug and the other 100 did not receive the drug, revealed that lopinavir/ritonavir had no benefits when it comes to diminishing mortality or improving the state of patients with severe symptoms [25].

Controversial discussions also involved the use of Ibuprofen, a Non-steroidal anti-inflammatory drug that is used to treat fever, or inflammation [26]. Since the pandemic was declared there has been a preoccupation regarding ibuprofen and its role in making people more vulnerable to contacting the virus. Thus, right after the declaration of the pandemic, in a letter addressed to The Lancer Journal, researchers pointed out that ibuprofen could make people with diabetes, cardiac disease or hypertension more likely to get infected with virus and have severe symptoms [27]. However, while firstly, WHO recommended people who are infected with the virus not to take ibuprofen, only one day after that recommendation, on 18 March 2020, WHO corrected its statement and mentioned that it ”does not recommend against ibuprofen” [28]. Even more, a study focusing on the use of ibuprofen showed that the drug does not make patients feel worse [29] and another study that analyzed the use of ibuprofen and paracetamol of 403 COVID 19 confirmed patients revealed that compared to paracetamol, ibuprofen did not aggravated the clinical state of the patients [30].

While other drugs failed to show beneficial effects on the treatment of COVID 19, drugs like dexamethasone, which is included in the UK RECOVERY trial, revealed positive effects on people suffering from COVID 19: the drug lowered the risk of death in patients on ventilators from 40% to 28% and in patients who were in need of oxygen, from 25% to 20%, but did not influence the state of patients who did not need oxygen [31, 32].

Another highly tested drug was Remdesivir, an antiviral drug produced by Gilead Sciences that was previously used in treating Ebola [33]. The information regarding its positive effects on treating COVID 19 is also contradictory. A study conducted from February 6 2020 until March 12 2020, on 237 patients, showed that the drug did not bring any benefits for people that had severe symptoms of COVID 19 [34], while a more recent study revealed that Remdesivir had a more positive effect in reducing the time of recovery in patients with COVID 19 that showed signs of respiratory issues, than it had the placebo effect [35]. However, the FDA approved on October 22 2020, the use of Remdesivir in the case of adults and also children aged 12 or older who have at least 44 kilograms, who are infected with the virus and need to be treated in the hospital [36], and as of November 20 2020, FDA allows, in emergency cases, the use of Remdesivir in combination with Baricitinib, for adults and children aged two or older that require oxygen and treatment in the hospital [37].

Social media and COVID 19 misinformation

Together with the health crisis, the COVID 19 pandemic generated an information crisis, often described as an infodemic, that is represented by the spread of fake news, misguided and false information, especially in the online environment [38].

In this context, social media plays an essential role in disseminating information. Social media consists of internet based channels that provide people with the opportunity to interact, communicate in asynchronous way and in real time, with either small or large audiences where value is derived from user generated content [39]. Social media comprises multiple social networks, which according to Boyd and Ellison, offer users the possibility to create profiles that

are public, or semi-public, to create a list of people with whom they can interact and share information and to view the list of connections that other users make [40].

Social media channels are often used in time of crisis not only by citizen, but also by official authorities, emergency services, because they can facilitate communication and the spread of valuable information that can contribute to surpassing the crisis [41]. Social networks like Facebook, Whatsapp, Twitter, Instagram can function as sources that have the ability to confirm or complete the information communicated by the authorities, while also receiving feedback from the public [42]. Thus, sending messages through social media channels is a strategy that can help authorities obtain feedback on certain proposals regarding public health policies [43]. Even more, a study regarding the influence of social media on the way people protect their health during the pandemic, showed that social media can have positive impact on increasing awareness about public health and protection against the virus [44].

However, during the pandemic, while authorities can use social media to keep the public informed, a major issue generated by social media, that public health representatives have to face, is the spread of fake news [45].

Fake news are represented by fabricated information designed in the form of news communicated by the media that do not share the same process of organization and do not have the same intent, and fake news are related to misinformation: information that is false or misleading, and disinformation: a type of false information whose aim is to deceive people [46].

Thus, the internet became a favorable environment for spreading conspiracy theories or false information about alternative treatment for the virus. Since people were stressed and frightened by the uncertainty of the situation, they started to consider reasonable and valid any

information that presented explanations in regards to the virus [47]. Thus, when referring to health information, false news often undermine the credibility of official sources, they create confusion among people and favor the faster spread of the virus [48].

Misinformation during the pandemic can negatively influence peoples' health because false information is not easy to recognize, because it can determine people to change their behavior in a way that is harmful to their health and those around them. Thus, since the pandemic was declared, false information has been spread about the origin of the virus, about what caused it, how it spreads and what treatment is efficient for eliminating it [49]. However, a study focusing on the WhatsApp platform showed that when the information on social media is shared by trusted sources, it can increase knowledge about the virus and encourage people to adopt preventive behavior [50].

During the time of crisis, on platforms like WhatsApp or Facebook, more and more false news and unverified information about the virus began to be shared. With millions of users worldwide, WhatsApp became one of the platforms where most fake news were shared by forwarding messages to many users [51], while Facebook was characterized as the core, epicenter of misinformation [52].

When it comes to health misinformation on social media, the most discussed subjects are alternative cures involving certain food or drinks, hygiene related actions and treatment drugs. Thus, among the most "recommended" practices for preventing or curing COVID were drinking hot water every 15 minutes in order for the virus to go into the stomach, eating garlic, taking vitamin C or even pointing a hairdryer to the nostrils because the heat could eliminate the virus [53].

False news that circulated on social media regarding the virus also involve the idea that the virus was created on purpose in a lab, three in ten Americans considering true this information [54].

However, many other unverified methods were shared and the most forwarded messages on WhatsApp presented information about the fact that if people hold their breath for ten seconds without coughing then they are not infected with the virus, about the idea that at temperatures of 30-35 Celsius degrees the virus will die, messages about the release of the vaccine or about drugs allegedly recommended by Chinese doctors that could be efficient in eliminating the virus [55].

Nonetheless, misinformation became a major issue in the context of the pandemic, but also a subject of interest for researchers. A study focusing on the spread of fake news showed that most news reconfigure and twist the original information thus creating a different context, and that most of them contain false information about public authorities and health organizations [56].

Another study found that people who tend to rely on their intuition or who possess little scientific knowledge about certain subjects, encountered difficulties in differentiating true and false information [57]. Thus, misleading or unverified information can negatively influence the way people behave. For example, people in USA who died after they consumed chloroquine may have used the drug because news about it mentioned that it could treat and eliminate the virus [58]. Even more, a study concerning misinformation on Facebook revealed that posts made from verified accounts contained more false information than the accounts that were not verified [59], while other study conducted from 23 April 2020 to 27 April 2020, focused on

perception about contradictory information and stated that 73% of participants mentioned they observed or were exposed to contrasting messages usually communicated by politicians or health experts [60].

Apart from influencing peoples' beliefs or health practices, COVID 19 fake news also influenced the activity of health professionals. Social media managed to increase the level of trust in information that comes from people's personal opinions rather than professionals [61], and doctor's credibility is often affected. In order to improve these situations, doctors must be willing to use social media not just to send messages, but to actively communicate with people, to offer feedback, to share their experiences and rectify and clarify the fake news presented on social media [62].

Among action from health professionals, in order to combat COVID 19 fake news, social media networks as well as public authorities must implement some strategies. For example, the government of United Kingdom developed collaboration programs between its rapid response teams and social media platforms, and Taiwan introduced greater fines for news that were proven to be false [63]. Moreover, even though some social networks such as Facebook or Twitter already implemented algorithms to identity and remove fake accounts [64], or to correct information [65], they should further develop efficient strategies in order to validate the information that people share [66].

The influence of the pandemic on doctors' credibility and relationship with patients

The way information regarding the virus was communicated online and offline during the pandemic played an essential role in the process of maintaining trust in health professionals. In

this regard, a previous longitudinal study conducted in Poland revealed that trust in physicians has declined from 2018 – 2020, and emphasized the idea that the decrease may be caused by the health problems that people had to cope with during the pandemic and the problems with the healthcare system of the country [67]. In Romanian context, a previous study showed that the communication process of the healthcare system was poor and confusing, and that public health authorities at national level focused more on global information about the virus, while local authorities failed to succeed in providing their "share of information" [68]. Another study, which focused on analyzing the online communication of Public Health Agencies from Italy, United States and Sweden, revealed that compared to Sweden and the United States, agencies from Italy collaborated more with other organizations, and that overall, the communication process of the agencies was coordinated by their members, that agencies also communicated with governments, but they rarely collaborated with political or non-governmental organizations [69]. Hence, while trust in the government and communication from authorized organizations is essential, the importance of trusting the professionals is highlighted by a study conducted in Thailand, which showed that in the cases in which people have low levels of trust in the government, trust in professionals can have a positive influence on the adoption of protective measures at the individual level [70].

Furthermore, another previous study conducted in Poland, revealed that information can have the power to influence the level of trust that people have in the healthcare system and in healthcare professionals, suggesting that an increase of trust in hospitals, may be associated with a decrease of trust in physicians [71].

While focusing on studying people’s response to non- pharmaceutical interventions, conspiracy theories and alternative treatments, a study conducted in Finland showed that the level of trust people have in the system implemented in order to provide information about the virus, has an essential role in the way people react to the official measures recommended. Hence, most participants in the study were between 40 and 60 years of age, and the study emphasized that people who were less willing to comply with the non-pharmaceutical interventions implemented by the government, tended to believe more in conspiracies and had low levels of trust in the sources which provided information about the virus [72].

Another study, which focused on examining the relationship between trust in the healthcare system and people’s choice of seeking medical help when they experienced COVID – 19 symptoms, concluded that high levels of trust in the healthcare system can increase the probability of asking for medical help when people first notice COVID – 19 symptoms [73].

Taking into account the aspects mentioned above, we can infer that peoples’ trust in doctors was affected during the pandemic. In this regard, in the context of misinformation, one of the reasons why people lost trust in doctors may be the fact that, besides using social media for communicating information, for networking or for interacting with patients, many medical or dental practitioners used social media to express their professional opinions about the virus, opinions which were not validated and which later proven to be inaccurate [74]. In other words, health professionals may have contributed to the spread of misinformation, and such behavior can contribute to the decrease of trust in medical processes and in healthcare professionals [75]. Other researchers who focused on examining medical misinformation, found that most doctors (94.2%) stated that patients had medical misinformation, and the subjects about they

had the most inaccurate information were represented by COVID – 19 vaccines, COVID – 19 origin, treatment or essential oils [76]. Furthermore, a previous study discovered that trust in doctors increased with age, and communication difficulties decreased, and that trust in doctors decreased while the level of education and communication difficulties increased [77].

Hence, while acknowledging that the pandemic influenced the trust in medical professionals, another aspect that was negatively influenced was the relationships between doctors and their patients. A study which focused on examining the doctor – patient interaction from the perspective of both groups of people, revealed differences in the respondents' opinions. Thus, most doctors stated that they still make eye contact (72%) and that they still show patients empathy, but only few patients declared that their doctors made eye contact (56,8%) or showed them empathy (43,2%) [78].

~~Methods and materials~~ Materials and methods

Research design

The present study was conducted on Romanian healthcare professionals including doctors, nurses and medical students. The method used is quantitative. The questionnaire was administrated online, the data was collected through the help of Google forms, and was disseminated on groups of healthcare professionals and students on platforms such as Facebook and WhatsApp, during the period April 2021– June 2021. The data we collected was firstly exported to Microsoft Excel, and then it was analyzed with IBM Statistical Package for the Social Sciences, version 20. The respondents were informed about the purpose of the study, about the fact that they were allowed to withdraw at any time, and they were asked to give

their consent for participating in the study. The average time needed to complete the questionnaire was 15 minutes.

Considering the validity of our research, we took into account the theoretical information from the literature regarding the development of a questionnaire. Our team of researchers together with health specialists have configured the dimensions, and operationalized the concepts in accordance with the theoretical approaches identified at the current stage of the research. Even more, we pre-tested the questionnaire before disseminating in order to guarantee the validity of the instrument. Thus, the questionnaire was completed by 50 respondents in the pre-testing stage. Considering the reliability of the research, we used split half reliability method. We split our sample in half, and we checked the variables in from our sub-samples in order to see if the variables provided convergent results. The convergent results we obtained by applying the split half method showed that we obtained a high fidelity measurement.

Formatted: Font: Not Bold

The research instrument

In order to conduct the research we used a quantitative method while having a questionnaire as an instrument. In this regard, we developed a questionnaire which comprises four sections: A. Influence of the pandemic on the professional activity of medical staff (items A1 to A4), B. Perception about the authorities' communication process (items B1 to B11), C. Perception about the communication of non- validated treatments (items C1 to C20), and D. Sociodemographic questions (items D1 – D9), such as: gender, age, living environment, professional degree, field of specialization. The sociodemographic questions were used in order to identify different or similar attitudes between specific groups. The questionnaire can be

Formatted: Justified, Line spacing: Double

found in “S1.Appendix English version of the questionnaire”, and in “S2. Appendix Romanian version of the questionnaire.” Before disseminating the questionnaire, the instrument was tested on 30 doctors who work in the field of cardiology and general medicine. The respondents understood clearly the questions and did not report any issue in the process of answering them. Hence, the questionnaire comprises close ended and open ended questions (Items A1, A4,B3, B11, C19, C20, D2, D5, D6,) dihotomic questions as well as questions whose answers were measured on a 7 point Likert scale. For example, item A2 measured the extent to which the respondents considered that the pandemic influenced the way they carried out their professional activity (1- “to an extremely little extent, 7 “to an extremely great extent”), or item B2 measure the respondents’ level of agreement with statements regarding the way authorities communicated during the pandemic (1 – “strongly disagree, 7-“ strongly agree”).

Formatted: Font: 12 pt, Not Bold, Font color: Text 1

Sampling and data collection procedures

~~The present study was conducted on Romanian healthcare professionals including doctors, nurses and medical students. The questionnaire was administered online, the data was collected through the help of Google forms, and was disseminated on groups of healthcare professionals and students on platforms such as Facebook and WhatsApp, during the period April 2021– June 2021. The data we collected was firstly exported to Microsoft Excel, and then it was analyzed with IBM Statistical Package for the Social Sciences, version 20. At the beginning of the questionnaire, the respondents were informed about the purpose of the study, about the fact that they were allowed to withdraw at any time, and they were asked to give their consent for participating in the study. The average time needed to complete the questionnaire~~

~~was 15 minutes, and the research received approval from The Council of the Faculty of Sociology and Communication, approval request Nr.378/30.03.2021.~~

In order to conduct the research we used a quantitative method while having as an instrument a questionnaire. The responses were collected online, with the help of Google forms, and the questionnaire was self – administrated. The research received approval from The Council of the Faculty of Sociology and Communication, approval request Nr.378/30.03.2021. Taking into account the sampling method and the calculation of the study sample, we used random, probabilistic sampling method. We took into consideration specialists, physicians,and medical students from Brasov, and we applied the snowballing method in order to disseminate the questionnaire. The sample of our study comprises 536 respondents, and includeds doctors, nurses as well as medical students from Romania.~~.~~ ~~Out of the 536 respondents, 460 (85.8%) were female and 76 (14.2%) were male. A total of 411 respondents live in the urban area (76.7%), while 125 (23.3%) live in the rural area. Most respondents (286, 53.4%) are between 18 and 35 years of age, 142 respondents (26.5%) are between 36 and 50 years of age, 102 respondents (19.0%) are between 51 and 65 years of age, and 6 of them (1.1) are over 65 years of age. When it comes to the professional degree of the respondents, most of them are students at a university nursing program (122, 22.8%), and medical students (120, 22.4%). However, a total of 102 respondents (19.0%) are senior specialists medical – doctors, and 70 (13.1%) are nurses who have a higher education diploma. When it comes to the respondents field of specialization, most of them (70.5%) operate in the field of general medicine, while others are family doctors (10.4%), pediatricians (3%), dentists or oncologists (1.9%), surgeons of doctors who are specialized in internal medicine (1.5%), or~~

Formatted: Font: Not Bold

infectious disease doctors, radiologists or cardiologists (1.1%). Furthermore, most of the respondents (77.2%) stated that they did not work a unit with COVID – 19 patients while few of them (22.8%) stated that they worked in such a unit at the time the research was conducted. Thus, all the characteristics of the sample are presented in Table 1.

Table 1. Sample characteristics (n = 536).

	**Category**	**Count**	**Percentage**
Gender	Female	460	88.8%
	Male	76	14.2%
Living environment	Urban	411	76.7%
	Rural	125	23.3%
Age	18-35 years old	286	53.4%
	36-50 years old	142	26.5%
	51–65 years old	102	19.0%
	Over 65 years old	6	1.1%
Professional degree	Senior specialist medical - doctor	102	19.0%
	Specialist medical - doctor	46	8.6%
	Resident	28	5.2%

Formatted: Normal, Justified, Line spacing: Double

Formatted: Normal, Justified, Line spacing: Double
Formatted: Normal, Justified, Line spacing: Double
Formatted: Normal, Justified, Line spacing: Double
Formatted: Normal, Justified, Line spacing: Double
Formatted: Normal, Justified, Line spacing: Double

	~~Nurse with higher education diploma~~	~~70~~	~~13.1%~~
	~~Nurse with other studies than higher education~~	~~48~~	~~9.0%~~
	~~Medical student~~	~~120~~	~~22.4%~~
	~~Student at university nursing program~~	~~122~~	~~22.8%~~
~~Field of specialization~~	~~General medicine~~	~~378~~	~~70.5%~~
	~~Family doctor~~	~~56~~	~~10.4%~~
	~~Pediatrics~~	~~16~~	~~3%~~
	~~Stomatology~~	~~10~~	~~1.9%~~
	~~Oncology~~	~~10~~	~~1.9%~~
	~~Surgery~~	~~8~~	~~1.5%~~
	~~Internal medicine~~	~~8~~	~~1.5%~~
	~~Virology/ infectious disease doctor~~	~~6~~	~~1.1%~~

Formatted: Normal, Justified, Line spacing: Double

Formatted: Normal, Justified, Line spacing: Double

Formatted: Normal, Justified, Line spacing: Double

Formatted: Normal, Justified, Line spacing: Double

Formatted: Normal, Justified, Line spacing: Double

Formatted: Normal, Justified, Line spacing: Double

Formatted: Normal, Justified, Line spacing: Double

Formatted: Normal, Justified, Line spacing: Double

Formatted: Normal, Justified, Line spacing: Double

Formatted: Normal, Justified, Line spacing: Double

Formatted: Normal, Justified, Line spacing: Double

Formatted: Normal, Justified, Line spacing: Double

	Cardiology	6	1.1%
	Radiology	6	1.1%
	Other	32	6%
Works in a unit with COVID – 19 patients	Yes	122	22.8%
	No	414	77.2%

Formatted: Normal, Justified, Line spacing: Double

Formatted: Normal, Justified, Line spacing: Double

Formatted: Normal, Justified, Line spacing: Double

Formatted: Normal, Justified, Line spacing: Double

The research instrument

In order to conduct the research we used a quantitative method while having a questionnaire as an instrument. In this regard, we developed a questionnaire which comprises four sections: A. Influence of the pandemic on the professional activity of medical staff (items A1 to A4), B. Perception about the authorities' communication process (items B1 to B11), C. Perception about the communication of non- validated treatments (items C1 to C20), and D. Sociodemographic questions (items D1 – D9), such as: gender, age, living environment, professional degree, field of specialization. The sociodemographic questions were used in order to identify different or similar attitudes between specific groups. The questionnaire can be found in "S1.Appendix English version of the questionnaire", and in "S2. Appendix Romanian version of the questionnaire." Before disseminating the questionnaire, the instrument was tested on 30 doctors who work in the field of cardiology and general medicine. The

~~respondents understood clearly the questions and did not report any issue in the process of answering them. Hence, the questionnaire comprises close ended and open ended questions (Items A1, A4,B3, B11, C19, C20, D2, D5, D6,) dihotomic questions as well as questions whose answers were measured on a 7 point Likert scale. For example, item A2 measured the extent to which the respondents considered that the pandemic influenced the way they carried out their professional activity (1- "to an extremely little extent, 7 "to an extremely great extent"), or item B2 measure the respondents' level of agreement with statements regarding the way authorities communicated during the pandemic (1 – "strongly disagree, 7 " strongly agree").~~

Data analysis

Data was analyzed with IBM Statistical Package for the Social Sciences, version 20. In order to analyze the data and identify differences and similarities between the attitudes of certain groups, t tests for independent samples were performed. The t test were performed among groups: male/female, working in unit with COVID – 19 patients/ not working in unit with COVID – 19 patients, urban/rural area, and professional degree: medical staff/students. Hence, in order to be able to analyze the results depending on professional degree, we computed the variable of professional degree which had the following values: senior specialist medical – doctor, specialist medical – doctor, resident, nurse with higher education diploma, nurse with other studies than higher education, medical student, student at university nursing program, in a new variable. Thus, doctors, nurses and residents, were integrated in a new group called "medical staff", while medical students and students at university nursing programs were

integrated in the group “students”. Moreover, for a better understanding of the way some variables correlate with each other, (for example: respondents satisfaction with the way authorities communicated during the pandemic and age, respondents’ opinion about the way misinformation about alternative treatments influenced doctors’ credibility and age), we also calculated the Pearson coefficient.

Results

Out of the 536 respondents, 460 (85.8%) were female and 76 (14.2%) were male. A total of 411 respondents live in the urban area (76.7%), while 125 (23.3%) live in the rural area. Most respondents (286, 53.4%) are between 18 and 35 years of age, 142 respondents (26.5%) are between 36 and 50 years of age, 102 respondents (19.0%) are between 51 and 65 years of age, and 6 of them (1.1) are over 65 years of age. When it comes to the professional degree of the respondents, most of them are students at a university nursing program (122, 22.8%), and medical students (120, 22.4%). However, a total of 102 respondents (19.0%) are senior specialists medical – doctors, and 70 (13.1%) are nurses who have a higher education diploma. When it comes to the respondents field of specialization, most of them (70.5%) operate in the field of general medicine, while others are family doctors (10.4%), pediatricians (3%), dentists or oncologists (1.9%), surgeons of doctors who are specialized in internal medicine (1.5%), or infectious disease doctors, radiologists or cardiologists (1.1%). Furthermore, most of the respondents (77.2%) stated that they did not work a unit with COVID – 19 patients while few of them (22.8%) stated that they worked in such a unit at the time the research was conducted. Thus, all the characteristics of the sample are presented in Table 1.

Table 1. Sample characteristics (n = 536).

	Category	Count	Percentage
Gender	Female	460	88.8%
	Male	76	14.2%
Living environment	Urban	411	76.7%
	Rural	125	23.3%
Age	18-35 years old	286	53.4%
	36-50 years old	142	26.5%
	51 -65 years old	102	19.0%
	Over 65 years old	6	1.1%
Professional degree	Senior specialist medical - doctor	102	19.0%
	Specialist medical - doctor	46	8.6%
	Resident	28	5.2%
	Nurse with higher education diploma	70	13.1%
	Nurse with other studies than higher education	48	9.0%
	Medical student	120	22.4%
	Student at university nursing program	122	22.8%
Field of specialization	General medicine	378	70.5%
	Family doctor	56	10.4%
	Pediatrics	16	3%
	Stomatology	10	1.9%
	Oncology	10	1.9%
	Surgery	8	1.5%
	Internal medicine	8	1.5%
	Virology/ infectious disease doctor	6	1.1%
	Cardiology	6	1.1%
	Radiology	6	1.1%
	Other	32	6%
Works in a unit with COVID – 19 patients	Yes	122	22.8%
	No	414	77.2%

1) To what extent information about alternative treatments affected the credibility of medical staff?

The results of our research revealed that respondents were of the opinion that information about alternative treatments for COVID -19 affected the credibility of healthcare professionals. Hence, most respondents (32.5%), stated that trust in healthcare professionals was affected to a an extremely great extent by the information about alternative treatments, many of them declared that credibility was affected to a very great extent (23.1%), and to a great extent (21.3%) (S3 tables with results for 1st research question -Table 2 Table 1).

Formatted: Superscript

Table 1. The extent to which information about alternative treatments affected trust in physicians

		Frequency	Percent	Valid Percent	Cumulative Percent
Valid	to an extremely little extent	14	2.6	2.6	2.6
	to a very little extent	10	1.9	1.9	4.5
	to a little extent	42	7.8	7.8	12.3
	nor to a little, neither to a great extent	58	10.8	10.8	23.1
	to a great extent	114	21.3	21.3	44.4
	to a very great extent	124	23.1	23.1	67.5
	to an extremely great extent	174	32.5	32.5	100.0
	Total	536	100.0	100.0	

Furthermore, the Pearson correlation performed between the extent to which respondents believed that information about alternative treatments affected people's trust in doctors and the age of the respondents, revealed a weak, negative and statistically significant correlation between the two variables (r(534)= -.155, p=0.001) (Table 32). Hence, as the age of

the medical staff decreases, the extent to which they believe the credibility of doctors was affected increases. In other words, compared to older healthcare professionals, younger healthcare professionals tend to believe more that information about alternative treatments affected trust in doctors. One possible explanation for this result can be that younger people tend to be fonder of keeping up with trends and being up to date, and in this context, it is possible that they came into contact more frequently with information about certain alternative treatments for COVID – 19, this making them more aware about the way such treatments can undermine doctor's credibility.

Table 3~~2~~. Pearson correlation between information about alternative treatments and age

		C14. The extent to which information about alternative treatments affected trust in physicians	D2. Age
C14.[1] The extent to which information about alternative treatments affected trust in physicians	Pearson Correlation	1	-.155**
	Sig. (2-tailed)		.000
	N	536	536
D2[2]. Age	Pearson Correlation	-.155**	1
	Sig. (2-tailed)	.000	
	N	536	536

**. Correlation is significant at the 0.01 level (2-tailed).

[1] C14 – refers to the question 14 from the section C of the manuscript (The extent to which information about alternative treatments affected trust in physicians), section which refers to Perception about the communication of non- validated treatments

[2] D2 - refers to question 2 from the D section of the manuscript (age), which refers to Sociodemographic characteristics of the respondents

Formatted: Font: 10 pt

Formatted: Font: 10 pt, Not Superscript/ Subscript

Formatted: Font: 10 pt, Not Superscript/ Subscript

Formatted: Font: 10 pt

In order to observe if there any differences in the opinion of the respondents depending on certain variables including, age, gender, or living environment, we performed t tests for

independent samples. The results of the significant t tests (Table 43), showed that students believed to a greater extent (M= 5.60, SD=1.49), that information about alternative treatments negatively affects the credibility of doctors, than the medical staff (M=5.33, SD=1.54). Also, respondents who declared they worked in a unit without COVID – 19 patients (M=5.53, SD=1.49), were more of the opinion that information about alternative cures affected trust in health professionals, than respondents who worked in a unit with COVID – 19 patients (M=5.19, SD=1.61). One possible explanation would be that, doctors who interacted with COVID – 19 patients may have observed that when being put in the situation to receive medical care in the hospital, patients still had faith and trust in doctors. Moreover, another explanation is that respondents who did not come into contact with COVID – 19 patients were not that close with the situation and thus they might have had a more distorted perception about the situation than those professionals who interacted with COVID – 19 patients. Moreover, the results of the research also showed that female respondents (M=5.51, SD=1.48), believed more than male respondents (M=5.10, SD=1.70), that trust in healthcare professionals was affected by the information about alternative treatments.

Table 43. Significant t-test results: comparisons between variables

					t-test for Equality of Means						
	Group	N	Mean	S. D.	t	df	p	Mean Difference	Std. Error Difference	CI4 Lower	Upper
Variables: Information about alternative treatments _ Professional degree[1]	Medical staff	294	5.33	1.54	-2.04	534	.04	-.27	.13	-.52	-.01
	Student	242	5.60	1.49							

Variables: Information about alternative treatments _working unit	Unit with COVID -19 patients	122	5.19	1.61	-2.13	534	.03	-.33	.15	-.64	-.02
	Unit without COVID 19 patients	414	5.53	1.49							
Variables: Information about alternative treatments _gender	Male	76	5.10	1.70	-2.16	534	.03	-.40	.18	-.77	-.03
	Female	460	5.51	1.48							

[1]Index variable from the professional degrees of respondents. Student: medical student and student at university nursing program, Medical Staff: Senior specialist medical – doctor, Specialist medical – doctor, Resident, Nurse with higher education diploma, Nurse with other studies than higher education

2) What is the knowledge of medical staff about the type of drugs that had positive effects on treating the disease and about alternative treatments?

Considering the type of drugs which were known to have positive effects on treating the virus, the research revealed that type of drug about which the respondents have heard it had positive effects against the virus was Dexamethasone (46.6%), closely followed by Remdesivir (40.5%) and Azithromicin (38.4%). However, some of the respondents also mentioned Chloroquine, Hydroxychloroquine (23.1%), Ibuprofen (19.8%), Tocilizumab (15.9%), and Favipiravir (13.8%) as drugs known to have positive effects when dealing with COVID – 19 (S4 Tables with results to the 2nd research question Table 5~~Table 4~~). Hence, the research showed that the medical staff had knowledge about the type of drugs tested or used against the virus, which were taught to be efficient in treating the disease.

Formatted: Superscript

~~**Table 4.** Drugs known to have positive effects in treating the virus: the perception of medical staff~~

	~~Frequency~~	~~Valid percent~~
~~Amoxicillin~~	~~36~~	~~6.7%~~

Azithromicin	206	38.4%
Chloroquine, Hydroxychloroquine	124	23.1%
Dexamethasone	250	46.6%
Doxycycline	32	6.0%
Favipiravir	74	13.8%
Ibuprofen	106	19.8%
Lopinavir/Ritonavir	56	10.4%
Oseltamivir, Peramivir or Zanamivir	32	6.0%
Remdesivir	217	40.5%
Tocilizumab	85	15.9%
Umifenovir	17	3.2%

In the context of respondents' perception about alternative methods of preventing and treating the virus, the findings show that, most of them stated that they heard about the fact that alcohol consumption can prevent the infection with the virus (24.3%), that drinking warm water every 15 minutes may help eliminate the virus (21.3%), but also that pointing the hot air of the hairdryer to the nostrils leads to the elimination of the virus (16.8%) (S4 tables with results to the 2nd reseach question Table 6Table 5).

Formatted: Superscript

Table 5. Medical staff's knowledge about alternative methods of preventing and treating the virus

		Frequency	Percent	Valid Percent	Cumulative Percent
Valid	drinking alcohol helps you eliminate the virus	79	14.7	14.7	14.7
	drinking alcohol prevents the infection with the virus	130	24.3	24.3	39.0
	rinsing the nostrils with disinfectant eliminates the virus	81	15.1	15.1	54.1
	drinking hot water every 15 minutes eliminates the virus	114	21.3	21.3	75.4

pointing hot air to the nostrils leads to the elimination of the virus	90	16.8	16.8	92.2
other	42	7.8	7.8	100.0
Total	536	100.0	100.0	

3) How satisfied is the medical staff with the way medical and non-medical information was communicated during the pandemic?

The findings of the study revealed that respondents were mostly dissatisfied with the way medical and non – medical information was communicated during the pandemic. Hence, the sum of the responses with negative valences of the study participants (extremely dissatisfied, very dissatisfied and dissatisfied), showed that 238 of them, (44.4%) were dissatisfied with the process of sending medical and non- medical information, while the sum of the positive responses (satisfied, very satisfied, extremely satisfied) showed that 162 of them (30.2%), were satisfied with the communication process (S5 Tables with results to the 3rd research question Table 7 Table 6). In other words, the study highlighted that respondents registered mostly low level of satisfaction with the way information was sent during the pandemic.

Formatted: Superscript

Table 6. The level of satisfaction with the way information about drugs used to treat the virus were communicated at national level

		Frequency	Percent	Valid Percent	Cumulative Percent
Valid	extremely dissatisfied	52	9.7	9.7	9.7
	very dissatisfied	76	14.2	14.2	23.9
	dissatisfied	110	20.5	20.5	44.4
	Nor dissatisfied, neither satisfied	136	25.4	25.4	69.8
	satisfied	108	20.1	20.1	89.9
	very satisfied	30	5.6	5.6	95.5
	Extremely satisfied	24	4.5	4.5	100.0

Total	536	100.0	100.0

Furthermore, in the context of the medical staff's satisfaction with the way information about drugs used to treat the virus was communicated at national level, the research showed that as age of the respondents decreases, the level of satisfaction increases (r(534)= -.091, p=0.035) (Table 87). Thus, according to this result, it can be inferred that younger people were more satisfied than older people, with how information about drugs used to treat the virus was communicated.

Table 87. Pearson Correlation: satisfaction with the way information about drugs used to treat the virus was communicated and age

		B10. Satisfaction with the way information about drugs used to treat the virus was communicated	D2. Age
B10[1]. Satisfaction with the way information about drugs used to treat the virus was communicated	Pearson Correlation	1	-.091*
	Sig. (2-tailed)		.035
	N	536	536
D2[2]. Age	Pearson Correlation	-.091*	1
	Sig. (2-tailed)	.035	
	N	536	536

*. Correlation is significant at the 0.05 level (2-tailed).

[1] B10- refers to the question 10 from the section B of the manuscript (Satisfaction with the way information about drugs used to treat the virus was communicated) section which refers to Perception about the authorities' communication process

[2]D2 - refers to question 2 from the D section of the manuscript (age), which refers to Sociodemographic characteristics of the respondents.

Formatted: Font: 10 pt

Formatted: Font: 10 pt, Superscript

Formatted: Font: 10 pt

Formatted: Font: 10 pt, Superscript

Formatted: Font: 10 pt

Moreover, when asked to evaluate the efficiency of the communication strategies adopted by authorities in order to send information about the virus, most respondents stated that the strategies were effective. Thus, the sum of the responses with negative valences shows

that 144 of them (26, 9%) described the communication strategies as inefficient, while 266 of them (49, 6%) described them as efficient (S5 Tables with results to the 3rd research question Table 9Table 8). One interesting result of the analysis, was that, when trying to examine if the responses of the study participants about the efficiency of communication strategies differ depending on certain variables such as working unit, gender, working unit, living environment, the analysis found no differences between the responses of males and females, of people working in units without COVID – 19 patients and people not working in units with COVID – 19 patients, or in people from the rural and urban area.

Table 8. Perception about the efficiency of communication strategies adopted by authorities

		Frequency	Percent	Valid Percent	Cumulative Percent
Valid	Extremely inefficient	22	4.1	4.1	4.1
	very inefficient	38	7.1	7.1	11.2
	inefficient	84	15.7	15.7	26.9
	nor efficient, neither inefficient	126	23.5	23.5	50.4
	efficient	134	25.0	25.0	75.4
	very efficient	80	14.9	14.9	90.3
	extremely efficient	52	9.7	9.7	100.0
	Total	536	100.0	100.0	

In the context of the information about drugs tested and used in the treatment against COVID – 19, the results showed that students believe to a greater extent that such information was communicated in a coherent manner (M=4.05, SD=1.63), than the medical staff (M=3.79, SD=1.53) (t(534)= -2.05, p<0.05) (Table 109.). Hence, one possible explanation for this result

Formatted: Superscript

Formatted Table

would be that, due the experience and knowledge of the medical staff, people who were already working in the healthcare system, such people have greater expectations from authorities when it comes to sending medical information, than medical students.

Table 109. Significant t test for information about drugs used to treat the virus and professional degree

	t-test for Equality of Means										
	Group	N	Mean	S. D.	t	df	p	Mean Difference	Std. Error Difference	CI4 Lower	Upper
Information about drugs tested and used to treat the disease[1] _ Professional degree[2]	Medical staff	294	3.79	1.53	-2.05	534	.03	-.28	.13	-.55	-.01
	Student	242	4.05	1.63							

[1] The extent to which respondents believe that information about drugs tested and used to treat the virus was communicated in a coherent manner

[2] Index variable from the professional degrees of respondents. Student: medical student and student at university nursing program, Medical Staff: Senior specialist medical – doctor, Specialist medical – doctor, Resident, Nurse with higher education diploma, Nurse with other studies than higher education

(4) What is the perception of medical staff about the role of social media in spreading misinformation about the virus?

The results of the research revealed that respondents were inclined to believe more that social media was a proper environment for spreading fake medical information during the pandemic. By analyzing the information from S6 Tables with results to the 4th research question Table 11Table 10, it can be observed that the sum of the responses with negative valences (4.5%) (to an extremely little extent, to a very little extent and to a little extent) is much lower than the sum of the responses with positive valences (89.9%) (to an extremely great extent, to a very great extent, to a great extent). Hence, most participants of the study believe that social media

Formatted: Superscript

platforms favored the transmission of fake medical news during the pandemic. Furthermore, when trying to find differences in the responses of the participants depending on age, gender, living environment, professional degree or working unit (with COVID – 19 patients or without COVID – 19 patients), we observed that their responses did not differ depending on such variables. Thus, it can be inferred that, regardless of age, gender, living environment, professional degree or working unit, respondents' perception was that social media had a role in spreading fake medical information.

~~**Table 10.** Perception about the extent to which social media contributed to the spread of medical fake news~~

		Frequency	Percent	Valid Percent	Cumulative Percent
Valid	to an extremely little extent	2	.4	.4	.4
	to a very little extent	10	1.9	1.9	2.2
	to a little extent	12	2.2	2.2	4.5
	nor to a little, neither to a great extent	30	5.6	5.6	10.1
	to a great extent	62	11.6	11.6	21.6
	to a very great extent	88	16.4	16.4	38.1
	to an extremely great extent	332	61.9	61.9	100.0
	Total	536	100.0	100.0	

However, even though respondents were of the opinion that social media was an environment in which was sent fake medical information, some of them still believe that social media platforms are appropriate for sending official information about the virus. Thus, considering the results from S6 Tables with results to the 4th research question Table 12 ~~Table 11~~, the sum of responses with positive valences (40.3%) is almost equal to the sum of responses with negative valences (45.1%) meaning that the opinions of the study participants were divided when it comes to sending official information about the virus on social media.

~~**Table 11.** Perception about the extent to which social media represents an appropriate environment for sharing official COVID – 19 information~~

		~~Frequency~~	~~Percent~~	~~Valid Percent~~	~~Cumulative Percent~~
~~Valid~~	~~to an extremely little extent~~	~~86~~	~~16.0~~	~~16.0~~	~~16.0~~
	~~to a very little extent~~	~~78~~	~~14.6~~	~~14.6~~	~~30.6~~
	~~to a little extent~~	~~52~~	~~9.7~~	~~9.7~~	~~40.3~~
	~~nor to a little, neither to a great extent~~	~~78~~	~~14.6~~	~~14.6~~	~~54.9~~
	~~to a great extent~~	~~72~~	~~13.4~~	~~13.4~~	~~68.3~~
	~~to a very great extent~~	~~74~~	~~13.8~~	~~13.8~~	~~82.1~~
	~~to an extremely great extent~~	~~96~~	~~17.9~~	~~17.9~~	~~100.0~~
	~~Total~~	~~536~~	~~100.0~~	~~100.0~~	

A factor which showed a weak but statistically significant influence on respondents' opinion about sending COVID – 19 official information on social media was age. Hence, the results of the Pearson correlation (r (534) = -.175, p=0.000), showed that as age decreases, the extent to which respondents believed that social media is an environment in which official information about the virus should be communicated decreases (Table 13~~12~~). In other words, younger respondents believed to a greater extent than older respondents that official information should also be communicated on social media. One possible explanation for this results would be that young people gather most of their information from online sources, and they also engage more with social media platforms, and thus it is possible that they would also like to see official and important information on such platforms.

Table 13~~2~~. Person correlation between the extent to which social media represents an appropriate environment for sharing official COVID – 19 info and age

	C1. The extent to which social media represents an appropriate environment for sharing official COVID – 19 info	D2. Age

C1[1]. The extent to which social media represents an appropriate environment for sharing official COVID – 19 info	Pearson Correlation	1	-.175**
	Sig. (2-tailed)		.000
	N	536	536
D2[2]. Age	Pearson Correlation	-.175**	1
	Sig. (2-tailed)	.000	
	N	536	536

**. Correlation is significant at the 0.01 level (2-tailed).

[1] C1 – refers to question 1 from the section C of the manuscript (The extent to which social media represents an appropriate environment for sharing official COVID – 19 info), section which refers to Perception about the communication of non- validated treatments

[2] D2 - refers to question 2 from the D section of the manuscript (age), which refers to Sociodemographic characteristics of the respondents.

Formatted: Font: 10 pt

Formatted: Font: 10 pt, Superscript

Formatted: Font: 10 pt

Formatted: Font: 10 pt, Superscript

Formatted: Font: 10 pt

Furthermore, when dividing the study participants in medical staff (doctors, nurses) and students (medical students or students at the university nursing programs), we found that students (M=4.31, SD=2.11) believed to a greater extent than the medical staff (M= 3.88, SD=2.07) that official information about the virus should also be sent on social media ($t(534) = -2.36$, $p < 0.05$) (Table 13). Next, when dividing the sample by living environment, participants living in the urban area (M=4.19, SD=2.10) were inclined more than those living in the rural area (M=3.72, SD=2.05), to believe that official information could also be sent on social media ($t(534) = 2.23$, $p < 0.05$) (Table 14~~3~~).

Table 14~~3~~. Significant t tests for sharing official information on social media professional degree and living environment

					t-test for Equality of Means					
	Group	N	Mean	S. D.	t	df	p	Mean Difference	Std. Error Difference	CI4 Lower	CI4 Upper
Official information	Medical staff	294	3.88	2.07	-2.36	534	.01	-.42	.18	-.78	-.07

on social media _ Professional degree[1]	Student	242	4.31	2.11							
Official information on social media _living environment	Urban area	411	4.19	2.10	2.23	534	.02	.47	.21	.05	.89
	Rural area	125	3.71	2.05							

[1]Index variable from the professional degrees of respondents. Student: medical student and student at university nursing program, Medical Staff: Senior specialist medical – doctor, Specialist medical – doctor, Resident, Nurse with higher education diploma, Nurse with other studies than higher education

(5) What aspects of the professional activity of the medical staff were affected most by the COVID – 19 pandemic?

The findings of our research showed that most respondents stated that the patient – doctor relationship was most affected by the pandemic (38.4%). However, a smaller percent of respondents declared that the working schedule was the most affected (26.9%), or the collaboration with their peers (23.9%) (S7 Tables with results to the 5th research question Table 15 ~~Table 14~~).

~~Table 14. The aspect of professional life which was most influenced by the pandemic~~

		~~Frequency~~	~~Percent~~	~~Valid Percent~~	~~Cumulative Percent~~
~~Valid~~	~~patient – doctor relationship~~	~~206~~	~~38.4~~	~~38.4~~	~~38.4~~
	~~work schedule~~	~~144~~	~~26.9~~	~~26.9~~	~~65.3~~
	~~collaboration with peers~~	~~128~~	~~23.9~~	~~23.9~~	~~89.2~~
	~~other~~	~~58~~	~~10.8~~	~~10.8~~	~~100.0~~
	~~Total~~	~~536~~	~~100.0~~	~~100.0~~	

Furthermore, taking into account the group of medical staff (doctors, nurses) and the group of students (medical students and students at university nursing program), the results revealed that the most respondents who stated that the patient- doctor relationship was affected most

by the pandemic was the group of medical staff (144 compared to 62) (S7 Tables with results to the 5th research question Table 16 Table 15). One possible explanation for this result is that, by being in constant contact with their patients, doctors and nurses were more inclined to perceive that the relation with their patients has deteriorated during the pandemic.

Table 15. Main aspect of professional life influenced by the pandemic * professional degree - Cross tabulation

		Professional degree[1]		Total
		Medical staff	Student	
A3. Main aspect of professional life influenced by the pandemic	patient – doctor relationship	144	62	206
	work schedule	70	74	144
	collaboration with peers	62	66	128
	other	18	40	58
Total		294	242	536

[1]Index variable from the professional degrees of respondents. Student: medical student and student at university nursing program, Medical Staff: Senior specialist medical – doctor, Specialist medical – doctor, Resident, Nurse with higher education diploma, Nurse with other studies than higher education

Discussion

During the COVID – 19 pandemic, one of the major issues people had to face, was the spread of misinformation about the virus, its origins and its treatment. In this regard, we analyzed the perception of medical staff (including doctors, nurses, medical students and students in the university nursing program) about the way medical and non – medical information was communicated during the pandemic. In the context of the so called infodemic [11], and the effects of misinformation on people's trust in doctors, most participants of our study declared that the information about alternative treatments for the virus affected the credibility of health

professionals. Hence, from this point of view, our study is in line with previous studies which highlighted the fact that lately, trust in physician decreased [67], and which suggested that social media managed to determine people to trust the personal opinions of other people rather than the opinion of the professionals [61]. Furthermore, since other researchers pointed out that many medical practitioners used social media to express professional opinions that were later found inaccurate [74], and thus they may have contributed to the spread of misinformation [75], we argue that the credibility of physicians might have also been affected by this type of behavior.

An interesting result of our research showed that as the age of medical staff decreases, the extent to which they believe that information about alternative treatments affects doctors' credibility increases. Hence, younger healthcare professionals believed to a greater extent than older healthcare professionals, that information about alternative treatments affected negatively people's trust in doctors. This results might have as possible explanation, the fact that younger people tend to spend more time on social media platforms, and they may have interacted more than older professionals, with misinformation about the virus, this making them more able to be aware of the negative effects of fake news. Moreover, the type of unit in which the respondents worked, was a factor which influenced the opinion of the respondents, our findings showing that, the medical staff who did not work in unit with COVID -19 patients, believed to a greater extent than those who worked in such units, that information about alternative treatments negatively influenced doctors' credibility. Given this result we argue that is it possible for those professionals who did not interact with COVID -19 patients, and who thus

were more distant from the situation, to have a more distorted image regarding the way people's levels of trust in them changed in the context of the pandemic.

Considering the role of social media in spreading misinformation, our study is in line with previous studies which support the idea that such channels favored the communication of fake news during the pandemic [49, 50, 51]. In this regard, regardless of age, professional degree or living environment, most healthcare professionals who participated in our study were of the opinion that social media contributed to the spread of misinformation. However, our study also showed that when it comes to communicating official information on social media, younger respondents (students) believed to a greater extent than older respondents (doctors, nurses), that such channels should be used to send official information about the virus. Taking into account these results, the fact that healthcare professionals acknowledge that social media favors the spread of misinformation, and that many of them still believe they should be used in order to communicate official information, shows that at personal level, professionals were not affected that much by misinformation, them being able to differentiate more easily between real and fake news. In other words, we argue that while people in general were negatively influenced by the fake news they read on social media, as it was shown in previous studies which highlighted that people trusted the information on social networks, they shared un-validated information and had trouble with differentiating real from fake news [57, 79] or that exposure to health misinformation may influence people's intention to engage in certain behaviors [80], healthcare professionals may be less influenced by fake news, due to their knowledge.

Considering the knowledge of medical staff about the type of drugs that had positive effects on treating the virus, the findings of the research showed that the respondents had opinions which were in line with the results found in other studies. Hence, according to the research, most respondents stated that the drug which was known to have positive effects against the virus was Dexamethasone (46.6%), it being followed by Remdesivir (40.5%). Thus, positive effects of Dexamethasone were also highlighted by studies [31, 32], while study [35] showed positive effects of Remdesivir. Moreover, during the period in which we conducted our research, (April – June 2021), among the drugs which were approved for administration against the virus were Remdesivir, Tocilizumab – which was authorized first in June 2021, drugs which were also acknowledged by the respondents of our research. Even more, one of the authors of the article (L.R.) is a doctor and was directly involved in the process of taking care of COVID – 19 patients, so the author can confirm that among the drugs which were in trial, or which were approved for administration against COVID-19 were also the drugs which were acknowledged by the respondents of our research.

In the context of medical staff's knowledge about alternative treatments, most respondents declared they had heard about the fact that alcohol can prevent the infection, that warm water drunk every 15 minutes, and the hot air from the hairdryer pointed to the nostrils can help eliminate the virus. From this point of view, our study is in line with a previous study [53], which also described these methods.

When it comes to the respondents' level of satisfaction about the way medical and non – medical information was communicated during the pandemic, generally, the research revealed that most respondents were dissatisfied with the communication process. In the case

of communication strategies adopted by authorities, the results showed that most respondents were satisfied with them. However, in the context of sending information about the drugs used to treat the disease, the research showed that younger healthcare professionals were more satisfied with the communication process than older healthcare professionals. This results might be due to the fact that physicians with more experience have higher expectations from authorities than students.

Another area on which we focused our research was the professional activity of the medical staff during the pandemic. In this regard, our findings revealed that, according to the respondents of our study, the aspect that was mostly affected by the pandemic was the doctor-patient relationship. Hence, our research is in line with other studies [78], which showed that the pandemic affected the way doctors interacted with their patients.

Furthermore, on the basis of the results of our study we argue that not only the process of vaccination created ethical issues, but also the process of communication [81]. Thus, these ethical issues were perceived by the medical staff and they would require a further examination in order to be able to create communication guides which can be regarded as essential instruments not only for the research process of the medical staff and healthcare professionals with management positions, but also for their current medical activity [82,83].

Conclusions

During the pandemic, healthcare professionals did not have to deal only with challenges regarding their health and the health of their patients, but also with the problems created by the spread of medical misinformation~~. In this regard, besides fighting the pandemic, physician also had to fight the so called infodemic. Fake news spread on social media about various~~

~~alternative treatments for the virus and the opinions of certain professionals about treatment methods which later proven to be inaccurate negatively influenced the credibility of doctors.~~ A~~Hence, a~~ccording to the main findings~~results~~ of our research, generally, the medical staff (doctors, nurses, medical students, students at university nursing program), believed that information about alternative treatments affected people's trust in doctors, but younger healthcare professionals and those working in units without COVID - 19 patients believed to a greater extent than older healthcare professionals and people working in units with COVID – 19 patients that fake news about treatments for the virus affected the credibility of doctors.

Furthermore, regardless of age, age, gender, living environment, professional degree or working unit, the medical staff acknowledged the role of social media in spreading fake news, but when it comes to using social media in order to communicate official information, younger healthcare professionals were more inclined to believe that such networks were appropriate for the communication of official information. ~~This results can suggest that while professionals were aware of the role of social media in spreading medical misinformation and in affecting trust in doctors, due to their knowledge, at personal level they were less affected by that type of information, many of them believing that social media should also be used for sending official information.~~

In the context of the drugs used to treat the virus, the results pointed out that the medical staff had knowledge about the drugs known to have positive effects in treating the virus, their perception being in line with previous studies which focused on this matter. ~~Moreover, the medical staff was aware of the alternative treatments which were promoted on~~

~~social media, the method of drinking alcohol in order to prevent the infection being the method that most of the respondents have heard about.~~

When it comes to the influence of the pandemic on the professional activity of the medical staff, the respondents declared that the aspect which was most affected was the doctor – patient relationship. In this regard, we argue that, by influencing peoples' trust in doctors, the medical fake news spread during the pandemic, implicitly had a role in deteriorating the relation between doctors and their patients.

Therefore, the healthcare professionals were generally dissatisfied with the way medical and non – medical information was communicated during the pandemic, but younger professionals were satisfied than older professionals. Overall, the medical staff believed that fake news managed to undermine doctors' credibility that social media platforms favor the spread of such news, and they had knowledge about the drugs which were known to have positive effects on the virus and about the alternative treatments.

Taking into account the results of the research, the paper has some theoretical and practical implications. From a theoretical point of view, the paper contributes to the literature on the matter of fake news and its influence on the trust of healthcare professionals, a strength of the paper being the fact that it analyzed the opinions of medical staff (doctors, nurses, medical students and students at university nursing program). From a practical point of view, the paper brings awareness to the phenomenon of fake news regarding medical treatments and the negative influence it has on doctors' credibility. Another practical implication refers to the fact that the paper brings attention to the issue of using social media as a mean to communicate official information, many healthcare professionals, especially the younger ones,

stating that such networks could be appropriate for sharing official information. Furthermore, by highlighting that the most affected aspect of the professional activity of doctors was the relationship with their patients, the study also shows that actions need to be taken in order to restore people's trust in doctors and improve the process of communication between them. Hence, on the basis of the findings and implications of the study, we further discuss limitations and future research directions.

Limitations and future research directions

While our study proved relevant information regarding the perception of healthcare professionals about the way medical and non – medical information was communicated in time of the pandemic, it also has some limitations.

One limitation is represented by the fact that the perception of healthcare professionals was studied only by using quantitative methods. In this regard, a future research should focus on obtaining information from doctors while using qualitative methods too. Next, the study was conducted only on Romanian healthcare professionals, and thus, a future research should take into consideration a comparison between the opinions of professionals from different countries. Another limitation is represented by the fact that we only asked respondents to state the aspect which was most influenced by the pandemic, but we did not asked them to offer detail about other type of challenges encountered. Thus, a future research should focus on analyzing the extent to which aspects of the professional activity of doctors were affected, and on analyzing more deeply the challenges they had to face during the pandemic.

Furthermore, since our research revealed that many respondents believed that social media platforms could be appropriate for sharing official information, we draw attention to a problem that can arise in this context. Since people know that such platforms favor the spread of fake news, if we encourage the use of social media in order to communicate official information, don't we risk to discredit that information as it is possible for people to consider that such information is fake too? We believe that this issue should be taken into account and studied in a future research.

Author Contributions

Conceptualization: Claudiu Coman, Maria Cristina Bularca

Data curation: Claudiu Coman, Liliana Rogozea, Angela Repanovici, Maria Cristina Bularca

Formal analysis: Maria Cristina Bularca, Claudiu Coman

Investigation: Claudiu Coman, Maria Cristina Bularca, Angela Repanovici, Liliana Rogozea

Methodology: Maria Cristina Bularca, Claudiu Coman

Project administration: Claudiu Coman, Liliana Rogozea, Angela Repanovici

Resources: Maria Cristina Bularca, Liliana Rogozea, Angela Repanovici

Supervision: Claudiu Coman, Liliana Rogozea, Angela Repanovici

Writing – original draft: Maria Cristina Bularca, Claudiu Coman

Writing – review & editing: Maria Cristina Bularca, Claudiu Coman, Liliana Rogozea, Angela Repanovici

References

1. Sanders JM, Monogue ML, Jodlowski TZ, Cutrell J. B.Pharmacologic Treatments for Coronavirus Disease 2019 (COVID-19): A Review. JAMA. 2020; 323(18):1824–1836. doi:10.1001/jama.2020.6019

2. Shereen MA, Khan S, Kazmi A, Bashir N, Siddique R. COVID-19 infection: Origin, transmission, and characteristics of human coronaviruses. J Adv Res. 2020; 24:91-98 doi: 10.1016/j.jare.2020.03.005

3. Kristina SA, Herliana N, Hanifah S. The perception of role and responsibilities during covid-19 pandemic: A survey from Indonesian pharmacists. Int J Pharm Res , 2020; *12*(2). doi: 10.31838/ijpr/2020.SP2.369

4. World Health Organization (WHO). Timeline of WHO's response to COVID-19. [Internet]. World Health Organization. 2020 June 29 [cited 2020 Nov 27] Available from: https://www.who.int/news/item/29-06-2020-covidtimeline

5. European Centre for Disease Prevention and Control. COVID-19 situation update worldwide, as of 27 November 2020 [Internet]. European Centre for Disease Prevention and Control. 2020 June 29 [cited 2021 Nov 25] Available from: https://www.ecdc.europa.eu/en/geographical-distribution-2019-ncov-cases.

6. British Broadcasting Corporation. COVID vaccine: First 'milestone' vaccine offers 90% protection. [Internet]. 2020 Nov 9 [cited 2020 Nov 27] Available from: https://www.bbc.com/news/health-54873105.

7. Kommenda N, Jones FH. COVID vaccine tracker: when will a coronavirus vaccine be ready? [Internet]. The Guardian. 2020 Nov 10 [cited 2020 Nov 27] Available from: https://www.theguardian.com/world/ng-interactive/2020/nov/10/covid-vaccine-tracker-when-will-a-coronavirus-vaccine-be-ready

8. Ahsan W, Javed S, Al Bratty M, Alhazmi HA, Najmi A. Treatment of SARS-CoV-2: How far have we reached? *Drug Discov Ther*. 2020; 14(2):67-72. doi: 10.5582/ddt.2020.03008

9. Kupferschmidt K, Cohen J. Race to find COVID-19 treatments accelerates. *Science*. 2020; 367(6485): 1412-1413. doi: 10.1126/science.367.6485.1412

10. World Health Organization (WHO). Solidarity clinical trial for COVID-19 treatments [Internet]. World Health Organization 2019. [cited 2020 Nov 27] Available from: https://www.who.int/emergencies/diseases/novel-coronavirus-2019/global-research-on-novel-coronavirus-2019-ncov/solidarity-clinical-trial-for-covid-19-treatments

11. Zarocostas J. How to fight an infodemic. *The Lancet*. 2020; *395*(10225):676. doi: 10.1016/S0140-6736(20)30461-X

12. Tuccori M, Convertino I, Ferraro S, Cappello E, Valdiserra G, Focosi D et al. The Impact of the COVID-19 "Infodemic" on Drug-Utilization Behaviors: Implications for Pharmacovigilance. *Drug Saf*. 2020;43:699–709 doi:10.1007/s40264-020-00965-w

13. Rosa SGV, Santos WC. Clinical trials on drug repositioning for COVID-19 treatment. *Rev Panam Salud Publica*. 2020; 44: e40 doi: 10.26633/RPSP.2020.40

14. Sahraei Z, Shabani M, Shokouhi S, Saffaei A. Aminoquinolines against coronavirus disease 2019 (COVID-19): chloroquine or hydroxychloroquine. *Int J Antimicrob Agents*. 2020; *105945*(10.1016) doi:10.1016/j.ijantimicag.2020.105945

15. White NJ, Watson JA, Hoglund RM, Chan XHS, Cheah PY, Tarning J. COVID-19 prevention and treatment: A critical analysis of chloroquine and hydroxychloroquine clinical pharmacology. PLoS Med. 2020; 17(9): e1003252. doi:10.1371/journal.pmed.1003252

16. Liu J, Cao R, Xu M, Wang X, Zhang H, Hu H. et al. Hydroxychloroquine, a less toxic derivative of chloroquine, is effective in inhibiting SARS-CoV-2 infection in vitro. Cell Discov. 2020; 6(16) doi:10.1038/s41421-020-0156-0 .

17. Wang M, Cao R, Zhang L, Yang X, Liu J, Xu M. et.al. Remdesivir and chloroquine effectively inhibit the recently emerged novel coronavirus (2019-nCoV) in vitro. *Cell Res*. 2020; 30:269–271. doi:10.1038/s41422-020-0282-0

18. Davidescu EI, Odajiu I, Bunea T, Sandu G, Stratan L, Aramă V. et.al. Treatment with hydroxychloroquine in patients with covid-19. Experience of a neurology department. *Farmacia*. 2020; *68*(4): 597-605. doi:10.31925/farmacia.2020.4.3

19. Gautret P, Lagier JC, Parola P, Meddeb L, Mailhe M, Doudier B. et.al. Hydroxychloroquine and azithromycin as a treatment of COVID-19: results of an open-label non-randomized clinical trial. *Int. J. Antimicrob. Agents*. 2020; 105949. doi:10.1016/j.ijantimicag.2020.105949.

20. Thomson K, Nachlis H. Emergency Use Authorizations During the COVID-19 Pandemic: Lessons From Hydroxychloroquine for Vaccine Authorization and Approval. *JAMA.* 2020; 324(13):1282–1283. doi:10.1001/jama.2020.16253.

21. U.S Food & Drug Administration. FDA cautions against use of hydroxychloroquine or chloroquine for COVID-19 outside of the hospital setting or a clinical trial due to risk of heart rhythm problems. [Internet]. Food and Drug Administration. 2020 Jul 1 [cited 2020 Nov 27] Available from: https://www.fda.gov/drugs/drug-safety-and-availability/fda-cautions-against-use-hydroxychloroquine-or-chloroquine-covid-19-outside-hospital-setting-or .

22. Recovery. No clinical benefit from use of hydroxychloroquine in hospitalized patients with COVID-19. [Internet]. Recovery 2020 June 5 [cited 2020 Nov 27] Available from: https://www.recoverytrial.net/news/statement-from-the-chief-investigators-of-the-randomised-evaluation-of-covid-19-therapy-recovery-trial-on-hydroxychloroquine-5-june-2020-no-clinical-benefit-from-use-of-hydroxychloroquine-in-hospitalised-patients-with-covid-19 .

23. Naveed M, Uddin S, Abdullah KS, Ishaq SE, Ahmad T. Various Evidence-Based Hypothetical and Experimental Treatment Approaches and Their Effectiveness against COVID-19 Worldwide: A Comprehensive Literature Review. *EJMO* 2020; 4(4):265–285. doi: 10.14744/ejmo.2020.52538

24. Chu C M, Cheng VCC, Hung IFN, Wong MML, Chan KH, Chan KS, et.al. Role of lopinavir/ritonavir in the treatment of SARS: initial virological and clinical findings. *Thorax*. 2004; 59(3):252-256. doi:10.1136/thorax.2003.012658

25. Cao B, Wang Y, Wen D, Liu W, Wang J, Fan G, et al. A trial of lopinavir-ritonavir in adults hospitalized with severe covid-19. *N. Engl . J. Med.* 2020; 382(19): 1787-1799. doi:10.1056/NEJMoa2001282

26. Vosu J, Britton P, Howard-Jones A, Isaacs D, Kesson A, Khatami A, et al. Is the risk of ibuprofen or other non-steroidal anti-inflammatory drugs increased in COVID-19?. *J Paediatr Child Health*. 2020; *56*(10): 1645-1646. doi:10.1111/jpc.15159

27. Fang L, Karakiulakis G, Roth M. Are patients with hypertension and diabetes mellitus at increased risk for COVID-19 infection? *Lancet Respir Med*. 2020; 8(4):e21.8 doi:10.1016/S2213-2600(20)30116-8

28. Science alert. Updated: WHO Now Doesn't Recommend Avoiding Ibuprofen For COVID-19 Symptoms [Internet]. Science alert 2020 Mar 17 [cited 2020 Nov 27]. Available from: https://www.sciencealert.com/who-recommends-to-avoid-taking-ibuprofen-for-covid-19-symptoms

29. Esba LCA, Alqahtani RA, Thomas A, Shamas N, Alswaidan L, Mardawi G. Ibuprofen and NSAID Use in COVID-19 Infected Patients Is Not Associated with Worse Outcomes: A Prospective Cohort Study. *Infect Dis Ther*. 2020; 1-16. doi:10.1007/s40121-020-00363-w

30. Rinott E, Kozer E, Shapira Y, Bar-Haim A, Youngster I. Ibuprofen use and clinical outcomes in COVID-19 patients. *Clin Microbiol Infect*. 2020; 26(9):1259.e5-1259.e7. doi:10.1016/j.cmi.2020.06.003

31. Roberts M. Coronavirus: Dexamethasone proves first life-saving drug. [Internet]. British Broadcasting Corporation 2020 June 16 [cited 2020 Nov 27] Available from: https://www.bbc.com/news/health-53061281.

32. Horby P, Lim WS, Emberson JR, Mafham M, Bell JL, Linsell L, et al.. Dexamethasone in hospitalized patients with Covid-19-preliminary report. *N. Engl. J. Med*. 2020. doi:10.1056/nejmoa2021436

33. Lu CC, Chen MY, Lee WS, Chang YL. Potential therapeutic agents against COVID-19: What we know so far. *J Chin Med Assoc*. 2020; 83(6):534-536. doi: 10.1097/JCMA.0000000000000318

34. Wang Y, Zhang D, Du G, Du R, Zhao J, Jin Y, et al. Remdesivir in adults with severe COVID-19: a randomised, double-blind, placebo-controlled, multicentre trial. *The Lancet*. 2020; 395(10236):1569-1578. doi:10.1016/S0140-6736(20)31022-9

35. Beigel JH, Tomashek KM, Dodd LE, Mehta AK, Zingman BS, Kalil AC. et al. Remdesivir for the treatment of Covid-19. *N. Engl. J. Med*. 2020. https://www.nejm.org/doi/10.1056/NEJMoa2007764

36. U.S Food and Drug Administration. FDA Approves First Treatment for COVID-19 [Internet]. Food and Drug Administration 2020 Oct 22 [cited 2020 Nov 27] Available from: https://www.fda.gov/news-events/press-announcements/fda-approves-first-treatment-covid-19.

37. U.S Food and Drug Administration. Coronavirus (COVID-19) Update: November 20, 2020 [Internet]. Food and Drug Administration 2020 Nov 20 [cited 2020 Nov 27] Available from: https://www.fda.gov/news-events/press-announcements/coronavirus-covid-19-update-november-20-2020.

38. Glasdam S, Stjernswärd S. Information about the COVID-19 pandemic–a thematic analysis of different ways of perceiving true and untrue information. *SSHOP.* 2020; (2)1: 100090. https://doi.org/10.1016/j.ssaho.2020.100090

39. Caleb TC, Hayes RA. Social Media: Defining, Developing, and Divining. *Atl J Commun.*2015; 23:1: 46-65. https://doi.org/10.1080/15456870.2015.972282

40. Boyd DM, Ellison NB.Social network sites: Definition, history, and scholarship. *J Comput Mediat Commun.* 2007; *13*(1): 210-230. https://doi.org/10.1111/j.1083-6101.2007.00393.x

41. Reuter C, Stieglitz S, Imran M.Social media in conflicts and crisesBehav. *Inf. Technol*. 2020; *39*(3): 241-251. https://doi.org/10.1080/0144929X.2019.1629025

42. Vasconcellos-Silva PR, Castiel LD. COVID-19, fake news, and the sleep of communicative reason producing monsters: the narrative of risks and the risks of narratives. *Cad Saude Publica*. 2020); *36*(7): e00101920. https://doi.org/10.1590/0102-311x00101920

43. Pulido CM, Ruiz-Eugeni L, Redondo-Sama G, Villarejo-Carballido B. A New Application of Social Impact in Social Media for Overcoming Fake News in Health. *Int. J. Environ. Res. Public Health*. 2020; *17*(7):2430. **https://doi.org/10.3390/ijerph17072430**

44. Al-Dmour H, Salman A, Abuhashesh M, Al-Dmour R.Influence of social media platforms on public health protection against the COVID-19 pandemic via the mediating effects of public health awareness and behavioral changes: integrated model. *J. Medical Internet Res*. 2020; *22*(8):e19996.

45. Wong JEL, Leo YS, Tan CC. COVID-19 in Singapore—Current Experience: Critical Global Issues That Require Attention and Action. *JAMA.* 2020; 323(13):1243–1244. doi:10.1001/jama.2020.2467

46. Lazer DM, Baum MA, Benkler Y, Berinsky AJ, Greenhill KM, Menczer F, et al. The science of fake news. *Science*. 2018; *359*(6380):1094-1096. DOI: 10.1126/science.aao2998

47. Moscadelli A, Albora G, Biamonte MA, Giorgetti D, Innocenzio M, Paoli S, et al. Fake News and Covid-19 in Italy: Results of a Quantitative Observational Study. *Int. J. Environ. Res. Public Health.* 2020; *17*:5850

48. Mian A, Khan S. Coronavirus: The spread of misinformation. *BMC Medicine*. 2020; 18(1):1-2 doi:10.1186/s12916-020-01556-3 \

49. Pan American Health Organization. Understanding the infodemic and misinformation in the fight against covid-19. [Internet]. Pan American Health Organization 2020 May 1 [cited 2020 Nov 27] Available from: https://www.paho.org/en/documents/understanding-infodemic-and-misinformation-fight-against-covid-19.

50. Bowles J, Larreguy H, Liu, S. Countering misinformation via WhatsApp: Preliminary evidence from the COVID-19 pandemic in Zimbabwe. *PloS one*. 2020; *15*(10): e0240005. https://doi.org/10.1371/journal.pone.0240005

51. Ittefaq M, Hussain SA, Fatima M. COVID-19 and social-politics of medical misinformation on social media in Pakistan. *Media Asia*. 2020; *47*(1-2): 75-80. https://doi.org/10.1080/01296612.2020.1817264

52. The National Law Review. There's a Fake News Pandemic. Could COVID-19 and Trademarks be the Cure? [Internet]. The National Law Review 2020 Jul 7 [cited 2020 Nov 27] Available from: https://www.natlawreview.com/article/there-s-fake-news-pandemic-could-covid-19-and-trademarks-be-cure

53. O'connor C, Murphy M. Going Viral: Doctors Must Combat Fake News in the Fight against Covid-19. *Ir Med J*. 2020; 113(5): 85-85.

54. Pew Research Center. Nearly three-in-ten Americans believe COVID-19 was made in a lab. [Internet]. Pew research center 2020 April 8 [cited 2020 Nov 27] Available from: https://www.pewresearch.org/fact-tank/2020/04/08/nearly-three-in-ten-americans-believe-covid-19-was-made-in-a-lab/

55. Health Analytics Asia. 50 Fake 'frequently forwarded' COVID-19 WhatsApp messages. [Internet]. Health Analytics Asia 2020 April 2 [cited 2020 Nov 27] Available from: https://www.ha-asia.com/50-fake-frequently-forwarded-covid-19-whatsapp-messages/

56. Brennen JS, Simon F, Howard PN, Nielsen RK. Types, sources, and claims of COVID-19 misinformation. *Reuters Institute*. 2020; *7*: 1-13.

57. Pennycook G, McPhetres J, Zhang Y, Lu JG, Rand DG. Fighting COVID-19 misinformation on social media: Experimental evidence for a scalable accuracy-nudge intervention. *Psychol*. 2020; *31*(7): 770-780. https://doi.org/10.1177/0956797620939054

58. Barua Z, Barua S, Aktar S, Kabir N, Li M. Effects of misinformation on COVID-19 individual responses and recommendations for resilience of disastrous consequences of misinformation. *Prog Disaster Science*. 2020; *8*: 100119. doi:10.1016/j.pdisas.2020.100119

59. Ahmed N, Shahbaz T, Shamim A, Khan KS, Hussain SM, Usman A. The COVID-19 Infodemic: A Quantitative Analysis Through Facebook. *Cureus.* 2020; 12(11): e11346. doi: 10.7759/cureus.11346

60. Nagler RH, Vogel RI, Gollust SE, Rothman AJ, Fowler EF, Yzer MC. Public perceptions of conflicting information surrounding COVID-19: Results from a nationally representative survey of US adults. *PloS one*. 2020; *15*(10): e0240776. doi:10.1371/journal.pone.0240776

61. Orso D, Federici N, Copetti R, Vetrugno L, Bove T. Infodemic and the spread of fake news in the COVID-19-era. *Eur J Emerg Med*. 2020; 27(5):327-328. doi:10.1097/MEJ.0000000000000713

62. McNab C. What social media offers to health professionals and citizens. *Bull World Health Organ*. 2009; 87(8):566. doi:10.2471/blt.09.066712

63. Ma X, Vervoort D, Luc JG. When misinformation goes viral: access to evidence-based information in the COVID-19 pandemic. *J. Glob. Health. Sci*. 2020; *2*(1):e13 doi:10.35500/jghs.2020.2.e13

64. Tasnim S, Hossain MM, Mazumder H. Impact of rumors and misinformation on COVID-19 in social media. *J Prev Med Public Health*. 2020; *53*(3):171-174.

65. Bode L, Vraga EK.See something, say something: Correction of global health misinformation on social media. J Health Commu. 2018; *33*(9):1131-1140. doi:10.1080/10410236.2017.1331312

66. Chou WYS, Oh A, Klein WM. Addressing health-related misinformation on social media. *Jama*. 2018; *320*(23): 2417-2418. doi:10.1001/jama.2018.16865.

67. Stasiuk K, Polak M, Dolinski D, Maciuszek J. The credibility of health information sources as predictors of attitudes toward vaccination—the results from a longitudinal study in Poland. *Vaccines*. 2021; *9*(8):933 doi: 10.3390/vaccines9080933

68. Cernicova-Buca M, Palea A. An appraisal of communication practices demonstrated by romanian district public health authorities at the outbreak of the COVID-19 pandemic. *Sustainability*. 2021; *13*(5): 1-19, doi: 10.3390/su13052500

69. Tagliacozzo S, Albrecht F, Ganapati NE. International Perspectives on COVID-19 Communication Ecologies: Public Health Agencies' Online Communication in Italy, Sweden, and the United States. *Am Behav Sci*. 2021; *65*(7), 934-955 doi: 10.1177/0002764221992832

70. Saechang O, Yu J, Li Y. Public trust and policy compliance during the COVID-19 pandemic: The role of professional trust. *Healthcare*. 2021; 9 (2):1-13 doi: 10.3390/healthcare9020151.

71. Lewandowski R, Goncharuk AG, Cirella GT. Restoring patient trust in healthcare: medical information impact case study in Poland. *BMC Health Serv. Res.* 2021; *21*(1):1-11 doi: 10.1186/s12913-021-06879-2

72. Soveri A, Karlsson LC, Antfolk J, Lindfelt M, Lewandowsky S. Unwillingness to engage in behaviors that protect against COVID-19: the role of conspiracy beliefs, trust, and endorsement of complementary and alternative medicine. *BMC Public Health*. 2021; *21*(1): 1-12 doi: 10.1186/s12889-021-10643-w

73. Antinyan A, Bassetti T, Corazzini L, Pavesi F. Trust in the health system and COVID-19 treatment. Front. Psychol.. 2021; *12*:1-14 doi:10.3389/fpsyg.2021.643758

74. Law RW, Kanagasingam S, Choong KA. Sensationalist social media usage by doctors and dentists during Covid-19. *Digit. Health*. 2021; *7*:1-12 doi: 10.1177/20552076211028034

75. Leonard MB, Pursley DM, Robinson LA, Abman SH, Davis JM. The importance of trustworthiness: lessons from the COVID-19 pandemic. Pediatr. Res. 2021; 1-4 doi:10.1038/s41390-021-01866-z

76. Wood JL, Lee GY, Stinnett SS, Southwell BG. A Pilot Study of Medical Misinformation Perceptions and Training Among Practitioners in North Carolina (USA). *INQUIRY: The Journal of Health Care Organization, Provision, and Financing*, 2021; *58*:1-6 doi: 10.1177/00469580211035742

77. Gopichandran V, Sakthivel K. Doctor-patient communication and trust in doctors during COVID 19 times—A cross sectional study in Chennai, India. *Plos One*. 2021: *16*(6), 1-11 doi: 10.1371/journal.pone.0253497

78. Nwoga HO, Ajuba, MO, Ezeoke UE. Effect of COVID-19 on doctor-patient relationship. *Int J Community Med Public Health*. 2020; *7(12)*: 2394-6040, doi: 10.18203/2394-6040.ijcmph20205136

79. Rocha YM, de Moura GA, Desidério GA, de Oliveira CH, Lourenço FD, de Figueiredo NLD. The impact of fake news on social media and its influence on health during the COVID-19 pandemic: A systematic review. *J. Public Health*. 2021; 1-10 doi:10.1007/s10389-021-01658-z

80. Greene CM, Murphy G. Quantifying the effects of fake news on behavior: Evidence from a study of COVID-19 misinformation. *J Exp Psychol Appl*. 2021 Dec;27(4):773-784. doi: 10.1037/xap0000371.

81.Rogozea LM, Sechel G, Bularca MC, Coman C, Cocuz ME. Who's Getting Shots First? Dealing With the Ethical Responsibility for Prioritizing Population Groups in Vaccination. *Am J Ther*. 2021 Jun 22;28(4):e478-e487. doi: 10.1097/MJT.0000000000001400. PMID: 34228653.

82. Rogozea L, Purcaru D, Leaşu F, Nemet C. Biomedical research - opportunities and ethical challenges. *Rom J Morphol Embryol.* 2014;55(2 Suppl):719-22. PMID: 25178352.

83. Olimid AP, Rogozea LM, Olimid DA. Ethical approach to the genetic, biometric and health data protection and processing in the new EU General Data Protection Regulation (2018). *Rom J Morphol Embryol*. 2018;59(2):631-636. PMID: 30173275.

Supporting information

S1 Appendix English version of the questionnaire

(docx)

S2 Appendix Romanian version of the questionnaire

(docx)

S3 Tables with results to the 1st research question

S4 Tables with results to the 2nd research question

Formatted: Superscript

S5 Tables with results to the 3rd research question

Formatted: Superscript

S6 Tables with results to the 4th research question

S7 Tables with results to the 5th research question

Rebuttal letter

Claudiu Coman

Transilvania University of Brasov

claudiu.coman@unitbv.ro

Dear Sir/Madam

With this cover letter we submit the revised manuscript, initially entitled" Challenges in the communication process during the COVID-19 pandemic- a perspective of medical staff", and after complying with the suggestions of the reviewers, entitled "Misinformation about medication during the COVID – 19 pandemic: a perspective of medical staff" by Claudiu Coman, Maria Cristina Bularca, Angela Repanovici and Liliana Rogozea for publication in PLOS ONE.

We revised the manuscript according to the suggestions and recommendation made by the reviewers. We would like to thank the reviewers for taking time to review our paper and for providing such useful suggestions. We also thank the academic editor for reviewing our paper. We tried to comply with all the suggestions and recommendations made by the reviewers, and in this letter, we describe the changes we made to the text according to the recommendations of the reviewers.

Our manuscript needed major revisions. The changes were made while having active the "Track changes" function from Microsoft Word and the lines where the text was changed can be best viewed while having active the "All markup" option. Moreover, in order for our changes to be best seen, we will also provide in this cover letter, the lines from the revised manuscript with the "Track changes" function, and "All markup" option active. With regards to our response to Reviewer 1, the reviewer made a series of suggestions directly in the PDF version of our initial manuscript, but also provided a summary of those suggestions in the e-mail which was sent by the journal to the corresponding author. In this regard, we responded first to the comments highlighted in the summary from the e-mail, and then we responded to each point made by Reviewer 1 in the PDF version of our initial manuscript. Next, we responded to each point raised by Reviewer 2.

Our response to Reviewer 1:

We firstly thank the reviewer for taking time to review our manuscript and provide suggestions in order to improve it. We addressed all the suggestions made by the reviewer. When we describe how the text was changed, we also provide the lines where the text can be found in the revised manuscript with the option "Track changes" active. In this way, the changes can be viewed completely (the text we deleted, and the text we inserted). Next, we will firstly describe our answers to the comments which were summarized in the e-mail received by the corresponding author, and then we will present our responses to the comments made by the reviewer in the PDF version of our manuscript.

Reviewer 1 comments- as summarized in the email received by the corresponding author

Reviewer 1 point 1: the review comments attached. The required modifications can be summarized as following and the authors will find it in details in the attached file: the authors should review the journal guidelines and abide by it in manuscript preparation.

Response 1: We are grateful to the reviewer for the suggestion. We reviewed the guidelines of PLOS ONE journal again and we made sure our manuscript is prepared in accordance to the author guidelines which can be found on the journal's official website. We also checked the pdf files entitled "Download sample title, author list, and affiliation page" and "Download sample manuscript body", in order to make sure our manuscript is correctly formatted. Thus, we looked again at the guidelines for the sections which have to be included in the manuscript, the font and sizes for headings, table captions, referencing rules, etc., and we made sure our manuscript respects the guidelines of the journal.

Reviewer 1 point 2: the introduction section is too long and need to be summarized.

Response 2: We thank the reviewer for the useful suggestion. In order to comply with it, we tried to summarize our introduction. Thus, we would like to mention that we also took into account the comments the reviewer made in the pdf version of the manuscript. In this regard, there the reviewer recommended us to rephrase the first paragraph of our paper because the paragraph was not about the communication process: "the introductory paragraph is not related to communication process". We rephrased the paragraph and we added information in which we highlighted the fact that the COVID – 19 pandemic negatively influenced the communication process. The changes we made, the text deleted, added or rephrased can be best seen while having active the "Track changes" function and the "All markup" option provided by Microsoft Word. Thus, in the Introduction section of the paragraph, page 4 of the manuscript, lines 71-77, we made changes to the text, and the new introductory paragraph also addresses the subject of communication:

"The COVID 19 pandemic generated multiple changes in the way today's society members carry out their daily activities. One of the processes which was mostly affected by the pandemic was the communication process between institutions and the public, as well as between individuals. In this regard, from this perspective, while many domains were affected by the spread of the virus, such as the educational system or the cultural sector, the health sector was the one that faced the most challenges [1]."

Next, in the pdf version of our manuscript, the reviewer suggested that the details we gave regarding the virus could be summarized in one paragraph: "the history of covid-19 can be summarized in a single paragraph". In order to comply with the request, in the Introduction section, at page 4 of the manuscript, we summarized the text indicated by the reviewer.

The text the reviewer suggested us to summarize:

"Caused by severe acute respiratory syndrome coronavirus 2 (SARS-CoV-2) [2], the disease was firstly detected in December 2019, in Wuhan, China [3], and it fastly spread all over the world. The World Health Organization was informed about a pneumonia outbreak in Wuhan on December 31 2019, the number of cases continued to increase, and on March 11 2020 the World

Health Organization characterized COVID 19 as a pandemic [4]. Being highly contagious, the virus affected a large number of people, and as of November 27 over 61 million cases were reported [5]. Even though many companies and institutions are struggling to develop a vaccine, Pfizer, Gamaleya Research Institute, University of Oxford, and a preliminary analysis of the vaccine proposed by Pfizer showed that the vaccine is able to prevent more than 90% of people from getting infected with COVID 19 [6], so far no vaccine was approved as a general and universal vaccine against COVID 19 [7]. Ever since the pandemic was declared, finding the right treatment for the virus has become a priority for researchers and doctors from all over the world. In this regard, large number of trials started to be conducted, and in order to find an efficient drug treatment against the virus, one method that was adopted was testing and administrating to patients, drugs that were previously used for curing other viruses [8]. Thus, on March 20 2020, The World Health Organization launched the SOLIDARITY clinical trial, a trial that monitored the effects on patients infected with COVID 19, of specific drugs that proven to be effective in the treatment of other diseases: remdesivir, interferon beta, chloroquine and hydroxychloroquine -previously used for Malaria, as well as drugs used on HIV patients: lopinavir and ritonavir [9]. However, according to the interim results published on October 15 2020 by WHO, even though those drugs were taught to have positive effects on treating COVID 19, they had little influence or no influence at all on mortality in general, on the need and initiation of ventilation and on the recovery process [10]."
The way we summarized the text can be seen at lines 102-114- in the revised version of our manuscript (The full change, the text deleted and the text summarizes is visible at lines 78-114).

The text we summarized (lines 102 -114 with the "Track changes" and "All Markup" option active:

"Caused by severe acute respiratory syndrome coronavirus 2 [2], the disease was firstly detected in December 2019, in Wuhan, China [3]. Due to the evolution of the virus, the World Health Organization declared the pandemic in March 2020 [4], and as of November 27 over 61 million cases were reported [5]. In this regard, although several companies are struggling to develop a vaccine, and some of the proposed vaccines showed promising results [6], so far no vaccine was approved in order to be administrated to the entire population [7]. Ever since the pandemic was declared, many companies started to be preoccupied with finding a treatment, and one method used that was adopted was administrating to patients, drugs that were previously used for curing other viruses [8]. Thus, one of the most well - known trials started was the SOLIDARITY trial, which focused on using various drugs including chloroquine and hydroxychloroquine, lopinavir or ritonavir [9]. However, even if those drugs were taught to have positive effects on treating the virus, they did not have a significant influence on preventing mortality in general [10]".

Next, in order to reduce the information written in the Introduction section, as the reviewer suggested, we also deleted the last paragraph of the Introduction section, paragraph in which we provided details about the concepts that we addressed next in the Literature review section. Thus, at lines 118 – 122 in the revised manuscript with "Track changes" and "All Markup" option active, we deleted the following text:

"Hence, considering the purpose of our paper and the research questions, we believed it was necessary to analyze the literature on the drugs used to treat COVID – 19, on the role of social

media platforms in spreading fake information about the virus and potential treatments, and on the way the pandemic influenced the credibility of doctors and their relationship with their patients."

Reviewer 1 point 3: the section titles need to be reviewed and fixed.

Response 3: We thank the reviewer for the useful suggestion. We checked again the author guidelines provided by the journal on its official website, regarding sections of the manuscript. In this regard, we corrected the section which was entitled "Methods and materials" in the initial version of our manuscript, with the correct form, which is "Materials and methods". The change can be seen in the revised manuscript at page 17, line 364, while having active the "Track changes" and "All markup" options from Microsoft Word. We reviewed all of our section titles and made sure they are correct.

Reviewer 1 point 4: the resuklts section include too much tables need to be focusing on the most significant tables and attach the other tablesas supplementary tables.

Response 4: We are grateful to the reviewer for such useful suggestion. We addressed the suggestion, we looked at the tables included in the Results section and we integrated in the section only the most significant tables. The other tables were deleted from the text and added to supplementary information. Thus, we created Word documents with supplementary information for each of our research questions. In this regard in S3_Tables with results to the 1st research question we included Table 2 ; in S4_Tables with results to the 2nd research question we included Table 5 and Table 6; in S5_Tables with results to the 3rd research question we included Table 7 and Table 9; in S6_Tables with results to the 4th research question we included Table 11 and Table 12; in S7_Tables with results to the 5th research question we included Table 15 and Table 16.

Reviewer 1 point 5: the methods section is missing the research design, sampling method and the calculation of the study sample and the validity and reliability section.

Response 5: We are very grateful to the reviewer for suggesting us to improve the methods section of our paper. With regards to the research design section, we added this section to our manuscript and we explained in detail the research design. Even more, we deleted some information from the Sampling and data collection procedures and we added it to the research design section because it was more suitable there. In this regard, at pages 17-18 of the manuscript, between lines 365- 385 can be found the Research design section of our paper, which comprises the following text:

"The present study was conducted on Romanian healthcare professionals including doctors, nurses and medical students. The method used is quantitative. The questionnaire was administrated online, the data was collected through the help of Google forms, and was disseminated on groups of healthcare professionals and students on platforms such as Facebook and WhatsApp, during the period April 2021– June 2021. The data we collected was firstly exported to Microsoft Excel, and then it was analyzed with IBM Statistical Package for the Social Sciences, version 20. The respondents were informed about the purpose of the study, about the fact that they were allowed to withdraw at any time, and they were asked to give their consent for participating in the study. The average time needed to complete the questionnaire was 15 minutes. Considering the validity

of our research, we took into account the theoretical information from the literature regarding the development of a questionnaire. Our team of researchers together with health specialists have configured the dimensions, and operationalized the concepts in accordance with the theoretical approaches identified at the current stage of the research. Even more, we pre-tested the questionnaire before disseminating in order to guarantee the validity of the instrument. Thus, the questionnaire was completed by 50 respondents in the pre-testing stage. Considering the reliability of the research, we used split half reliability method. We split our sample in half, and we checked the variables in from our sub-samples in order to see if the variables provided convergent results. The convergent results we obtained by applying the split half method showed that we obtained a high fidelity measurement.

In order to create the research design section and to also improve the way our paper is structured, we made changes to the section "Sampling and data collection procedures". In this regard, we deleted some text and we reformulated some phrases. The section comprises the following text, which can be found at pages 17-18 of the revised manuscript with "Track changes" and "All markup" option active, lines 419-427:

"In order to conduct the research we used a quantitative method while having as an instrument a questionnaire. The responses were collected online, with the help of Google forms, and the questionnaire was self – administrated. The research received approval from The Council of the Faculty of Sociology and Communication, approval request Nr.378/30.03.2021. Taking into account the sampling method and the calculation of the study sample, we used random, probabilistic sampling method. We took into consideration specialists, physicians, and medical students from Brasov, and we applied the snowballing method in order to disseminate the questionnaire. The sample of our study comprises 536 respondents, and included doctors, nurses as well as medical students from Romania."

With regards to the sampling method, we would like to thank the reviewer for pointing out that we should give more information about the sampling procedure. Even though in the initial version of our manuscript we described the sample of our research, how the questionnaire was distributed and to whom, we added more specific information about the sampling method. Hence, at page 20 of the manuscript, lines 423 - 426, we explained that we used a random, probabilistic sampling method:

"Taking into account the sampling method and the calculation of the study sample, we used random, probabilistic sampling method. We took into consideration specialists, physicians and medical students from Brasov, and we applied the snowballing method in order to disseminate the questionnaire."

Reviewer 1 point 6: the conclusion section need to be summarized and conclude the main study findings and its significance.

Response 6: We are grateful to the reviewer for the suggestion. In order to comply with it we tried to summarize our Conclusions section, to highlight again the main findings of the research and the significance of our study. In this regard, the text which was written in Conclusions in the initial

version of our manuscript was improved. In this regard, we deleted some of the redundant information which was written in this section. The information we deleted:

“In this regard, besides fighting the pandemic, physician also had to fight the so called infodemic. Fake news spread on social media about various alternative treatments for the virus and the opinions of certain professionals about treatment methods which later proven to be inaccurate negatively influenced the credibility of doctors.” (Lines 789-792)

“This results can suggest that while professionals were aware of the role of social media in spreading medical misinformation and in affecting trust in doctors, due to their knowledge, at personal level they were less affected by that type of information, many of them believing that social media should also be used for sending official information” (lines 803-807)

“Moreover, the medical staff was aware of the alternative treatments which were promoted on social media, the method of drinking alcohol in order to prevent the infection being the method that most of the respondents have heard about” (lines 811-813).

“Hence, on the basis of the findings and implications of the study, we further discuss limitations and future research directions.” (Lines 838-839).

Next, we took into account the recommendation of the reviewer and we started the section by presenting the main findings of our research. Since we had several research questions, we presented our main findings in relation to those research questions. Next, the reviewer recommended us to explain the significance of our study. Thus, in the paper we had already written the theoretical and practical implication of our paper. In this regard, we did not delete the implications because we consider that the implications emphasize why the study conducted is important and how it can be further taken into consideration. Next, we did not delete the limitations and future research directions either, because we considered necessary to highlight how and why our study has limitations but also how it could be further developed or extended.

Reviewer 1 point 7: the references are too much need to be filtered and summarized to 30 or 40 refrences maximum. Regards

Response 7: We are very grateful to the reviewer for this recommendation and we appreciated the interest in improving our paper. However, when we started to write the article, we wanted to make sure our paper will be well documented and that it will address all the theoretical concepts and aspects needed. In this regard, we made a thorough research and literature review on the medication used in order to treat the virus, on the way social media contributed to the spread of misinformation about the virus, and on the way trust in doctors and the doctor- patient relation was affected during the pandemic. Thus, we read many research paper because we wanted for our paper to provide an overall view on the subject addressed. In this regard, we consider that all the references we used are relevant for the subject approached and for the research that we conducted, and therefore we could not delete more than half of them. In other words, through the references cited we support and sustain our arguments, we show how other researchers approached similar matters and thus we could not delete more than half of our references because we considered that by deleting them we could no longer have a strong and well consolidated theoretical background

and we could not properly explain how we wanted to address the matted of medical misinformation and its effects from the perspective of medical staff. Even more, the journal does not have a limitation regarding the length of the article or the number of references: “Manuscripts can be any length. There are no restrictions on word count, number of figures, or amount of supporting information”. In addition, we have seen articles which addressed subjects related to health and the COVID – 19 pandemic, and which were published in PLOS ONE, that have more than 40 references. For example, one article entitled “Severity of infection with the SARS- CoV -2 B1.1.7 lineage among hospitalized COVID – 19 patients in Belgium” **(**https://journals.plos.org/plosone/article?id=10.1371/journal.pone.0269138**),** has 76 references, and another article, entitled “The coronavirus disease 2019 (COVID -19) vaccination psychological antecedent assessment using the ARABIC 5c validated tool: An online survey in 13 Arab countries” **(**https://journals.plos.org/plosone/article?id=10.1371/journal.pone.0260321**)** has 71 references.

Reviewer 1 comments- as pointed by the reviewer in the PDF version of our manuscript

Reviewer 1 point 1: A perspective of medical staff

Response 1: We thank the reviewer for the suggestion. We put “:” instead of “-“in our title, before the phrase “a perspective of medical staff”. The change can be seen at line 2 of the revised manuscript.

Reviewer 1 point 2: the abstract need to be summarized to 250 to 300 words by the main important information in each part ....it is recommended to avoid long paragraphs and to paraphraze and summarize the ideas in short paragraphs.

Response 2: We are grateful to the reviewer for the recommendation. In order to comply with it we summarized our abstract to 219 words. In this regard, we deleted the text which was written in the Abstract section, and instead, at page 3 of the revised manuscript with “Track changes” and “All markup” option on, at lines 50 –68 we inserted the following text:

“Background. Healthcare professionals had to face numerous challenges during the pandemic, their professional activity being influenced not only by the virus, but also by the spread of medical misinformation. In this regard, we aimed to analyze, from the perspective of medical staff, the way medical and non - medical information about the virus was communicated during the pandemic in order to raise awareness about the way misinformation affected the medical staff.
Methods and findings. The study was conducted on Romanian healthcare professionals. They were asked to answer to a questionnaire and the sample of the research includes 536 respondents. The findings revealed that most respondents stated that information about alternative treatments against the virus affected the credibility of health professionals, and that younger professionals believed to a greater extent that trust in doctors was affected. The research also showed that respondents were well informed about the drugs used in clinical trials in order to treat the virus.
Conclusions. Healthcare professionals declared that the spread of misinformation regarding alternative treatments, affected their credibility and the relationship with their patients. Healthcare professionals had knowledge about the drugs used in clinical trials, and they acknowledged the

role of social media in spreading medical misinformation. However, younger professionals also believed that social media could be used to share official information about the virus."

Reviewer 1 point 3: the introductory paragraph is not related to communication process.

Response 3: We thank the reviewer for pointing this out. We explained how we addressed this point above in this Cover letter, in point 2 raised by the reviewer in the summary which was written in the e-mail sent to the corresponding author. However, we will present again the way we changed the introductory paragraph in order for it to be related to communication process. In this regards, in the Introduction section of the paragraph, page 4 of the manuscript with "Track changes" and "All markup active", lines 71-77, we made changes to the text, and the new introductory paragraph also addresses the subject of communication:

"The COVID 19 pandemic generated multiple changes in the way today's society members carry out their daily activities. One of the processes which was mostly affected by the pandemic was the communication process between institutions and the public, as well as between individuals. In this regard, from this perspective, while many domains were affected by the spread of the virus, such as the educational system or the cultural sector, the health sector was the one that faced the most challenges [1]."

Reviewer 1 point 4: the history of covid-19 can be summarized in a single paragraph.

Response 4: We are very grateful to the reviewer for the recommendation. We tried to comply with it and we summarized the history of COVID -19. Earlier in this cover letter we explained how we addressed this point because the reviewer also mentioned it in the summary which was written in the e-mail sent to the corresponding author. In this regard, we summarized the indicated text, and at page 5 of the manuscript with "Track changes" and "All markup" option active, lines 102-114 we added the following text:

"Caused by severe acute respiratory syndrome coronavirus 2 [2], the disease was firstly detected in December 2019, in Wuhan, China [3]. Due to the evolution of the virus, the World Health Organization declared the pandemic in March 2020 [4], and as of November 27 over 61 million cases were reported [5]. In this regard, although several companies are struggling to develop a vaccine, and some of the proposed vaccines showed promising results [6], so far no vaccine was approved in order to be administrated to the entire population [7]. Ever since the pandemic was declared, many companies started to be preoccupied with finding a treatment, and one method used that was adopted was administrating to patients, drugs that were previously used for curing other viruses [8]. Thus, one of the most well - known trials started was the SOLIDARITY trial, which focused on using various drugs including chloroquine and hydroxychloroquine, lopinavir or ritonavir [9]. However, even if those drugs were taught to have positive effects on treating the virus, they did not have a significant influence on preventing mortality in general [10]".

Reviewer 1 point 5: the stydy aim is to assess the perception and this other aim is not included as an intervention, so it is better to rephrased as to recommend future researches or interventions to raise......

Response 5: We thank the reviewer for the useful suggestion. We tried our best in addressing the recommendation. In this regard, we rephrased the part of the purpose indicated by the reviewer. In other words, the reviewer suggested us to rephrase the last part of our purpose, to rephrase the expression "in order to raise awareness about the way misinformation affected medical staff". Hence, at page 6 of the manuscript with "Track changes" and "All Markup" option active, lines 129 –133 we rephrased the purpose and added the following text:

"The purpose of the paper is to analyze, from the perspective of medical staff, the way medical and non - medical information about the virus was communicated during the pandemic to encourage the development of future research or interventions in order to raise awareness about the way misinformation affected medical staff."

Due to the suggestion of the reviewer, we had to change the way we described the purpose of our paper in other sections of our manuscript too. Thus, the purpose of the paper was changed in the way recommended by the reviewer, also at lines: 52 -55 (in the Abstract section).

Reviewer 1 point 6: please to consider the restructuring of the manuscript as per the journal guidelines and the title of each section. Also, the literature review section is very long and it should be fixed to bo not more than 2 to 2 and half pages summarizing the main ideas.

Response 6: We are very grateful to the reviewer for suggesting us to check again the guidelines of the journal. As we previously explained in this Cover letter, (due to the fact that the same point was also highlighted by the reviewer in the summary which was written in the e-mail sent to the corresponding author), we checked again the guidelines and made sure our manuscript is formatted according to the guidelines. We also checked again the titles of the section which should be included in the manuscript, and at page 17 of the revised manuscript with "Track changes" and "All markup" option active, line 364 we changed "Methods and materials" to "Materials and methods".
With regards to summarizing our Literature review and deleting references from our paper, we present again the explanation we gave earlier in the Cover letter, at point 7 made by the reviewer in the e-mail sent to the corresponding author:

We are very grateful to the reviewer for this recommendation and we appreciated the interest in improving our paper. However, when we started to write the article, we wanted to make sure our paper will be well documented and that it will address all the theoretical concepts and aspects needed. In this regard, we made a thorough research and literature review on the medication used in order to treat the virus, on the way social media contributed to the spread of misinformation about the virus, and on the way trust in doctors and the doctor- patient relation was affected during the pandemic. Thus, we read many research paper because we wanted for our paper to provide an overall view on the subject addressed. In this regard, we consider that all the references we used are relevant for the subject approached and for the research that we conducted, and therefore we could not delete more than half of them. In other words, through the references cited we support and sustain our arguments, we show how other researchers approached similar matters and thus we could not delete more than half of our references because we considered that by deleting them

we could no longer have a strong and well consolidated theoretical background and we could not properly explain how we wanted to address the matted of medical misinformation and its effects from the perspective of medical staff. Even more, the journal does not have a limitation regarding the length of the article or the number of references: “Manuscripts can be any length. There are no restrictions on word count, number of figures, or amount of supporting information”. In addition, we have seen articles which addressed subjects related to health and the COVID – 19 pandemic, and which were published in PLOS ONE, that have more than 40 references. For example, one article entitled “Severity of infection with the SARS- CoV -2 B1.1.7 lineage among hospitalized COVID – 19 patients in Belgium” (https://journals.plos.org/plosone/article?id=10.1371/journal.pone.0269138), has 76 references, and another article, entitled “The coronavirus disease 2019 (COVID -19) vaccination psychological antecedent assessment using the ARABIC 5c validated tool: An online survey in 13 Arab countries” (https://journals.plos.org/plosone/article?id=10.1371/journal.pone.0260321) has 71 references.

Reviewer 1 point 7: Research Design (please to review examples of the journal manuscript preparation)

Response 7: We thank the reviewer for pointing out that we should described more thoroughly the Research design of our paper. We explained how we addressed this suggestion earlier in this Cover letter, because the reviewer highlighted the suggestion in the summary from the e-mail sent to the corresponding author too. However, we will present again the way we complied with the suggestion. We did review examples of the journal manuscript preparation, and after we had done so, we deleted some text from the section “Sampling and data collection procedures” and moved it to the new section created. In this regard, at pages 17-18 of the revised manuscript with “Track changes” and “All markup” option active, lines 365-385, we inserted a sub-section entitled “Research design” which comprises the following text:

“The present study was conducted on Romanian healthcare professionals including doctors, nurses and medical students. The method used is quantitative. The questionnaire was administrated online, the data was collected through the help of Google forms, and was disseminated on groups of healthcare professionals and students on platforms such as Facebook and WhatsApp, during the period April 2021– June 2021. The data we collected was firstly exported to Microsoft Excel, and then it was analyzed with IBM Statistical Package for the Social Sciences, version 20. The respondents were informed about the purpose of the study, about the fact that they were allowed to withdraw at any time, and they were asked to give their consent for participating in the study. The average time needed to complete the questionnaire was 15 minutes. Considering the validity of our research, we took into account the theoretical information from the literature regarding the development of a questionnaire. Our team of researchers together with health specialists have configured the dimensions, and operationalized the concepts in accordance with the theoretical approaches identified at the current stage of the research. Even more, we pre-tested the questionnaire before disseminating in order to guarantee the validity of the instrument. Thus, the questionnaire was completed by 50 respondents in the pre-testing stage. Considering the reliability of the research, we used split half reliability method. We split our sample in half, and we checked

the variables in from our sub-samples in order to see if the variables provided similar results. The convergent results we obtained by applying the split half method showed that we obtained a high fidelity measurement."

Reviewer 1 point 8: methods and data (please to review the journal authors guideline).Also the reserch design is missed, please to clarify the research design used.

Response 8: We thank the reviewer for the suggestion. We reviewed again the journal author guidelines. Also, we added a research design section and the text contained in the section can be found at lines 365-385 of the manuscript with the "Track changes" and "All markup" option active.

Reviewer 1 point 9: start new sentence (line 333) in the PDF version of our manuscript

Response 9: We thank the reviewer for the recommendation. We complied with it and we started a new sentence, at page 17 of the manuscript with "Track changes" and "All markup" option active, lines 372 we deleted the words "At the beginning of the questionnaire", and we started a new sentence with "The respondents were informed…".

Reviewer 1 point 10: Also this section should not include the data interpretation or analysis. it should include only description.

Response 10: The reviewer referred to the "Sample and data collection procedure" section. We are grateful to the reviewer for the suggestion and in order to comply with it we made some changes to the text which was written in this section. In this regard, the data interpretation and analysis was removed from the section, and was moved to the "Results" section of our paper. The deleted text together with the table can be seen at lines 427 –444 of the revised manuscript with "Track changes" and "All markup" option active. The text we inserted in the "Results" section can be seen at lines 486-501 of the manuscript:

"Out of the 536 respondents, 460 (85.8%) were female and 76 (14.2%) were male. A total of 411 respondents live in the urban area (76.7%), while 125 (23.3%) live in the rural area. Most respondents (286, 53.4%) are between 18 and 35 years of age, 142 respondents (26.5%) are between 36 and 50 years of age, 102 respondents (19.0%) are between 51 and 65 years of age, and 6 of them (1.1) are over 65 years of age. When it comes to the professional degree of the respondents, most of them are students at a university nursing program (122, 22.8%), and medical students (120, 22.4%). However, a total of 102 respondents (19.0%) are senior specialists medical – doctors, and 70 (13.1%) are nurses who have a higher education diploma. When it comes to the respondents field of specialization, most of them (70.5%) operate in the field of general medicine, while others are family doctors (10.4%), pediatricians (3%), dentists or oncologists (1.9%), surgeons of doctors who are specialized in internal medicine (1.5%), or infectious disease doctors, radiologists or cardiologists (1.1%). Furthermore, most of the respondents (77.2%) stated that they did not work a unit with COVID – 19 patients while few of them (22.8%) stated that they worked in such a unit at the time the research was conducted. Thus, all the characteristics of the sample are presented in Table 1.

Table 1. Sample characteristics (n = 536).

	Category	Count	Percentage
Gender	Female	460	88.8%
	Male	76	14.2%
Living environment	Urban	411	76.7%
	Rural	125	23.3%
Age	18-35 years old	286	53.4%
	36-50 years old	142	26.5%
	51 -65 years old	102	19.0%
	Over 65 years old	6	1.1%
Professional degree	Senior specialist medical - doctor	102	19.0%
	Specialist medical - doctor	46	8.6%
	Resident	28	5.2%
	Nurse with higher education diploma	70	13.1%
	Nurse with other studies than higher education	48	9.0%
	Medical student	120	22.4%
	Student at university nursing program	122	22.8%
Field of specialization	General medicine	378	70.5%
	Family doctor	56	10.4%
	Pediatrics	16	3%
	Stomatology	10	1.9%
	Oncology	10	1.9%
	Surgery	8	1.5%
	Internal medicine	8	1.5%

	Virology/ infectious disease doctor	6	1.1%
	Cardiology	6	1.1%
	Radiology	6	1.1%
	Other	32	6%
Works in a unit with COVID – 19 patients	Yes	122	22.8%
	No	414	77.2%

”

Reviewer 1 point 11: please to explain how you calculated the sample size and the type of sampling that you used.

Response 11: We thank the reviewer for the suggestion. We offered an explanation for this point, which was also mentioned by the reviewer in the summary provided in the e-mail sent to the corresponding author. However, we will present again the explanation, which can be found at lines 413-416 of the manuscript with "Track changes" and "All markup" option active:

"Taking into account the sampling method and the calculation of the study sample, we used random, probabilistic sampling method. We took into consideration specialists, physicians and medical students from Brasov, and we applied the snowballing method in order to disseminate the questionnaire."

Reviewer 1 point 12: this section should be trasfered before data presentation and analysis with the methods part before data analysis

Response 12: The reviewer was referring to "The research instrument" section. We thank the reviewer for the suggestion. Since the section was already written before the "Data analysis" section, we moved the section before "Sampling and data collection procedures". The deleted text can be seen at lines 446-465 in the revised the manuscript with "Track changes" and "All markup" option active. The section was moved and so, the following text can be found in the revised manuscript at lines 387-406:

"In order to conduct the research we used a quantitative method while having a questionnaire as an instrument. In this regard, we developed a questionnaire which comprises four sections: A. Influence of the pandemic on the professional activity of medical staff (items A1 to A4), B. Perception about the authorities' communication process (items B1 to B11), C. Perception about the communication of non- validated treatments (items C1 to C20), and D. Sociodemographic questions (items D1 – D9), such as: gender, age, living environment, professional degree, field of specialization. The sociodemographic questions were used in order to identify different or similar attitudes between specific groups. The questionnaire can be found in "S1.Appendix English

version of the questionnaire", and in "S2. Appendix Romanian version of the questionnaire." Before disseminating the questionnaire, the instrument was tested on 30 doctors who work in the field of cardiology and general medicine. The respondents understood clearly the questions and did not report any issue in the process of answering them. Hence, the questionnaire comprises close ended and open ended questions (Items A1, A4, B3, B11, C19, C20, D2, D5, D6,) dihotomic questions as well as questions whose answers were measured on a 7 point Likert scale. For example, item A2 measured the extent to which the respondents considered that the pandemic influenced the way they carried out their professional activity (1- "to an extremely little extent, 7 "to an extremely great extent"), or item B2 measure the respondents' level of agreement with statements regarding the way authorities communicated during the pandemic (1 – "strongly disagree, 7-"strongly agree")."

Reviewer 1 point 13: the validity and reliability section is missed , please to discuss it clearly **Response 13:** We thank the reviewer for the recommendation. In order to address the recommendation, we inserted into our manuscript information about the validity and reliability of our research in the "Research design" section. In this regard, at page 18 of the manuscript with "Track changes" and "All markup" option active, lines 376 – 385, we inserted the following explanation:

"Considering the validity of our research, we took into account the theoretical information from the literature regarding the development of a questionnaire. Our team of researchers together with health specialists have configured the dimensions, and operationalized the concepts in accordance with the theoretical approaches identified at the current stage of the research. Even more, we pre-tested the questionnaire before disseminating in order to guarantee the validity of the instrument. Thus, the questionnaire was completed by 50 respondents in the pre-testing stage.
Considering the reliability of the research, we used split half reliability method. We split our sample in half, and we checked the variables in from our sub-samples in order to see if the variables provided similar results. The convergent results we obtained by applying the split half method showed that we obtained a high fidelity measurement."

Reviewer 1 point 14: you have two tables number by number 1 two times. please to review the tables numbering and indexing in the maneuscript.

Response 14: We are very grateful to the reviewer for pointing this out. We checked again all the numbers of the tables and corrected all the mistakes. Now in the revised manuscript, all the tables are correctly numbered.

Reviewer 1 point 15: these codes need to be interpretted (to give its full interpretaion under each table)

Response 15: We thank the reviewer for the suggestion. The reviewer was referring to the numbers of the questions which appear in the tables with correlations and t tests. Those numbers represent the number of the questions from the questionnaires which were included in the t tests or in the correlations. In other words, the numbers refer to the variables used in order to make the

tests and the correlations. For example, in Table 3, C14 means, the question 14 from the questionnaire, which belongs to section C. Section C refers to Perception about the communication of non- validated treatments. So, under each table from our manuscript (including the tables which we put in supplementary information) we added an explanation of the codes (numbers).

We would like to mention that the numbers of our tables changed, because in the initial manuscript we had two tables numbered 1, so now we corrected the mistake. Thus, we further present the explanation we gave in the revised manuscript with "Track changes" and "All markup" option active, under each table:

Table 3 (which was table 2 in the initial manuscript). The following explanation was added under the table: "[1] 1 C14 – refers to the question 14 from the section C of the manuscript (The extent to which information about alternative treatments affected trust in physicians), section which refers to Perception about the communication of non- validated treatments; [2]D2 - refers to question 2 from the D section of the manuscript (age), which refers to Sociodemographic characteristics of the respondents

Table 8 (which was Table 7 in the initial manuscript). The following explanation was added under the table "[1] B10- refers to the question 10 from the section B of the manuscript (Satisfaction with the way information about drugs used to treat the virus was communicated) section which refers to Perception about the authorities' communication process; [2]D2 - refers to question 2 from the D section of the manuscript (age), which refers to Sociodemographic characteristics of the respondents."

Table 13 (which was Table 12 in the initial manuscript). The following explanation was added under the table "[1] C1 – refers to question 1 from the section C of the manuscript (The extent to which social media represents an appropriate environment for sharing official COVID – 19 info), section which refers to Perception about the communication of non- validated treatments; [2]D2 - refers to question 2 from the D section of the manuscript (age), which refers to Sociodemographic characteristics of the respondents.

Table 16 (which was Table 15 in the initial manuscript and which is in Supplementary information - S7 Tables with results to the 5th research question). The following explanation was added under the table "[2]A3 – refers to question 3 from the section A of the manuscript (Main aspect of professional life influenced by the pandemic), section which refers to Influence of the pandemic on the professional activity of medical staff; The explanation for [1] *professional degree* was already written under the table in the initial version of our manuscript.

Reviewer 1 point 16: the variables need to be clear on the table

Response 16: We thank the reviewer for pointing this out. The reviewer was referring to the variables from the table which had the number 3 in the initial version of our manuscript. The table now has the number 4, because we corrected the way we numbered the tables. Hence, in order to be clear which the variables in the table are, we put the word "variables" in front of the variables which were tested. The changes to the table can be seen in the revised version of our manuscript with "Track changes" and "All markup" option active at page 29:

“**Table 4.** Significant t-test results: comparisons between variables

	t-test for Equality of Means										
	Group	N	Mean	S. D.	t	df	p	Mean Difference	Std. Error Difference	CI4	
										Lower	Upper
Variables: Information about alternative treatments _ Professional degree[1]	Medical staff	294	5.33	1.54	-2.04	534	.04	-.27	.13	-.52	-.01
	Student	242	5.60	1.49							
Variables: Information about alternative treatments _working unit	Unit with COVID -19 patients	122	5.19	1.61	-2.13	534	.03	-.33	.15	-.64	-.02
	Unit without COVID 19 patients	414	5.53	1.49							
Variables: Information about alternative treatments _gender	Male	76	5.10	1.70	-2.16	534	.03	-.40	.18	-.77	-.03
	Female	460	5.51	1.48							

[1]Index variable from the professional degrees of respondents. Student: medical student and student at university nursing program, Medical Staff: Senior specialist medical – doctor, Specialist medical – doctor, Resident, Nurse with higher education diploma, Nurse with other studies than higher education”

Reviewer 1 point 17: there keys need to be written in full interpretaion under each table.

Response 17: We thank the reviewer for the recommendation. We complied with it, and as we explained at one of the previous points of the reviewer, the keys (or codes) refer to the number of the question from the questionnaire, and the letter refers to the section of the questionnaire. Hence, the reviewer referred to the table which had the number 7 in the initial version of our manuscript. The table has the number 8 in the revised version of our manuscript with “Track changes” and “All markup” option active, because we corrected the way we numbered the tables. Under table 8, at page 33 of the manuscript we added the following explanation:

“[1] B10- refers to the question 10 from the section B of the manuscript (Satisfaction with the way information about drugs used to treat the virus was communicated) section which refers to Perception about the authorities’ communication process; [2]D2 - refers to question 2 from the D section of the manuscript (age), which refers to Sociodemographic characteristics of the respondents.”

Reviewer 1 point 18: the tables are too much, please to focus on the highly significant tables and add the others as a supplementary tables. it is recommended to reduce the number of tables to 5 or 6 tables

Response 18: We are very grateful to the reviewer for the useful suggestion. We complied with the suggestion and we deleted some tables from the manuscript and added them as supplementary information. Early in this Cover letter we provided an explanation for the tables, because this point was also included in the summary provided by the reviewer in the e-mail sent to the corresponding author. We let in the manuscript only the important tables: the tables with correlations and t tests, and the table with sociodemographic characteristics of the respondents. Thus, we presented again the explanation for the way we included the tables in supplementary information:

We created Word documents with supplementary information for each of our research questions. In this regard in S3_Tables with results to the 1st research question we included Table 1; in S4_Tables with results to the 2nd research question we included Table 4 and Table 5; in S5_Tables with results to the 3rd research question we included Table 6 and Table 8; in S6_Tables with results to the 4th research question we included Table 10 and Table 11; in S7_Tables with results to the 5th research question we included Table 14 and Table 15.

Reviewer 1 point 19: the conclusion section should be summarized to one paragraph summarize your important results and its significance and the future related researches

Response 19: We thank the reviewer very much for the recommendation. We answered to this point previously in this Cover letter, because the same point was also mentioned in the summary provided by the reviewer in the e-mail sent to the corresponding author by the journal ("the conclusion section need to be summarized and conclude the main study findings and its significance."). In this regard, we present again the redundant information we deleted from the Conclusions section, the way we highlighted the main results, their significance as well as the future research directions.

The information we deleted from the Conclusions section:

"In this regard, besides fighting the pandemic, physician also had to fight the so called infodemic. Fake news spread on social media about various alternative treatments for the virus and the opinions of certain professionals about treatment methods which later proven to be inaccurate negatively influenced the credibility of doctors." (Lines 789-792)

"This results can suggest that while professionals were aware of the role of social media in spreading medical misinformation and in affecting trust in doctors, due to their knowledge, at personal level they were less affected by that type of information, many of them believing that social media should also be used for sending official information" (lines 803-807)

"Moreover, the medical staff was aware of the alternative treatments which were promoted on social media, the method of drinking alcohol in order to prevent the infection being the method that most of the respondents have heard about" (lines 811-813).

“Hence, on the basis of the findings and implications of the study, we further discuss limitations and future research directions.” (Lines 838-839).

Next, we took into account the recommendation of the reviewer and we started the section by presenting the main findings of our research. Since we had several research questions, we presented our main findings in relation to those research questions. Next, the reviewer recommended us to explain the significance of our study. Thus, in the paper we had already written the theoretical and practical implication of our paper. In this regard, we did not delete the implications because we consider that the implications emphasize why the study conducted is important and how it can be further taken into consideration. Next, we did not delete the limitations and future research directions either, because we considered necessary to highlight how and why our study has limitations but also how it could be further developed or extended.

Reviewer 1 point 20: please to review your refrences and filter it to 30 to 40 refrences as 83 refrences are too much refrences

Response 20: We are very grateful to the reviewer for the suggestion and we understand the perspective of the reviewer. We would like to mention that we gave an explanation to this point early in this Cover letter, because the point was included in the summary which was sent by e-mail to the corresponding author. However, we insert again below the explanation for this point, explanations in which we show why we were unable to fully comply with the suggestion of the reviewer and delete more than half of our references:

We are very grateful to the reviewer for this recommendation and we appreciated the interest in improving our paper. However, when we started to write the article, we wanted to make sure our paper will be well documented and that it will address all the theoretical concepts and aspects needed. In this regard, we made a thorough research and literature review on the medication used in order to treat the virus, on the way social media contributed to the spread of misinformation about the virus, on the way misinformation influenced people’s confidence in the opinion of doctors and on the way the doctor- patient relation was affected during the pandemic. Thus, we searched and found many research papers and we reviewed all of them because we wanted for our paper to provide an overall view on the subject addressed. In this regard, all the references we used are relevant for the subject approached and for the research that we conducted. In other words, through the references cited we support and sustain our arguments, we show how other researchers approached similar matters and thus we could not afford to reduce them. By reducing them we could no longer have a strong and well consolidated theoretical background and we could not properly explain how we wanted to address the matter of medical misinformation and its effects from the perspective of medical staff. Even more, the journal does not have a limitation regarding the length of the article or the number of references: “Manuscripts can be any length. There are no restrictions on word count, number of figures, or amount of supporting information”. In addition, we have seen articles which addressed subjects related to health and the COVID – 19 pandemic, and which were published in PLOS ONE, that have more than 40 references. For example, one article entitled “Severity of infection with the SARS- CoV -2 B1.1.7 lineage among hospitalized COVID – 19 patients in Belgium” (https://journals.plos.org/plosone/article?id=10.1371/journal.pone.0269138), has 76 references,

and another article, entitled "The coronavirus disease 2019 (COVID -19) vaccination psychological antecedent assessment using the ARABIC 5c validated tool: An online survey in 13 Arab countries" (https://journals.plos.org/plosone/article?id=10.1371/journal.pone.0260321) has 71 references.

We would like to mention again that we did our best in trying to address all the suggestions of the reviewer and that we are thankful to the reviewer for all the points raised, for the time spent on analyzing our paper and for providing us very useful recommendations!

Response to reviewer 2

Reviewer 2 comment: The study is interesting and shows the point of view of health professionals, misinformation affected trustwith the patient. Another fact is that even among professionals, there are different perceptions about the spread of fakenews, according to age and occupation.

Response from authors: We are very grateful to the reviewer for his/hers kind words, and we appreciate the time the reviewer spent on reviewing our paper. We addressed all the recommendations of the reviewer and we will present each of the changes we made to the text. Before describing the way we addressed all the comments, we would like to mention that the changes can be best seen in the revised version of our manuscript, which has the "Track changes" and "All markup" options active.

Reviewer 2 comment 1: Thus, I suggest adequacy in the title, as it is not expressing exactly what the study observed.

Response 1: We thank the reviewer for the very useful suggestion. In order to comply with it, we changed the title of our manuscript in order for it to be more appropriate and more in line with the aim and the results of our study. In this regard, the new title of the manuscript is "Misinformation about medication during the COVID – 19 pandemic – a perspective of medical staff" (Lines 2-3). The title now highlights the fact that the study focused on misinformation about medication during the pandemic, and on the effects that misinformation had on doctors, from the perspective of specialists (doctors, nurses, medical students).

Reviewer 2 comment 2: The survey instrument was validated by a sufficient number of professionals; however, I did not find the attached instrument to be evaluated and to verify that the questions supported the statistical data that was generated. It is important to send supplementary material S1 so that the reviewer can evaluate the work impartially.

Response 2: We are very grateful to the reviewer for pointing this out. However, when we submitted the manuscript, we did upload the questionnaire as supplementary information, both in Romanian language and in English (S1_Appendix English version of the questionnaire; S2_Appendix Romanian version of the questionnaire).| In order to comply with the recommendation of the reviewer, we will try to upload again the questionnaire, and we will also insert it at the end of this document, so that the reviewer can have access to it. In this regard, the reviewer can find below the English and Romanian version of our questionnaire.

S1_Appendix English version of the questio

S2_Appendix Romanian version of t

Reviewer 2 comment 3: The Information on drugs used to treat COVID 19 topic of the Literature review covers the year 2020 and serves to locatethe context that health professionals were in at the time of answering the questionnaire, however, there is a lack ofinformation on the drugs that were being recommended by the WHO in the period of application of the questionnaire,which was from April to June 2021. Contextualizing how the data were in the period when the instrument was applied can directly impact the conclusion:"Healthcare professionals knew about the drugs used in clinical trials".

Response 3: We thank the reviewer for the useful suggestion. We searched for sources which contained information regarding the types of drugs available and approved in the period in which we conducted our research (April – June 2021) and we saw that among the drugs approved were also the drugs about which the respondents to our research had knowledge. Besides drugs, the news regarding the virus started to focus also on information about possible vaccines, so the information about antiviral drugs started to be published more rarely. Hence, our conclusion regarding the fact that "Healthcare professionals knew about the drugs used in clinical trials" is still true. Thus, we researched the literature and added an explanation in our Discussion section, but we did not insert the references into our paper, because Reviewer 1 mentioned that we have many references in our paper and that we should reduce them. However, Reviewer 2 can consult the references because we will insert them here after we provide the explanation. Hence, in the Discussion section of our manuscript, page 43, lines 753-760 we added the following explanation:

"Moreover, during the period in which we conducted our research, (April – June 2021), among the drugs which were approved were Remdesivir Tocilizumab – which was authorized first in June 2021, drug which were also acknowledged by the respondents of our research" [Reference 84, Reference 85)]."
Even more, one of the authors of the article (L.R.) is a doctor and was directly involved in the process of taking care of COVID – 19 patients, so the author can confirm that among the drugs which were in trial, or which were approved for administration against COVID-19 were also the drugs which were acknowledged by the respondents of our research.

Reference 84: Food and drug administration. Coronavirus (COVID-19) Drugs [Internet]. Food and Drug Administration. [cited 2022 June 20] Available from: https://www.fda.gov/drugs/emergency-preparedness-drugs/coronavirus-covid-19-drugs

Reference 85: Murdock, J. The Latest Updates on COVID-19 Treatments and Medications in the Pipeline. [Internet]. 23 May 2022 [cited 2022 June 20] Available from: https://www.goodrx.com/conditions/covid-19/coronavirus-treatments-on-the-way

Reviewer 2 comment 4: Minor revisions: When reading, there are differences in font size/type. E.g. lines 206 and. 534

Response 4: We thank the reviewer for pointing this out. We would firstly like to mention that line 206 has the number 245 in the revised version of the manuscript with “Track changes” and “All markup” option active, and line 534 has the number 645. In order to make sure there will no differences in font/size type, we checked again our manuscript and we corrected the mistakes. In this regard, we made sure the text from our manuscript is all formatted with Calibri, size 12.

We thank again the reviewer for spending time on reviewing our paper and for providing us very useful suggestions!

We are very grateful to the reviewers and the academic editor for all the suggestions, comments and points raised in order to improve our paper!

Sincerely,
Prof. Dr. Claudiu Coman
