# Supplementary material for: Misinformation about medication during the COVID– 19 pandemic: A perspective of medical staff
Source: PLoS One. 2022 Oct 27;17(10):e0276693. doi: 10.1371/journal.pone.0276693 (PMC9612566; doi:10.1371/journal.pone.0276693)
Supplement: Attachment — Submitted filename: Response to Reviewers..docx [file pone.0276693.s010.docx]

**Rebuttal letter**

Claudiu Coman

Transilvania University of Brasov

[claudiu.coman@unitbv.ro](mailto:claudiu.coman@unitbv.ro)

Dear Sir/Madam

With this cover letter we submit the revised manuscript, initially entitled” Challenges in the communication process during the COVID-19 pandemic- a perspective of medical staff”, and after complying with the suggestions of the reviewers, entitled “Misinformation about medication during the COVID – 19 pandemic: a perspective of medical staff” by Claudiu Coman, Maria Cristina Bularca, Angela Repanovici and Liliana Rogozea for publication in PLOS ONE.

We revised the manuscript according to the suggestions and recommendation made by the reviewers. We would like to thank the reviewers for taking the time to review our paper and for providing such useful suggestions. We also thank the academic editor for reviewing our paper. We tried to comply with all the suggestions and recommendations made by the reviewers, and in this letter, we describe the changes we made to the text according to the recommendations of the reviewers.

Our manuscript needed minor revisions. The changes were made while having active the “Track changes” function from Microsoft Word and the lines where the text was changed can be best seen while having active the “All markup” option. Moreover, in order for our changes to be best seen, we will also provide in this cover letter, the lines from the revised manuscript with the “Track changes” function, and “All markup” option active. With regards to our response to Reviewer 1, the reviewer made a series of suggestions directly in the PDF version of our revised manuscript. In this regard, we responded to the comments the reviewer made in the PDF version.

**Our response to Reviewer 1:**

We firstly thank the reviewer for taking time to review our manuscript again and provide suggestions in order to improve it. We addressed all the suggestions made by the reviewer. When we described how the text was changed, we also provide the lines where the text can be found in the revised manuscript with the option “Track changes” active. In this way, the changes can be seen completely (the text we deleted, and the text we inserted). Next, we present our response to the comments made by the reviewer in the PDF version of our manuscript.

**Reviewer 1 comments- made in the PDF version of our revised manuscript**

**Reviewer 1 comment 1:** Research design need to be separated in a separate line.

**Response 1:** We thank the reviewer for the suggestion. At line 307 in our revised manuscript the expression “Research design” was next to the title Materials and methods. In order to comply with the suggestion of the reviewer we put the expression “Research design” in a separate line below the title. In this regard, the change can be seen while having active the “Track changes” function and “All markup” option from Microsoft Word, at lines 307-308.

**Reviewer 1 comment 2:** what type of qualitative research design ?

**Response 2:** We thank the reviewer for pointing this out. However, we specified in the research design section that we did not use qualitative research design, but quantitative research design. The type of quantitative research design we used is descriptive. In this regard, we added some information in the subsection Research design, and we explained that we used descriptive quantitative research design. The change can be seen at line 310 in the revised manuscript with “Track changes” and “All Markup” function active:

“The method used is quantitative and descriptive”

**Reviewer 1 comment 3:** please to review and confirm that type of sampling method as it is used usually wit the qualitative data collection.

**Response 3:** We are thankful to the reviewer for pointing that we should check again if the sampling method is appropriate for the type of research design we used. We checked and reviewed again the type of sampling and we, the authors, made sure that the type of sampling method is appropriate for the research design we used, which is quantitative, not qualitative.

**Reviewer 1 comment 4**: the tables should be presented in sequence with numbering which is different from the supplementary tables numbering. please to review and fix it.

**Response 4:** We are grateful to the reviewer for the very useful suggestion. We made sure that the tables are numbered in sequence. In this regard, we renamed all the tables from our supplementary files in order for them not be confused with the numbers of the tables within the manuscript. We further describe each change that we made to the text while having active the “Track changes” function and the “All markup” option:

Because some data was missing from table 2 in our revised manuscript, we inserted again Table 2 within the text. The table was initially put into supplementary files, S3 Tables with results to the 1^st^ research question, but since we inserted it into the text again, we deleted the supplementary file called S3 Tables with results to the 1st research question. The insertion of the table can be found at line 406, page 20 of the revised manuscript and the change of deleting the supplementary file can be seen at line 1007 of the revised manuscript with “Track changes” function and “All Markup” option active.

Next,

Lines 452-453: S4_ Tables with results to the 2nd research question_Table 5 – became S3_ Tables with results to the 2nd research question_Table A

Lines 462 – 462: S4_ tables with results to the 2nd reseach question_Table 6 – became ( S3_ tables with results to the 2nd reseach question_Table B

Next, from the supplementary file entitled (S4_ Tables with results to the 3rd research question – we deleted the table that had the number 7 in our revised manuscript and we inserted it within the text. The inserted table can be found at line 477 in our revised manuscript, page 25.

Next, the table that had the number 8, now has the number 6 in order for the tables within our manuscript to be numbered in sequence. The change can be seen after the line 484.

Then, at lines 490- 491: S5_ Tables with results to the 3rd research question_Table 9 became S4_ Tables with results to the 3rd research question_Table C.

Then, the table that had the number 10, now has the number 7 in order for the tables within our manuscript to be numbered in sequence. The change can be seen at line 504.

Then, from S5 Tables with results to the 4th research question_ we deleted the table that had the number 11 in the supplementary file and we inserted it again into the manuscript. In this regard, the table received the number 8, in order for our tables to be numbered in sequence. The inserted table can be found at line 529 in our manuscript with “Track changes” and “All markup” option active.

Then, at line 534, S6 Tables with results to the 4th research question_Table 12 became S5 Tables with results to the 4th research question_Table D.

Then, the table that had the number 13, now has the number 9 in order for the tables within our manuscript to be numbered in sequence. The change can be seen at line 548.

Then, the table that had the number 14, now has the number 10 in order for the tables within our manuscript to be numbered in sequence. The change can be seen at line 562.

Then, at lines 573-574: S7 Tables with results to the 5th research question_Table 15 became S6 Tables with results to the 5th research question_Table E.

Then, at lines 580 – 581: S7 Tables with results to the 5th research question_Table 16 became S6 Tables with results to the 5th research question_Table F.

In this regard, due to the fact that we inserted into the manuscript some of the tables that were in the supplementary files in order for our tables to be numbered in sequence, our supplementary files changed:

S3 Tables with results to the 1st research question – was deleted completely

S4 Tables with results to the 2nd research question_ became S3 Tables with results to the 2nd research question

S5 Tables with results to the 3rd research question_ became S4 Tables with results to the 3rd research question_

S6 Tables with results to the 4th research question_ became S5 Tables with results to the 4th research question_

S7 Tables with results to the 5th research question_ became S6 Tables with results to the 5th research question

The changes can be seen at lines 1007 – 1011 in our revised manuscript with “Track changes” and “All markup” options active.

**Reviewer 1 comment 5:** is there any missed data?

**Response 5:** We are very grateful to the reviewer for pointing out that some data was missing from our tables. The reviewer was referring to Table 2 which Was at line 404 in the PDF version of our manuscript. We inserted again the table with all the data available. The inserted table can be seen between lines 406 – 407 with “Track changes” function and “All markup” option active.

| **Table 2.** The extent to which information about alternative treatments affected trust in physicians | | | | | |
| --- | --- | --- | --- | --- | --- |
|  | | Frequency | Percent | Valid Percent | Cumulative Percent |
| Valid | to an extremely little extent | 14 | 2.6 | 2.6 | 2.6 |
|  | to a very little extent | 10 | 1.9 | 1.9 | 4.5 |
|  | to a little extent | 42 | 7.8 | 7.8 | 12.3 |
|  | nor to a little, neither to a great extent | 58 | 10.8 | 10.8 | 23.1 |
|  | to a great extent | 114 | 21.3 | 21.3 | 44.4 |
|  | to a very great extent | 124 | 23.1 | 23.1 | 67.5 |
|  | to an extremely great extent | 174 | 32.5 | 32.5 | 100.0 |
|  | Total | 536 | 100.0 | 100.0 |  |

**Reviewer 1 comment 6:** is there any missed data?

**Response 6:** We are very grateful to the reviewer for pointing out that some data was missing from our tables. The reviewer was referring to the table which had the number 7 in our revised manuscript, which Was at line 474 in the PDF version of our manuscript. We inserted again the table with all the data available. The inserted table now has the number 5 so that our tables can be numbered in sequence and the table can be seen between lines 477 – 478 with “Track changes” function and “All markup” option active

| **Table 5.** The level of satisfaction with the way information about drugs used to treat the virus were communicated at national level | | | | | |
| --- | --- | --- | --- | --- | --- |
|  | | Frequency | Percent | Valid Percent | Cumulative Percent |
| Valid | extremely dissatisfied | 52 | 9.7 | 9.7 | 9.7 |
|  | very dissatisfied | 76 | 14.2 | 14.2 | 23.9 |
|  | dissatisfied | 110 | 20.5 | 20.5 | 44.4 |
|  | Nor dissatisfied, neither satisfied | 136 | 25.4 | 25.4 | 69.8 |
|  | satisfied | 108 | 20.1 | 20.1 | 89.9 |
|  | very satisfied | 30 | 5.6 | 5.6 | 95.5 |
|  | Extremely satisfied | 24 | 4.5 | 4.5 | 100.0 |
|  | Total | 536 | 100.0 | 100.0 |  |

**Reviewer 1 comment 7:** is there any missed dara?

**Response 7:** We are very grateful to the reviewer for pointing out that some data was missing from our tables. The reviewer was referring to the table which Had the number 11 in our revised manuscript, which was at line 525 in the PDF version of our manuscript. We inserted again the table with all the data available. The inserted table now has the number 8 so that our tables can be numbered in sequence and the table can be seen between lines 429 – 430 with “Track changes” function and “All markup” option active

| **Table 8.** Perception about the extent to which social media contributed to the spread of medical fake news | | | | | |
| --- | --- | --- | --- | --- | --- |
|  | | Frequency | Percent | Valid Percent | Cumulative Percent |
| Valid | to an extremely little extent | 2 | .4 | .4 | .4 |
|  | to a very little extent | 10 | 1.9 | 1.9 | 2.2 |
|  | to a little extent | 12 | 2.2 | 2.2 | 4.5 |
|  | nor to a little, neither to a great extent | 30 | 5.6 | 5.6 | 10.1 |
|  | to a great extent | 62 | 11.6 | 11.6 | 21.6 |
|  | to a very great extent | 88 | 16.4 | 16.4 | 38.1 |
|  | to an extremely great extent | 332 | 61.9 | 61.9 | 100.0 |
|  | Total | 536 | 100.0 | 100.0 |  |

**Reviewer 1 comment 8:** where is table 15?

**Response 8:** We thank the reviewer for the question. Table 15 was inserted as supplementary information. Due to the fact that we changes the numbers of the tables in order for the table within our manuscript to not be confused with the ones from the supplementary files, Table 15 is now Table E and can be found in the file entitled: S6 Tables with results to the 5th research question_Table E.

We are thankful to the reviewer for all the points raised, for the time spent on reviewing our paper and for providing us very useful suggestions!

**Our response to Reviewer 2:**

We are grateful to the reviewer for all the useful suggestions and we appreciate the time the reviewer spent on reviewing our paper. We addressed all the recommendations of the reviewer and we will present each of the changes we made to the text. Thus, we would like to mention that the changes can be best viewed in the revised version of our manuscript, which has the “Track changes” and “All markup” options active.

**Reviewer 2 comment 1 – as found in the decision letter received by e-mail by the corresponding author:** Lines 404, 474, 525 need to be revised because it has some formatting problem.

**Response 1:** We thank the reviewer for the very useful suggestion. In order to comply with it we checked the format of all the lines mentioned by the reviewer and we tried to fix them.

We thank again the reviewer for the kind words and for all the time she spent on analyzing our paper!

We are very grateful to the reviewers and to the academic editor for all the suggestions, comments and points raised in order to improve our paper!

Sincerely,

Prof. Dr. Claudiu Coman
